# Bioactive Triterpenoid Saponins From the Seeds of *Aesculus chinensis* Bge. var. *chekiangensis*

**DOI:** 10.3389/fchem.2019.00908

**Published:** 2020-01-23

**Authors:** Nan Zhang, Shuxiang Wei, Shijie Cao, Qiang Zhang, Ning Kang, Liqin Ding, Feng Qiu

**Affiliations:** ^1^School of Chinese Materia Medica, Tianjin University of Traditional Chinese Medicine, Tianjin, China; ^2^Tianjin State Key Laboratory of Modern Chinese Medicine, Tianjin University of Traditional Chinese Medicine, Tianjin, China

**Keywords:** *Aesculus chinensis* Bge. var. *chekiangensis* (Hu et Fang) Fang, phytochemistry, triterpenoid saponins, cytotoxic activities, neuroprotective activities

## Abstract

Phytochemical investigation of *Aesculus chinensis* Bge. var. *chekiangensis* (Hu et Fang) Fang obtained 33 triterpenoid saponins, including 14 new ones, aesculiside C–P (**1**–**14**). The structure elucidations were performed through comprehensive MS, 1D and 2D-NMR analysis, and their absolute configuration was unambiguously determined by X-ray diffraction analysis as well as Mo_2_(OAc)_4_-induced ECD method for the first time. All the substances were examined for their cytotoxic activities against three tumor cell lines, Hep G2, HCT-116, and MGC-803. Of these, compounds **8**, **9**, **14–16**, **18**, and **22** exhibited potent cytotoxicities against all cell lines with IC_50_ of 2–21 μM, while compounds **3**, **6**, **7**, **17–19**, **20**, **24**, and **28** depicted moderate activity (IC_50_ 13 to >40 μM). On these bases, the preliminary structure-activity correlations were also discussed. Meanwhile the neuroprotective properties of triterpenoid saponins from *Aesculus* genus were evaluated for the first time. Among them, compounds **1**, **4**, **12**, **20**, **22**, **25**, **29**, and **31** exhibited moderate activities against C_O_Cl_2_-induced PC12 cell injury.

## Introduction

*Aesculus chinensis* Bge. var. *chekiangensis* (Hu et Fang) Fang is a shrubby or small tree belonging to the *Hippocastanaceae* family which is widely distributed in China. The dry seeds of this plant, together with *Aesculus chinensis* Bge and *Aesculus wilsonii* Rehd, are the major sources of the traditional Chinese medicine “Suo Luo Zi.” Traditionally, it has been exploited to treat chest and abdominal pain, dysentery and ague (Yang et al., 1999a; Zhang et al., 2006). Earlier phytochemical study of *Aesculus chinensis* Bge. var. *chekiangensis* (Hu et Fang) Fang obtained various types of isolates, for example, triterpenoids (Yuan et al., 2013), flavonoids (Kapusta et al., 2007), coumarins (Niu et al., 2015) together with steroids (Zhang et al., 2009). Polyhydroxylated triterpenoid saponins, isolated from *Aesculus* genus (Wei et al., 2004; Kim et al., 2017) with great structural diversity, have been proved to be the major bioactive principles including anticancer (Patlolla et al., 2006), neuroprotective (Cheng et al., 2016), anti-inflammatory (Matsuda et al., 1997), antioxidative (Küçükkurt et al., 2010), and antiedematous activities (Piller, 1976). As part of our continuous research to screen cytotoxic and neuroprotective compounds of this type, a series of new triterpenoids (**1–14**) along with 19 reported analogs (**15–33**) from the seeds of *Aesculus chinensis* Bge. var. *chekiangensis* (Hu et Fang) Fang were obtained. Their cytotoxic activity and neuroprotective activity were also examined. Herein, the isolation, structural elucidation, cytotoxic activity, and neuroprotective activities of these isolates are described.

## Materials and Methods

### General Experimental Procedures

Optical rotations were recorded on a Rudolph (Hackettstown, NJ) Autopol V automatic polarimeter. The UV spectra were acquired on a UNICO 2102PCS spectrophotometer. The IR spectra were obtained in a KBr-disc (cm^−1^) on a Brucker Tensor II spectrometer. NMR spectra were carried out on a Bruker (Billerica, MA) AM-600 spectrometer at 25°C referencing to the residuals of pyridine-*d*_5_. High-Resolution-ESI-MS (HR-ESI-MS) was conducted on a Waters (Milford, MA) Xevo G2-S UPLC-Q/TOF equipped with an ACQUITY UPLC BEH C18 (2.1 × 50 mm, Waters 1.7 μm, USA). Analytical HPLC was performed on a Waters e2695 system equipped with a 2998 PDA detector using a YMC- Pack-ODS-A column (250 × 4.6 mm, 5 μm). Semi-preparative HPLC was performed using a Shimadzu LC-6AD Series instrument equipped with a YMC Packed C_18_ column (5 μm, 250 × 10.0 mm, YMC Co., Ltd., Kyoto, Japan) and detected with a DAD detector set at 205 and 230 nm. Column chromatography (CC) was done with Sephadex LH-20 (GE Healthcare Co. Ltd., USA), ODS RP-C_18_ (40–75 μm Merck Darmstadt, Germany), Macroporous resin D101 (Chemical Plant of Nankai University, Tianjin, China), Silica gel (200–400 mesh, Qingdao Haiyang Chemical, China). All reagents used were of analytical grade (Concord Technology Co. Ltd., Tianjin, China).

### Plant Material

Seeds of *Aesculus chinensis* Bge. var. *chekiangensis* (Hu et Fang) Fang were purchased from the Anguo Chinese medicine market (Hebei Province, P.R. China) in August 2015 and identified by professor Lijuan Zhang (Tianjin University of Traditional Chinese Medicine). The specimen was kept at the School of Chinese Materia Medica, Tianjin University of Traditional Chinese Medicine.

### Extraction and Isolation

The dried seeds of *A. chinensis* Bge. (8.8 kg) were extracted with 70% ethanol (10 L) under reflux for three times (3 h) at 70°C. After the solvent was removed under reduced pressure at <45°C, a dark residue (2,100 g) was obtained. The residue was adsorbed onto D101 resin and then sequentially eluted with H_2_O, a gradient of EtOH in water to give the corresponding fractions. The 60% EtOH–H_2_O part was chromatographed on silica gel, eluting with a gradient of 0–100% CH_2_Cl_2_/CH_3_OH to yield four fractions (A–D).

Fraction B (27.0 g) was separated by an RP C_18_ CC (MeOH–H_2_O, from 20:80 to 100:0) to give 10 subfractions B1–B10. Subfraction B4 was further purified by an RP-HPLC (MeCN–H_2_O, 40:60, 3.0 ml/min) to obtain compounds **4** (13.2 mg, *t*_R_ 21.2 min), **10** (15.6 mg, *t*_R_ 23.2 min), and **24** (20.6 mg, *t*_R_ 30.4 min). Further purification of Fr. B6 using preparative RP-HPLC (MeCN–H_2_O, 43:57, 3.0 ml/min) yielded compounds **1** (48.5 mg, *t*_R_ 16.5 min) and **3** (10.0 mg, *t*_R_ 19.2 min). Compounds **22** (11.1 mg, *t*_R_ 8.9 min), **26** (13.5 mg, *t*_R_ 14.7 min), **29** (32.0 mg, *t*_R_ 23.4 min), and **33** (17.6 mg, *t*_R_ 26.5 min) were obtained from Fr. B7 using a Sephadex LH-20 column and further purified by RP-HPLC (MeCN–H_2_O 45:55, v/v, 3.0 ml/min). Subfraction B10 was purified by preparative HPLC to afford compounds **2** (12.0 mg, *t*_R_ 26.5 min), **6–9** (9.7 mg, 21.3 mg, 15.5 mg, 22.7 mg; *t*_R_ 14.8 min, 16.6 min, 19,4 min, 21.2 min, respectively), and **14–16** (31.1 mg, 12.2 mg, 11.0 mg; *t*_R_ 31.2 min, 32.3 min, 33.1 min, respectively) using 50% MeCN/H_2_O.

Fraction C (12.0 g) was subjected to an ODS RP-C18 column (MeOH/H_2_O, 10:90 to 100:0, v/v) to give six subfractions (C1–C6). Compound **18** (25.3 mg, *t*_R_ 31.5 min) was purified by preparative HPLC using 30% MeCN/H_2_O from subfraction C1. Compounds **31** (11.9 mg, *t*_R_ 26.5 min) and **32** (39.6 mg, *t*_R_ 28.3 min) were gotten from C2 using the same preparative HPLC procedure with 32% MeCN/H_2_O. Fraction C4 was subjected to a Sephadex LH-20 column (MeOH) and then purified by recycling preparative HPLC with 33% MeCN/H_2_O to yield compounds **17** (9.8 mg, *t*_R_ 22.3 min), **19** (14.5 mg, *t*_R_ 24.1 min), and **28** (21.4 mg, *t*_R_ 25.9 min). Fraction C6 was separated using Sephadex LH-20 (MeOH) to obtain four subfractions (C6A–C6D). C6B and C6D were purified using preparative HPLC (35% MeCN/H_2_O) to yield compounds **20** (59.8 mg, *t*_R_ 25.4 min), **21** (40.7 mg, *t*_R_ 27.2 min), and **30** (21.4 mg, *t*_R_ 16.7 min).

Fraction D (9.0 g) was applied to an RP C_18_ CC eluting with gradient MeOH–H_2_O from 10:90 to 100:0 followed by a Sephadex LH-20 column (MeOH) to afford four major subfractions (D1–D4). Subfraction D1 was purified on an RP HPLC (MeCN–H_2_O 20:80, v/v, 3.0 ml/min) to afford compounds **2** (28.1 mg, *t*_R_ 19.8 min) and **5** (30.5 mg, *t*_R_ 22.4 min). Subfraction D2 was purified on an RP HPLC (MeCN–H_2_O 23: 77, v/v, 3.0 ml/min) to yield compounds **11** (46.0 mg, *t*_R_ 16.6 min) and **27** (41.8 mg, *t*_R_ 18.9 min). Compounds **12** (22.6 mg, *t*_R_ 14.7 min), **13** (16.0 mg, *t*_R_ 16.6 min), **23** (22.3 mg, *t*_R_ 17.7 min), and **25** (11.2 mg, *t*_R_ 18.3 min) were obtained by an RP HPLC (MeCN–H_2_O 25:75, v/v, 3.0 ml/min) from subfraction D4.

Aesculiside C (**1**), white amorphous powder; ${\left[\alpha \right]}_{\text{D}}^{\text{25}}$ − 6.0 (*c* 0.10, MeOH); ^1^H NMR and ^13^C NMR data (see Tables 1, **3**); HR-ESI-MS: *m/z* 1073.5149 [M–H]^−^ (calcd. for C_52_H_81_O_23_, 1073.5169).

**Table 1:** ^1^H NMR spectroscopic data (δ) for compounds **1**–**7**^a^ (δ in ppm, *J* in Hz).

**Proton**	**1**	**2**	**3**	**4**	**5**	**6**	**7**
**1**	0.81 *m*	0.84 *m*	0.85 *m*	0.81 *m*	0.83 *m*	0.74 *m*	0.74 *m*
	1.37 *m*	1.41 *m*	1.42 *m*	1.36 *m*	1.40 *m*	1.30 *m*	1.30 *m*
**2**	1.86 *m*	1.90 *m*	1.90 *m*	1.87 *m*	1.90 *m*	1.86 *m*	1.86 *m*
	2.19 *m*	2.19 *m*	2.20 *m*	2.16 *m*	2.17 *m*	2.23 *m*	2.23 *m*
**3**	3.26 (*dd*, 11.6, 4.3)	3.28 (*dd*, 11.7, 4.5)	3.25 (*dd*, 11.7, 4.5)	3.21 (*dd*, 11.6, 4.3)	3.25 (*dd*, 11.6, 4.3)	3.39 *m*	3.38 *m*
**5**	0.73 *m*	0.72 *m*	0.73 *m*	0.69 *m*	0.71 *m*	0.80 *m*	0.80 *m*
**6**	1.22 *m*	1.26 *m*	1.25 *m*	1.23 *m*	1.28 *m*	1.18 *m*	1.18 *m*
	1.44 *m*	1.46 *m*	1.47 *m*	1.45 *m*	1.47 *m*	1.49 *m*	1.49 *m*
**7**	1.26 *m*	1.28 *m*	1.30 *m*	1.26 *m*	1.28 *m*	1.22 *m*	1.23 *m*
	1.53 *m*	1.56 *m*	1.57 *m*	1.52 *m*	1.55 *m*	1.47 *m*	1.47 *m*
**9**	1.67 *m*	1.70 *m*	1.71 *m*	1.67 *m*	1.69 *m*	1.61 *m*	1.61 *m*
**11**	1.86 *m*	1.79 *m*	1.83 *m*	1.85 *m*	1.79 *m*	1.69 *m*	1.69 *m*
	1.91 *m*	1.87 *m*	1.90 *m*	1.91 *m*	1.87 *m*	1.80 *m*	1.80 *m*
**12**	5.47 *br s*	5.39 *br s*	5.41 *br s*	5.43 *br s*	5.38 *br s*	5.40 *br s*	5.40 *br s*
**15**	1.63 *m*	1.66 *m*	1.67 *m*	1.63 *m*	1.65 *m*	1.59 *m*	1.59 *m*
	1.89 *m*	1.98 *m*	1.99 *m*	1.89 *m*	1.98 *m*	1.82 *m*	1.82 *m*
**16**	4.76 *m*	4.89 *m*	4.89 *m*	4.72 *m*	4.88 *m*	4.48 *m*	4.48 *m*
**18**	2.85 (*dd*, 14.1, 4.6)	2.93 (*dd*, 14.3, 4.6)	2.98 (*dd*, 14.3, 4.6)	2.82 (*dd*, 14.0, 4.5)	2.93 (*dd*, 14.2, 4.5)	3.08 *m*	3.08 *m*
**19**	1.45 *m*	1.41 *m*	1.45 *m*	1.37 *m*	1.41 *m*	1.39 *m*	1.38 *m*
	3.11 (*t*, 13.5)	3.10 (*t*, 13.5)	3.12 (*t*, 13.5)	3.07 (*t*, 13.6)	3.09 (*t*, 13.6)	3.09 *m*	3.07 *m*
**21**	6.43 (*d*, 9.8)	6.41 (*d*, 10.0)	6.48 (*d*, 9.8)	6.39 (*d*, 9.8)	6.41 (*d*, 10.0)	6.61 (*d*, 10.1)	6.58 (*d*, 10.1)
**22**	4.45 (*d*, 9.8)	4.81 (*d*, 10.0)	4.88 (*d*, 10.1)	4.45 (*d*, 9.8)	4.81 (*d*, 10.0)	6.25 (*d*, 10.1)	6.30 (*d*, 10.1)
**23**	1.26 *s*	1.28 *s*	1.29 *s*	1.20 *s*	1.23 *s*	1.32 *s*	1.32 *s*
**24**	0.98 *s*	0.87 *s*	0.88 *s*	0.96 *s*	0.86 *s*	3.33 (*d*, 12.0)	3.31 (*d*, 11.3)
						4.25 (*d*, 12.0)	4.25 (*d*, 11.3)
**25**	0.83 *s*	0.83 *s*	0.84 *s*	0.83 *s*	0.83 *s*	0.64 *s*	0.64 *s*
**26**	1.07 *s*	1.10 *s*	1.10 *s*	1.05 *s*	1.08 *s*	0.78 *s*	0.78 *s*
**27**	1.84 *s*	1.86 *s*	1.87 *s*	1.82 *s*	1.85 *s*	1.81 *s*	1.81 *s*
**28**	4.21 (*d*, 10.3)	3.70 (*d*, 10.3)	3.72 (*d*, 10.4)	4.21 *m*	3.69 (*d*, 10.4)	3.37 *m*	3.40 *m*
	4.31 (*d*, 10.3)	3.98 (*d*, 10.3)	3.98 (*d*, 10.4)	4.31 *m*	3.97 (*d*, 10.4)	3.61 *m*	3.62 *m*
**29**	1.09 *s*	1.12 *s*	1.13 *s*	1.09 *s*	1.11 *s*	1.07 *s*	1.07 *s*
**30**	1.26 *s*	1.31 *s*	1.36 *s*	1.25 *s*	1.31 *s*	1.30 *s*	1.30 *s*
**C**_**3**_	GlcA-*p*	GlcA-*p*	GlcA-*p*	GlcA-*p*	GlcA-*p*	GlcA-*p*	GlcA-*p*
**1′**	4.98 (*d*, 7.1)	5.00 (*d*, 7.0)	4.99 (*d*, 8.1)	4.89 (*d*, 6.7)	4.97 (*d*, 6.7)	4.90 (*d*, 7.6)	4.90 (*d*, 7.4)
**2′**	4.31 *m*	4.36 (*dd*, 11.0, 7.9)	4.34 *m*	4.35 *m*	4.40 *m*	4.28 *m*	4.27 *m*
**3′**	4.33 *m*	4.38 *m*	4.36 *m*	4.06 *m*	4.09 (*t*, 8.5)	4.08 *m*	4.08 *m*
**4′**	4.56 *m*	4.61 *m*	4.58 (*t*, 9.0)	4.47 *m*	4.56 *m*	4.58 *m*	4.57 *m*
**5′**	4.59 *m*	4.65 *m*	4.61 *m*	4.54 *m*	4.63 *m*	4.59 *m*	4.58 *m*
**C_′_**	Gal-*p*	Gal-*p*	Gal-*p*	Glc-*p*	Glc-*p*	Glc-*p*	Glc-*p*
**1**″	5.22 (*d*, 7.7)	5.25 (*d*, 7.7)	5.25 (*d*, 7.5)	5.38 (*d*, 7.6)	5.43 (*d*, 7.6)	5.61 (*d*, 7.6)	5.61 (*d*, 7.6)
**2**″	4.51 (*t*, 8.0)	4.56 (*dd*, 9.6, 7.6)	4.55 (*t*, 8.4)	4.33 *m*	4.39 *m*	4.37 *m*	4.37 *m*
**3**″	4.15 *m*	4.19 *m*	4.21 *m*	4.23 *m*	4.26 *m*	4.20 *m*	4.19 *m*
**4**″	4.70 (*d*, 3.3)	4.71 (*d*, 3.3)	4.71 (*d*, 3.3)	4.17 *m*	4.18 *m*	4.20 *m*	4.20 *m*
**5**″	4.02 *m*	4.05 (*t*, 6.8)	4.05 *m*	3.90 *m*	3.93 (*dd*, 9.5, 3.7)	3.67 *m*	3.67 *m*
**6**″	4.38 (*dd*, 10.2, 4.2)	4.41 (*dd*, 10.6, 5.0)	4.40 (*dd*, 10.8, 4.8)	4.23 (*overlapped*)	4.27 (*dd*, 11.0, 5.0)	4.32 (*dd*, 11.2, 4.8)	4.31 (*d*, 11.2)
	4.59 (*t*, 10.4)	4.62 (*t*, 10.6)	4.62 (*t*, 10.8)	4.50 (*t*, 10.8)	4.53 (*overlapped*)	4.42 (*d*, 11.2)	4.41 (*dd*, 11.2, 5.2)
${\text{C}}_{4^{\text{′}}}$	Glc-*p*	Glc-*p*	Glc-*p*	Glc-*p*	Glc-*p*	Glc-*p*	Glc-*p*
**1**^**‴**^	5.20 (*d*, 7.9)	5.20 (*d*, 7.8)	5.21 (*d*, 7.7)	5.14 (*d*, 7.8)	5.20 (*d*, 7.8)	5.20 (*d*, 7.6)	5.21 (*d*, 7.6)
**2**^**‴**^	4.02 *m*	4.05 (*t*, 6.8)	4.05 *m*	4.02 *m*	4.05 (*t*, 7.8)	4.04 *m*	4.03 *m*
**3**^**‴**^	4.17 *m*	4.20 *m*	4.19 *m*	4.18 *m*	4.20 *m*	4.24 *m*	4.23 *m*
**4**^**‴**^	4.16 *m*	4.20 *m*	4.20 *m*	4.31 *m*	4.20 *m*	4.17 *m*	4.16 *m*
**5**^**‴**^	3.95 *m*	3.98 (*d*, 9.9)	3.96 *m*	3.94 *m*	3.99 *m*	3.98 *m*	3.97 *m*
**6**^**‴**^	4.23 (*t*, 9.6)	4.27 (*overlapped*)	4.27 *m*	4.45 (*d*, 10.8)	4.50 (*d*, 10.2)	4.47 (*d*, 10.2)	4.47 (*overlapped*)
	4.45 (*overlapped*)	4.49 (*dd*, 10.6, 5.0)	4.49 (*d*, 10.2)	4.52 (*overlapped*)	4.52 (*overlapped*)	4.49 (*overlapped*)	4.48 (*overlapped*)
**C**_**21**_	Ac	Ac	Tig	Ac	Ac	Ac	Ac
**2**^**′*′′′***^	2.11 *s*	2.09 *s*		2.07 *s*	2.09 *s*	2.06 *s*	1.92 *s*
**3**^**′*′′′***^			7.00 (*q*, 7.1)				
**4**^**′*′′′***^			1.61 (*d*, 7.0)				
**5**^**′*′′′***^			1.87 *s*				
**C**_**22**_ **or C**_**28**_	Ac	Ac		Ac	Ac	Tig	Ang
**2**^**′*′′′′***^	2.01 *s*			1.96 *s*			
**3**^**′*′′′′***^						6.99 (*q*, 6.9)	5.93 (*q*, 7.2)
**4**^**′*′′′′***^						1.47 (*d*, 6.9)	2.07 (*d*, 7.2)
**5**^**′*′′′′***^						1.87 *s*	2.09 *s*

a *NMR data (δ) were measured at 600 MHz in pyridine-d_5_ for **1**–**7***.

Aesculiside D (**2**), white amorphous powder; ${\left[\alpha \right]}_{\text{D}}^{\text{25}}$ − 6.0 (*c* 0.10, MeOH); ^1^H NMR and ^13^C NMR data (see Tables 1, **3**); HR-ESI-MS: *m/z* 1031.5067 [M–H]^−^ (calcd. for C_50_H_79_O_22_, 1031.5063).

Aesculiside E (**3**), white amorphous powder; ${\left[\alpha \right]}_{\text{D}}^{\text{25}}$ − 4.0 (*c* 0.09, MeOH); ^1^H NMR and ^13^C NMR data (see Tables 1, **3**); HR-ESI-MS: *m/z* 1071.5376 [M–H]^−^ (calcd. for C_53_H_83_O_22_, 1071.5376).

Aesculiside F (**4**), white amorphous powder; ${\left[\alpha \right]}_{\text{D}}^{\text{25}}$ + 10.0 (*c* 0.11, MeOH); ^1^H NMR and ^13^C NMR data (see Tables 1, **3**); HR-ESI-MS: *m/z* 1073.5153 [M–H]^−^ (calcd. for C_52_H_81_O_23_, 1073.5169).

Aesculiside G (**5**), white amorphous powder; ${\left[\alpha \right]}_{\text{D}}^{\text{25}}$ − 2.0 (*c* 0.11, MeOH); ^1^H NMR and ^13^C NMR data (see Tables 1, **3**); HR-ESI-MS: *m/z* 1031.5051 [M–H]^−^ (calcd. for C_50_H_79_O_22_, 1031.5063).

Aesculiside H (**6**), white amorphous powder; ${\left[\alpha \right]}_{\text{D}}^{\text{25}}$ − 14.0 (*c* 0.11, MeOH); ^1^H NMR and ^13^C NMR data (see Tables 1, **3**); HR-ESI-MS: *m/z* 1131.5586 [M + H]^+^ (calcd. for C_55_H_87_O_24_, 1131.5587).

Aesculiside I (**7**), white amorphous powder; ${\left[\alpha \right]}_{\text{D}}^{\text{25}}$ − 12.0 (*c* 0.10, MeOH); ^1^H NMR and ^13^C NMR data (see Tables 1, **3**); HR-ESI-MS: *m/z* 1129.5432 [M–H]^−^ (calcd. for C_55_H_85_O_24_, 1129.5431).

Aesculiside J (**8**), white amorphous powder; ${\left[\alpha \right]}_{\text{D}}^{\text{25}}$ − 7.0 (*c* 0.10, MeOH); ^1^H NMR and ^13^C NMR data (see Tables 2, 3); HR-ESI-MS: *m/z* 1171.5908 [M–H]^−^ (calcd. for C_58_H_91_O_24_, 1171.5900).

**Table 2:** ^1^H NMR spectroscopic data (δ) for compounds **8**–**14**^a^ (δ in ppm, *J* in Hz).

**Proton**	**8**	**9**	**10**	**11**	**12**	**13**	**14**
**1**	0.73 *m*	0.72 *m*	0.72 *m*	0.85 *m*	0.82 *m*	0.78 *m*	0.77 *m*
	1.29 *m*	1.29 *m*	1.29 *m*	1.39 *m*	1.37 *m*	1.33 *m*	1.34 *m*
**2**	1.88 *m*	1.84 *m*	1.85 *m*	1.96 *m*	1.95 *m*	1.92 *m*	1.94 *m*
	2.24 *m*	2.23 *m*	2.24 *m*	2.27 *m*	2.25 *m*	2.23 *m*	2.25 *m*
**3**	3.39 *m*	3.36 *m*	3.37 *m*	3.42 *m*	3.40 *m*	3.38 *m*	3.38 *m*
**5**	0.80 *m*	0.78 *m*	0.80 *m*	0.86 *m*	0.86 *m*	0.83 *m*	0.84 *m*
**6**	1.17 *m*	1.16 *m*	1.15 *m*	1.35 *m*	1.43 *m*	1.34 *m*	1.34 *m*
	1.47 *m*	1.46 *m*	1.48 *m*	1.62 *m*	1.61 *m*	1.59 *m*	1.59 *m*
**7**	1.23 *m*	1.19 *m*	1.20 *m*	1.26 *m*	1.27 *m*	1.23 *m*	1.24 *m*
	1.48 *m*	1.44 *m*	1.48 *m*	1.54 *m*	1.55 *m*	1.51 *m*	1.53 *m*
**9**	1.61 *m*	1.61 *m*	1.61 *m*	1.67 *m*	1.68 *m*	1.63 *m*	1.65 *m*
**11**	1.70 *m*	1.67 *m*	1.68 *m*	1.73 *m*	1.81 *m*	1.76 *m*	1.72 *m*
	1.81 *m*	1.80 *m*	1.81 *m*	1.85 *m*	1.86 *m*	1.81 *m*	1.84 *m*
**12**	5.42 *br s*	5.40 *br s*	5.34 *br s*	5.36 *br s*	5.46 *br s*	5.40 *br s*	5.39 *br s*
**15**	1.60 *m*	1.57 *m*	1.60 *m*	1.65 *m*	1.65 *m*	1.61 *m*	1.59 *m*
	1.83 *m*	1.79 *m*	1.91 *m*	1.96 *m*	1.94 *m*	1.86 *m*	1.84 *m*
**16**	4.47 *m*	4.43 *m*	4.82 *m*	4.87 *m*	4.81 *m*	4.70 *m*	4.48 *m*
**18**	3.08 *m*	3.09 *m*	2.89 *m*	2.93 *m*	2.87 *m*	2.88 *m*	3.10 *m*
**19**	1.37 *m*	1.35 *m*	1.37 *m*	1.41 *m*	1.42 *m*	1.35 *m*	1.41 *m*
	3.08 *m*	3.06 *m*	3.04 *m*	3.09 *m*	3.04 *m*	3.06 *m*	3.10 *m*
**21**	6.59 (*d*, 10.2)	6.61 (*d*, 10.1)	6.33 (*d*, 9.9)	6.39 (*d*, 9.9)	4.80 (*d*, 9.0)	6.37 (*d*, 9.8)	6.69 (*d*, 10.2)
**22**	6.28 (*d*, 10.2)	6.21 (*d*, 10.1)	4.76 (*d*, 9.9)	4.80 (*d*, 9.9)	4.40 (*d*, 9.0)	4.46 (*d*, 9.8)	6.32 (*d*, 10.2)
**23**	1.33 *s*	1.29 *s*	1.29 *s*	1.37 *s*	1.36 *s*	1.33 *s*	1.35 *s*
**24**	3.31 (*d*, 12.0)	3.32 (*d*, 12.0)	3.30 (*d*, 11.5)	3.51 (*d*, 11.6)	3.50 (*d*, 11.6)	3.47 (*d*, 11.6)	3.49 (*d*, 11.5)
	4.27 (*d*, 12.0)	4.23 (*d*, 12.0)	4.24 (*d*, 11.5)	4.34 (*d*, 11.6)	4.33 (*d*, 11.6)	4.29 (*d*, 11.6)	4.32 (*d*, 11.5)
**25**	0.65 *s*	0.63 *s*	0.62 *s*	0.73 *s*	0.75 *s*	0.71 *s*	0.71 *s*
**26**	0.79 *s*	0.77 *s*	0.76 *s*	0.82 *s*	0.96 *s*	0.91 *s*	0.80 *s*
**27**	1.83 *s*	1.81 *s*	1.80 *s*	1.85 *s*	1.86 *s*	1.81 *s*	1.84 *s*
**28**	3.37 *m*	3.33 *m*	3.63 *m*	3.67 *m*	4.23 *m*	4.18 *m*	3.39 *m*
	3.63 *m*	3.63 *m*	3.91 *m*	3.95 *m*	4.42 *m*	4.28 *m*	3.63 *m*
**29**	1.08 *s*	1.05 *s*	1.07 *s*	1.11 *s*	1.35 *s*	1.08 *s*	1.08 *s*
**30**	1.31 *s*	1.31 *s*	1.28 *s*	1.30 *s*	1.39 *s*	1.24 *s*	1.32 *s*
**C**_**3**_	GlcA-*p*	GlcA-*p*	GlcA-*p*	GlcA-*p*	GlcA-*p*	GlcA-*p*	GlcA-*p*
**1′**	4.89 (*d*, 6.7)	4.89 (*d*, 6.7)	4.87 (*d*, 7.6)	4.96 (*d*, 6.6)	4.94 (*d*, 6.6)	4.91 (*d*, 6.8)	4.91 (*d*, 6.8)
**2′**	4.28 *m*	4.26 *m*	4.27 *m*	4.39 *m*	4.38 *m*	4.34 *m*	4.36 *m*
**3′**	4.07 *m*	4.04 *m*	4.07 *m*	4.38 *m*	4.37 *m*	4.33 *m*	4.33 *m*
**4′**	4.57 *m*	4.55 *m*	4.54 *m*	4.59 (*t*, 8.5)	4.58 (*t*, 8.5)	4.53 (*t*, 7.9)	4.53 *m*
**5′**	4.58 *m*	4.58 *m*	4.55 *m*	4.64 (*d*, 9.7)	4.62 (*d*, 9.7)	4.58 (*d*, 9.7)	4.55 *m*
${\text{C}}_{2^{\text{′}}}$	Glc-*p*	Glc-*p*	Glc-*p*	Xyl-*p*	Xyl-*p*	Xyl-*p*	Xyl-*p*
**1^′′^**	5.61 (*d*, 7.2)	5.59 (*d*, 7.4)	5.60 (*d*, 7.8)	5.53 (*d*, 7.5)	5.51 (*d*, 6.3)	5.47 (*d*, 7.6)	5.49 (*d*, 6.1)
**2^′′^**	4.37 *m*	4.35 *m*	4.34 *m*	4.19 (*d*, 7.8)	4.19 *m*	4.14 *m*	4.16 *m*
**3^′′^**	4.20 *m*	4.18 *m*	4.19 *m*	4.01 *m*	4.00 *m*	4.09 *m*	4.11 *m*
**4^′′^**	4.19 *m*	4.17 *m*	4.19 *m*	4.42 *m*	4.41 *m*	4.37 *m*	4.38 *m*
**5^′′^**	3.66 *m*	3.65 *m*	3.67 (*d*, 9.7)	3.62 (*d*, 10.9)	3.61 (*d*, 9.5)	3.62 (*t like*, 9.5)	3.60 (*d*, 10.7)
				4.43 *m*	4.42 *m*	4.39 *m*	4.41 *m*
**6^′′^**	4.33 (*d*, 12.0)	4.29 (*dd*, 12.0, 5.2)	4.32 (*dd*, 10.2, 4.8)				
	4.42 (*d*, 12.0)	4.39 (*d*, 12.2)	4.42 (*t*, 10.2)				
${\text{C}}_{4^{\text{′}}}$	Glc-*p*	Glc-*p*	Glc-*p*	Glc-*p*	Glc-*p*	Glc-*p*	Glc-*p*
**1^′′′^**	5.21 (*d*, 6.7)	5.17 (*d*, 6.7)	5.20 (*d*, 6.7)	5.22 (*d*, 7.8)	5.22 (*d*, 7.8)	5.18 (*d*, 7.8)	5.19 (*d*, 7.8)
**2^′′′^**	4.03 *m*	4.02 *m*	4.03 *m*	4.06 (*d*, 7.9)	4.06 *m*	4.02 (*d*, 8.5)	4.03 *m*
**3^′′′^**	4.23 *m*	4.20 *m*	4.24 *m*	4.21 (*d*, 7.8)	4.21 *m*	4.17 *m*	4.22 *m*
**4^′′′^**	4.17 *m*	4.13 *m*	4.18 *m*	4.21 *m*	4.21 *m*	4.17 *m*	4.18 *m*
**5^′′′^**	3.97 *m*	3.95 *m*	3.96 *m*	4.13 (*dd*, 9.0, 3.5)	4.13 (*t*, 9.0)	3.96 *m*	3.97 *m*
**6^′′′^**	4.24 (*dd*, 11.6, 5.2)	4.45 (*t*, 12.2)	4.46 (*dd*, 11.8, 5.2)	4.28 (*dd*, 12.0, 5.8)	4.28 (*d*, 12.0)	4.24 (*dd*, 11.6, 5.8)	4.24 *m*
	4.48 (*d*, 11.6)	4.50 (*d*, 12.0)	4.49 (*dd*, 12.0, 5.2)	4.51 (*d*, 12.0)	4.51 (*d*, 11.6)	4.46 (*d*, 11.6)	4.46 *m*
**C**_**21**_	MB	IB	IB	Ac		Ac	Ang
**2^′′′′^**	2.47 *m*	2.61 *m*	2.65 *m*	2.09 *s*		2.06 *s*	
**3^′′′′^**	1.77 *m*	1.17 (*d*, 7.3)	1.22 (*d*, 7.0)				5.95 (*q*, 7.2)
	1.78 *m*						
**4^′′′′^**	0.92 (*t*, 7.4)	1.19 (*d*, 7.3)	1.18 (*d*, 6.9)				2.07 (*d*, 7.2)
**5^′′′′^**	1.19 (*d*, 7.0)						2.00 *s*
**C**_**22**_ **or C**_**28**_	Ang	Tig			Ac	Ac	Ang
**2^′′′′′^**					1.96 *s*	1.95 *s*	
**3^′′′′′^**	6.04 (*q*, 7.2)	6.97 (*q*, 6.8)					5.89 (*q*, 7.2)
**4^′′′′′^**	2.13 (*d*, 7.2)	1.48 (*d*, 6.8)					2.03 (*d*, 7.2)
**5^′′′′′^**	1.94 *s*	1.86 *s*					1.88 *s*

a *NMR data (δ) were measured at 600 MHz in pyridine-d_5_ for **8**–**14***.

**Table 3:** ^13^C NMR spectroscopic data (δ) for compounds **1**–**7**^a^ (δ in ppm).

**NO**.	**1**	**2**	**3**	**4**	**5**	**6**	**7**
**1**	38.7	38.7	38.6	38.7	38.6	38.7	38.7
**2**	26.5	26.6	26.5	26.5	26.4	26.8	26.8
**3**	89.1	89.2	89.1	89.2	89.2	91.4	91.4
**4**	39.4	39.5	39.4	39.4	39.4	43.9	43.9
**5**	55.6	55.7	55.6	55.6	55.6	56.4	56.4
**6**	18.3	18.4	18.3	18.3	18.3	18.8	18.8
**7**	33.0	33.1	33.0	33.0	33.0	33.5	33.5
**8**	39.9	40.0	39.9	40.0	39.9	40.2	40.2
**9**	46.8	46.9	46.8	46.8	46.8	47.0	47.0
**10**	36.6	36.7	36.6	36.6	36.6	36.6	36.6
**11**	23.8	23.8	23.7	23.8	23.7	24.3	24.3
**12**	123.9	123.3	123.3	123.9	123.2	123.5	123.5
**13**	142.7	143.5	143.4	142.7	143.4	143.1	143.1
**14**	41.7	41.8	41.7	41.7	41.7	41.9	41.9
**15**	34.6	34.4	34.3	34.6	34.3	35.1	35.1
**16**	67.5	67.8	67.8	67.5	67.7	68.7	68.9
**17**	46.9	48.1	48.1	46.9	48.0	48.5	48.3
**18**	40.5	40.4	40.3	40.5	40.3	40.3	40.3
**19**	47.2	47.8	47.7	47.2	47.7	47.5	47.5
**20**	35.9	36.1	36.3	35.9	36.0	36.4	36.6
**21**	81.5	81.9	81.9	81.5	81.8	79.6	79.7
**22**	70.7	72.8	72.8	70.7	72.7	74.3	73.9
**23**	28.0	28.0	27.9	28.0	27.9	22.7	22.8
**24**	16.9	16.7	16.8	16.9	16.6	63.6	63.6
**25**	15.6	15.7	15.6	15.6	15.5	15.8	15.8
**26**	16.6	16.9	16.6	16.6	16.7	17.0	17.0
**27**	27.4	27.4	27.3	27.4	27.3	27.8	27.8
**28**	66.3	66.0	65.8	66.3	65.9	63.7	63.9
**29**	29.7	29.8	29.8	29.7	29.7	29.7	29.8
**30**	19.9	20.2	20.2	19.9	20.1	20.3	20.4
**C**_**3**_	GlcA-*p*	GlcA-*p*	GlcA-*p*	GlcA-*p*	GlcA-*p*	GlcA-*p*	GlcA-*p*
**1′**	105.0	105.1	105.0	105.0	105.0	104.9	104.9
**2′**	82.2	82.3	82.1	81.0	80.9	79.9	80.0
**3′**	75.8	75.8	75.8	77.0	76.9	76.8	76.8
**4′**	81.9	81.8	81.9	82.1	82.1	82.2	82.2
**5′**	75.6	75.5	75.4	75.5	75.5	76.2	76.0
**6′**	171.9	172.2	171.9	172.1	172.1	172.7	172.6
${\text{C}}_{2^{\text{′}}}$	Gal-*p*	Gal-*p*	Gal-*p*	Glc-*p*	Glc-*p*	Glc-*p*	Glc-*p*
**1^′′^**	106.6	106.7	106.6	105.3	105.2	104.5	104.6
**2^′′^**	74.5	74.6	74.5	76.0	75.9	76.0	76.0
**3^′′^**	74.8	74.8	74.7	77.8	77.7	78.6	78.6
**4^′′^**	69.4	69.5	69.4	71.6	71.5	70.0	70.0
**5^′′^**	76.8	76.9	76.8	78.2	78.2	78.3	78.4
**6^′′^**	61.2	61.3	61.2	62.3	62.2	61.9	61.9
${\text{C}}_{4^{\text{′}}}$	Glc-*p*	Glc-*p*	Glc-*p*	Glc-*p*	Glc-*p*	Glc-*p*	Glc-*p*
**1^′′′^**	104.5	104.6	104.5	104.7	104.6	104.9	105.0
**2^′′′^**	74.7	74.8	74.7	74.8	74.7	75.2	75.2
**3^′′′^**	77.9	78.0	77.9	78.0	77.9	78.8	78.8
**4^′′′^**	71.4	71.5	71.4	71.4	71.4	71.8	71.8
**5^′′′^**	78.3	78.4	78.3	78.3	78.3	78.4	78.3
**6^′′′^**	62.3	62.4	62.3	62.7	62.6	62.6	62.7
**C**_**21**_	Ac	Ac	Tig	Ac	Ac	Ac	Ac
**1^′′′′^**	171.2	171.4	168.5	171.2	171.3	171.2	171.1
**2^′′′′^**	21.2	21.4	129.8	21.2	21.3	21.3	21.2
**3^′′′′^**			136.0				
**4^′′′′^**			14.0				
**5^′′′′^**			12.3				
**C**_**22**_ **or C**_**28**_	Ac			Ac		Tig	Ang
**1^′′′′′^**	170.6			170.6		168.7	168.5
**2^′′′′′^**	20.6			20.6		129.5	129.3
**3^′′′′′^**						137.5	137.5
**4^′′′′′^**						14.4	16.1
**5^′′′′′^**						12.6	21.4

a *NMR data (δ) were measured at 150 MHz in pyridine-d_5_ for **1**–**7***.

Aesculiside K (**9**), white amorphous powder; ${\left[\alpha \right]}_{\text{D}}^{\text{25}}$ − 9.0 (*c* 0.11, MeOH); ^1^H NMR and ^13^C NMR data (see Tables 2, 4); HR-ESI-MS: *m/z* 1159.5879 [M + H]^+^ (calcd. for C_57_H_91_O_24_, 1159.5900).

**Table 4:** ^13^C NMR spectroscopic data (δ) for compounds **8**–**14**^a^ (δ in ppm).

**NO**.	**8**	**9**	**10**	**11**	**12**	**13**	**14**
**1**	38.7	38.8	38.7	38.8	38.7	38.6	38.7
**2**	26.8	26.9	26.8	26.6	26.5	26.4	26.5
**3**	91.4	91.4	91.3	90.6	90.5	90.4	90.5
**4**	44.0	44.0	43.9	44.3	44.2	44.1	44.2
**5**	56.4	56.4	56.3	56.3	56.2	56.1	56.2
**6**	18.8	18.8	18.8	18.7	18.6	18.5	18.6
**7**	33.5	33.5	33.5	33.3	33.3	33.1	33.2
**8**	40.2	40.3	40.3	39.9	39.9	39.7	39.9
**9**	47.0	47.0	47.0	46.8	46.7	46.6	46.6
**10**	36.7	36.7	36.6	36.5	36.4	36.3	36.3
**11**	24.3	24.4	24.3	24.0	24.0	23.9	23.9
**12**	123.5	123.5	123.4	123.3	123.4	123.6	123.8
**13**	143.1	143.1	143.4	143.5	143.2	142.5	142.6
**14**	41.9	42.0	42.1	41.9	41.8	41.6	41.6
**15**	35.1	35.1	34.7	34.4	34.5	34.4	34.7
**16**	68.7	68.8	68.1	67.9	67.9	67.4	68.5
**17**	48.3	48.6	48.4	48.1	46.5	46.8	47.9
**18**	40.4	40.3	40.7	40.4	40.7	40.4	40.0
**19**	47.5	47.5	48.1	47.8	47.7	47.0	47.1
**20**	36.7	36.7	36.5	36.1	36.2	35.8	36.4
**21**	79.1	79.0	81.7	82.0	78.4	81.4	78.6
**22**	73.7	74.2	73.1	72.7	73.6	70.6	73.4
**23**	22.8	22.8	22.7	22.6	22.5	22.4	22.6
**24**	63.6	63.6	63.6	62.8	62.7	62.6	62.7
**25**	15.9	15.9	15.8	15.5	15.4	15.3	15.4
**26**	17.0	17.0	17.0	16.8	16.8	16.7	16.6
**27**	27.8	27.8	27.7	27.4	27.4	27.2	27.5
**28**	63.8	63.8	66.2	65.9	66.9	66.1	63.4
**29**	29.9	29.8	29.7	29.8	30.0	29.6	29.5
**30**	20.5	20.4	20.5	20.2	19.8	19.8	20.2
**C**_**3**_	GlcA-*p*	GlcA-*p*	GlcA-*p*	GlcA-*p*	GlcA-*p*	GlcA-*p*	GlcA-*p*
**1′**	104.9	105.0	104.9	104.8	104.8	104.8	104.8
**2′**	80.0	80.0	80.0	78.7	78.6	78.5	78.6
**3′**	76.8	76.8	76.8	76.3	76.3	76.1	76.4
**4′**	82.2	82.4	82.4	82.5	82.5	82.3	82.7
**5′**	76.0	76.1	76.1	75.6	75.6	75.5	75.6
**6′**	172.1	172.8	172.6	172.1	172.1	172.1	172.1
${\text{C}}_{2^{\text{′}}}$	Glc-*p*	Glc-*p*	Glc-*p*	Xyl-*p*	Xyl-*p*	Xyl-*p*	Xyl-*p*
**1^′′^**	104.6	104.6	104.5	104.7	104.5	104.5	104.6
**2^′′^**	76.0	76.1	76.0	75.7	75.6	75.6	75.6
**3^′′^**	78.6	78.7	78.3	78.5	78.5	78.3	78.4
**4^′′^**	70.1	70.1	70.0	70.8	70.7	70.6	70.7
**5^′′^**	78.4	78.4	78.6	67.1	67.1	66.9	67.1
**6^′′^**	61.9	61.9	61.8				
${\text{C}}_{4^{\text{′}}}$	Glc-*p*	Glc-*p*	Glc-*p*	Glc-*p*	Glc-*p*	Glc-*p*	Glc-*p*
**1^′′′^**	105.1	105.0	105.0	104.8	104.8	104.8	104.7
**2^′′′^**	75.2	75.2	75.2	74.9	74.9	74.7	74.8
**3^′′′^**	78.8	78.8	78.8	78.1	78.4	78.3	78.4
**4^′′′^**	71.8	71.8	71.7	71.5	71.4	71.2	71.3
**5^′′′^**	78.3	78.4	78.4	78.5	78.0	78.0	77.9
**6^′′′^**	62.6	62.7	62.6	62.3	62.2	62.1	62.2
**C**_**21**_	MB	IB	IB	Ac		Ac	Ang
**1^′′′′^**	176.6	177.0	177.6	171.5		171.1	167.6
**2^′′′′^**	42.1	35.2	35.1	21.4		21.1	128.9
**3^′′′′^**	27.4	19.8	19.9				137.1
**4^′′′′^**	12.3	19.5	19.5				15.8
**5^′′′′^**	17.2						21.0
**C**_**22**_ **or C**_**28**_	Ang	Tig			Ac	Ac	Ang
**1^′′′′′^**	168.3	168.7			170.7	170.4	168.0
**2^′′′′′^**	129.0	129.5			20.6	20.5	128.9
**3^′′′′′^**	139.0	137.7					137.0
**4^′′′′′^**	16.3	14.4					15.7
**5^′′′′′^**	21.2	12.7					20.8

a *NMR data (δ) were measured at 150 MHz in pyridine-d_5_ for **8**–**14***.

Aesculiside L (**10**), white amorphous powder; ${\left[\alpha \right]}_{\text{D}}^{\text{25}}$ − 16.0 (*c* 0.12, MeOH); ^1^H NMR and ^13^C NMR data (see Tables 2, 4); HR-ESI-MS: *m/z* 1077.5471 [M + H]^+^ (calcd. for C_52_H_85_O_23_, 1077.5482).

Aesculiside M (**11**), white amorphous powder; ${\left[\alpha \right]}_{\text{D}}^{\text{25}}$ + 12.0 (*c* 0.10, MeOH); ^1^H NMR and ^13^C NMR data (see Tables 2, 4); HR-ESI-MS: *m/z* 1017.4881 [M–H]^−^ (calcd. for C_49_H_77_O_22_, 1017.4906).

Aesculiside N (**12**), white amorphous powder; ${\left[\alpha \right]}_{\text{D}}^{\text{25}}$ + 4.0 (*c* 0.10, MeOH); ^1^H NMR and ^13^C NMR data (see Tables 2, 4); HR-ESI-MS: *m/z* 1017.4890 [M–H]^−^ (calcd. for C_49_H_77_O_22_, 1017.4906).

Aesculiside O (**13**), white amorphous powder; ${\left[\alpha \right]}_{\text{D}}^{\text{25}}$ + 8.0 (*c* 0.10, MeOH); ^1^H NMR and ^13^C NMR data (see Tables 2, 4); HR-ESI-MS: *m/z* 1059.4995 [M–H]^−^ (calcd. for C_51_H_79_O_23_, 1059.5012).

Aesculiside P (**14**), white amorphous powder; ${\left[\alpha \right]}_{\text{D}}^{\text{25}}$ − 4.2 (*c* 0.10, MeOH); ^1^H NMR and ^13^C NMR data (see Tables 2, 4); HR-ESI-MS: *m/z* 1141.5785 [M + H]^+^ (calcd. for C_57_H_89_O_23_, 1141.5795).

### Hydrolysis and Determination of Absolute Configuration of Sugars

A solution of **1–14** (1.0 mg, respectively) in 2 M HCl (4.0 ml) was heated at 90°C for 2 h. The reaction mixture was extracted with EtOAc (2 × 4 ml), and the aqueous phase was evaporated to dryness using a stream of N_2_. The residues and authentic sugar samples (d/l-galactose, d/l-glucose, d/l-xylose, and d-glucuronic acid) were, respectively, dissolved in pyridine (1.0 ml) containing L-cysteine methyl ester (1.0 mg) and heated at 60°C for 1 h, and then o-tolyisothiocyanate (1.0 ml) was added to the mixture and heated further for 1 h. Then each reaction mixture was analyzed by the Waters e2695 HPLC system using a 2998 PDA detector (at 250 nm). Analytical HPLC was performed on the YMC- Pack-ODS-A column (250 × 4.6 mm, 5 μm) eluting with A (0.1% formic acid): B (acetonitrile) = 80:20 (v/v) at 1.0 ml/min. The absolute configuration of sugars in each compound was established by a comparison of the retention times with the standards where the time differences (Δδ d-l) of one kind of sugar were sufficient to distinguish between d- and l-enantiomers (Tanaka et al., 2007; Zhang N. et al., 2018).

### Preparation of the Aglycone of Compound 14

Compound **14** (15.0 mg) in 2 M HCl (10.0 ml) was heated at 50°C for 4 h. The reaction mixture was extracted with EtOAc (2 × 10 ml), and the EtOAc phase was evaporated to dryness using a stream of N_2_. The residue was dissolved in THF (2.0 ml) and MeOH (1.00 ml), then NaOMe (2.00 mg, 2.2 eq) was added to the solution at 0°C. The mixture was stirred at 25°C for 4 h. The reaction was diluted with H_2_O (10.0 ml) and the mixture was extracted with ethyl acetate (3 × 3.00 ml). The ethyl acetate fraction was purified by a semipreparative RP HPLC (CH_3_CN–H_2_O, 45:65) to gain compound **14a**.

### Determination of the Absolute Configuration of the 21, 22-Diol Moieties in Compound 14a

First, Mo_2_(AcO)_4_ (1.0 mg) dissolved in DMSO (1.0 ml) was subjected to ECD measurement as blank control. Then compound **14a** (0.5 mg) and Mo_2_(AcO)_4_ (1.0 mg) were added to DMSO (1.0 ml) and scanned directly. The CD spectrum was recorded every 10 min until the Mo_2_(AcO)_4_-induced circular dichroism spectrum was stationary. The inherent ECD spectrum of **14a** was subtracted. The absolute configuration was elucidated by the diagnostic band at approximately 310–340 nm in the induced ECD spectrum.

### X-Ray Crystallographic Analysis of 14a

Single crystals of **14a** were obtained from CH_3_OH and H_2_O. The intensity data of **14a** was collected on a SuperNova, Dual, Cu at zero, AtlasS2 diffractometer at 100.00(11) K. The structure was elucidated with the SHELXS method and refined based on full-matrix least-squares on F2 using SHELXL-2018/3 (Sheldrick, 2015). Crystal data for **14a**: orthorhombic, space group P2_1_2_1_2 (no. 18), *a* = 11.7057(7) Å, *b* = 34.214(3) Å, *c* = 14.7361(9) Å, *V* = 5901.7(7) Å^3^, *Z* = 8, *T* = 100.00(11) K, μ(Cu Kα) = 0.617 mm^−1^, *D*_*calc*_ = 1.141 g/cm^3^, 20,468 reflections measured (5.166° ≤ 2Θ ≤ 148.994°), 10,735 unique (*R*_int_ = 0.0506, R_sigma_ = 0.0834) which were used in all calculations. The final *R*_1_ was 0.0837 (I > 2σ(I)) and *wR*_2_ was 0.2452 (all data). The crystallographic data of **14a** have been deposited at the Cambridge Crystallographic Data Centre (CCDC 1957449) and the data can be obtained from supporting information (Data Sheet 1_v1).

### Cytotoxicity Assay

The *in vitro* cytotoxicity of compounds **1**–**33** was measured by MTT assay (El-Readi et al., 2013; Xia et al., 2015) with 5-fluorouracil as the positive control. The human cancer cell lines, HepG2, HCT-116, and MGC-803 were purchased from ATCC. The tested cell lines were seeded in 96-well plates, and the plates were then incubated in a 37°C incubator containing 5% CO_2_ for 24 h. Subsequently, the tested compounds in DMSO were added to designated wells at a dosage of 3.125–50 μM. After 24 h, MTT was added to the culture medium and the absorbance at 490 nm was measured using a microplate reader.

### Neuroprotective Effect Assay

The neuroprotective effects of compounds **1**–**33** were tested against C_O_Cl_2_-induced PC12 cell injury (Zou et al., 2002) with MTT method. Rat pheochromocytoma cell line (PC12) was cultured in 96-well plates with RPMI-1640 supplemented with 10% (v/v) inactivated fetal bovine serum and 100 U/ml penicillin/streptomycin. The cells were maintained at 37°C in 5% CO_2_ and 95% humidified air incubator. Cells were pre-treated for 2 h with or without compounds before incubation in a medium containing 1 mM CoCl_2_. After 24 h, MTT was added to the culture medium, and the absorbance at 490 nm was measured using a microplate reader.

## Results

For the target of isolation of triterpene saponins, the 70% ethanol extracts of air-dried seeds of *Aesculus chinensis* Bge. var. *chekiangensis* (Hu et Fang) Fang were chromatographed through a D101 column and eluted with a gradient of EtOH in water. The 60% EtOH part was separated consequently as it contains abundant triterpene saponins under the guidance of UPLC-Q/TOF-MS. Thereafter, 14 undescribed triterpenoid saponins (**1–14**, aesculiside C–P) (Figure 1) and 19 known analogs (**15–33**) were afforded and identified (Figures S1–S157). The full assignments of the NMR data of compounds **1**–**14** are recorded in Tables 1–4.

**Figure 1:**
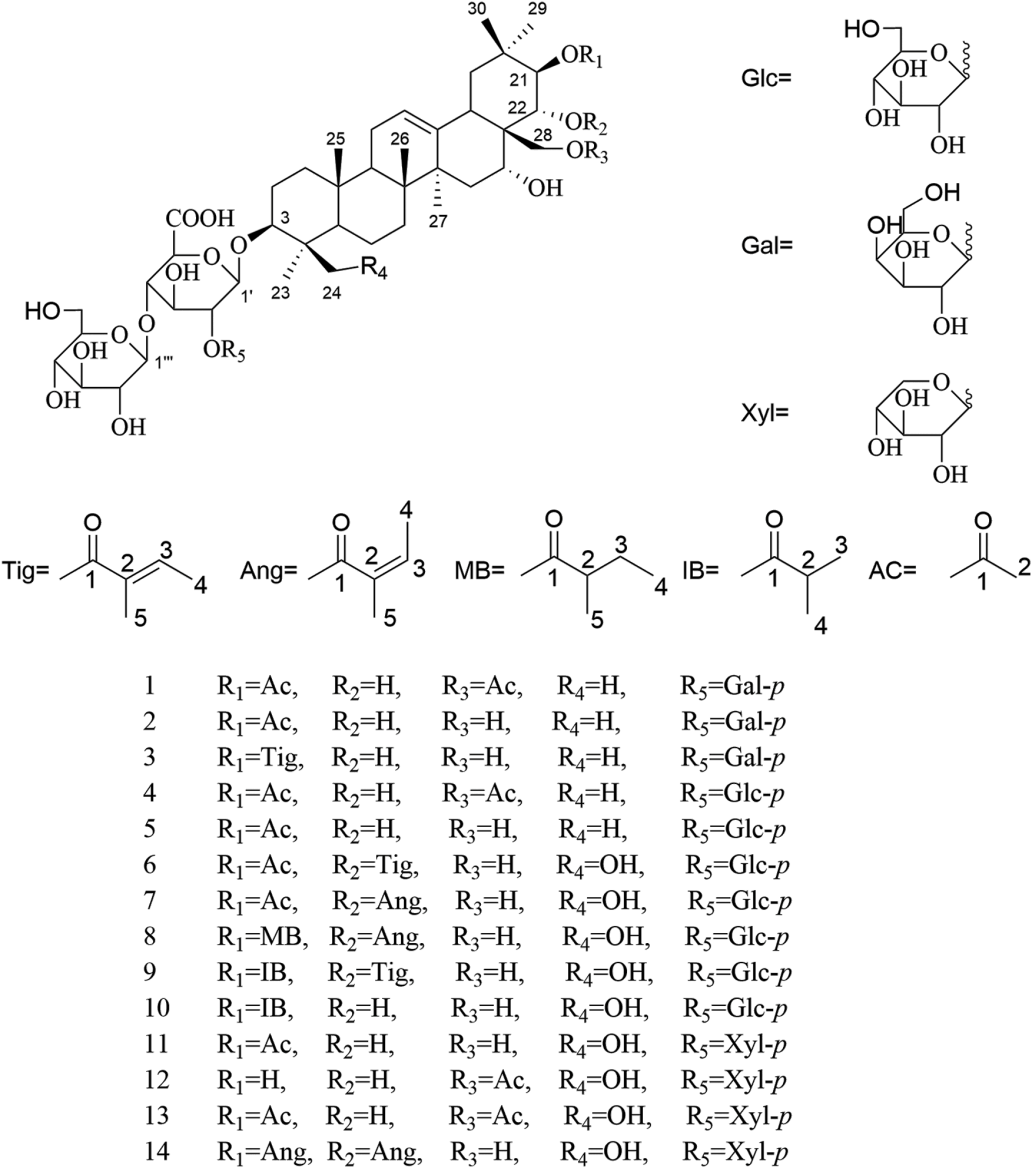
The structures of compounds **1–14**.

Aesculiside C (**1**) was isolated as a white amorphous powder which exhibited an ion peak at *m/z* 1073.5149 [M–H]^−^ (calcd. 1073.5169). Its molecular formula was confirmed as C_52_H_82_O_23_ based on HR-ESI-MS as well as ^13^C NMR spectroscopic data. The IR absorptions at 3,414 and 1,732 cm^−1^ implied the existence of the hydroxyl and carboxyl groups, respectively. The NMR data of **1** exhibited characteristic signals of a triterpenoid saponin.

**Figure 2:**
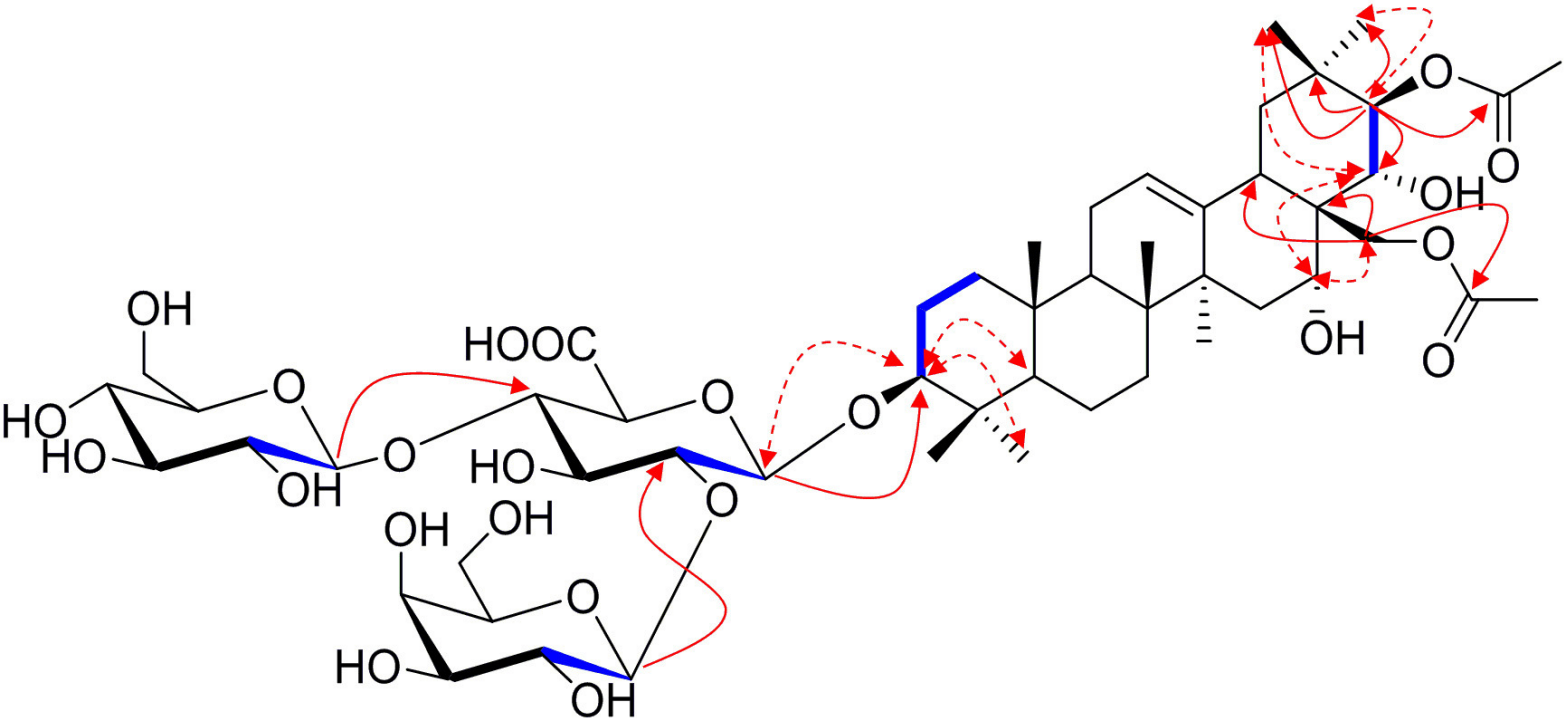
The selected HMBC (H → C), ^1^H-^1^H COSY (H **—** H) and NOESY (H ↔ H) correlations of compound **1**.

^1^H NMR of the aglycone portion indicated the presence of seven methyl protons at δ 0.83, 0.98, 1.07, 1.09, 1.26, 1.84 (each 3H except 1.26 for 6H, s), one olefinic proton at δ 5.47 (1H, br s), and a pair of geminal protons at δ 4.21 and 4.31 (1H each, d, *J* = 10.3 Hz), indicative of an olean-12-ene skeleton (Zhang S. L. et al., 2018). Four oxymethine proton signals assignable to H-3, H-16, H-21, and H-22 of the aglycone moiety were, respectively, observed at δ 3.26 (1H, dd, *J* = 11.6, 4.3 Hz), 4.76 (1H, m), 6.43 (1H, d, *J* = 9.8 Hz), and 4.45 (1H, d, *J* = 9.8 Hz), which further suggested the aglycon characteristic for 3, 16, 21, 22, 28-pentahydroxyolean-12-ene. As for the relative configurations of C-3, C-16, C-21, C-22, and C-28, the NOESY correlations between H-3/H-5/H_3_-23, H-21 /H_3_-29 suggested the H-3 and H-21, while the correlations between H-16 and H_2_-28, H_2_-28 and H-22, H-22, and H_3_-30 H_2_-28 and H-22, H-22, and H_3_-30 suggested β-orientations of H-16 and H-22 (Figure 2). On the basis of NOESY correlations and the vicinal coupling constants of the H-21 3β, 16α, 21β, 22α, 22α, 28-pentahydroxyolean-12-ene.

The 1D NMR spectra of **1** also showed two acetyl signals (δ_H_ 2.01, 2.11 and δ_C_ 170.6, 171.2, 20.6, 21.2). The cross peak between H-21 (δ 6.43) and C-1^′′′′^ (δ 171.2) in the HMBC spectrum established one of the acetyl groups was attached to C-21. The other acetyl group was assigned at C-28 according to the HMBC correlation of H_2_-28 (δ 4.31)/C1^′′′′′^ (170.6), which was further confirmed by the downfield chemical shifts of H_2_-28 and C-28 compared with typical oleanane-type triterpenoid (Aki et al., 2004; Zhang and Li, 2007; Yuan et al., 2012) (Figure 2).

The presence of three anomeric protons at δ 4.98 (1H, d, *J* = 7.1 Hz), 5.20 (1H, d, *J* = 7.9 Hz), 5.22 (1H, d, *J* = 7.7 Hz) was correlated with carbons at δ 105.0, 104.5, and 106.6 in HSQC spectrum, respectively, indicating trisaccharide residues. Acid hydrolysis of **1** yielded d-galactose, d-glucose, and d-glucuronic acid, which was established with HPLC analysis by comparing with authentic sugar samples after derivatization. Their relative configuration was determined to be β according to the large coupling constants. The ^1^H NMR and ^13^C NMR signals of the trisaccharide group were fully assigned by 2D-NMR spectra and compared with reference data (Yuan et al., 2013). Meanwhile, the upfield shifts of C-3 (δ 89.1) as well as the HMBC correlation between H-1′ (δ 4.98) with C-3 demonstrated that the trisaccharide unit was attached to C-3. The sequence of the sugar chain was further confirmed by the long correlations of H-1^′′^ (δ 5.22) and C-2′ (δ 82.2), H-1^′′′^ (δ 5.20) and C-4′ (δ 81.9) (Figure 2). Based on these data, compound **1** was concluded to be 3-*O*-[β-d-galactopyranosyl-(1→ 2)]-β-d-glucopyranosyl-(1→ 4)-β-d-glucuronopyranosyl-21β, 28-diacetyl-3β, 16α, 21β, 22α, 28-pentahydroxyolean-12-ene, named aesculiside C.

**Figure 3:**
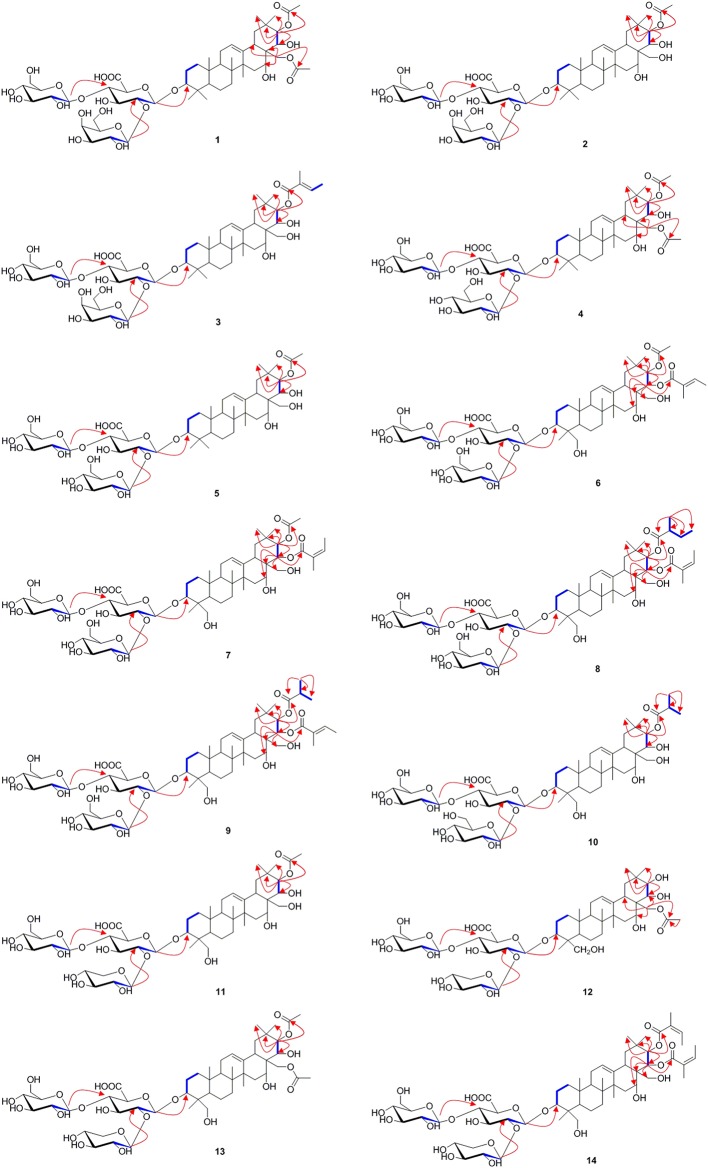
The selected HMBC (H → C) and ^1^H-^1^H COSY (H **—** H) correlations of compounds **1–14**.

Aesculiside D (**2**) with the molecular formula of C_50_H_80_O_22_ (*m/z* 1031.5067 [M–H]^−^; calcd. for C_50_H_79_O_22_, 1031.5063) was also obtained as a white, amorphous powder. Acid hydrolysis of **2** presented the same sugar moieties as compound **1**. The NMR data of **2** are similar to those of **1** except for the absence of an acetyl unit in **2**. The essential HMBC correlations of H-21 (δ 6.41)/C-1^′′′′^ (δ_C_ 171.4) indicated the acetyl unit was connected at C-21 (Figure 3). The remaining portion of **2** was superposable to **1** evidenced by careful analysis of their 2D NMR spectra. Thus, compound **2** was established as 3-*O*-[β-d-galactopyranosyl-(1→ 2)]-β-d-glucopyranosyl-(1→ 4)-β-d- glucuronopyranosyl-21β-acetyl-3β, 16α, 21β, 22α, 28-pentahydroxyolean-12-ene, namely aesculiside D.

Aesculiside E (**3**) was acquired as a white amorphous powder and its molecular formula was determined as C_53_H_84_O_22_ (*m/z* 1071.5376 [M–H]^−^; calcd. for C_53_H_83_O_22_, 1071.5376). Acid hydrolysis of **3** yielded d-galactose, d-glucose, and d-glucuronic acid. The NMR data of **3** showed a lot of resemblance with those of **2** except for the presence of a tigloyl moiety instead of an acetyl unit in **3**, which was supported by the characteristic olefinic quartet at δ 7.00 in its ^1^H-NMR spectrum. Moreover, the HSQC correlation signals of δ_H_ 1.61 with δ_C_ 14.0 and δ_H_ 1.87 with δ_C_ 12.3 confirmed the existence of a tigloyl group. The aforementioned data, together with the HMBC correlation from H-21 (δ 6.48) to C-1^′′′′^ (δ 168.5) confirmed the connection between the tigloyl group and C-21. Consequently, it was assigned as 3-*O*-[β-d-galactopyranosyl-(1→ 2)]-β-d-glucopyranosyl-(1→ 4)-β-d-glucuronopyranosyl-21β-tigloyl-3β, 16α, 21β, 22α, 28-pentahydroxyolean-12-ene.

Aesculiside F (**4**) and **1** gave the same molecular formula, deduced as C_52_H_82_O_23_ from its HR-ESI-MS and ^13^C NMR spectroscopic data. Comparison of the NMR data of **4** with those of **1** indicated that both saponins are closely related, differing at trisaccharide moiety where the galactose in **1** was replaced by a glucose in **4**, based on the distinction of their ^13^C NMR data (Table 3) (Yoshikawa et al., 1998), which was further verified by hydrolysis and derivatization as aforementioned. HMBC correlations revealed the position and sequences of the sugar moiety in **4** as described before. Hence, compound **4** was identified and named aesculiside F.

Aesculiside G (**5**), a white amorphous powder, was established to be an analog of **4** by HRESIMS and NMR spectrum interpretation. Careful analysis of their NMR data suggested that **4** possessed one more acetyl group compared to **5**. The key HMBC correlation from H-21 (δ 6.41) to C-1^′′′′^ (δ 171.3) suggested that the acetyl group was connected to C-21. The sugar residues in **5** was determined to be the same with those in **4** applying the method as before described. Accordingly, aesculiside G (**5**) was identified as 3-*O*-[β-d-glucopyranosyl-(1→ 2)]-β-d-glucopyranosyl-(1→ 4)-β-d-glucuronopyranosyl-21β-acetyl-3β, 16α, 21β, 22α, 28-pentahydroxyolean-12-ene.

Aesculiside H (**6**) and Aesculiside I (**7**) owned the same molecular formula of C_55_H_86_O_24_, according to their HR-ESI-MS data. The similar NMR spectra of **6** and **7** (Tables 1, 3) to those of **1**–**5** indicated that **6** and **7** are structural analogs of these compounds. The ^1^H NMR spectra of the aglycone portion of both compounds exhibited six tertiary methyl groups at δ 0.64 (Me-25), 0.78 (Me-26), 1.07 (Me-29), 1.30 (Me-30), 1.32 (Me-23), and 1.81 (Me-27). The absence of the characteristic singlet at δ_H_ 0.86 and δ_C_ 16.6 attributable to Me-24 in **5** and the additional resonances at δ_C_ 63.6 promoted that Me-24 could be oxygenated. This was corroborated by the key HMBC cross-peaks from H-24 (δ 4.25) to C-3 (δ 91.4), C-5 (δ 56.4). Thus, the structure of the aglycone of **6** and **7** was assigned as 3β, 16α, 21β, 22α, 24, 28-hexahydroxyolean-12-ene. Detailed NMR analysis disclosed that **6** and **7** possessed the same acyl group (acetyl) link to C-21 as that in **5**. The ^1^H and ^13^C NMR spectra of **6** displayed characteristic signals of a tigeloyl group (Tables 1, 3). The HMBC correlations from H-22 (δ 6.25) to C-1^′′′′′^ (δ 168.7) and from H-21 (δ 6.61) to C-1^′′′′^ (δ 171.2) provided definitive evidence that the acetyl was substituted at C-21, the tigeloyl group was substituted at C-22 in **6**. The connection of an angeloyl group to C-22 in **7** was validated by the HMBC correlations from H-22 (δ 6.30) to C-1^′′′′′^ (δ 168.5) and H-21 (δ 6.58) to C-1^′′′′^ (δ 171.1). The trisaccharide chain of **6** and **7** was the same as that of **5** as determined by the same method as mentioned before. Thus, the chemical structures of compounds **6** and **7** have been elucidated and named aesculiside H (**6**) and aesculiside I (**7**).

Aesculiside J (**8**) was assigned as C_58_H_92_O_24_ based on the [M–H]^−^ ion peak at *m/z* 1171.5908. Acid hydrolysis suggested that d-glucose and d-glucuronic acid existed in **8**. Comparison of the NMR spectroscopic data with those of compound **7** showed many similarities, except for the appearance of a 2-methylbutyryl moiety instead of the acetyl group in **7**, which was ascertained by the COSY correlations between H-2^′′′′^ and H-3^′′′′^, H-4^′′′′^ coupled with HMBC correlation of H-21 (δ 6.59)/C-1^′′′′^ (δ 176.6) and H-22 (δ 6.28)/C-1^′′′′′^ (δ 168.3). Thus, the structure of aesculiside J (**8**) was elucidated as 3-*O*-[β-d-glucopyranosyl-(1→ 2)]-β-d-glucopyranosyl-(1→ 4)-β-d-glucuronopyranosyl-21β-methylbutyryl-22α-angeloyl-3β, 16α, 21β, 22α, 24, 28-hexahydroxyolean-12-ene.

The elemental formula of aesculiside K (**9**) was confirmed as C_57_H_90_O_24_ by its HRESIMS data. The NMR data of **9** closely resembled those of **6** with the striking difference of the acetyl group signals at δ_C_ 171.2 and 21.3 in **6** replaced by an isobutyryl group signals at δ_C_ 177.0, 35.2, 19.8 and 19.5. COSY correlations of H-2^′′′′^/H-3^′′′′^, H-2^′′′′^/H-4^′′′′^ in conjunction with the HMBC cross-peaks of H-3^′′′′^ with C-1^′′′′^, C-2^′′′′^ and C-4^′′′′^ verified the existence of the isobutyryl group. HMBC correlations from H-21 (δ 6.61) to carbonyl carbon (δ 177.0) of the isobutyryl group, and from H-22 (δ 6.21) to carbonyl carbon (δ 168.7) of the tigeloyl group supported the attachment of the two acyl units to C21 and C22, respectively. Thus, the structure of aesculiside K (**9**) was fully elucidated as 3-*O*-[β-d-glucopyranosyl-(1→ 2)]-β-d-glucopyranosyl-(1→ 4)-β-d-glucuronopyranosyl-21β-isobutyryl-22α-tigeloyl-3β, 16α, 21β, 22α, 24, 28-hexahydroxyolean-12-ene.

Aesculiside L (**10**) had molecular formula of C_52_H_84_O_23_ established through its [M + H]^+^ ion peak at 1077.5471 and its NMR data. The sugar chain in **10** was same as **9**, using the same method as described before. Analysis of the ^1^H and ^13^C NMR spectroscopic data (Tables 2, 4) of **10** revealed a close structural resemblance to **9**, except for the absence of a tigeloyl group in **9**. The key HMBC correlations of H-21 to C-17, C-29, C-30, and C-1^′′′′^; and of H-22 to C-18, C-20 supported this deduction. Finally, the structure of **10** was proved and named aesculiside L.

According to the [M–H]^−^ ion peak at *m/z* 1017.4881 and its NMR data, the molecular formula of **11** was established to be C_49_H_78_O_22_, which is 30 mass units less than that of the known compound, aesculusosides C **(27)**. Detailed comparison of the NMR spectroscopic data between **11** and **27** revealed that they shared the same aglycone and C-21 substituent. d-xylose, d-glucose, and d-glucuronic acid were gained from acid hydrolysis of **11**. Further NMR analysis of the sugar portion suggested that the β-d-xylopyranose in **11** took the place of the β-d-glucose group substituent at C-2′ in **27**. Further confirmation was carried out by the significant cross peak: xyl-H-1 (δ_H_ 5.53) with glcA-C-2 (δ_C_78.7) in HMBC spectrum. Accordingly, aesculiside M (**11**) was unambiguously identified as 3-*O*-[β-d-xylopyranosyl-(1→ 2)]-β-d-glucopyranosyl-(1→4)-β-d-glucuronopyranosyl-21β-acetyl-3β, 16α, 21β, 22α, 24, 28-hexahydroxyolean-12-ene.

The molecular formula of aesculiside N (**12**) was calculated as C_49_H_78_O_22_ by virtue of its HR-ESI-MS spectrum. Its ^1^H NMR spectrum exhibited six singlet methyl protons [δ_H_ 0.75, 0.96, 1.35, 1.36, 1.39, 1.86] along with an olefinic proton at δ_H_ 5.46. The aforementioned spectroscopic data with its ^13^C-NMR data (Table 4) for the aglycone portion showed a close resemblance to those of protoaescigenin (Konoshima and Lee, 1986). The relative configuration of C-21 and C-22 was established to be 21β and 22α on the basis of the NOESY correlations of H-21/H_3_-29, H_2_-28/H-22/H-30 and the vicinal coupling constants of the H-21 and H-22 (*J* = 9.0 Hz). We also observed the presence of an acetyl moiety [δ_H_ 1.96 (3H, s); δ_C_ 20.6, 170.7] which was attached to C-28 due to the HMBC correlation from H-28 to the carbonyl carbon of the acetyl group. The resonances (1D and 2D NMR) of the sugar moieties and the results of hydrolysis of **12** revealed that **12** and **11** possessed the same trisaccharide chain at aglycone C-3. Thus, the structure of aesculiside N (**12**) was affirmed as 3-*O*-[β-d-xylopyranosyl-(1→ 2)]-β-d-glucopyranosyl-(1→ 4)-β-d-glucuronopyranosyl-28-acetyl-3β, 16α, 21β, 22α, 24, 28-hexahydroxyolean-12-ene.

Aesculiside O (**13**) possessed the molecular formula of C_51_H_80_O_23_ based on its HR-ESI-MS data. d-glucuronic acid, d-xylose, and d-glucose were afforded from **13** via the same procedure as before. The side-by-side analysis of the NMR spectroscopic resonances (Tables 2, 4) between **13** and **12** revealed that these two compounds owned similar structural features, with the only difference being due to an additional acetyl group connected with C-21 in **13**. HMBC correlations from H-21 (δ 6.37) to ester carbonyl (δ 171.1) of the acetyl unit confirm this proposal. Hence, the structure of **13** was elucidated as 3-*O*-[β-d-xylopyranosyl-(1→ 2)]-β-d-glucopyranosyl-(1→ 4)-β-d-glucuronopyranosyl-21β, 28-diacetyl-3β, 16α, 21β, 22α, 24, 28-hexahydroxyolean-12-ene.

**Figure 4:**
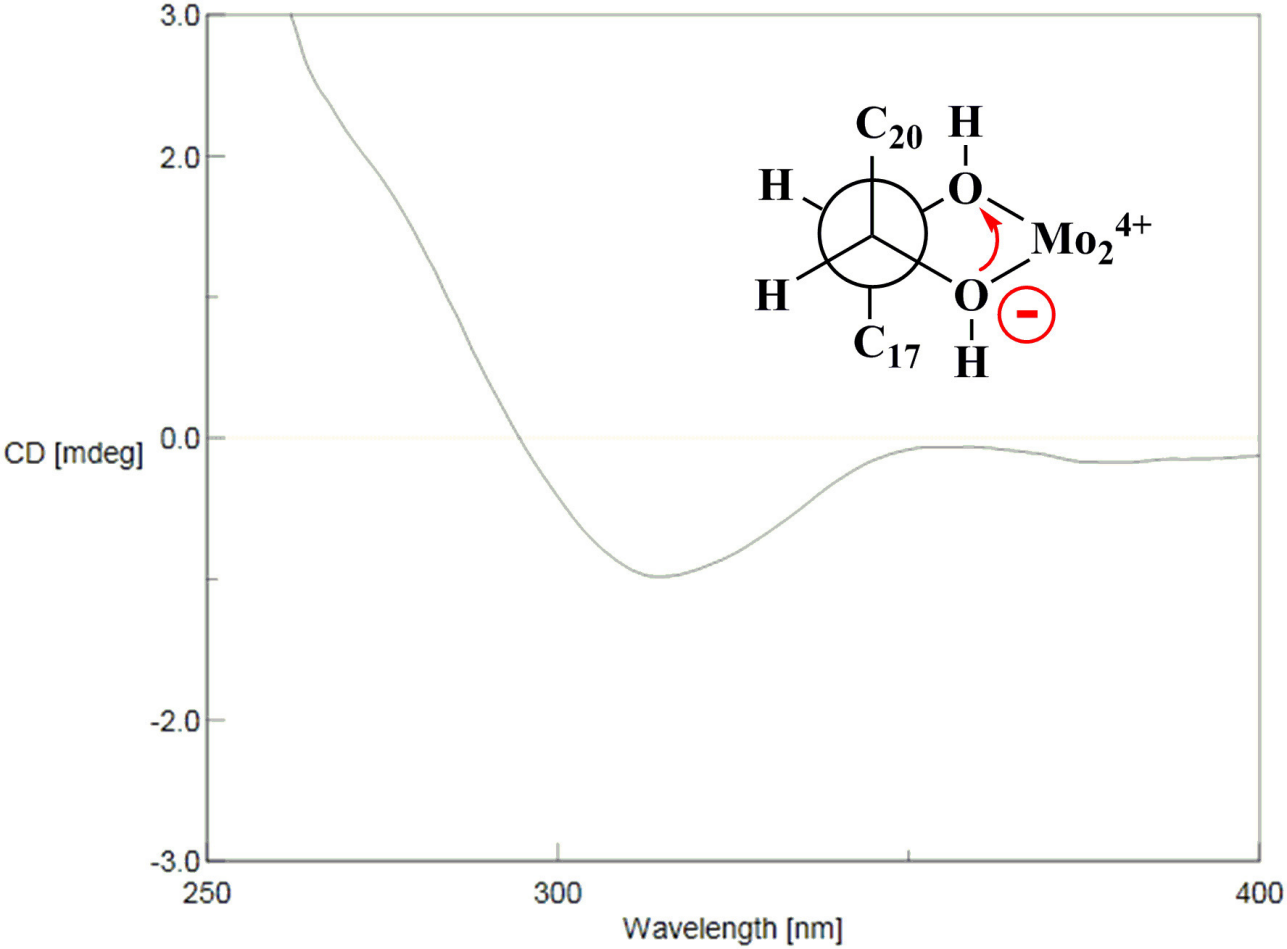
Mo_2_(OAc)_4_-induced CD (ICD) spectra of **14a**.

**Figure 5:**
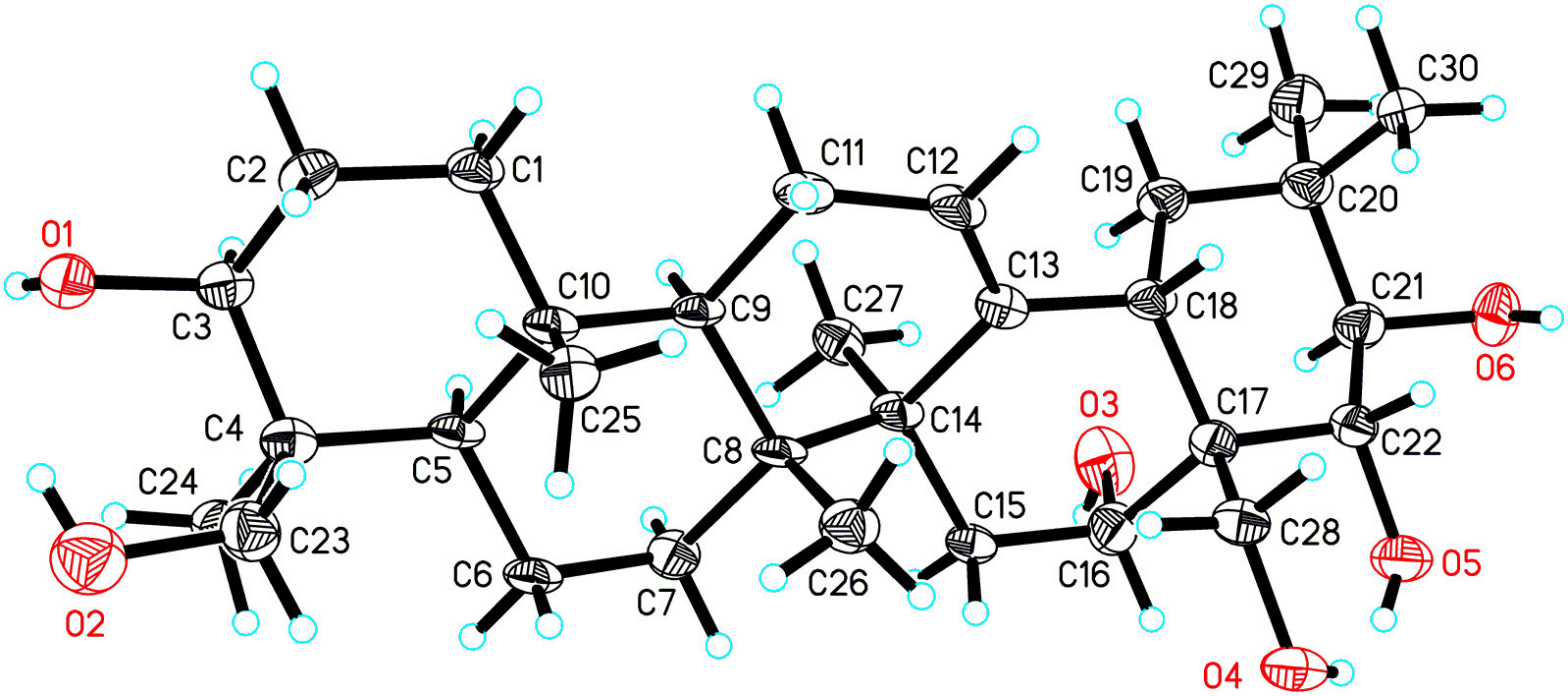
ORTEP drawing of **14a**.

The HR-ESI-MS of aesculiside P (**14**) yielded a [M + H] ^+^ ion with *m/z* 1141.5785, consistent with a molecular formula of C_57_H_88_O_23_ (calcd. for C_57_H_89_O_23_, 1141.5795). Analysis of its NMR data (Tables 2, 4) implied the identical trisaccharide chain to **13**, which is also established by acid hydrolysis results. The ^1^H NMR spectrum of its aglycone showed six tertiary methyls: δ_H_ 0.71 (Me-25), 0.80 (Me-26), 1.08 (Me-29), 1.32 (Me-30), 1.35 (Me-23), and 1.84 (Me-27); one olefinic proton: δ_H_ 5.39 (br s) and a pair of oxygenated methine protons: δ_H_ 6.69 and 6.32 (each 1H, d, *J* = 10.2 Hz). Meanwhile, the 1D-NMR spectra of **14** exhibited typical resonances of two angeloyl groups (Tables 2, 4) and the observed HMBC correlations of H-21/ C-1^′′′′^ and H-22/ C-1^′′′′′^ provided definitive evidence of their position. The relative configuration of **14** was established via NOESY experiment. The correlations between H-3/H-5/H_3_-23, H-21/H_3_-29 suggested the α-orientations of H-3 and H-21, the correlations between H-16/H_2_-28/H-22/H_3_-30 reminded β-orientations of H-16 and H-22. To further confirm its absolute configuration, we made many attempts. Owing to the amount of **14**, its aglycone (**14a**) was easily obtained by hydrolyzation and the absolute configuration of C-21 and C-22 in **14a** was determined by Mo_2_(AcO)_4_-induced CD. As shown in Figure 4, the ICD exhibited a negative cotton effect at 313 nm, suggesting the R configuration of C-21, according to the Snatzke rule (Snatzke et al., 1981; Di Bari et al., 2001). Fortunately, a single crystal of **14a** was obtained and suitable for X-ray crystallographic analysis (Figure 5). The Flack parameter of 0.27 (14) allowed an unambiguous assignment of the absolute configuratihon of **14a**. Based on these data, compound **14** was undoubtedly identified as 3-*O*-[β-d-xylopyranosyl-(1→ 2)]-β-d-glucopyranosyl-(1→ 4)-β-d-glucuronopyranosyl-21*R*, 22*R*-diangeloyl-3*S*, 16*R*, 21*R*, 22*R*, 24, 28-hexahydroxyolean-12-ene.

The absolute configurations of the aglycones of **1**–**13** were all deduced to be 3*S*, 16*R*, 21*R*, 22*R* based on the absolute configuration of **14** and their mutual biogenetic source.

Additionally, the 19 known compounds were identified (Figure S100) as 3-*O*-[β-d-galactopyranosyl-(1→ 2)]-β-d-glucopyranosyl-(1→ 4)-β-d-glucuronopyranosyl-21β, 22α-ditigeloyl-3β, 16α, 21β, 22α, 22α, 28-pentahydroxyolean-12-ene (**15**) (Kameyama and Fujimura, 2009), 3-*O*-[β-d-glucopyranosyl-(1→ 2)]-β-d-glucopyranosyl-(1→ 4)-β-d-glucuronopyranosyl-21β, 22α-diangeloyl-3β, 16α, 21β, 22α, 24, 28-hexahydroxyolean-12-ene (**16**), escin Ia (**17**), escin Ib (**18**), isoescin Ia (**18**), isoescin Ia (**19**), isoescin Ib (**20**) (Zhang et al., 1999), isoescin IIb (**21**) (Yang et al., 1999b), escin IIIa (**22**) (Yoshikawa et al., 1996), escin IV (**23**), escin V (**24**) (Yoshikawa et al., 1998), aesculusosides A-C (**25-27**) (Cheng et al., 2018), aesculioside A-B (**28, 29**) (Zhang et al., 1999), aesculiside A (**30**) (Cheng et al., 2018), desacylescin I (**31**) (Cheng et al., 2016), desacylescin II (**32**) (Yoshikawa et al., 1996), deacetylescinIIb (**33**) (Kimura et al., 2006) by comparisons of their spectroscopic data with reported values.

The cytotoxic activities against three human cancer cell lines (Hep G2, HCT-116, and MGC-803) of compounds **1**–**33** were evaluated using the MTT method, with 5-fluorouracil (5-FU) as positive control (Table 5). Among them, compounds **8**, **9**, **14**–**16**, **18**, **22** showed potent cytotoxicity against all the tested human cancer cell lines with IC_50_ ranging between 2 and 21 μM. Compounds **3**, **6**, **7**, **17**–**19**, **20**, **24**, **28** were less active (IC_50_: 13 to >40 μM) whereas the other isolates displayed no toxicity in all cell lines at 50 μM. These results suggested that the compounds with acylations at both C-21 and C-22 exhibited stronger inhibitory activities than those with acylations at C-21 and C-28 or only at C-21. In addition, it seems that the presence of the tigloyl, angeloyl, methylbutyryl, and isobutyryl groups affects the inhibitory activity of these compounds on the tested cell lines positively.

**Table 5:** Cytotoxic activity of compounds **1–33** by the MTT method.

**Compd**.	**IC**_**50**_ **(*****μ*****M)**^**a**^		
	**Hep G2**	**HCT-116**	**MGC-803**
**3**	>40	36 ± 2	>40
**6**	30 ± 0	17 ± 1	31 ± 1
**7**	23. ± 0	13 ± 1	29 ± 0
**8**	13 ± 0	3 ± 1	9 ± 0
**9**	21 ± 1	6 ± 1	9 ± 0
**14**	11 ± 0	8 ± 1	2 ± 0
**15**	11 ± 1	18 ± 1	6 ± 0
**16**	12 ± 0	8 ± 0	3 ± 0
**17**	25 ± 2	13 ± 1	31 ± 2
**18**	13 ± 0	16 ± 3	16 ± 1
**19**	>40	26 ± 1	30 ± 1
**20**	>40	32 ± 2	25 ± 1
**22**	10 ± 1	10 ± 1	18 ± 3
**24**	>40	22 ± 3	32 ± 1
**28**	>40	28 ± 1	>40
**5-FU**^**b**^	11 ± 1	5 ± 0	13 ± 1

a Results are expressed as means ± SD (n = 3). Compounds **1–2, 4, 5, 10–13, 21, 23, 25–27, 29–33** were inactive against all cell lines tested (IC_50_ > 40 μM).

b *Positive control*.

**Figure 6:**
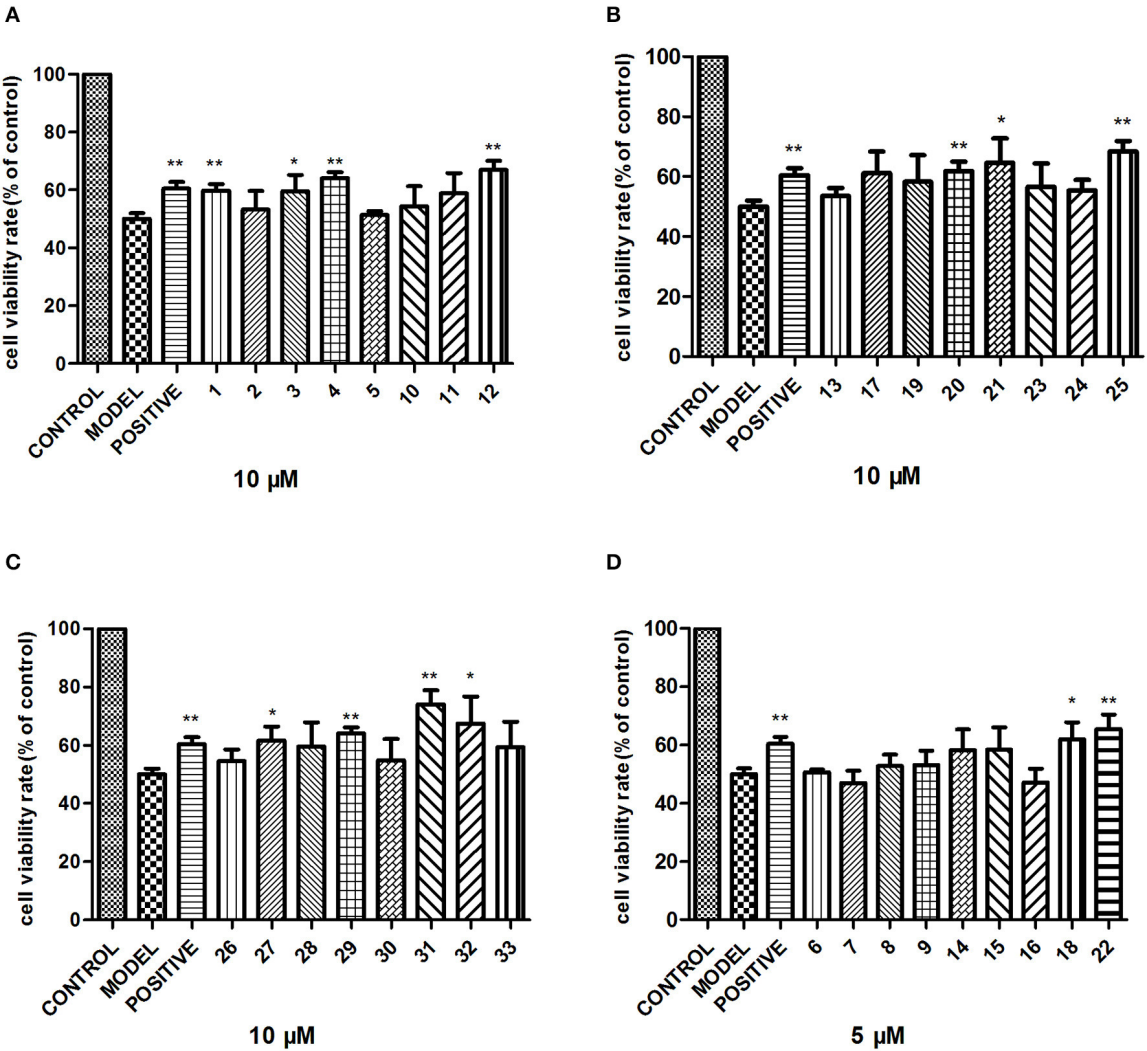
Neuroprotective activity of compounds **1–33**. PC12 cells were exposed to 1 mM CoCl_2_ for 24 h with or without the indicated concentrations of compounds **1–33**, and the cell viability was recorded: **(A)** compounds **1–5**, **10–12**; **(B)** compounds **13**, **17**, **19–21**, **23–25**; **(C)** compounds **26–33**; **(D)** compounds **6–9, 14–16**, **18**, **22**. The data are expressed as means ± SEM. Three independent experiments were performed. *P < 0.05, **P < 0.01.

To examine the neuroprotective effect, the cytotoxic activity of compounds **1**–**33** against PC12 cell line was first evaluated. Among them, compounds **6**–**9**, **14**–**16**, **18**, **22** showed no obvious cytotoxic effects on PC12 cells at a dose of 5 μM, while others at 10 μM. Next, 5 μM compounds **6**–**9**, **14**–**16**, **18**, **22** and 10 μM others were tested for their neuroprotective properties against COCl_2_-induced toxicity in PC12 cells with trolox as the positive control. Among these, compounds **1**, **4**, **12**, **20**, **22**, **25**, **29**, **31** exhibited moderate activities against C_O_Cl_2_-induced PC12 cell injury (Figure 6).

## Conclusion

Plants of the genus *Aesculus* have been proved to be rich in polyhydroxyoleanene triterpenoid saponins which have been characterized more than 100. When compared to the relatively extensive research on other species of *Aesculus* genus, little is known regarding the chemical constituents and the biological activity of the *Aesculus chinensis* Bge. var. *chekiangensis* (Hu et Fang) Fang species. The present paper reports 14 new polyhydroxy oleanene saponins (**1**–**14**) along with 19 known analogs from the seeds of *A. chinensis* Bge. var. *chekiangensis*. Structure elucidation was achieved *via* various techniques, and the absolute configuration of the aglycones was undoubtedly defined through X-ray diffraction analysis as well as Mo_2_(OAc)_4_-induced ECD method for the first time. Further cytotoxicity evaluation against three human tumor cell lines suggested that compounds **8**, **9**, **14–16**, **18**, **22** displayed strong inhibitory activities against all three cell lines; compounds **3**, **6**, **7**, **17–19**, **20**, **24**, **28** exhibited weak activities while the remaining isolates showed no toxicity at 50 μM. These results suggested that isolates with two acylations at C-21 and C-22 might be important for the cytotoxicity, especially substituted by tigloyl, angeloyl, methylbutyryl, and isobutyryl groups. In addition, the first test about the neuroprotective properties of triterpenoid saponins from *Aesculus* genus found that compounds **1**, **4**, **12**, **20**, **22**, **25**, **29**, **31** exhibited moderate activities against CoCl_2_-induced PC12 cell injury.

## Data Availability Statement

The crystallographic dataset generated for this study can be found in the Cambridge Crystallographic Data Centre under the CCDC number 1957449. All other datasets generated for this study are included in the article/Supplementary Material.

## Author Contributions

NZ and SW was responsible for the isolation and elucidated of compounds. NZ tested cytotoxicity, neuroprotective effects of the compounds, interpreted the data, and wrote the paper. SC, QZ, and NK revised the manuscript. LD and FQ were the project leaders organizing and guiding the experiment. All authors read and approved the final manuscript.

### Conflict of Interest

The authors declare that the research was conducted in the absence of any commercial or financial relationships that could be construed as a potential conflict of interest.
