# Supplementary material for: Bioactive Triterpenoid Saponins From the Seeds of Aesculus chinensis Bge. var. chekiangensis
Source: Front Chem. 2020 Jan 23;7:908. doi: 10.3389/fchem.2019.00908 (PMC6989559; doi:10.3389/fchem.2019.00908)
Supplement: Supplementary file 2 [file Data_Sheet_2.PDF]

Supplementary Material

List of Supplementary Figures

S1. ^{1}H NMR Spectrum (600 MHZ, Pyridine-d_5) of the new compound **1**
S2. ^{13}C NMR Spectrum (150 MHZ, Pyridine-d_5) of the new compound **1**
S3. HSQC Spectrum of the new compound **1**
S4. ^{1}H-^{1}H COSY Spectrum of the new compound **1**
S5. HMBC Spectrum of the new compound **1**
S6. NOESY Spectrum of the new compound **1**
S7. HRESIMS spectrum of the new compound **1**
S8. ^{1}H NMR Spectrum (600 MHZ, Pyridine-d_5) of the new compound **2**
S9. ^{13}C NMR Spectrum (150 MHZ, Pyridine-d_5) of the new compound **2**
S10. HSQC Spectrum of the new compound **2**
S11. ^{1}H-^{1}H COSY Spectrum of the new compound **2**
S12. HMBC Spectrum of the new compound **2**
S13. NOESY Spectrum of the new compound **2**
S14. HRESIMS spectrum of the new compound **2**
S15. ^{1}H NMR Spectrum (600 MHZ, Pyridine-d_5) of the new compound **3**
S16. ^{13}C NMR Spectrum (150 MHZ, Pyridine-d_5) of the new compound **3**
S17. HSQC Spectrum of the new compound **3**
S18. ^{1}H-^{1}H COSY Spectrum of the new compound **3**
S19. HMBC Spectrum of the new compound **3**
S20. NOESY Spectrum of the new compound **3**
S21. HRESIMS spectrum of the new compound **3**
S22. ^{1}H NMR Spectrum (600 MHZ, Pyridine-d_5) of the new compound **4**
S23. ^{13}C NMR Spectrum (150 MHZ, Pyridine-d_5) of the new compound **4**
S24. HSQC Spectrum of the new compound **4**
S25. ^{1}H-^{1}H COSY Spectrum of the new compound **4**
S26. HMBC Spectrum of the new compound **4**
S27. NOESY Spectrum of the new compound **4**
S28. HRESIMS spectrum of the new compound **4**
S29. ^{1}H NMR Spectrum (600 MHZ, Pyridine-d_5) of the new compound **5**
S30. ^{13}C NMR Spectrum (150 MHZ, Pyridine-d_5) of the new compound **5**
S31. HSQC Spectrum of the new compound **5**
S32. ^{1}H-^{1}H COSY Spectrum of the new compound **5**
S33. HMBC Spectrum of the new compound **5**
S34. NOESY Spectrum of the new compound **5**
S35. HRESIMS spectrum of the new compound **5**
S36. ^{1}H NMR Spectrum (600 MHZ, Pyridine-d_5) of the new compound **6**
S37. ^{13}C NMR Spectrum (150 MHZ, Pyridine-d_5) of the new compound **6**
S38. HSQC Spectrum of the new compound **6**
S39. ^{1}H-^{1}H COSY Spectrum of the new compound **6**
S40. HMBC Spectrum of the new compound **6**
S41. NOESY Spectrum of the new compound **6**

S42. HRESIMS spectrum of the new compound **6**
S43. ^{1}H NMR Spectrum (600 MHZ, Pyridine-d_5) of the new compound **7**
S44. ^{13}C NMR Spectrum (150 MHZ, Pyridine-d_5) of the new compound **7**
S45. HSQC Spectrum of the new compound **7**
S46. ^{1}H-^{1}H COSY Spectrum of the new compound **7**
S47. HMBC Spectrum of the new compound **7**
S48. NOESY Spectrum of the new compound **7**
S49. HRESIMS spectrum of the new compound **7**
S50. ^{1}H NMR Spectrum (600 MHZ, Pyridine-d_5) of the new compound **8**
S51. ^{13}C NMR Spectrum (150 MHZ, Pyridine-d_5) of the new compound **8**
S52. HSQC Spectrum of the new compound **8**
S53. ^{1}H-^{1}H COSY Spectrum of the new compound **8**
S54. HMBC Spectrum of the new compound **8**
S55. NOESY Spectrum of the new compound **8**
S56. HRESIMS spectrum of the new compound **8**
S57. ^{1}H NMR Spectrum (600 MHZ, Pyridine-d_5) of the new compound **9**
S58. ^{13}C NMR Spectrum (150 MHZ, Pyridine-d_5) of the new compound **9**
S59. HSQC Spectrum of the new compound **9**
S60. ^{1}H-^{1}H COSY Spectrum of the new compound **9**
S61. HMBC Spectrum of the new compound **9**
S62. NOESY Spectrum of the new compound **9**
S63. HRESIMS spectrum of the new compound **9**
S64. ^{1}H NMR Spectrum (600 MHZ, Pyridine-d_5) of the new compound **10**
S65. ^{13}C NMR Spectrum (150 MHZ, Pyridine-d_5) of the new compound **10**
S66. HSQC Spectrum of the new compound **10**
S67. ^{1}H-^{1}H COSY Spectrum of the new compound **10**
S68. HMBC Spectrum of the new compound **10**
S69. NOESY Spectrum of the new compound **10**
S70. HRESIMS spectrum of the new compound **10**
S71. ^{1}H NMR Spectrum (600 MHZ, Pyridine-d_5) of the new compound **11**
S72. ^{13}C NMR Spectrum (150 MHZ, Pyridine-d_5) of the new compound **11**
S73. HSQC Spectrum of the new compound **11**
S74. ^{1}H-^{1}H COSY Spectrum of the new compound **11**
S75. HMBC Spectrum of the new compound **11**
S76. NOESY Spectrum of the new compound **11**
S77. HRESIMS spectrum of the new compound **11**
S78. ^{1}H NMR Spectrum (600 MHZ, Pyridine-d_5) of the new compound **12**
S79. ^{13}C NMR Spectrum (150 MHZ, Pyridine-d_5) of the new compound **12**
S80. HSQC Spectrum of the new compound **12**
S81. ^{1}H-^{1}H COSY Spectrum of the new compound **12**
S82. HMBC Spectrum of the new compound **12**
S83. NOESY Spectrum of the new compound **12**
S84. HRESIMS spectrum of the new compound **12**
S85. ^{1}H NMR Spectrum (600 MHZ, Pyridine-d_5) of the new compound **13**
S86. ^{13}C NMR Spectrum (150 MHZ, Pyridine-d_5) of the new compound **13**
S87. HSQC Spectrum of the new compound **13**
S88. ^{1}H-^{1}H COSY Spectrum of the new compound **13**

S89. HMBC Spectrum of the new compound **13**
S90. NOESY Spectrum of the new compound **13**
S91. HRESIMS spectrum of the new compound **13**
S92. ^{1}H NMR Spectrum (600 MHZ, Pyridine-d_5) of the new compound **14**
S93. ^{13}C NMR Spectrum (150 MHZ, Pyridine-d_5) of the new compound **14**
S94. HSQC Spectrum of the new compound **14**
S95. ^{1}H-^{1}H COSY Spectrum of the new compound **14**
S96. HMBC Spectrum of the new compound **14**
S97. NOESY Spectrum of the new compound **14**
S98. HRESIMS spectrum of the new compound **14**
S99. The structure of compound **14a**
S100. The structures of compounds **15-33**
S101. ^{1}H NMR Spectrum (600 MHZ, Pyridine-d_5) of the new compound **15**
S102. ^{13}C NMR Spectrum (150 MHZ, Pyridine-d_5) of the new compound **15**
S103. HRESIMS spectrum of the new compound **15**
S104. ^{1}H NMR Spectrum (600 MHZ, Pyridine-d_5) of the new compound **16**
S105. ^{13}C NMR Spectrum (150 MHZ, Pyridine-d_5) of the new compound **16**
S106. HRESIMS spectrum of the new compound **16**
S107. ^{1}H NMR Spectrum (600 MHZ, Pyridine-d_5) of the new compound **17**
S108. ^{13}C NMR Spectrum (150 MHZ, Pyridine-d_5) of the new compound **17**
S109. HRESIMS spectrum of the new compound **17**
S110. ^{1}H NMR Spectrum (600 MHZ, Pyridine-d_5) of the new compound **18**
S111. ^{13}C NMR Spectrum (150 MHZ, Pyridine-d_5) of the new compound **18**
S112. HRESIMS spectrum of the new compound **18**
S113. ^{1}H NMR Spectrum (600 MHZ, Pyridine-d_5) of the new compound **19**
S114. ^{13}C NMR Spectrum (150 MHZ, Pyridine-d_5) of the new compound **19**
S115. HRESIMS spectrum of the new compound **19**
S116. ^{1}H NMR Spectrum (600 MHZ, Pyridine-d_5) of the new compound **20**
S117. ^{13}C NMR Spectrum (150 MHZ, Pyridine-d_5) of the new compound **20**
S118. HRESIMS spectrum of the new compound **20**
S119. ^{1}H NMR Spectrum (600 MHZ, Pyridine-d_5) of the new compound **21**
S120. ^{13}C NMR Spectrum (150 MHZ, Pyridine-d_5) of the new compound **21**
S121. HRESIMS spectrum of the new compound **21**
S122. ^{1}H NMR Spectrum (600 MHZ, Pyridine-d_5) of the new compound **22**
S123. ^{13}C NMR Spectrum (150 MHZ, Pyridine-d_5) of the new compound **22**
S124. HRESIMS spectrum of the new compound **22**
S125. ^{1}H NMR Spectrum (600 MHZ, Pyridine-d_5) of the new compound **23**
S126. ^{13}C NMR Spectrum (150 MHZ, Pyridine-d_5) of the new compound **23**
S127. HRESIMS spectrum of the new compound **23**
S128. ^{1}H NMR Spectrum (600 MHZ, Pyridine-d_5) of the new compound **24**
S129. ^{13}C NMR Spectrum (150 MHZ, Pyridine-d_5) of the new compound **24**
S130. HRESIMS spectrum of the new compound **24**
S131. ^{1}H NMR Spectrum (600 MHZ, Pyridine-d_5) of the new compound **25**
S132. ^{13}C NMR Spectrum (150 MHZ, Pyridine-d_5) of the new compound **25**
S133. HRESIMS spectrum of the new compound **25**
S134. ^{1}H NMR Spectrum (600 MHZ, Pyridine-d_5) of the new compound **26**
S135. ^{13}C NMR Spectrum (150 MHZ, Pyridine-d_5) of the new compound **26**
S136. HRESIMS spectrum of the new compound **26**

S137. ^{1}H NMR Spectrum (600 MHZ, Pyridine-d_5) of the new compound **27**
S138. ^{13}C NMR Spectrum (150 MHZ, Pyridine-d_5) of the new compound **27**
S139. HRESIMS spectrum of the new compound **27**
S140. ^{1}H NMR Spectrum (600 MHZ, Pyridine-d_5) of the new compound **28**
S141. ^{13}C NMR Spectrum (150 MHZ, Pyridine-d_5) of the new compound **28**
S142. HRESIMS spectrum of the new compound **28**
S143. ^{1}H NMR Spectrum (600 MHZ, Pyridine-d_5) of the new compound **29**
S144. ^{13}C NMR Spectrum (150 MHZ, Pyridine-d_5) of the new compound **29**
S145. HRESIMS spectrum of the new compound **29**
S146. ^{1}H NMR Spectrum (600 MHZ, Pyridine-d_5) of the new compound **30**
S147. ^{13}C NMR Spectrum (150 MHZ, Pyridine-d_5) of the new compound **30**
S148. HRESIMS spectrum of the new compound **30**
S149. ^{1}H NMR Spectrum (600 MHZ, Methanol-d_4) of the new compound **31**
S150. ^{13}C NMR Spectrum (150 MHZ, Methanol-d_4) of the new compound **31**
S151. HRESIMS spectrum of the new compound **31**
S152. ^{1}H NMR Spectrum (600 MHZ, Methanol-d_4) of the new compound **32**
S153. ^{13}C NMR Spectrum (150 MHZ, Methanol-d_4) of the new compound **32**
S154. HRESIMS spectrum of the new compound **32**
S155. ^{1}H NMR Spectrum (600 MHZ, Pyridine-d_5) of the new compound **33**
S156. ^{13}C NMR Spectrum (150 MHZ, Pyridine-d_5) of the new compound **33**
S157. HRESIMS spectrum of the new compound **33**

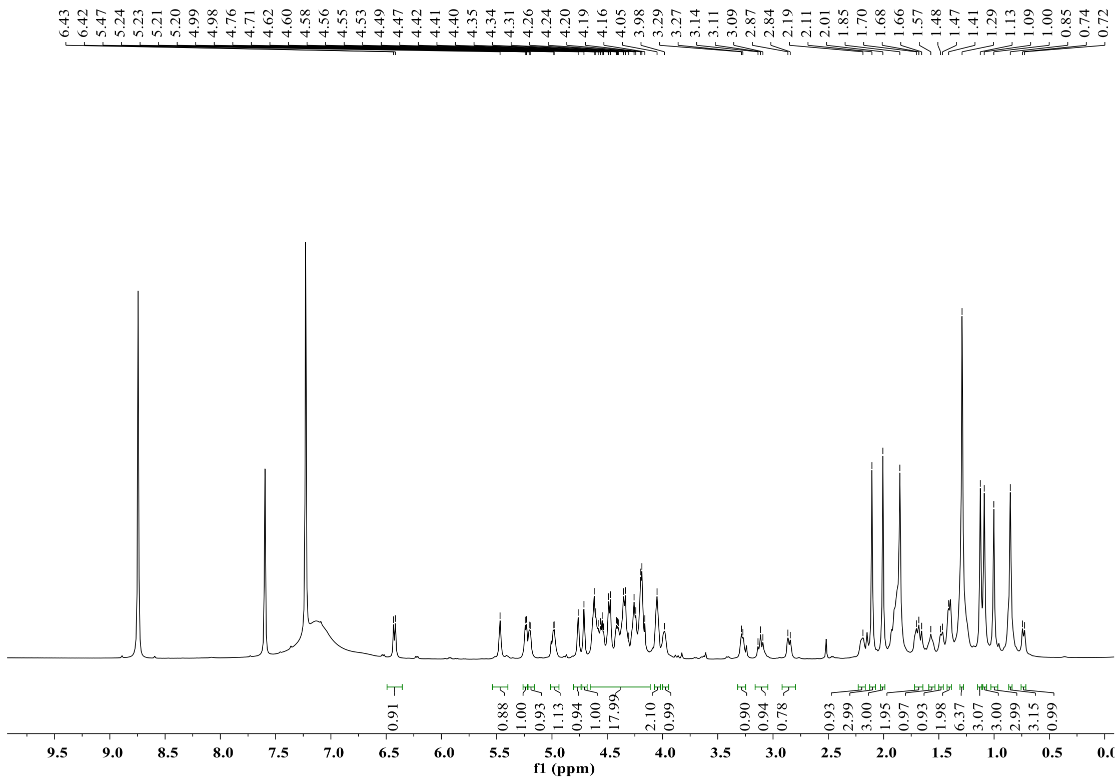

S1. ^{1}H NMR Spectrum (600 MHZ, Pyridine-d_5) of the new compound **1**

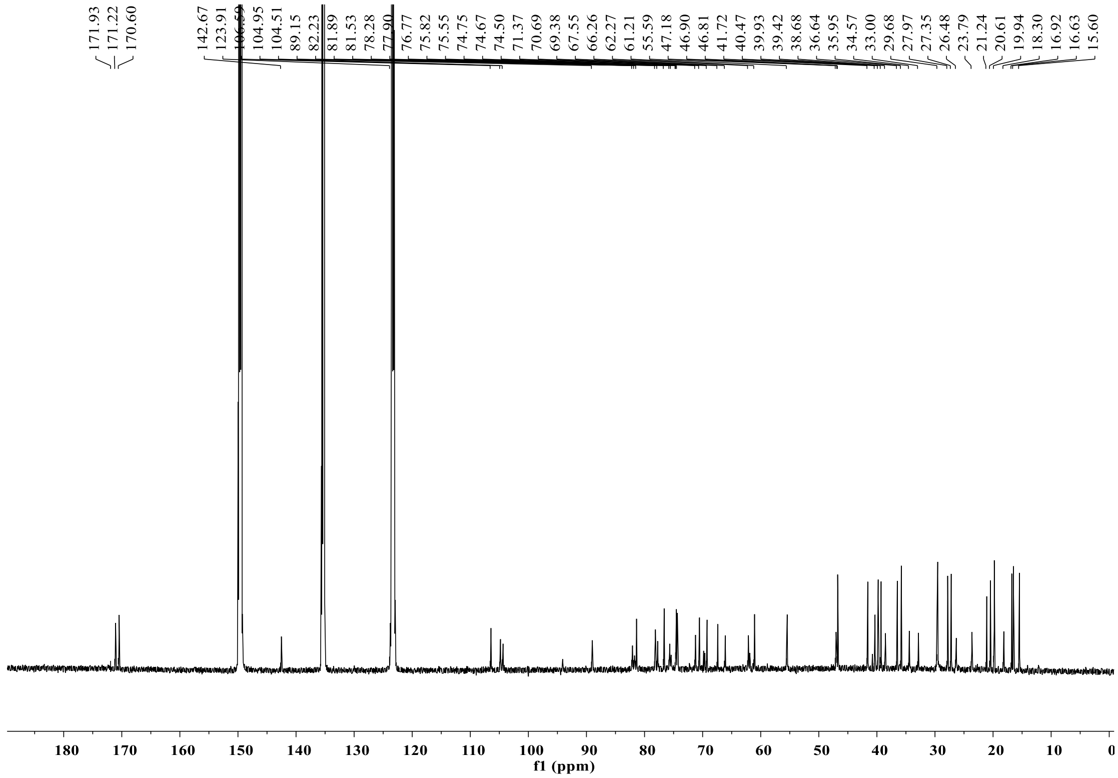

S2. ^{13}C NMR Spectrum (150 MHZ, Pyridine-d_5) of the new compound **1**

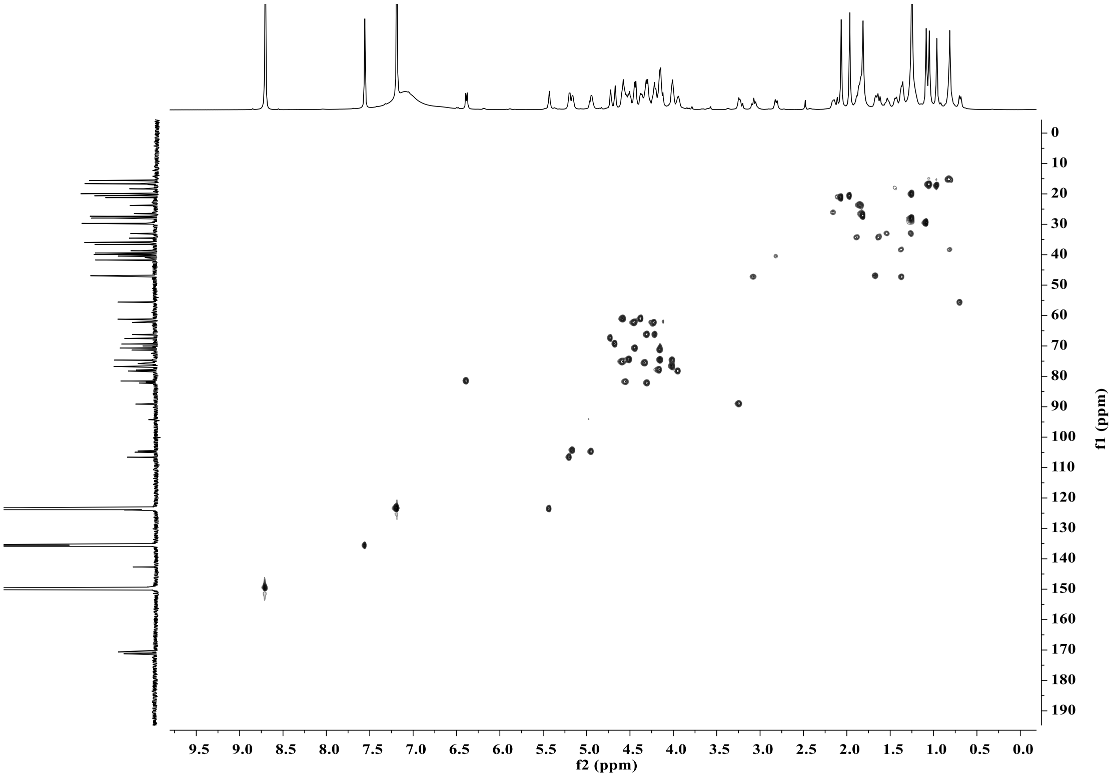

S3. HSQC Spectrum of the new compound **1**

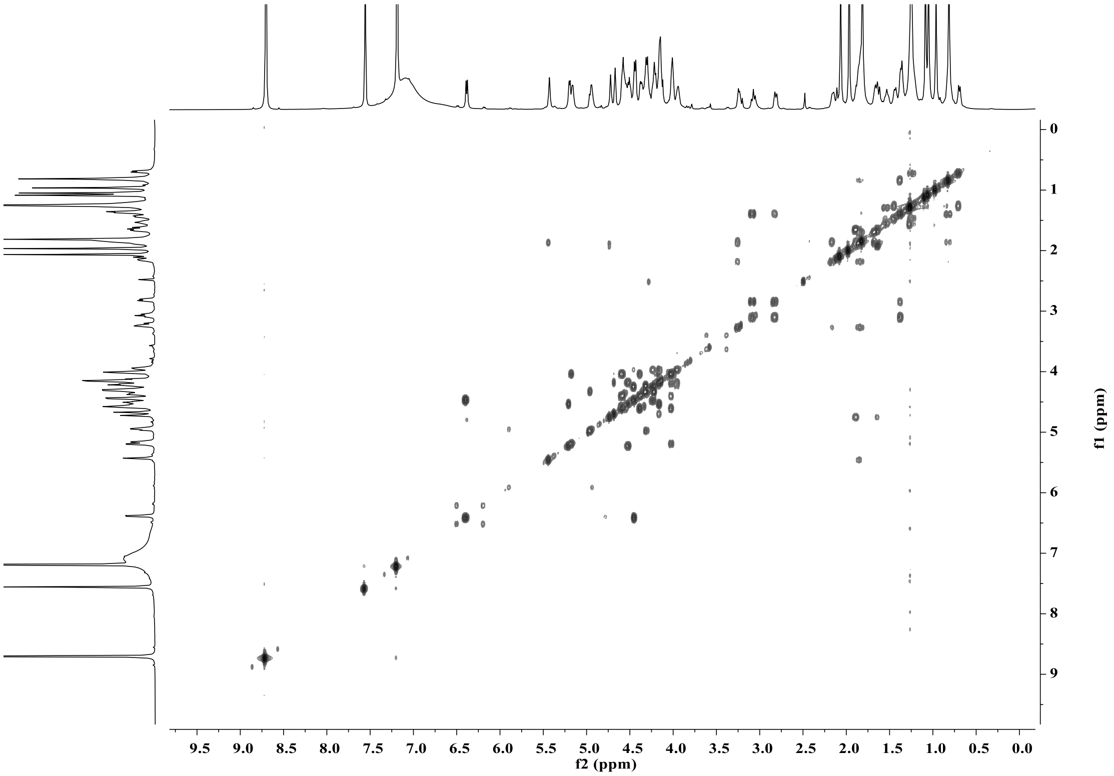

S4. ^{1}H-^{1}H COSY Spectrum of the new compound **1**

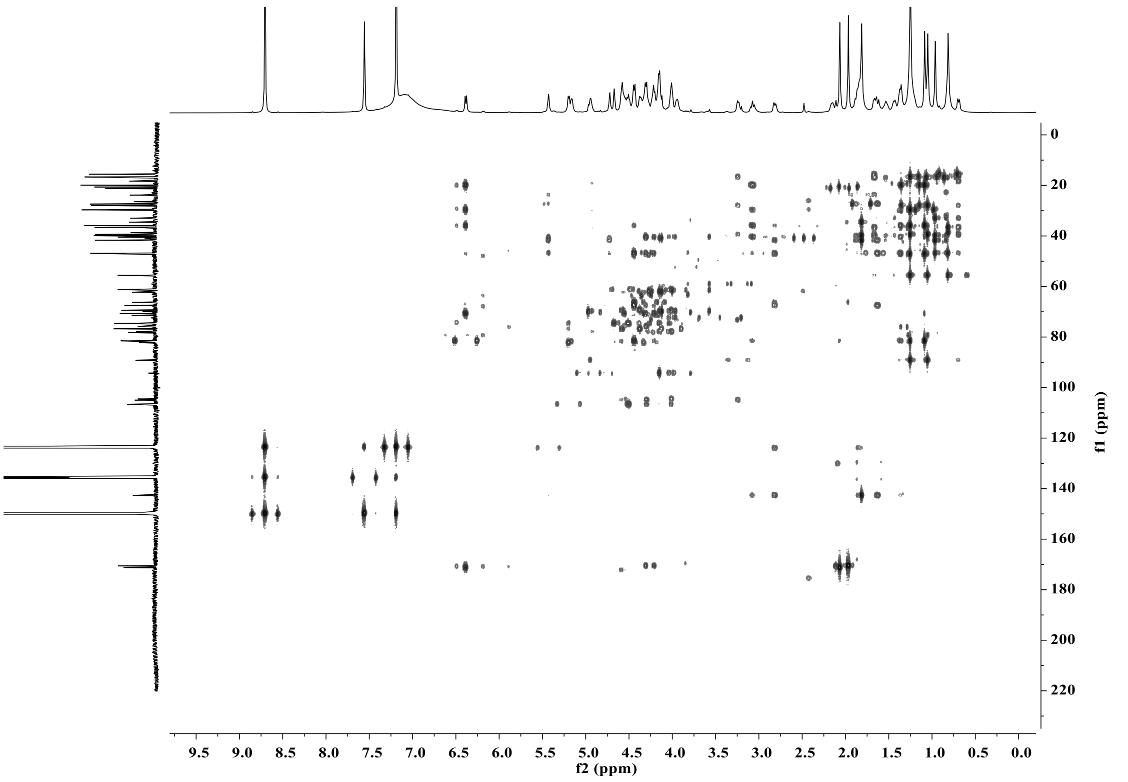

S5. HMBC Spectrum of the new compound **1**

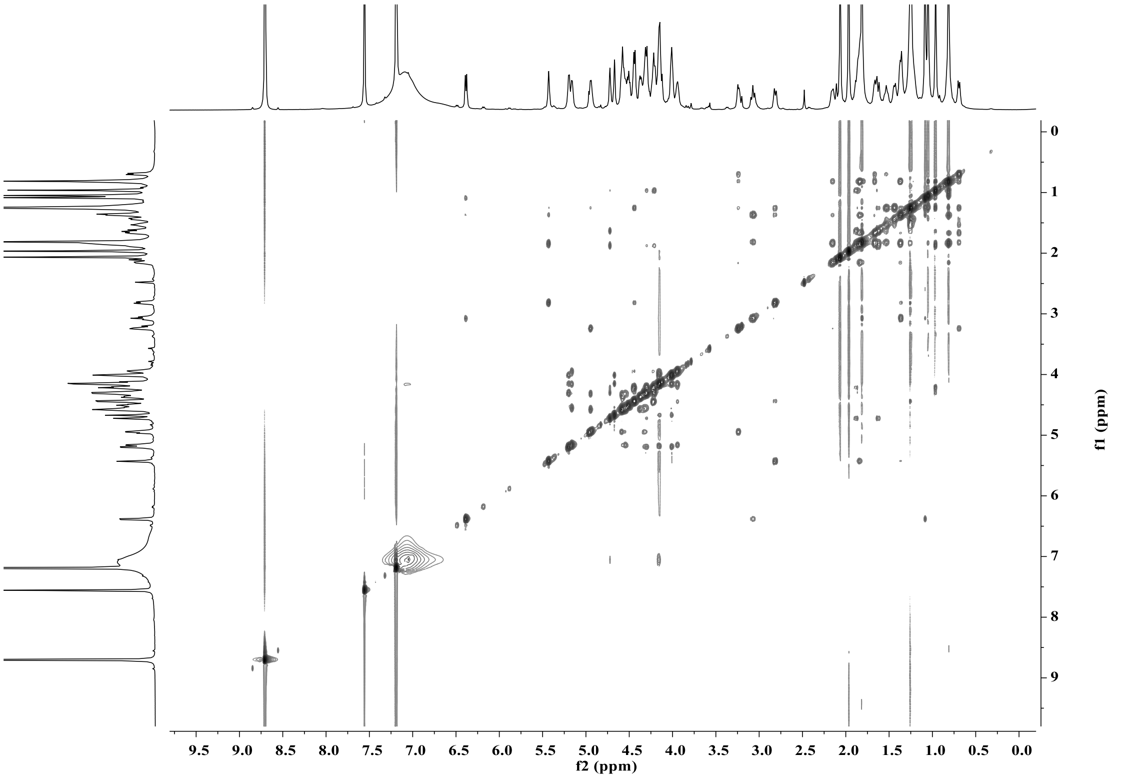

S6. NOESY Spectrum of the new compound **1**

Single Mass Analysis
Tolerance = 5.0 mDa / DBE: min = -1.5, max = 50.0
Element prediction: Off
Number of isotope peaks used for i-FIT = 3
Monoisotopic Mass, Even Electron Ions
499 formula(e) evaluated with 3 results within limits (up to 50 best isotopic matches for each mass)
Elements Used:
C: 0-500 H: 0-1000 O: 0-200

Mass	Calc. Mass	mDa	PPM	DBE	Formula	i-FIT	i-FIT Norm	Fit Conf %	C	H	O
1073.5149	1073.5169	-2.0	-1.9	12.5	C52 H81 O23	729.9	0.006	99.44	52	81	23
	1073.5110	3.9	3.6	21.5	C59 H77 O18	735.3	5.467	0.42	59	77	18
	1073.5145	0.4	0.4	43.5	C77 H69 O5	736.5	6.599	0.14	77	69	5

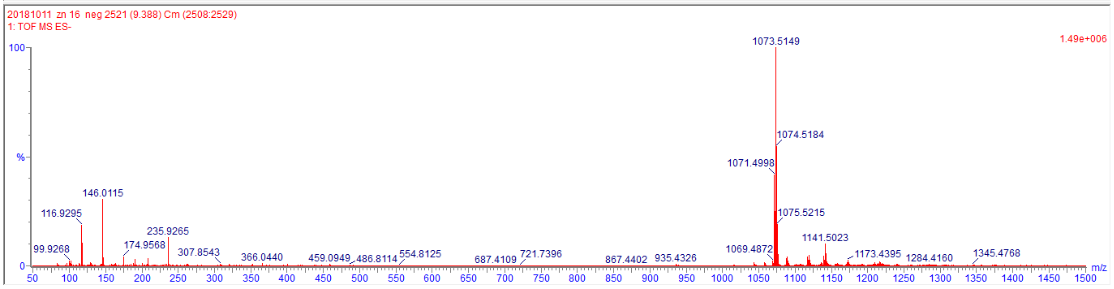

S7. HRESIMS spectrum of the new compound **1**

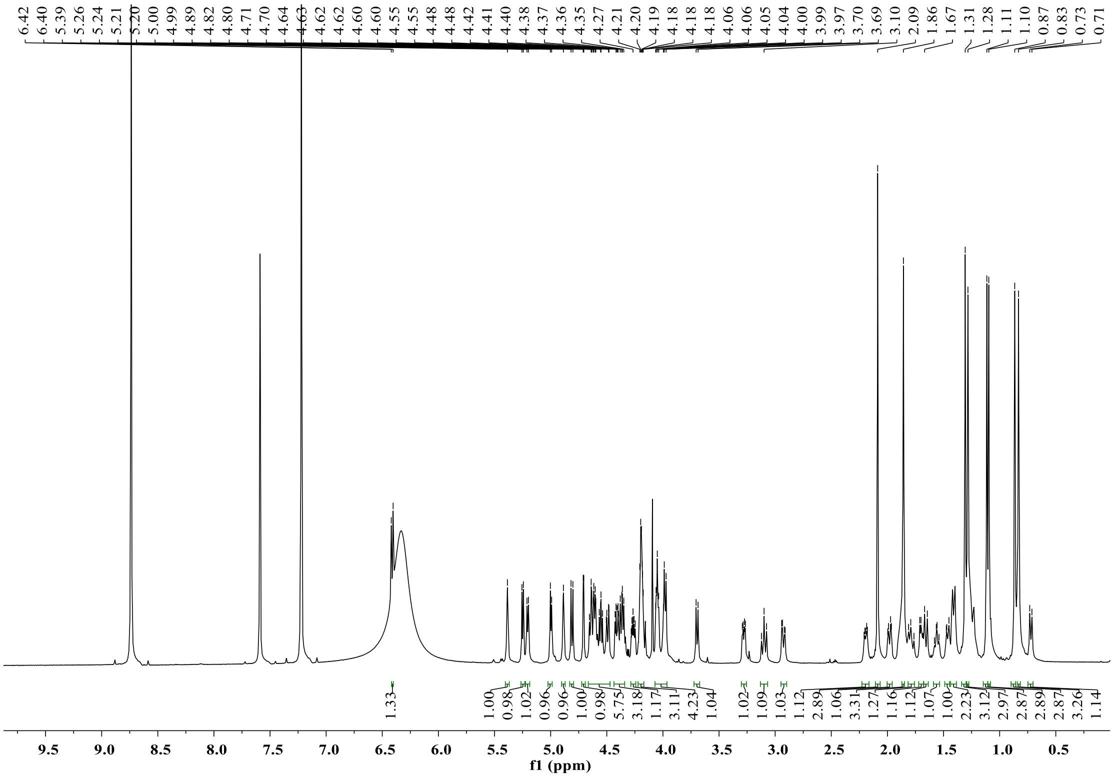

S8. ^{1}H NMR Spectrum (600 MHZ, Pyridine-d_5) of the new compound **2**

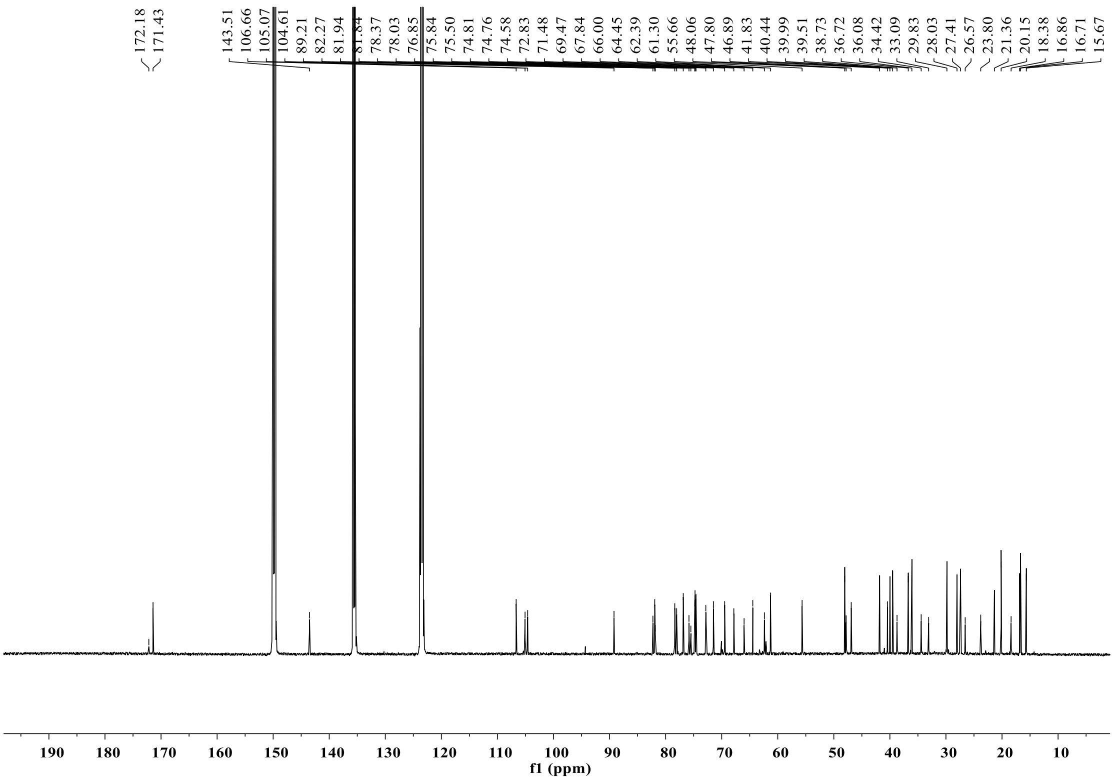

S9. ^{13}C NMR Spectrum (150 MHZ, Pyridine-d_5) of the new compound **2**

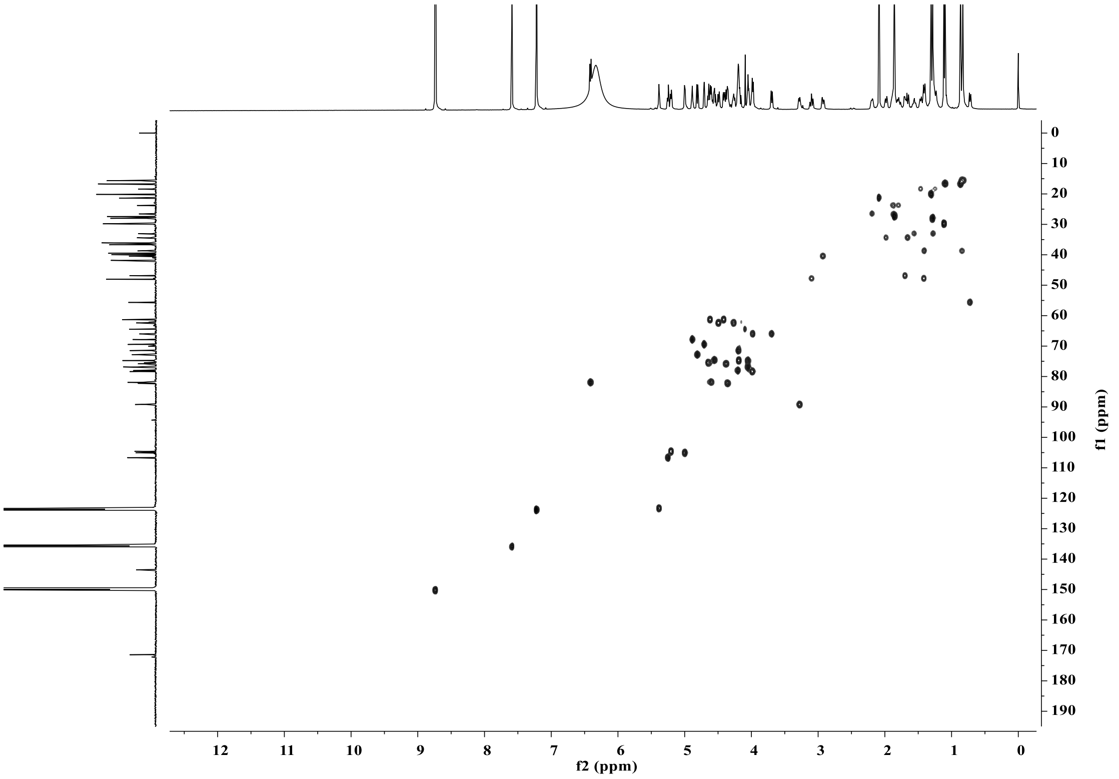

S10. HSQC Spectrum of the new compound **2**

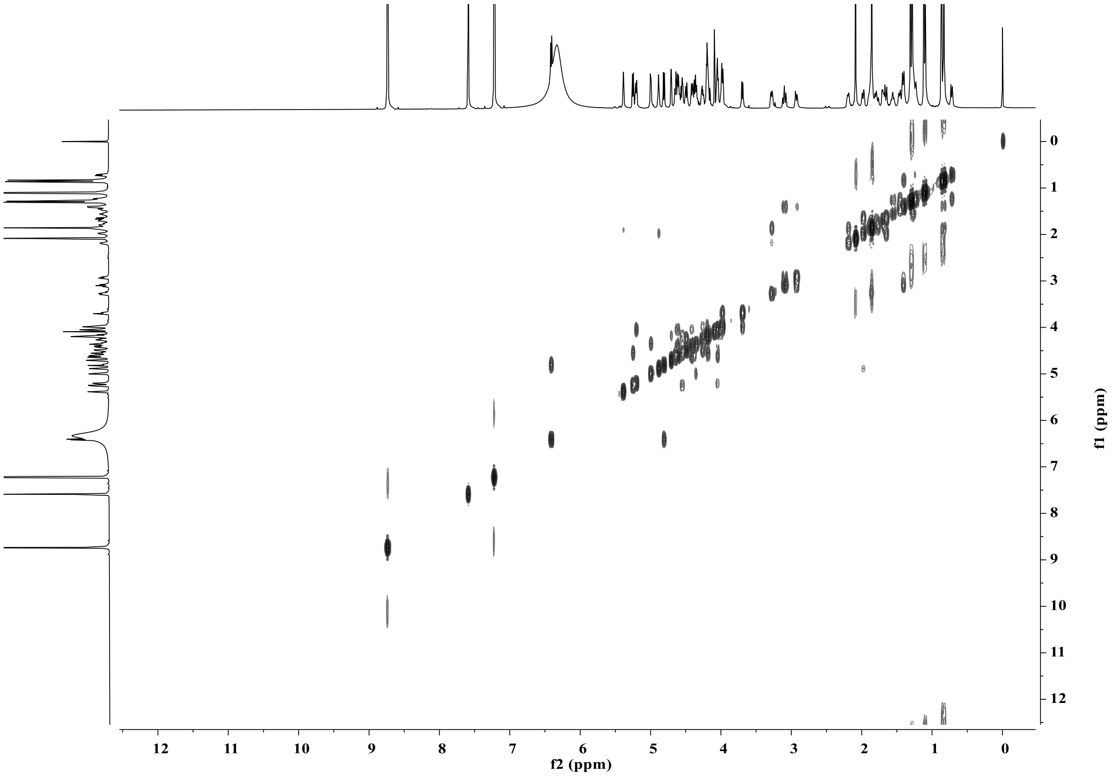

S11. ^{1}H-^{1}H COSY Spectrum of the new compound **2**

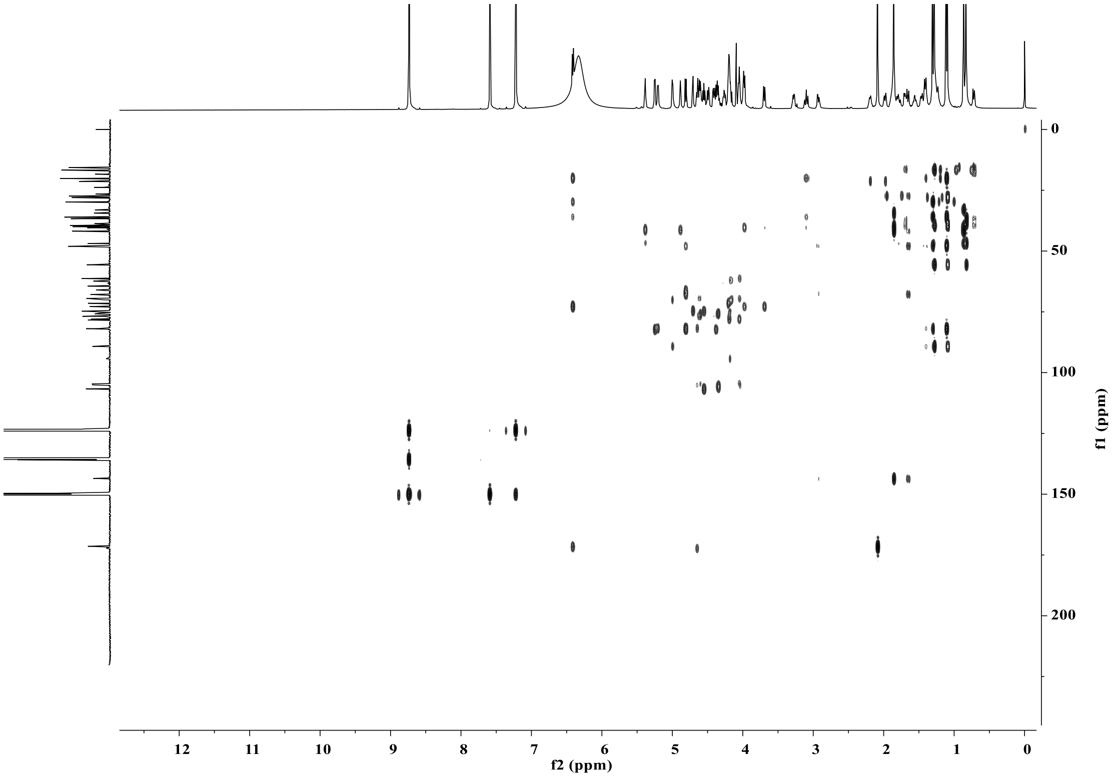

S12. HMBC Spectrum of the new compound **2**

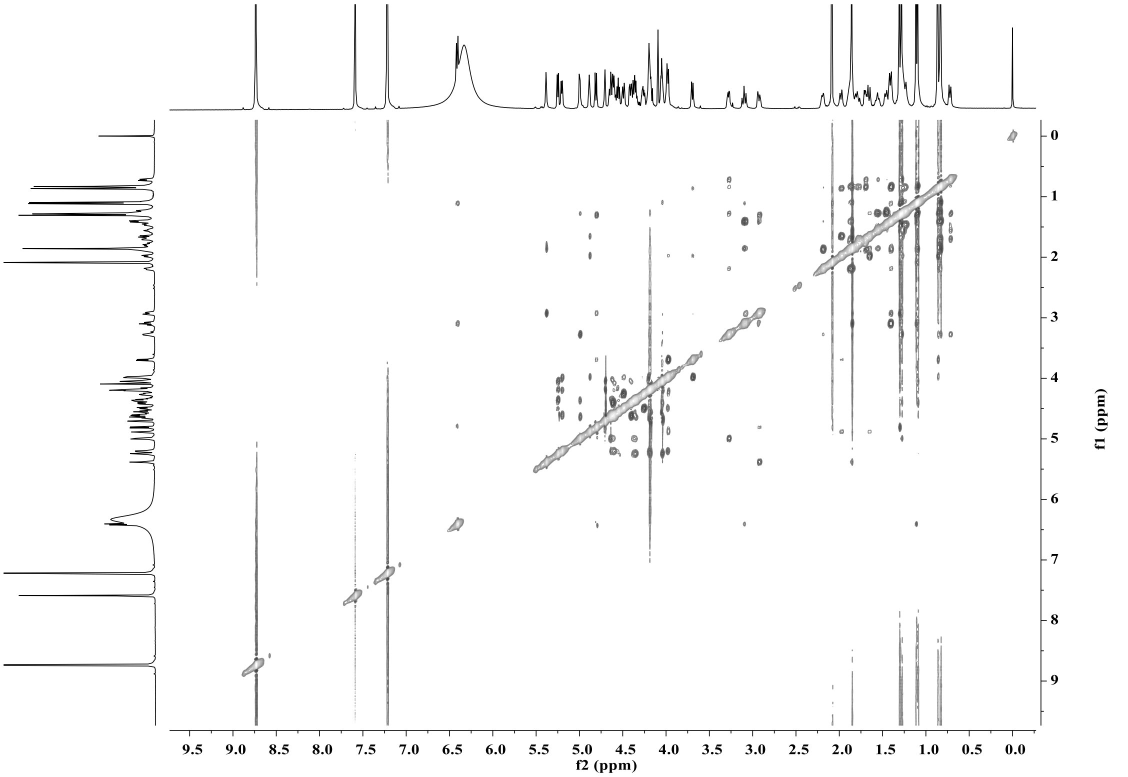

S13. NOESY Spectrum of the new compound **2**

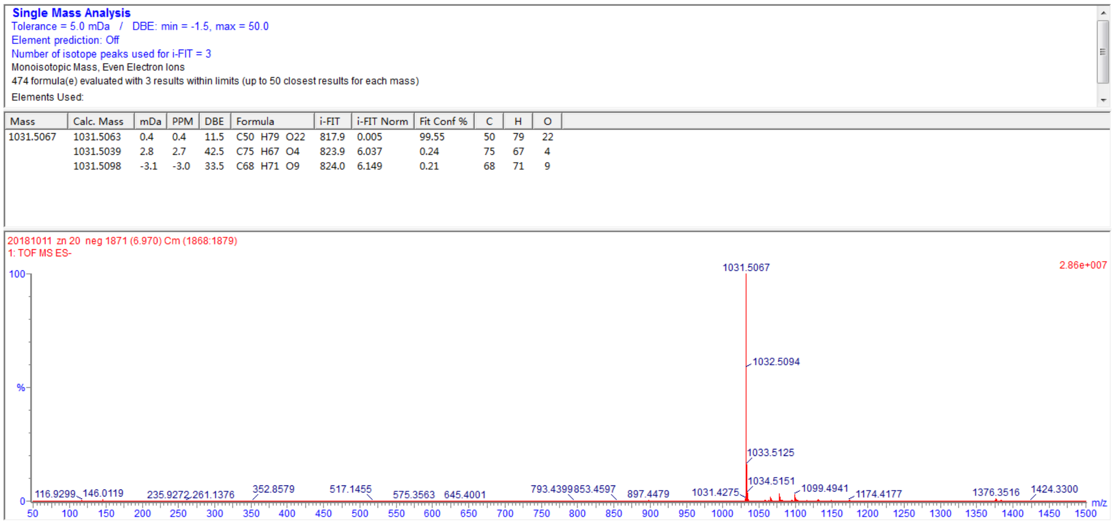

Single Mass Analysis
Tolerance = 5.0 mDa / DBE: min = -1.5, max = 50.0
Element prediction: Off
Number of isotope peaks used for i-FIT = 3
Monoisotopic Mass, Even Electron Ions
474 formula(e) evaluated with 3 results within limits (up to 50 closest results for each mass)
Elements Used:

Mass	Calc. Mass	mDa	PPM	DBE	Formula	i-FIT	i-FIT Norm	Fit Conf %	C	H	O
1031.5067	1031.5063	0.4	0.4	11.5	C50 H79 O22	817.9	0.005	99.55	50	79	22
	1031.5039	2.8	2.7	42.5	C75 H67 O4	823.9	6.037	0.24	75	67	4
	1031.5098	-3.1	-3.0	33.5	C68 H71 O9	824.0	6.149	0.21	68	71	9

S14. HRESIMS spectrum of the new compound **2**

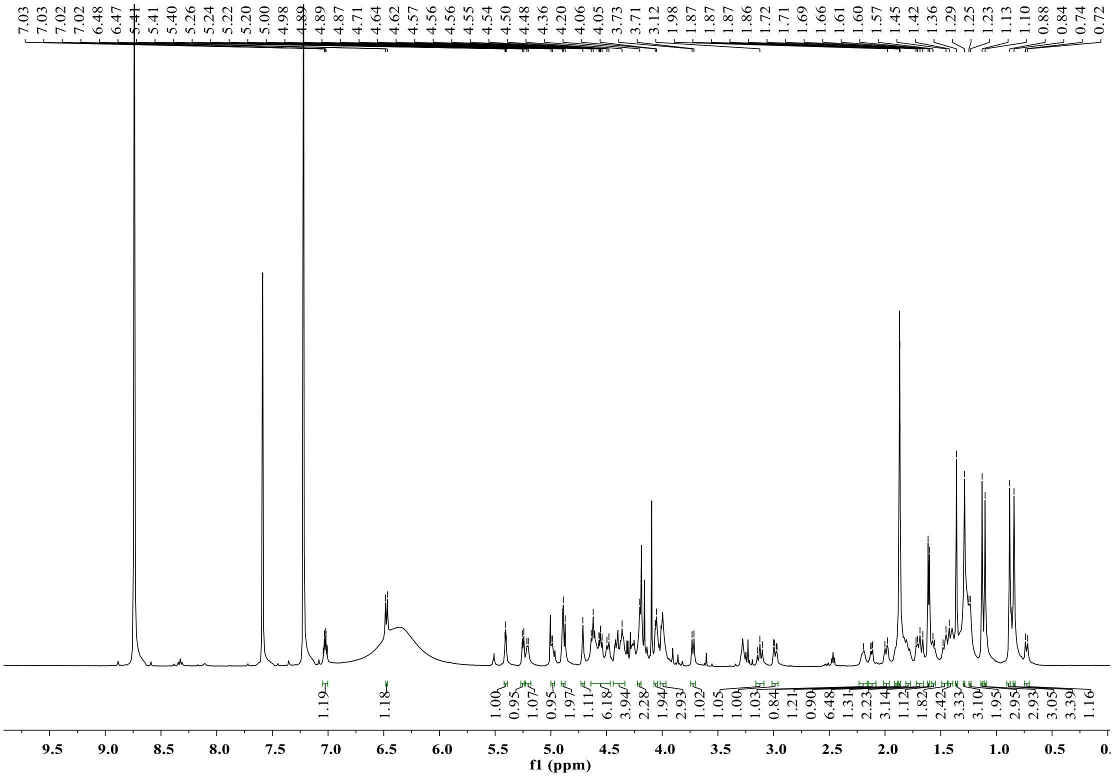

S15. ^{1}H NMR Spectrum (600 MHZ, Pyridine-d_5) of the new compound **3**

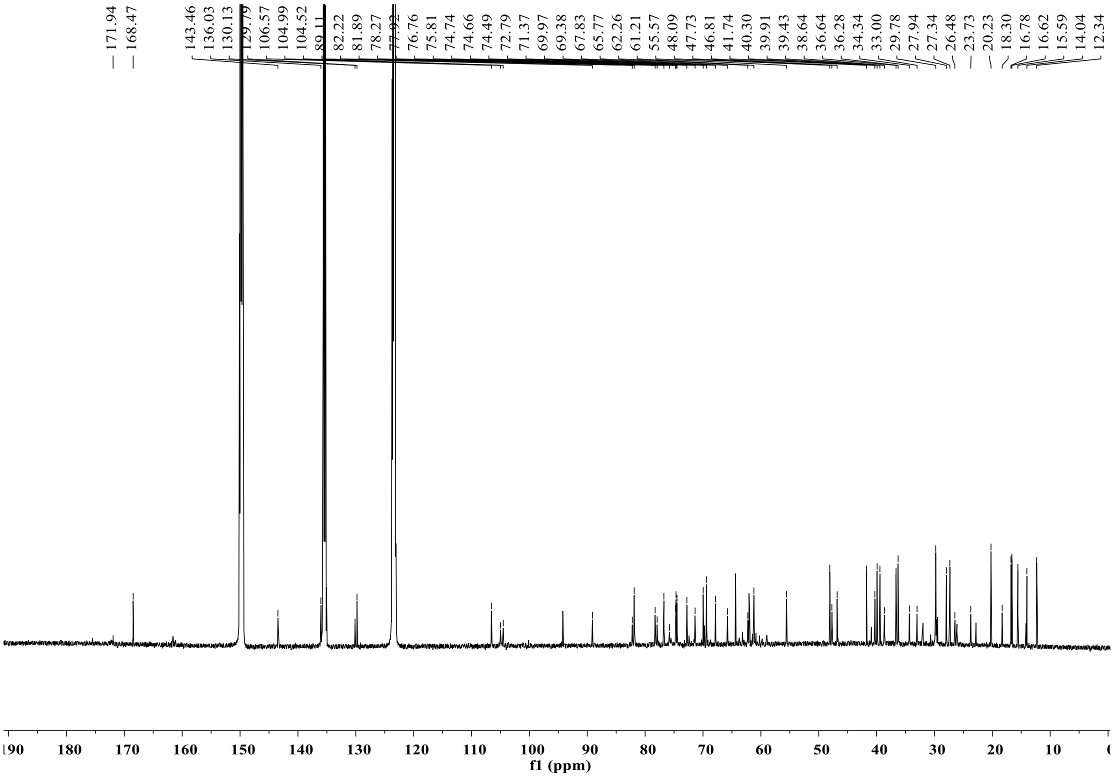

S16. ^{13}C NMR Spectrum (150 MHZ, Pyridine-d_5) of the new compound **3**

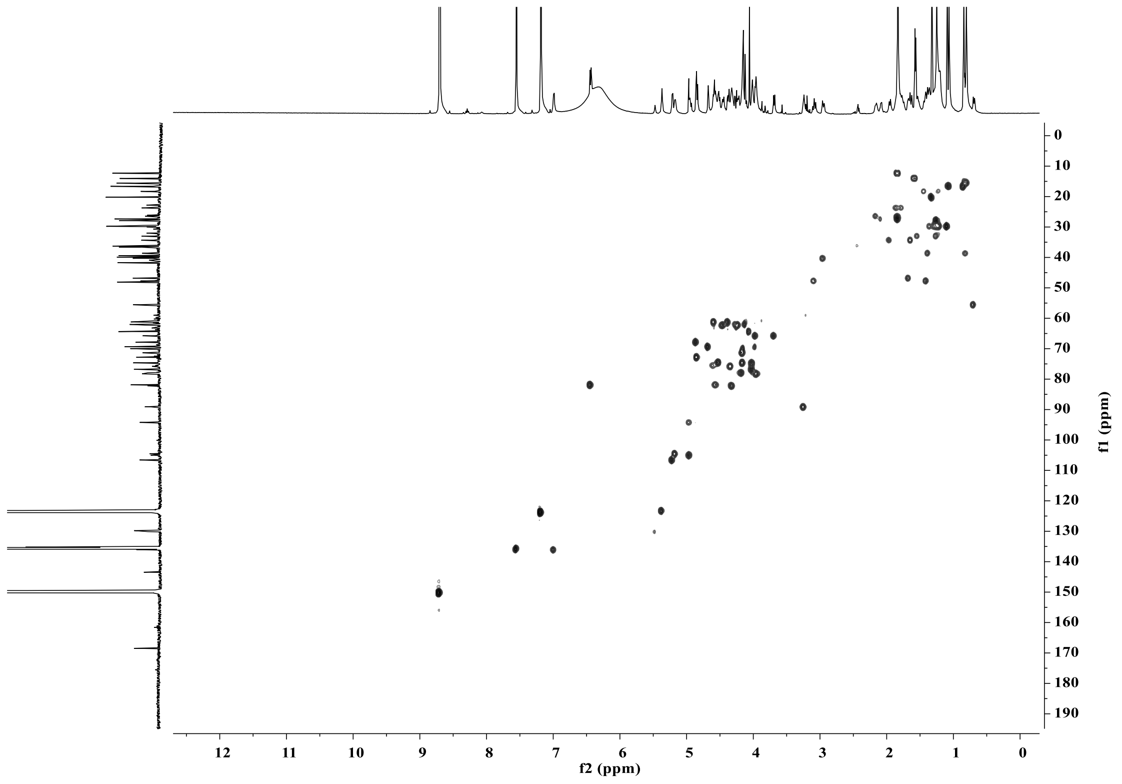

S17. HSQC Spectrum of the new compound **3**

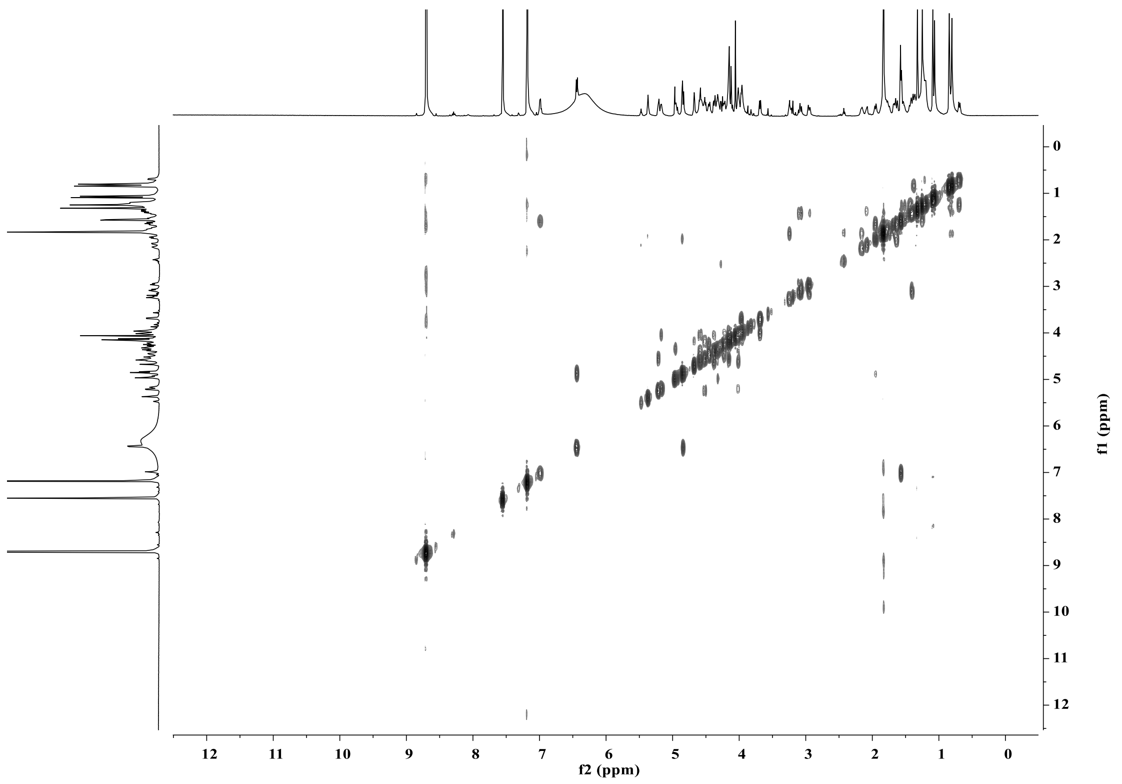

S18. ^{1}H-^{1}H COSY Spectrum of the new compound **3**

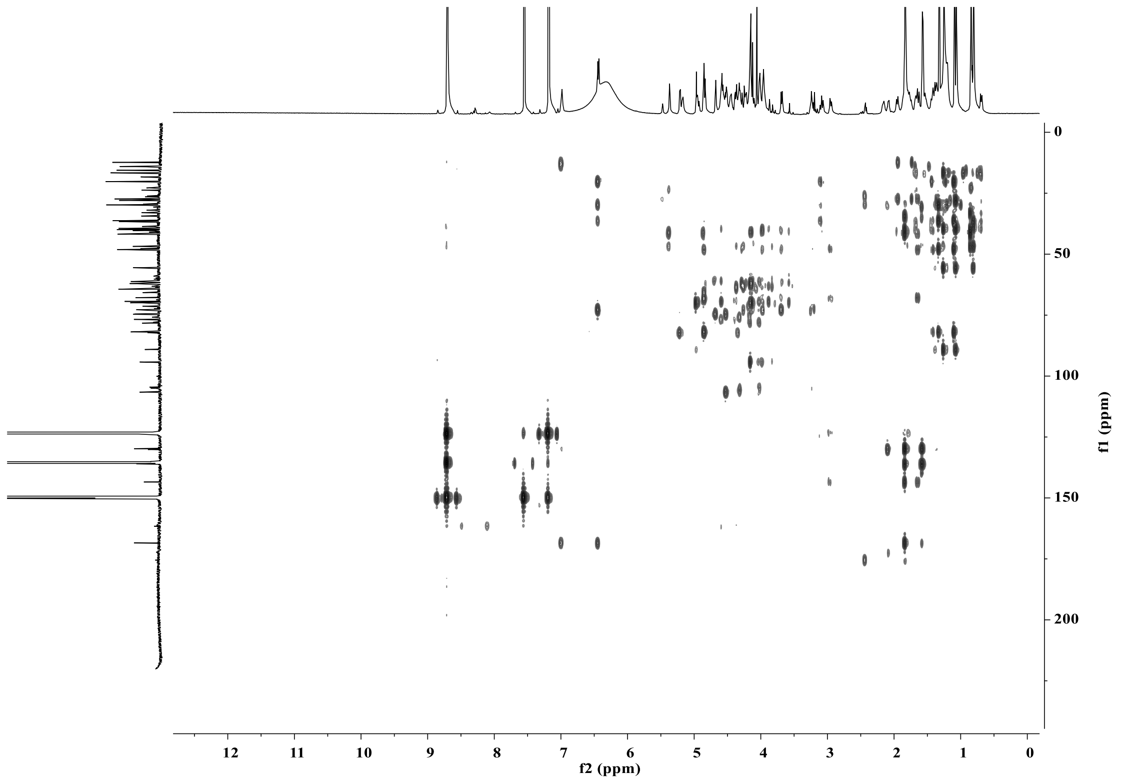

S19. HMBC Spectrum of the new compound **3**

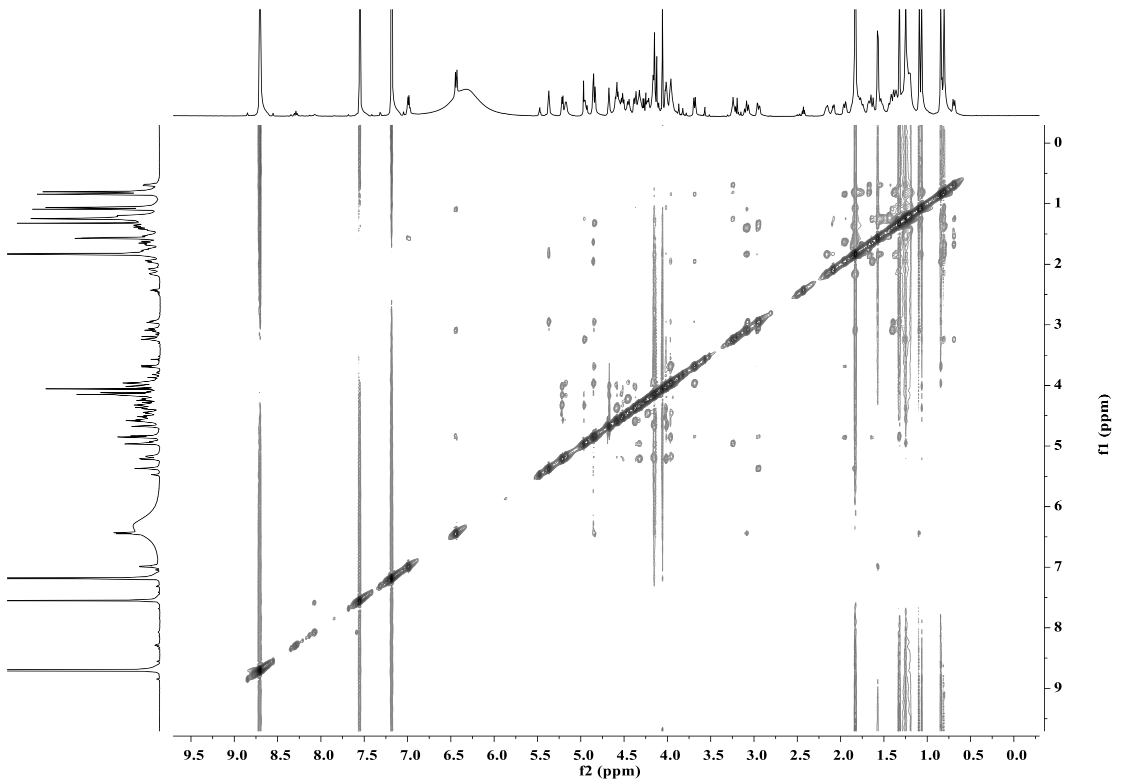

S20. NOESY Spectrum of the new compound **3**

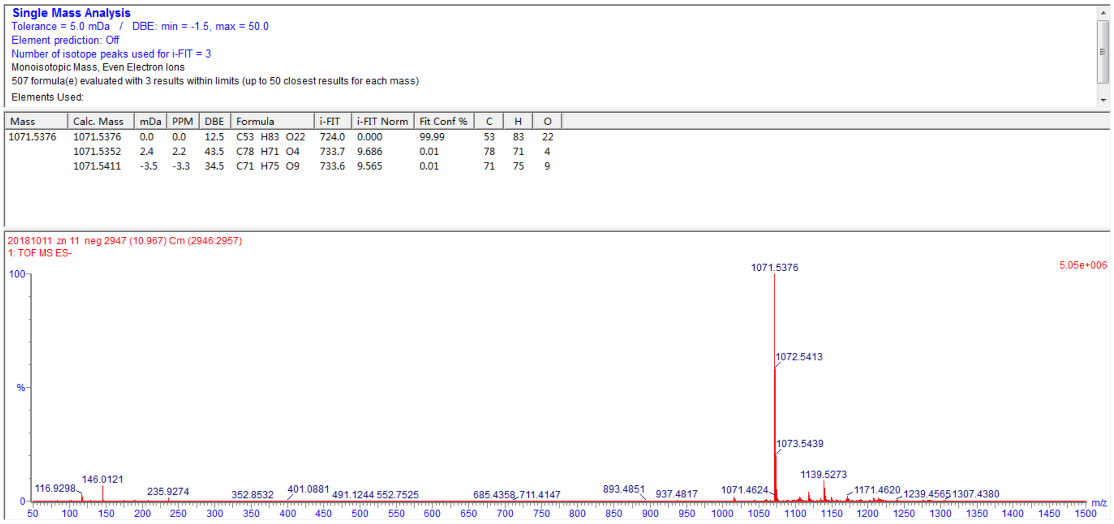

Mass	Calc. Mass	mDa	PPM	DBE	Formula	i-FIT	i-FIT Norm	Fit Conf %	C	H	O
1071.5376	1071.5376	0.0	0.0	12.5	C53 H83 O22	724.0	0.000	99.99	53	83	22
	1071.5352	2.4	2.2	43.5	C78 H71 O4	733.7	9.686	0.01	78	71	4
	1071.5411	-3.5	-3.3	34.5	C71 H75 O9	733.6	9.565	0.01	71	75	9

S21. HRESIMS spectrum of the new compound **3**

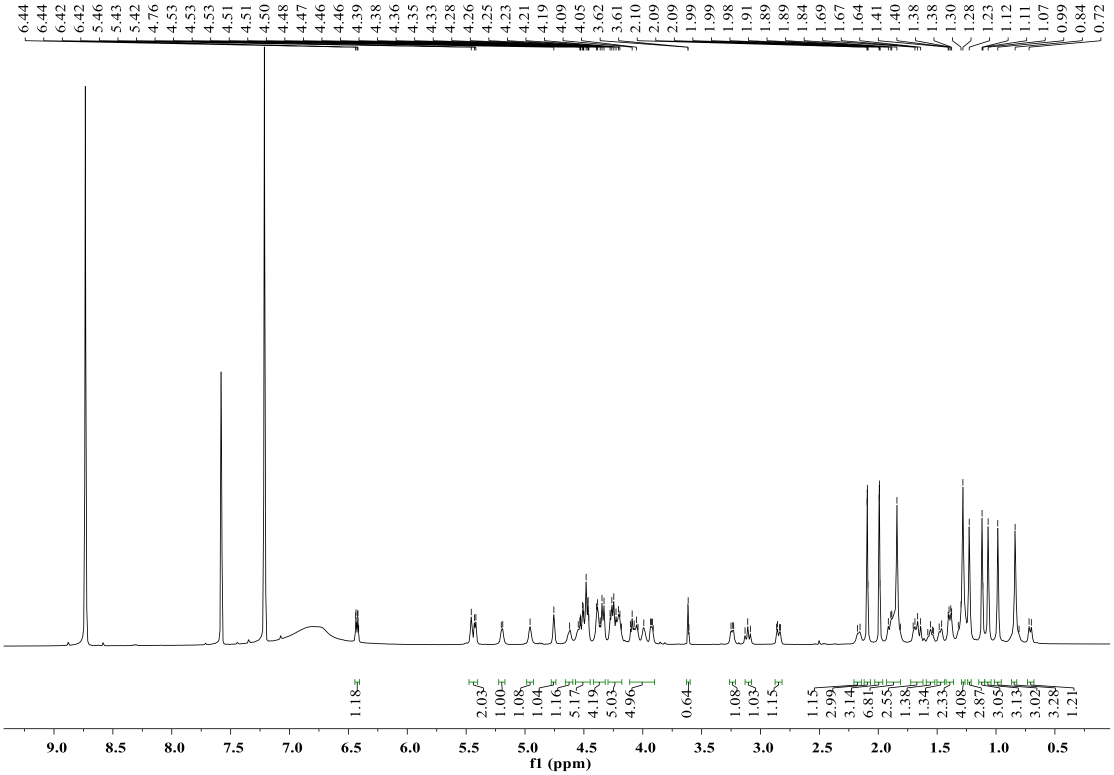

S22. ^{1}H NMR Spectrum (600 MHZ, Pyridine-d_5) of the new compound **4**

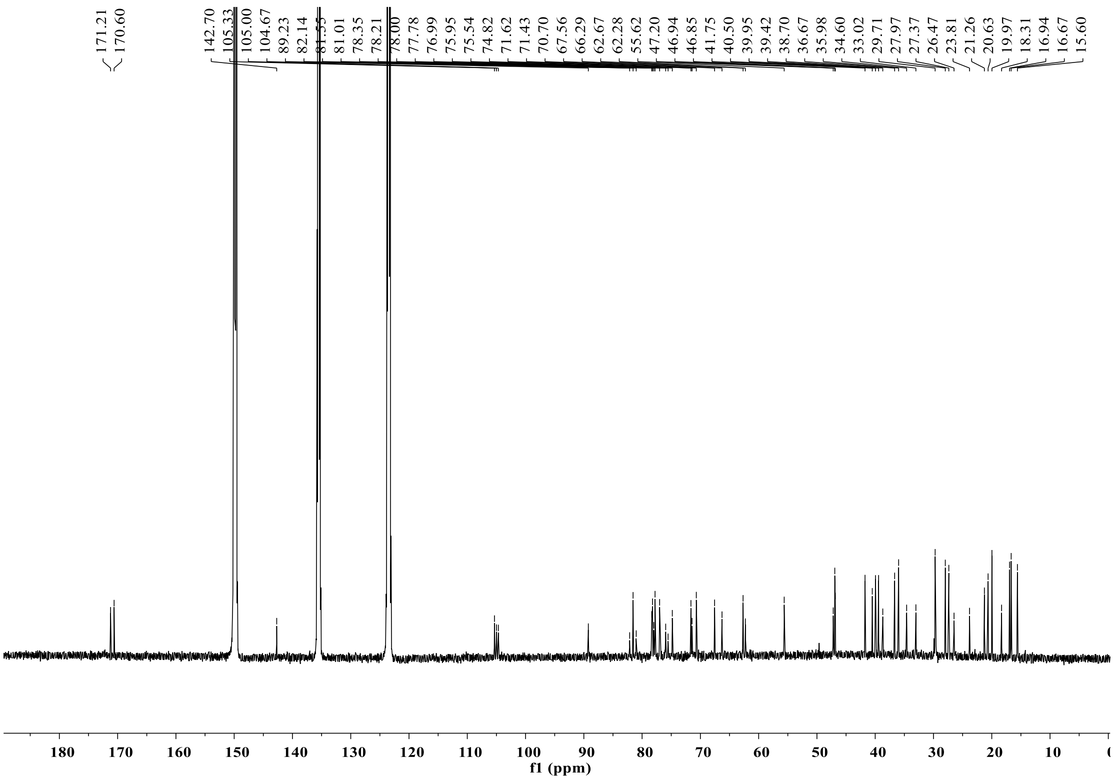

S23. ^{13}C NMR Spectrum (150 MHZ, Pyridine-d_5) of the new compound **4**

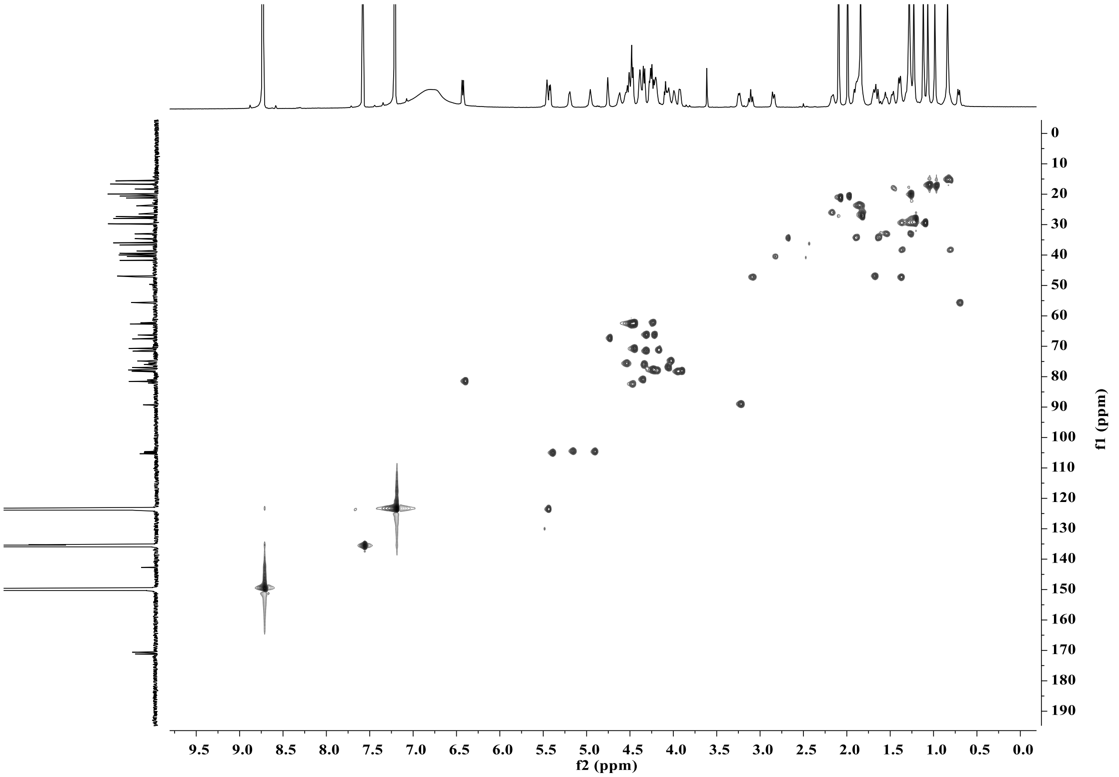

S24. HSQC Spectrum of the new compound **4**

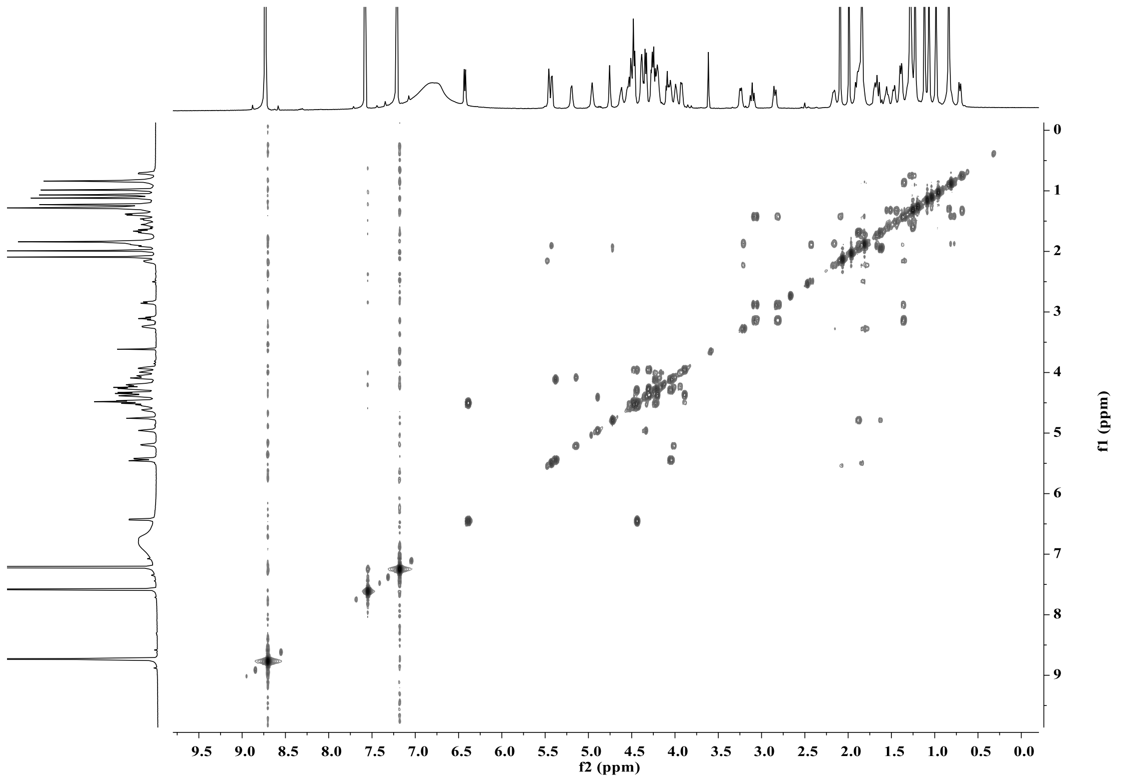

S25. ^{1}H-^{1}H COSY Spectrum of the new compound **4**

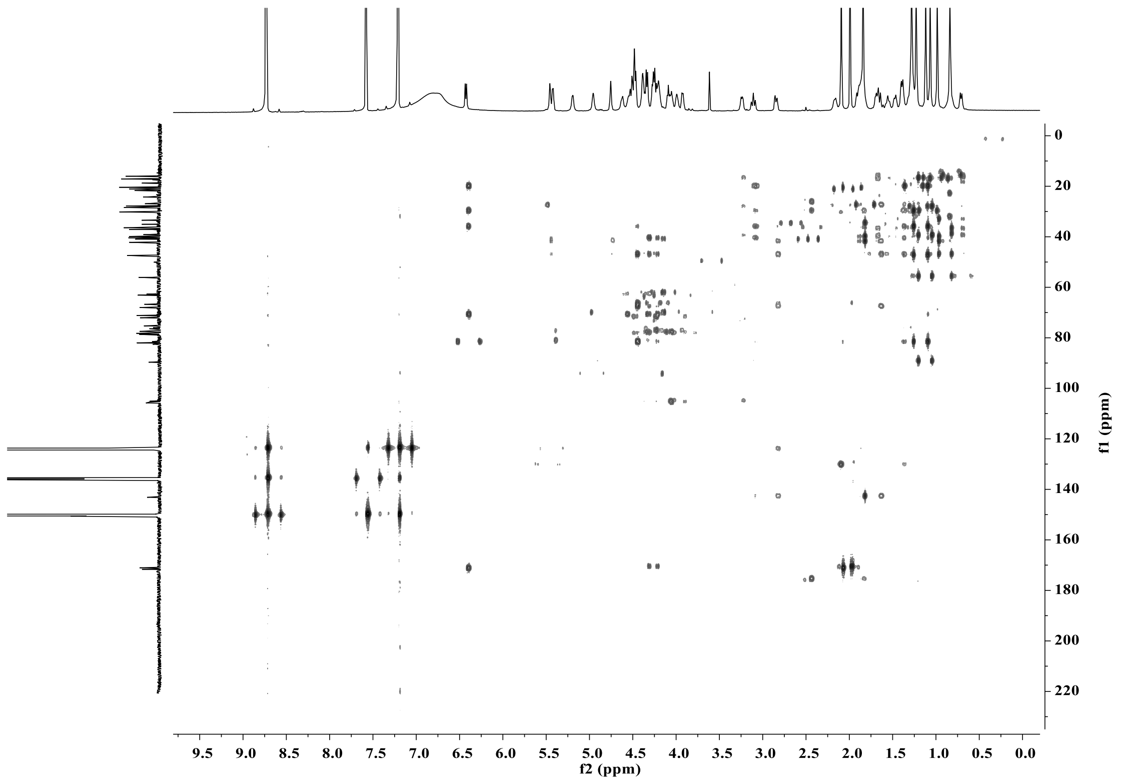

S26. HMBC Spectrum of the new compound **4**

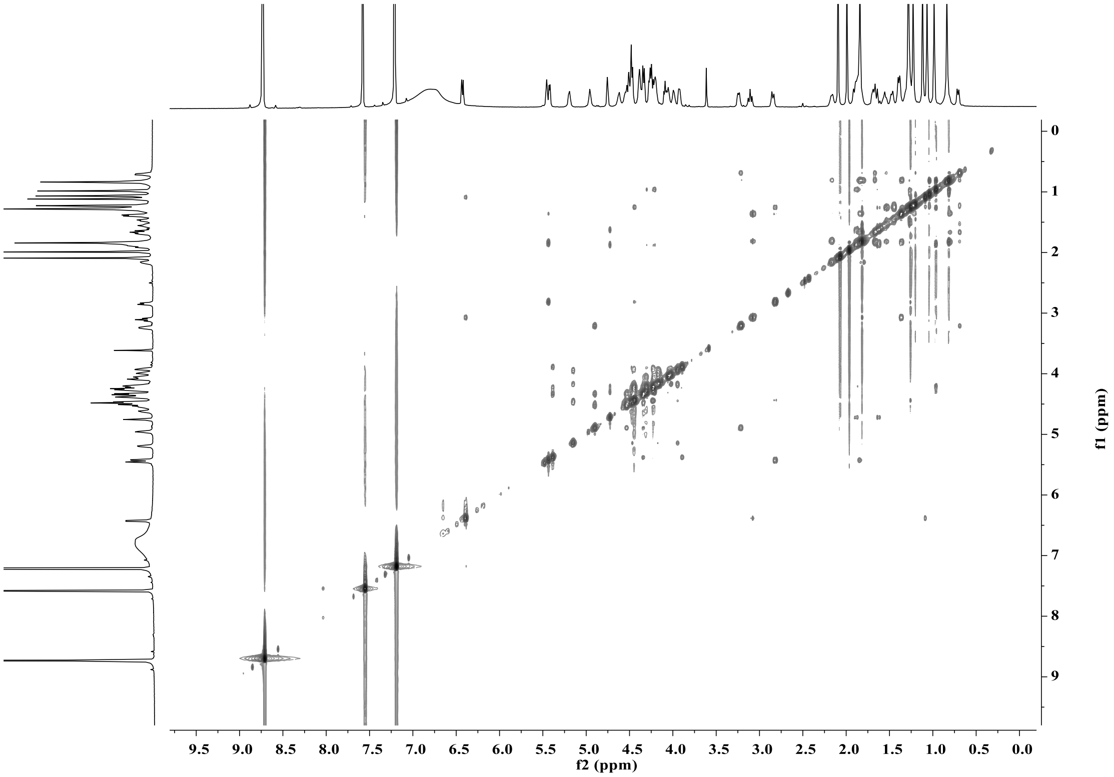

S27. NOESY Spectrum of the new compound **4**

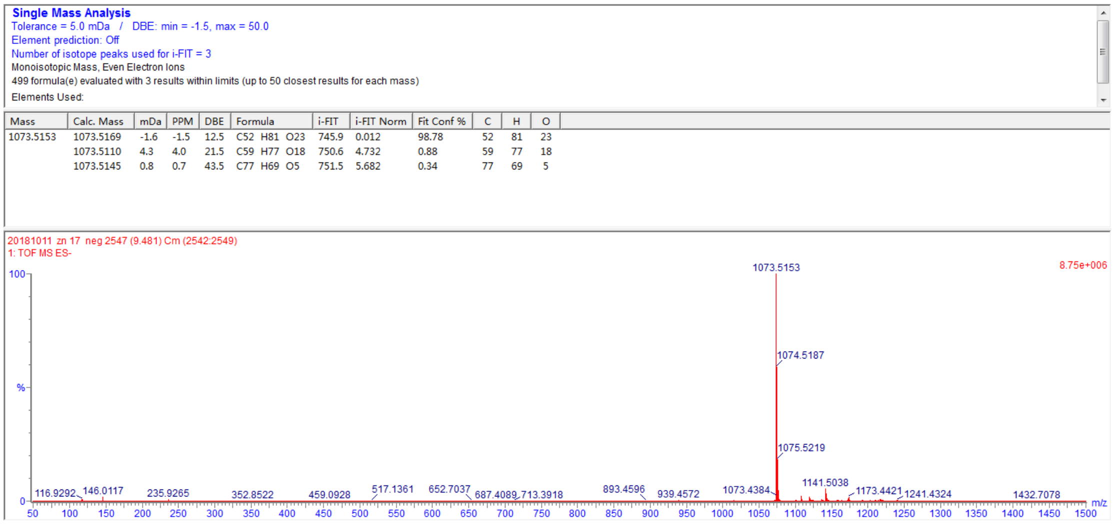

Mass	Calc. Mass	mDa	PPM	DBE	Formula	i-FIT	i-FIT Norm	Fit Conf %	C	H	O
1073.5153	1073.5169	-1.6	-1.5	12.5	C52 H81 O23	745.9	0.012	98.78	52	81	23
	1073.5110	4.3	4.0	21.5	C59 H77 O18	750.6	4.732	0.88	59	77	18
	1073.5145	0.8	0.7	43.5	C77 H69 O5	751.5	5.682	0.34	77	69	5

S28. HRESIMS spectrum of the new compound **4**

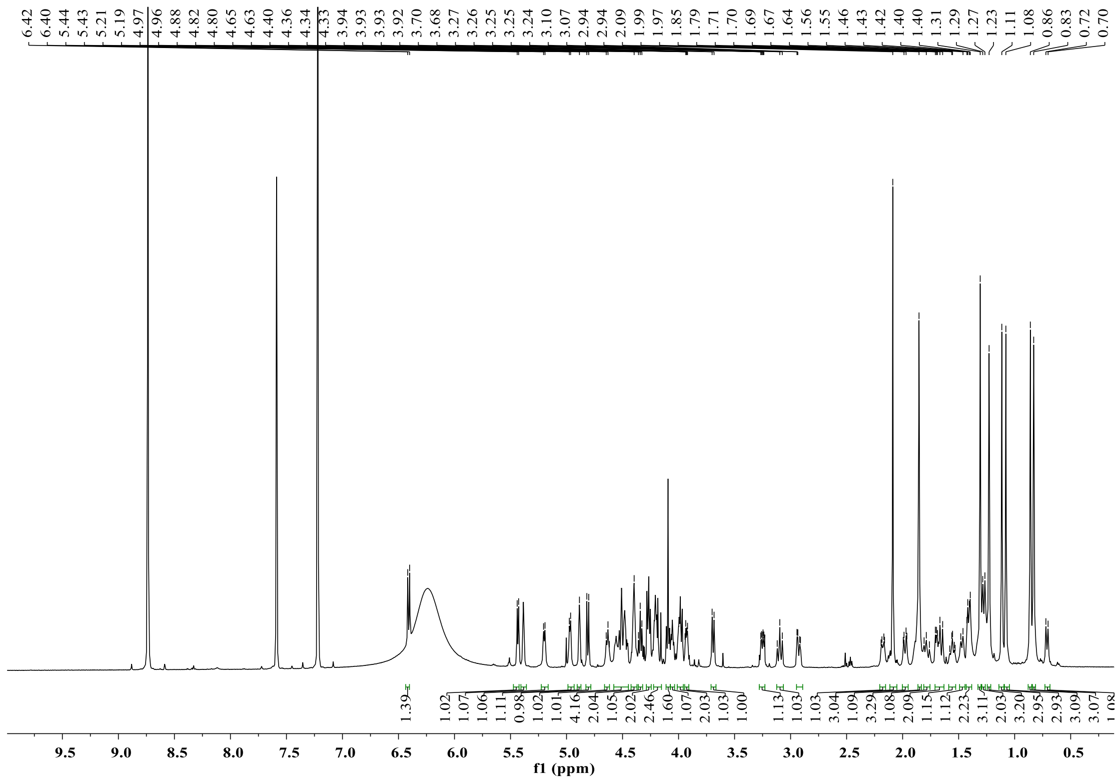

S29. ^{1}H NMR Spectrum (600 MHZ, Pyridine-d_5) of the new compound **5**

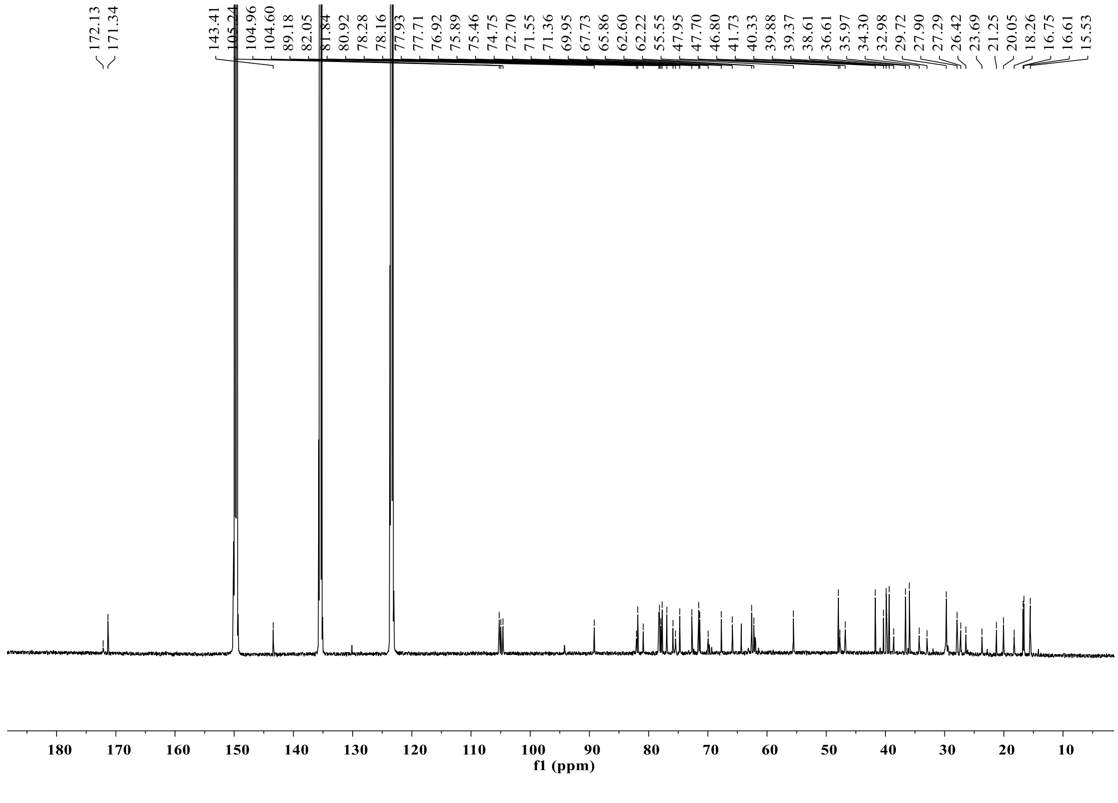

S30. ^{13}C NMR Spectrum (150 MHZ, Pyridine-d_5) of the new compound **5**

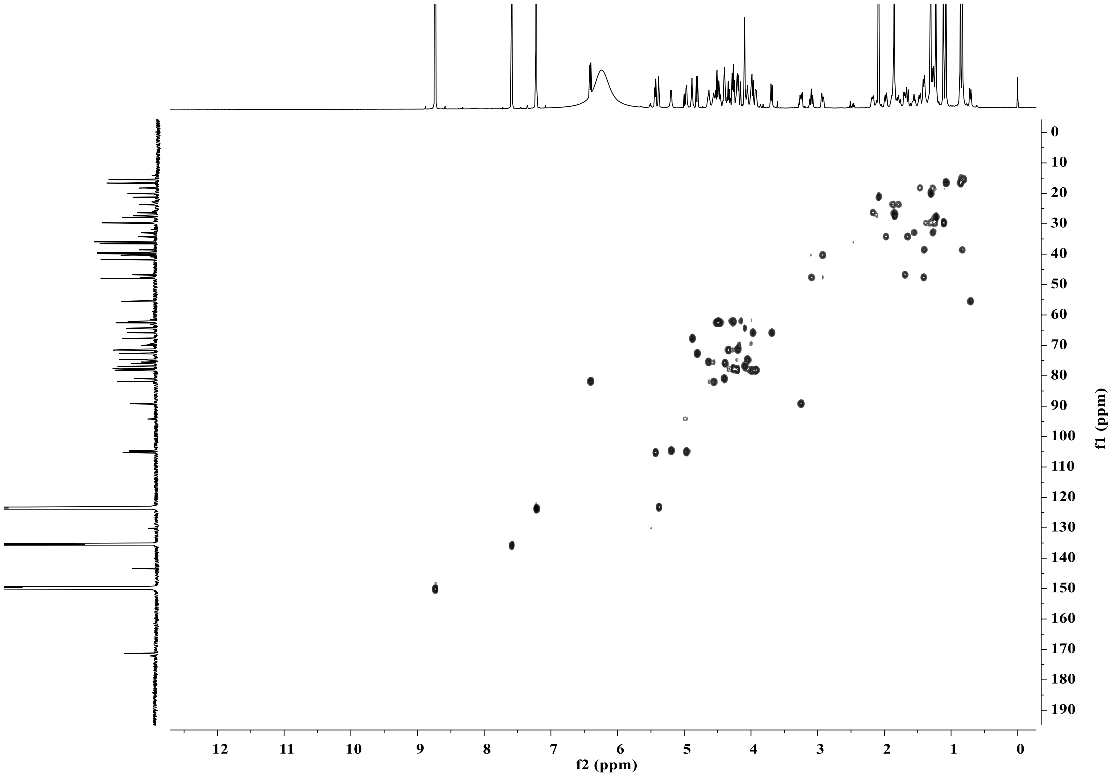

S31. HSQC Spectrum of the new compound **5**

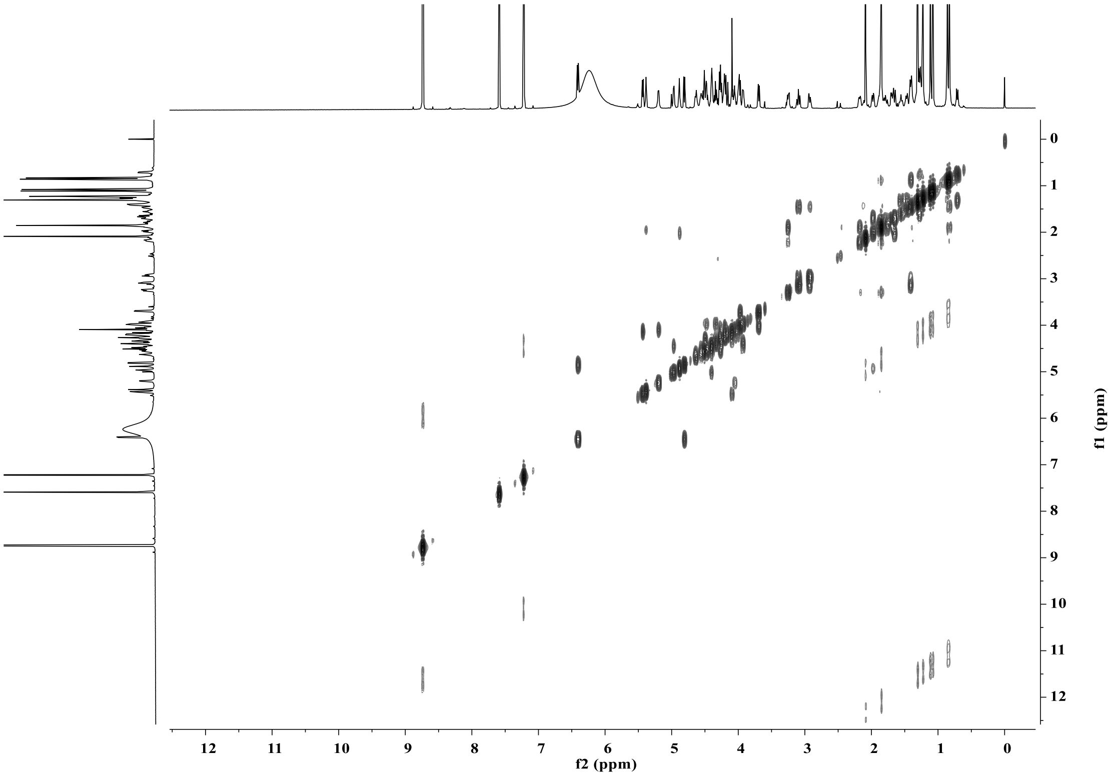

S32. ^{1}H-^{1}H COSY Spectrum of the new compound **5**

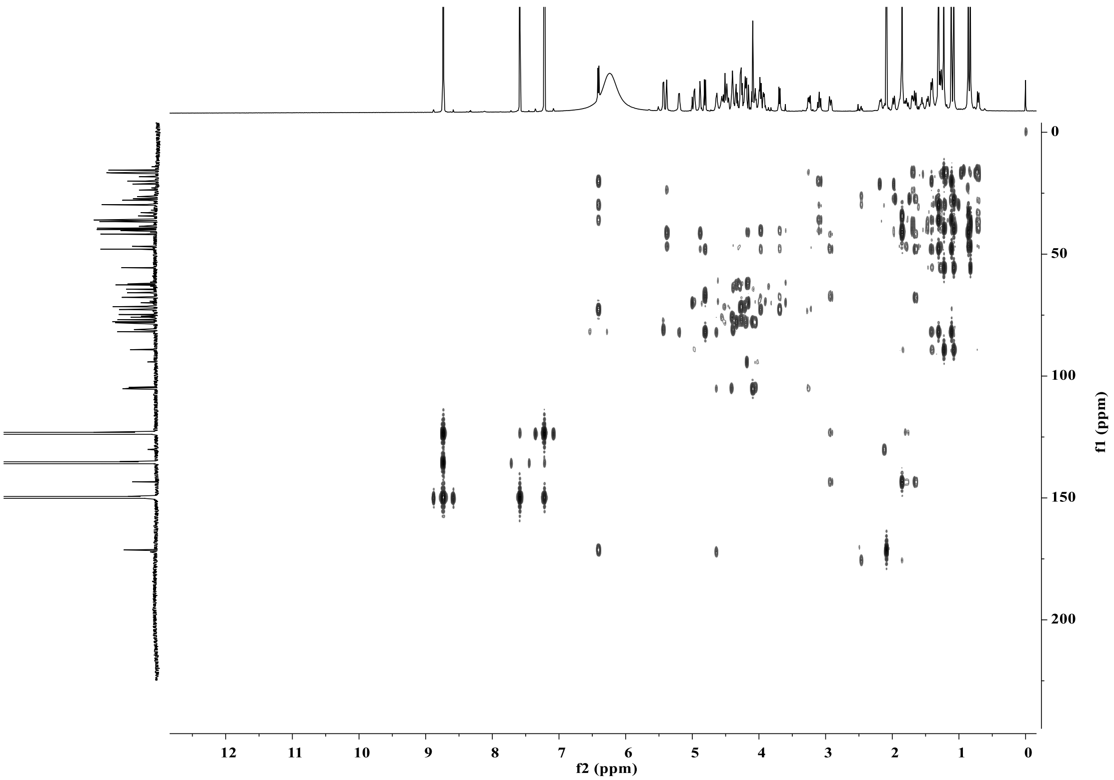

S33. HMBC Spectrum of the new compound **5**

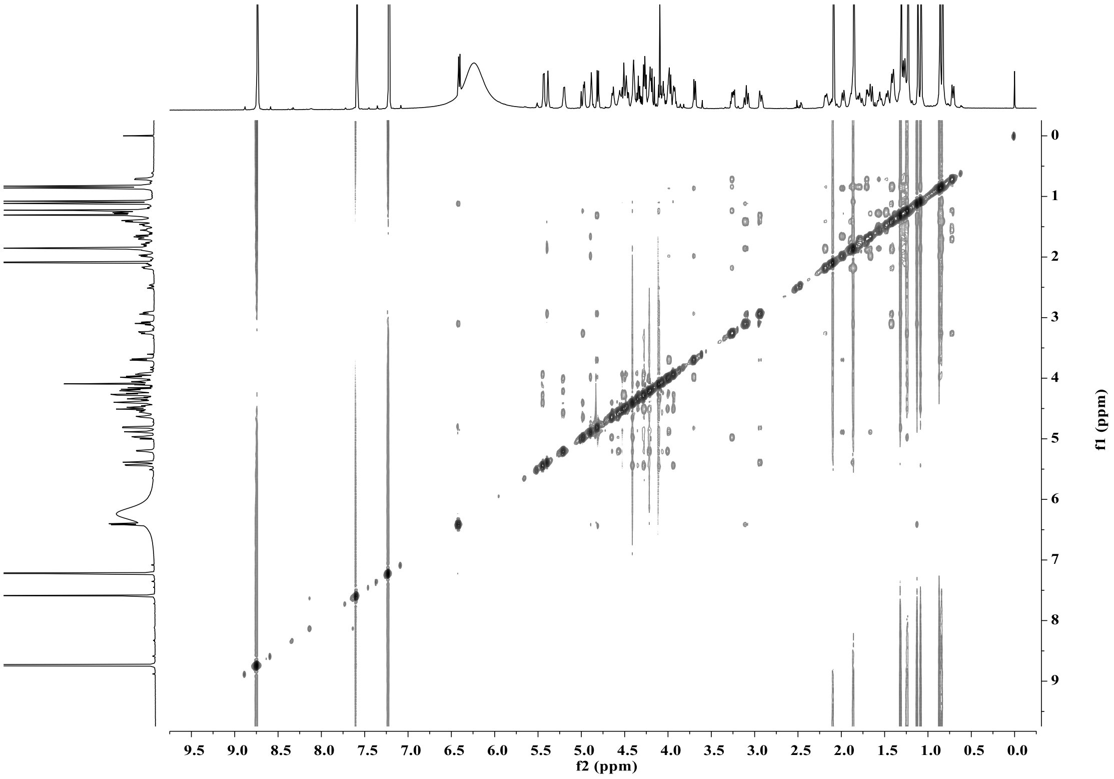

S34. NOESY Spectrum of the new compound **5**

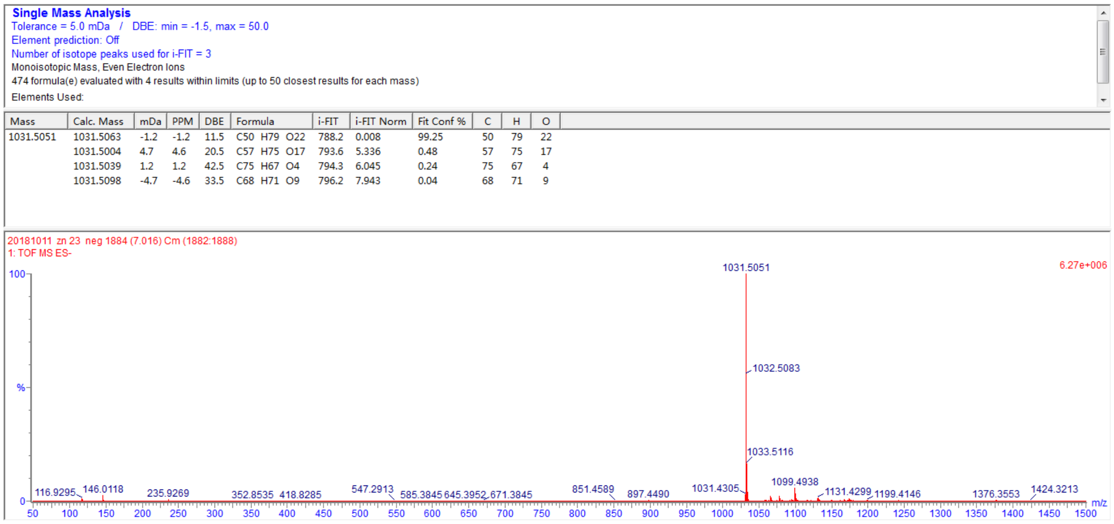

S35. HRESIMS spectrum of the new compound **5**

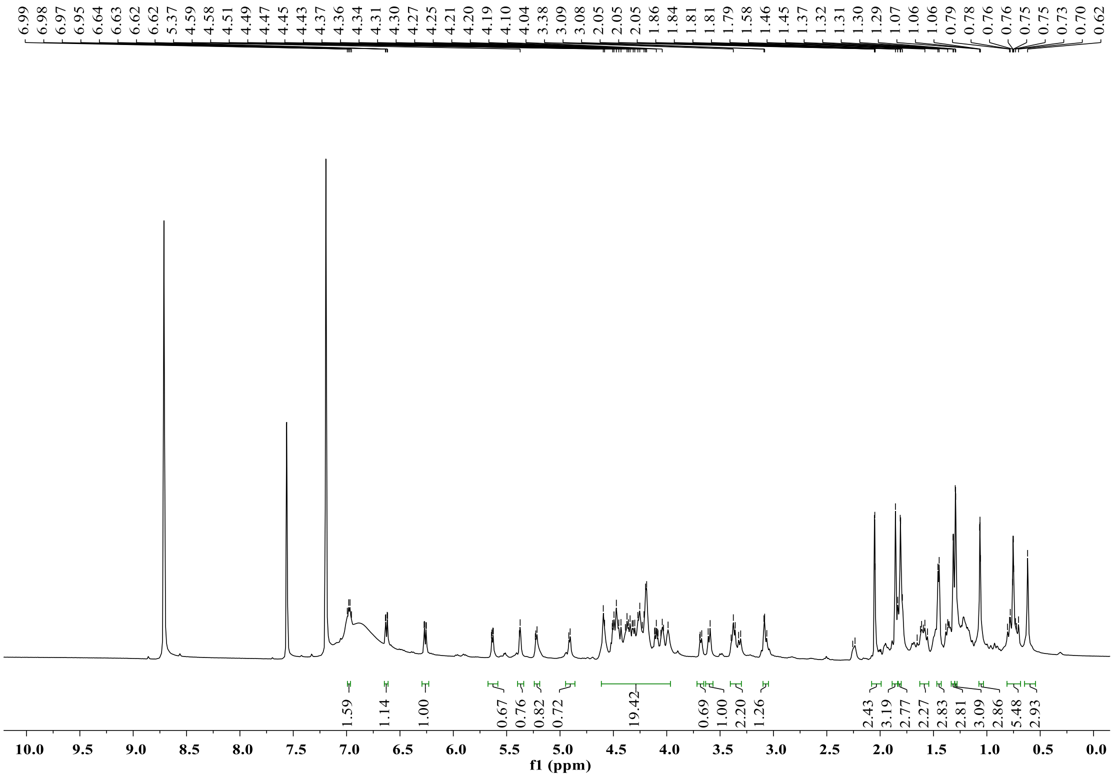

S36. ^{1}H NMR Spectrum (600 MHZ, Pyridine-d_5) of the new compound **6**

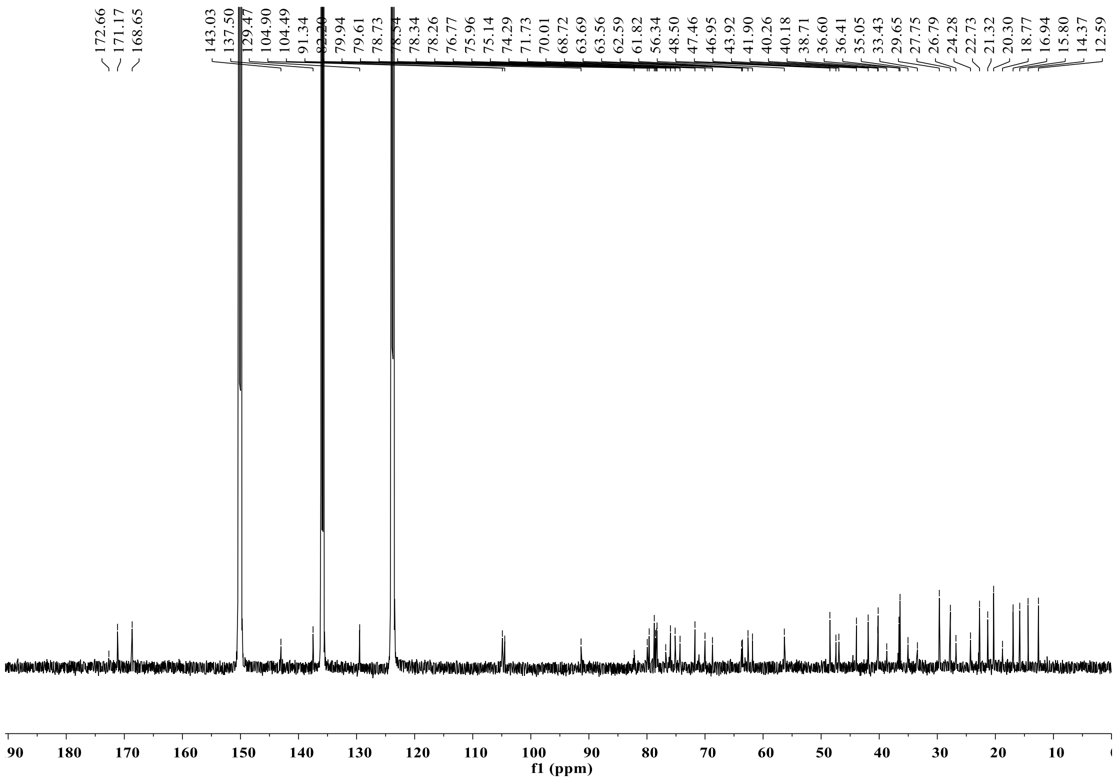

S37. ^{13}C NMR Spectrum (150 MHZ, Pyridine-d_5) of the new compound **6**

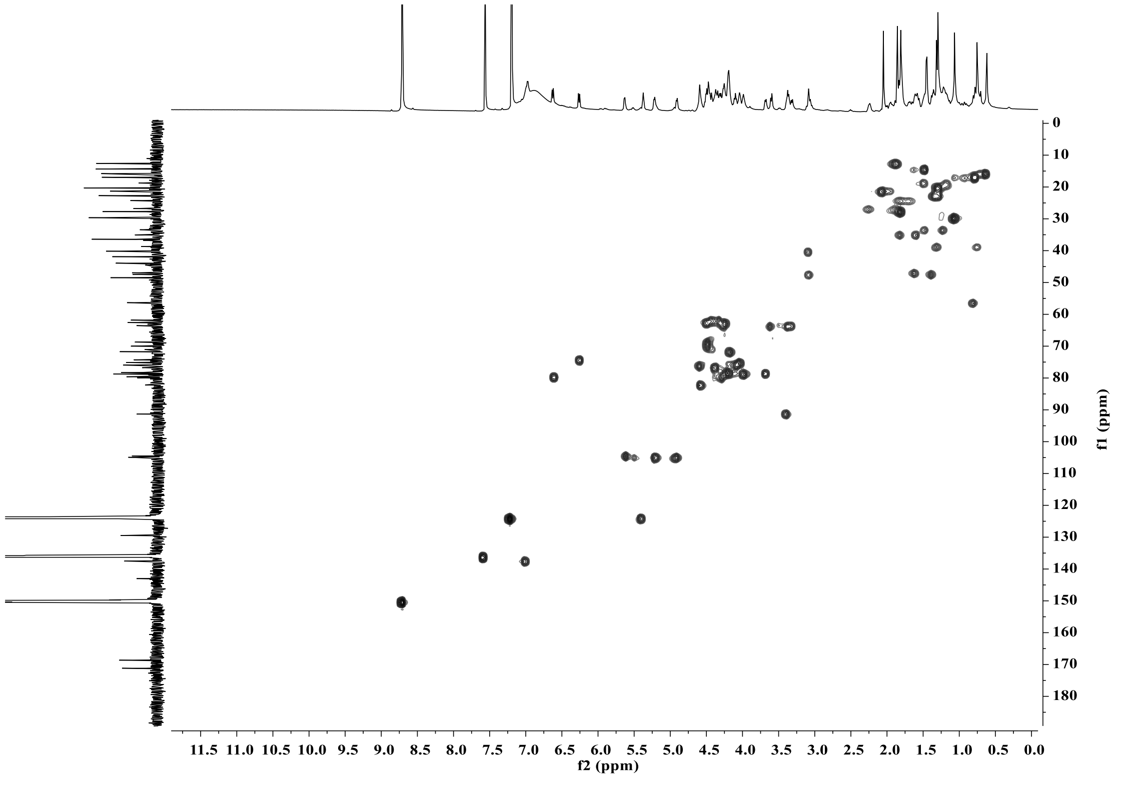

S38. HSQC Spectrum of the new compound **6**

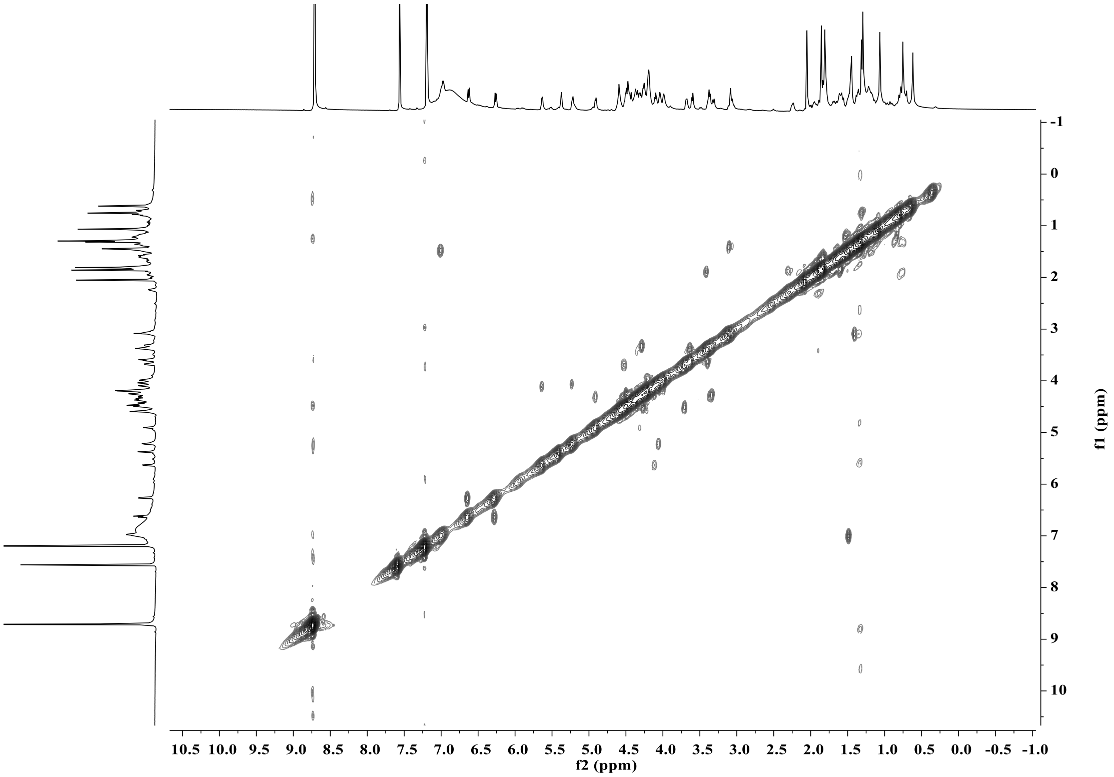

S39. ^{1}H-^{1}H COSY Spectrum of the new compound **6**

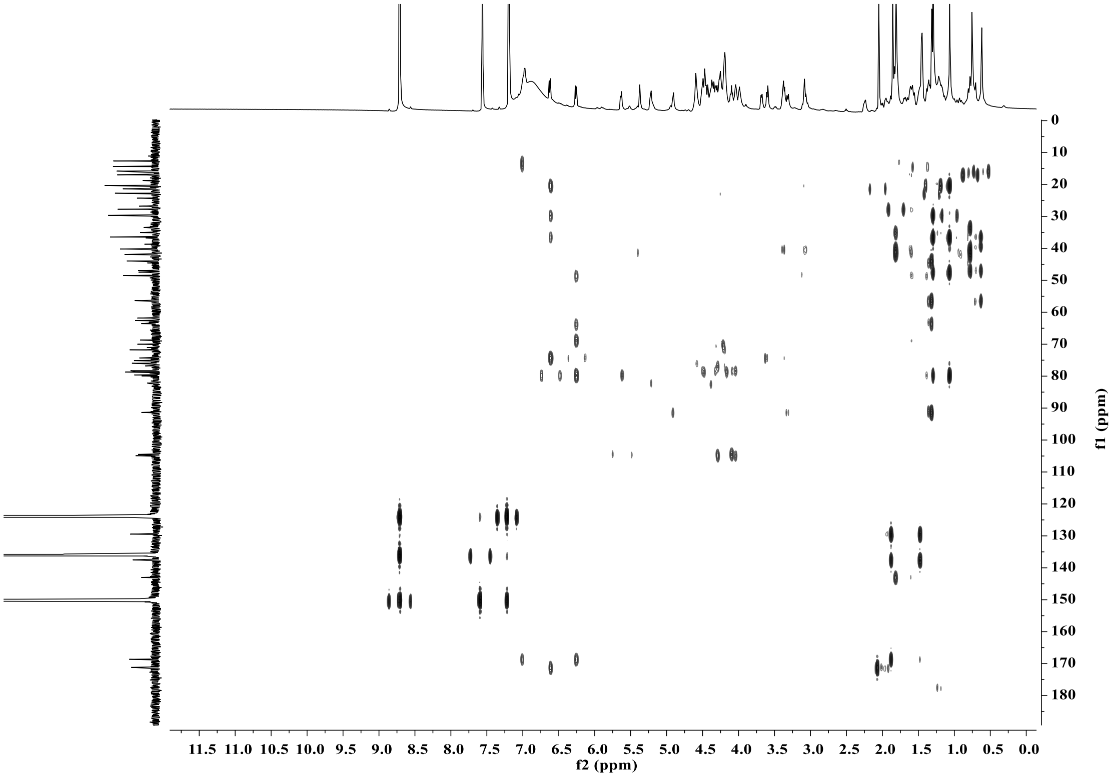

S40. HMBC Spectrum of the new compound **6**

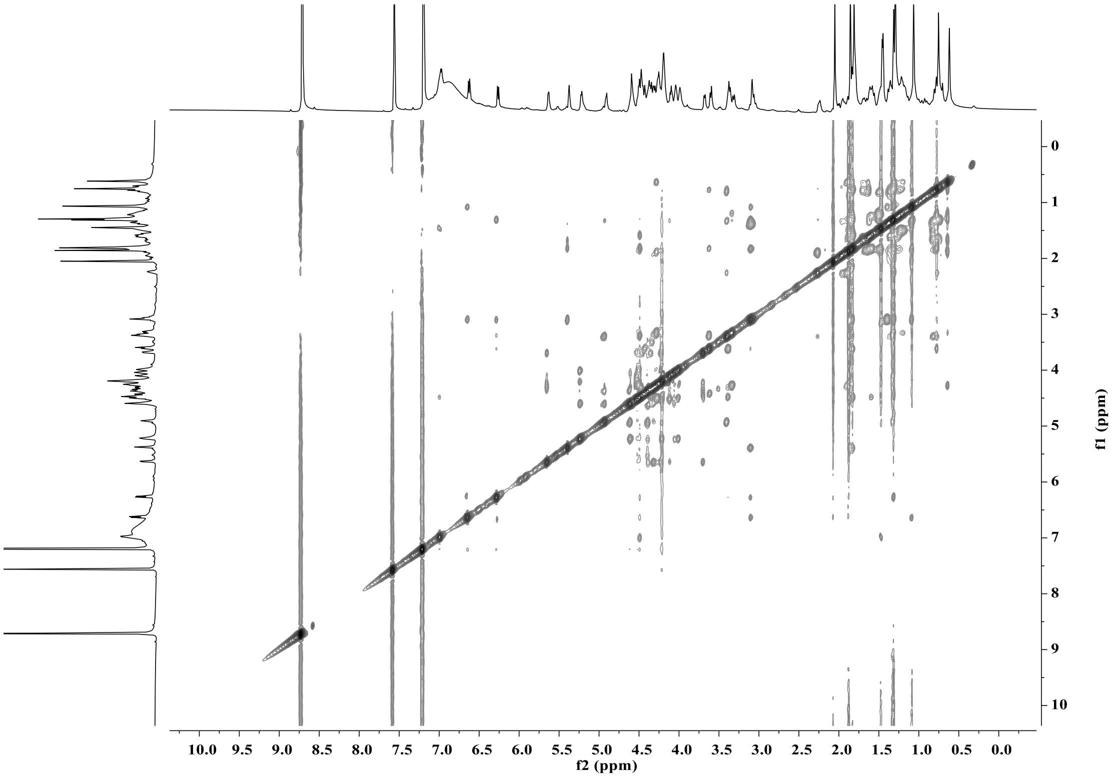

S41. NOESY Spectrum of the new compound **6**

Single Mass Analysis
Tolerance = 5.0 mDa / DBE: min = -1.5, max = 50.0
Element prediction: Off
Number of isotope peaks used for i-FIT = 3
Monoisotopic Mass, Even Electron Ions
562 formula(e) evaluated with 3 results within limits (up to 50 closest results for each mass)
Elements Used:

Mass	Calc. Mass	mDa	PPM	DBE	Formula	i-FIT	i-FIT Norm	Fit Conf %	C	H	O
1131.5586	1131.5587	-0.1	-0.1	12.5	C55 H87 O24	591.1	0.000	100.00	55	87	24
	1131.5564	2.2	1.9	43.5	C80 H75 O6	602.2	11.120	0.00	80	75	6
	1131.5622	-3.6	-3.2	34.5	C73 H79 O11	601.7	10.622	0.00	73	79	11

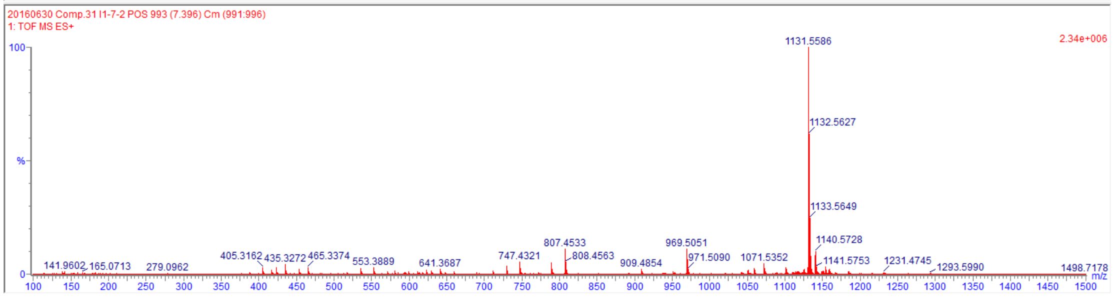

S42. HRESIMS spectrum of the new compound **6**

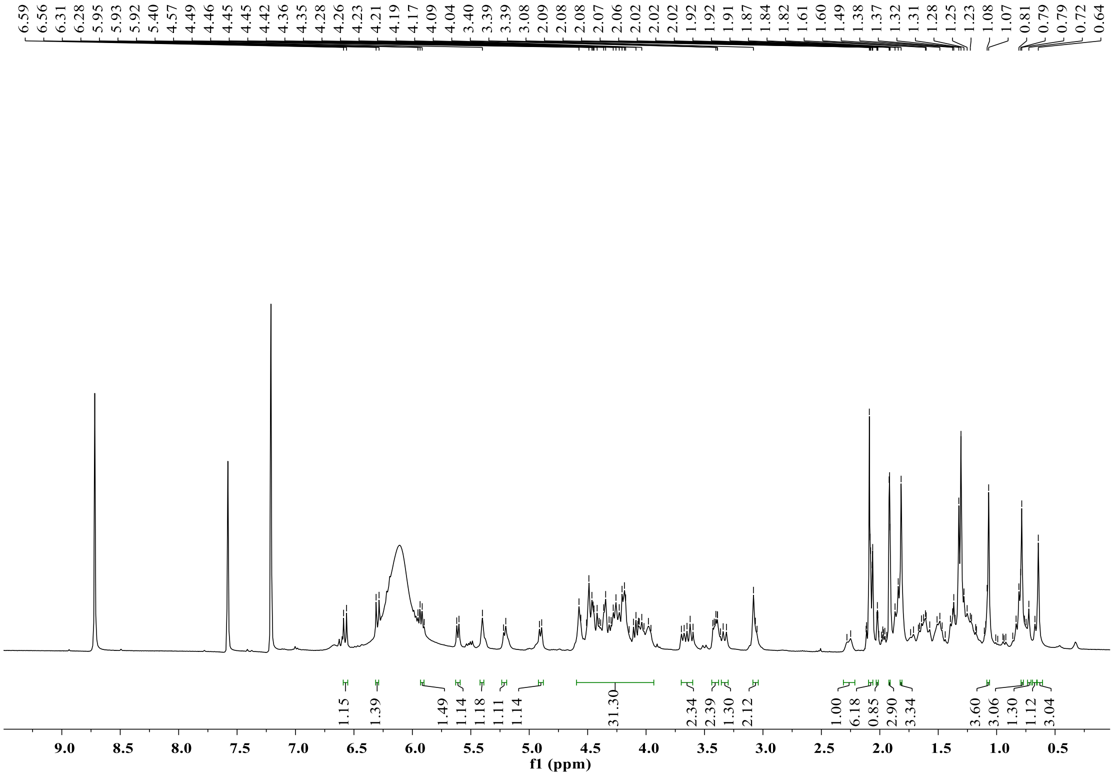

S43. ^{1}H NMR Spectrum (600 MHZ, Pyridine-d_5) of the new compound **7**

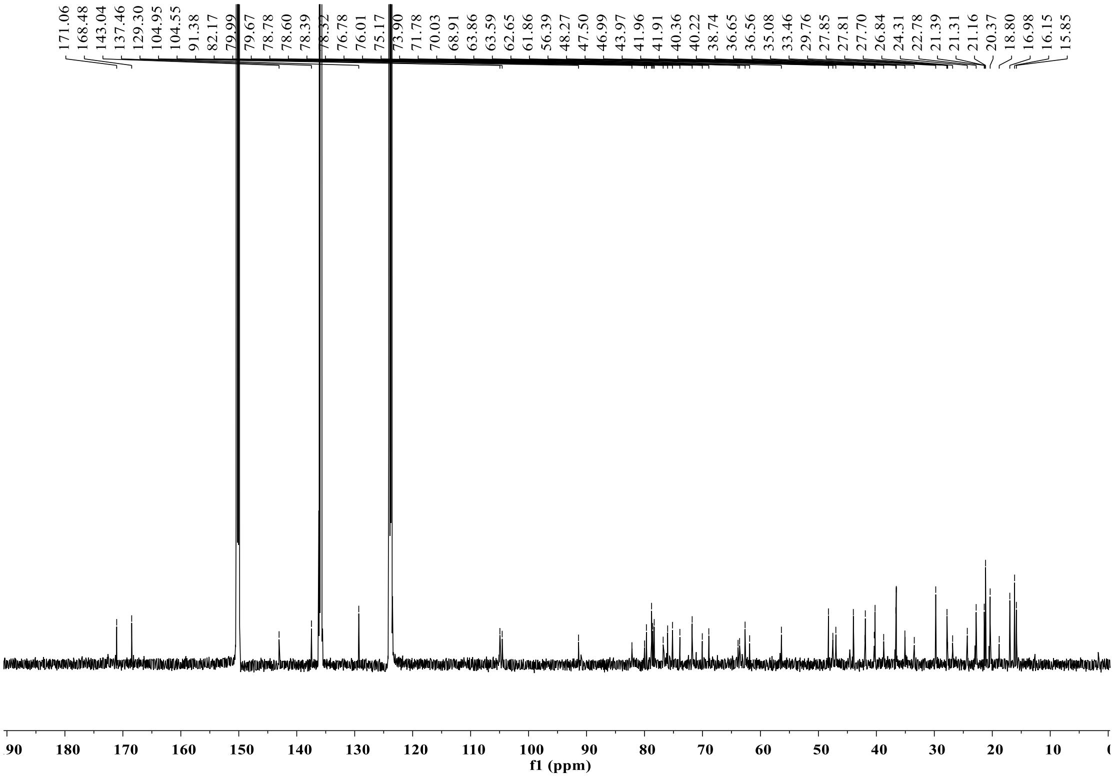

S44. ^{13}C NMR Spectrum (150 MHZ, Pyridine-d_5) of the new compound **7**

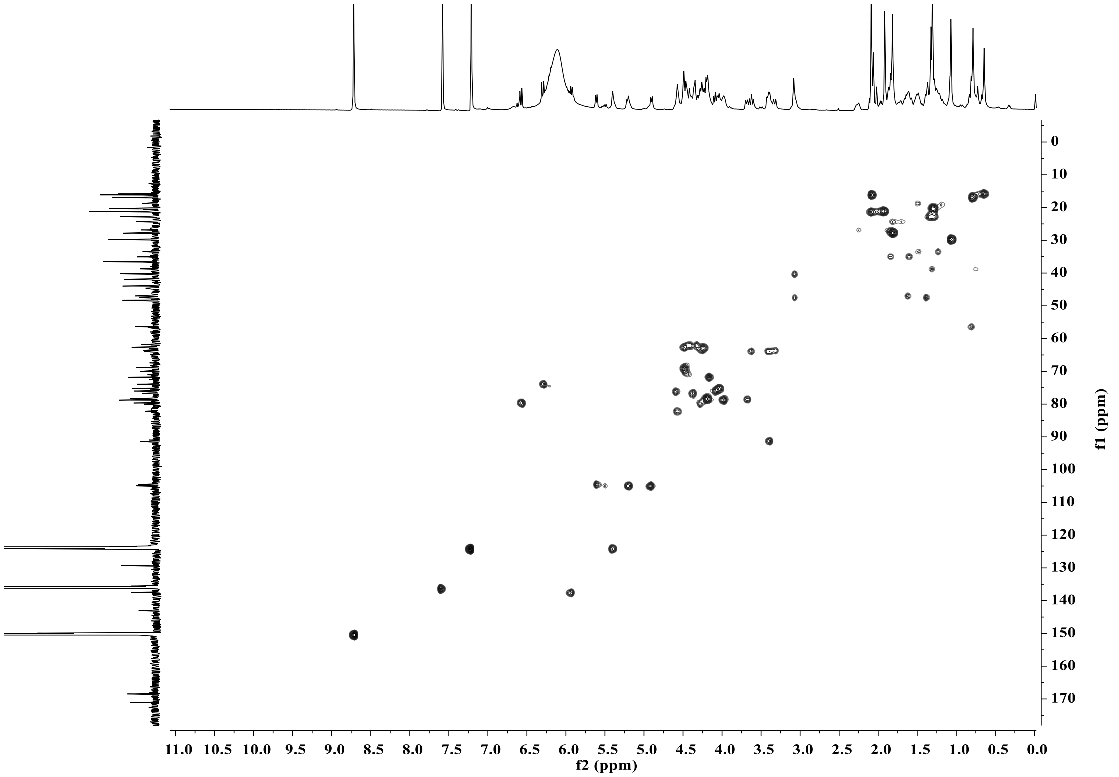

S45. HSQC Spectrum of the new compound **7**

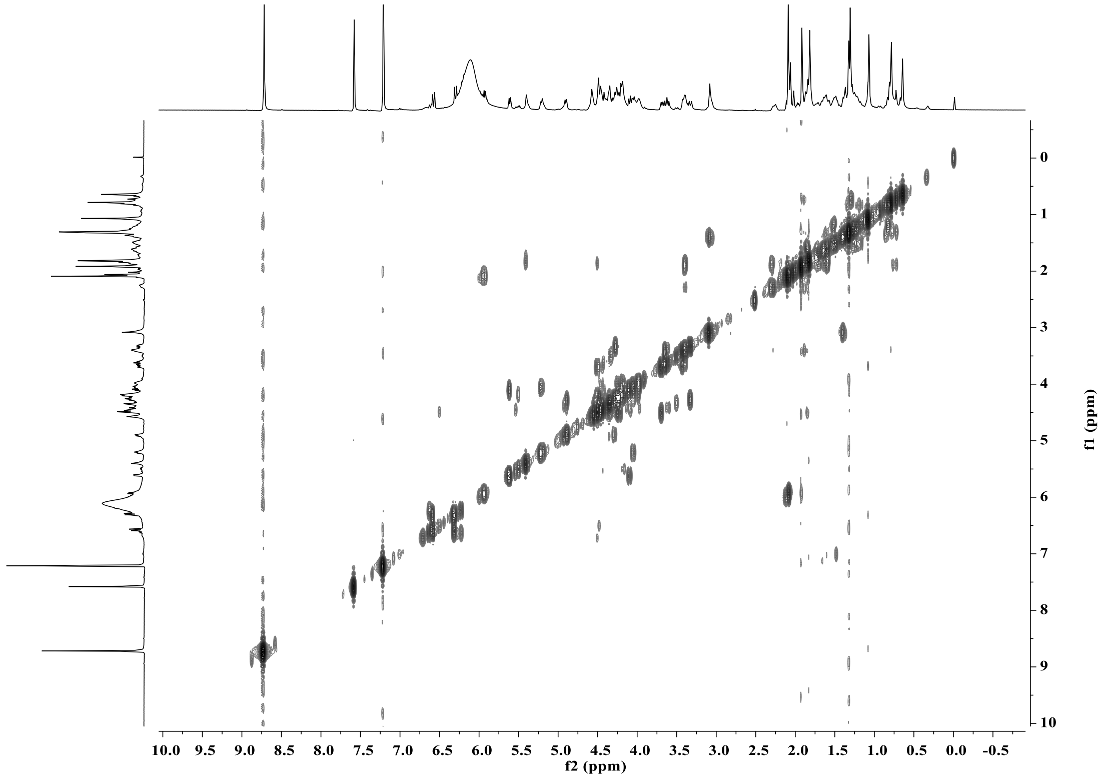

S46. ^1H-^1H COSY Spectrum of the new compound **7**

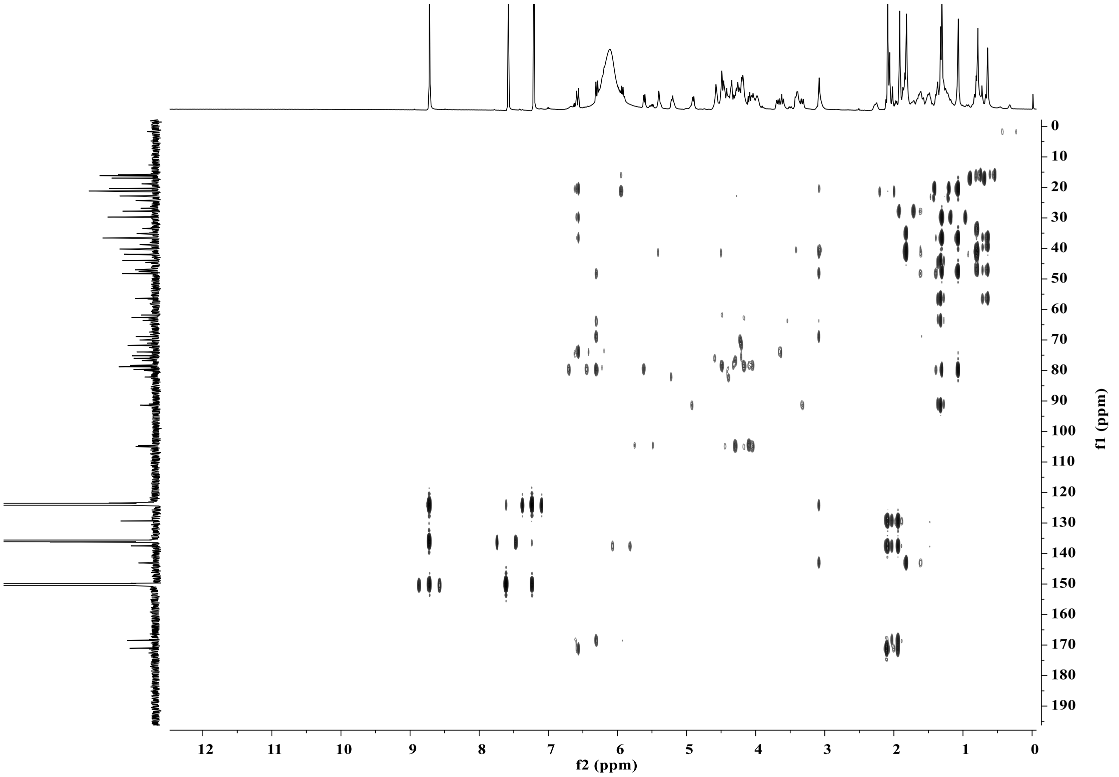

S47. HMBC Spectrum of the new compound **7**

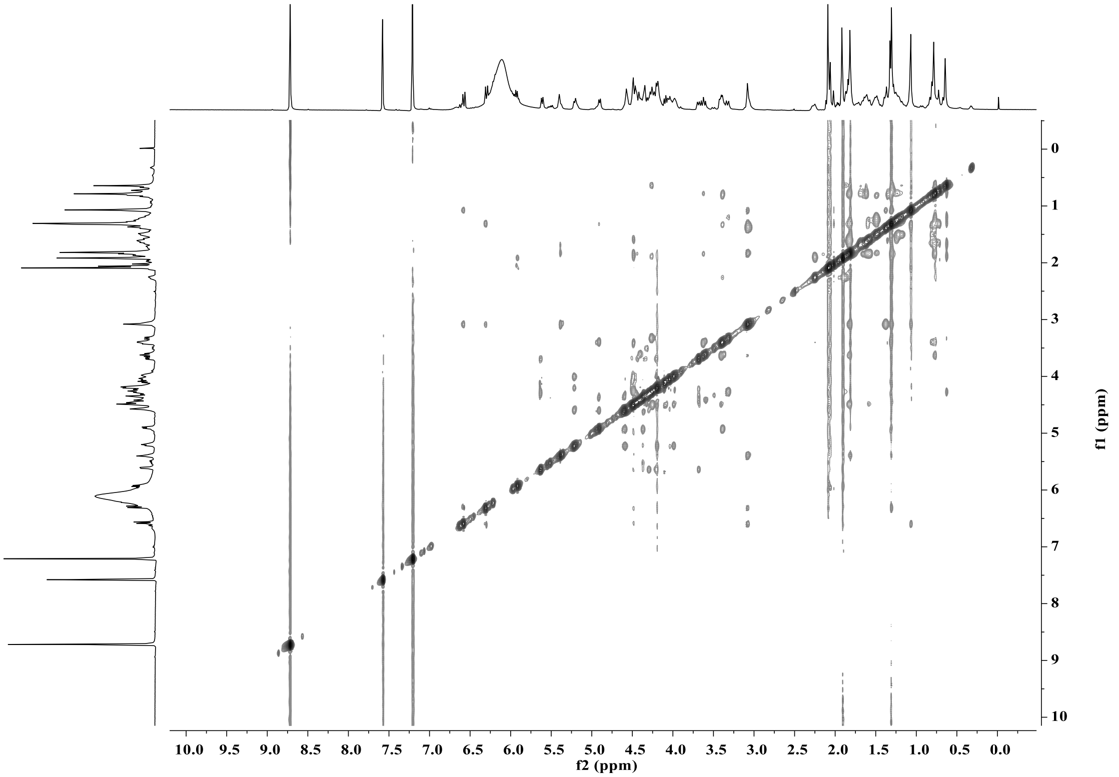

S48. NOESY Spectrum of the new compound **7**

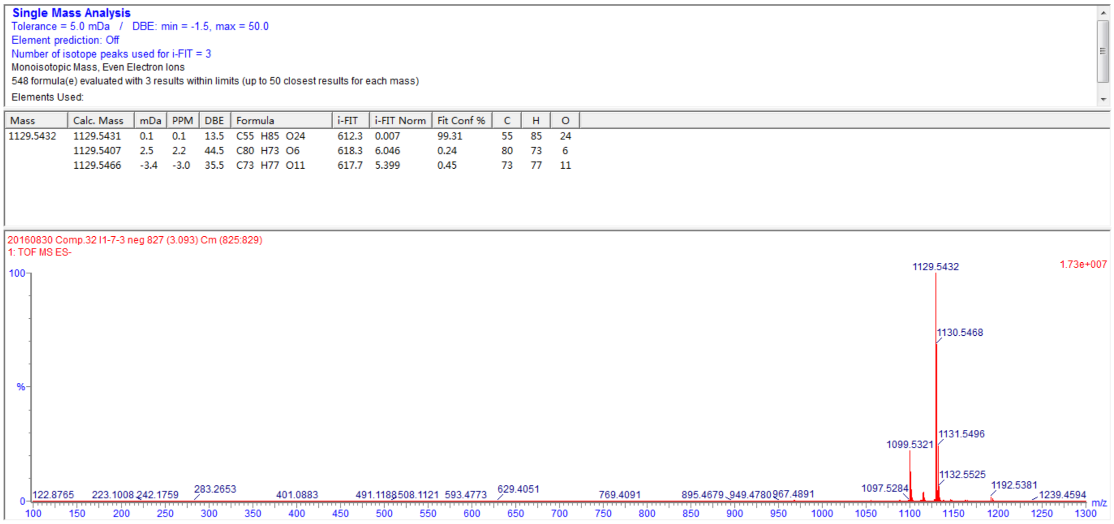

S49. HRESIMS spectrum of the new compound **7**

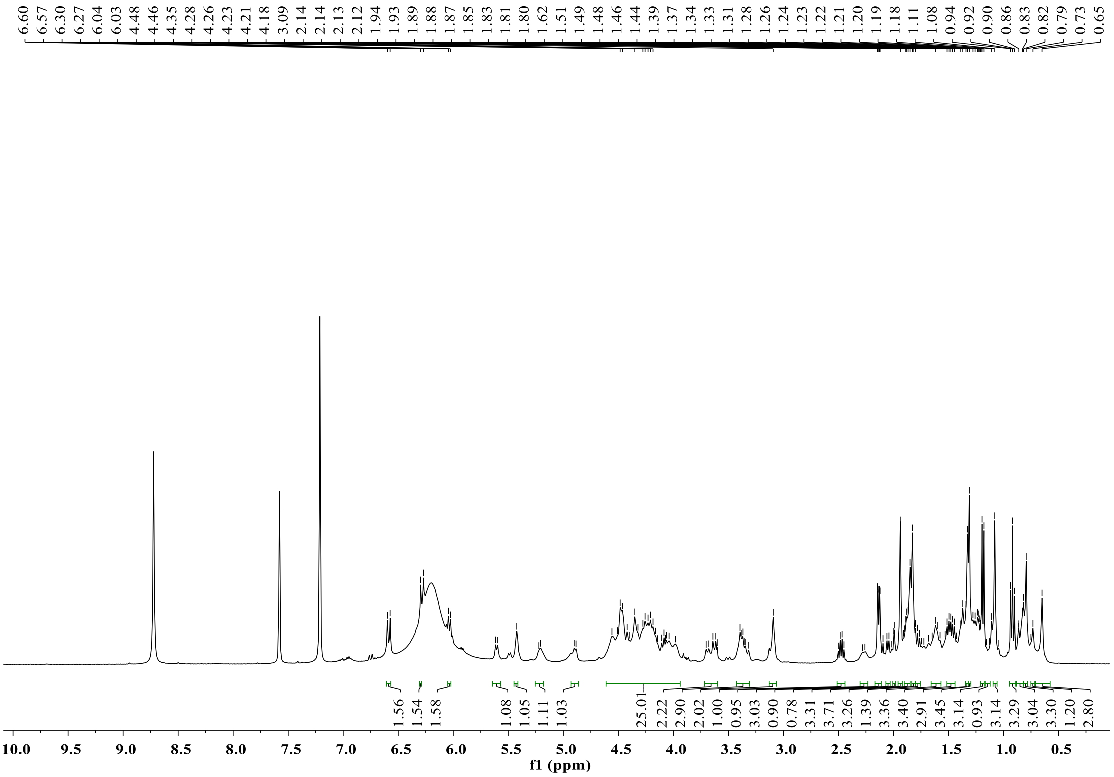

S50. ^{1}H NMR Spectrum (600 MHZ, Pyridine-d_5) of the new compound **8**

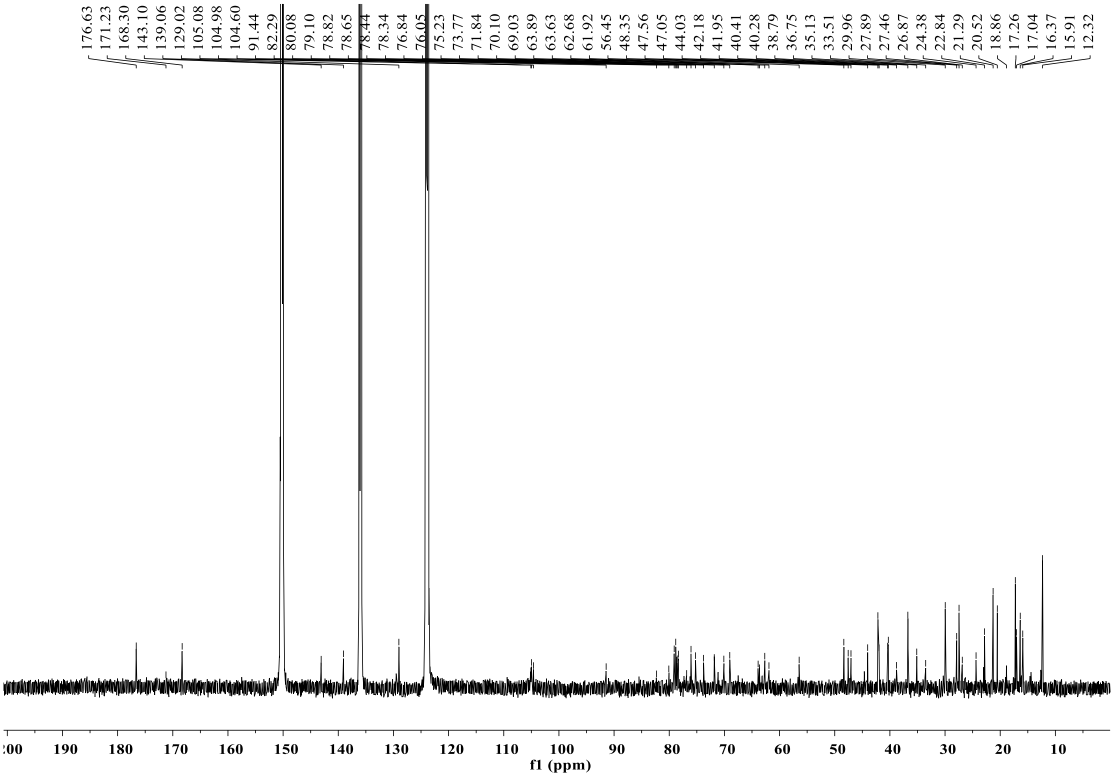

S51. ^{13}C NMR Spectrum (150 MHZ, Pyridine-d_5) of the new compound **8**

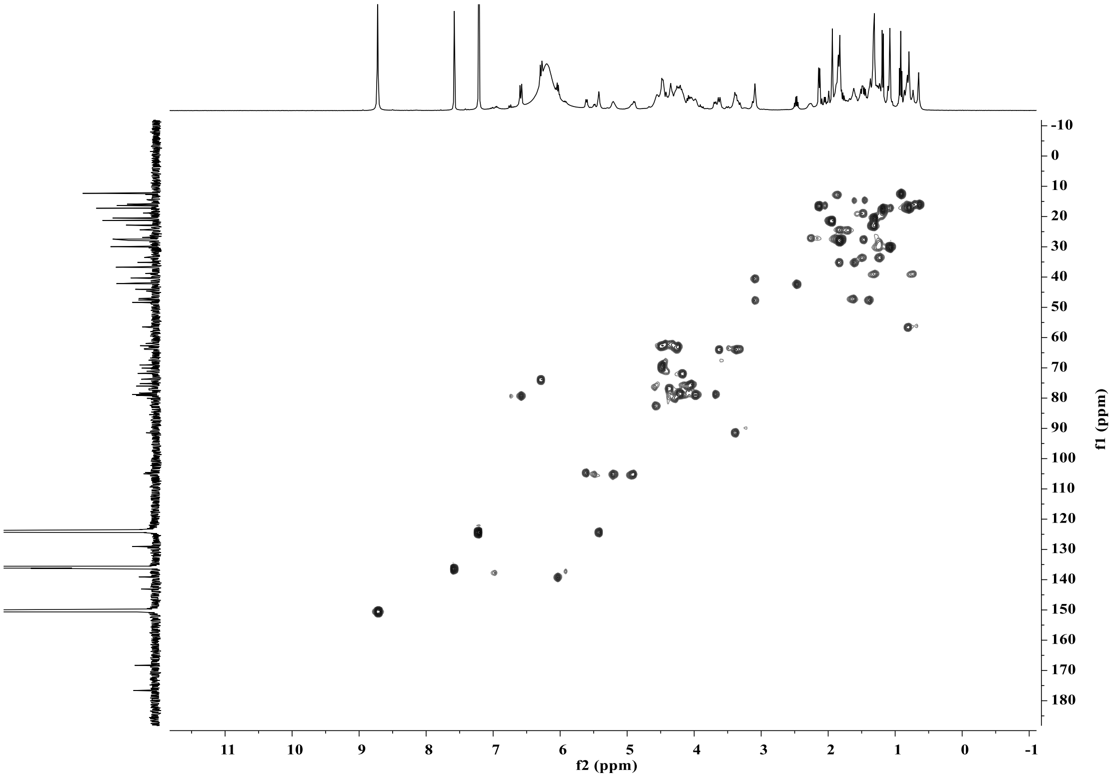

S52. HSQC Spectrum of the new compound **8**

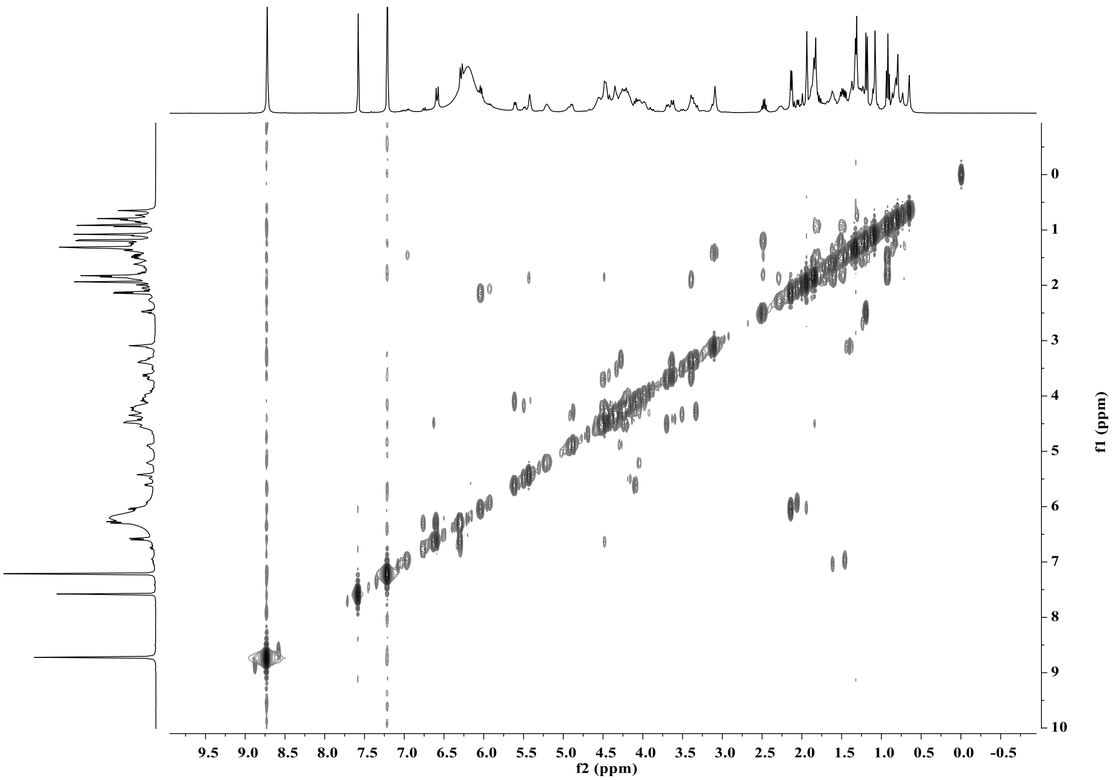

S53. ^{1}H-^{1}H COSY Spectrum of the new compound **8**

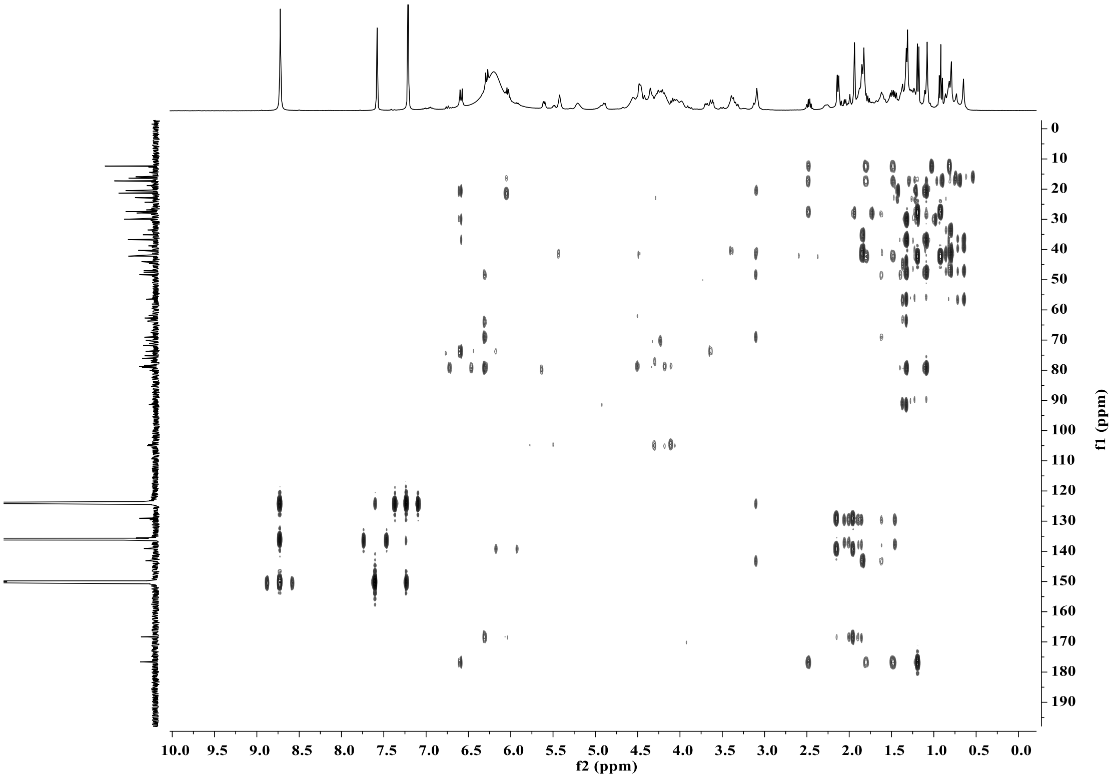

S54. HMBC Spectrum of the new compound **8**

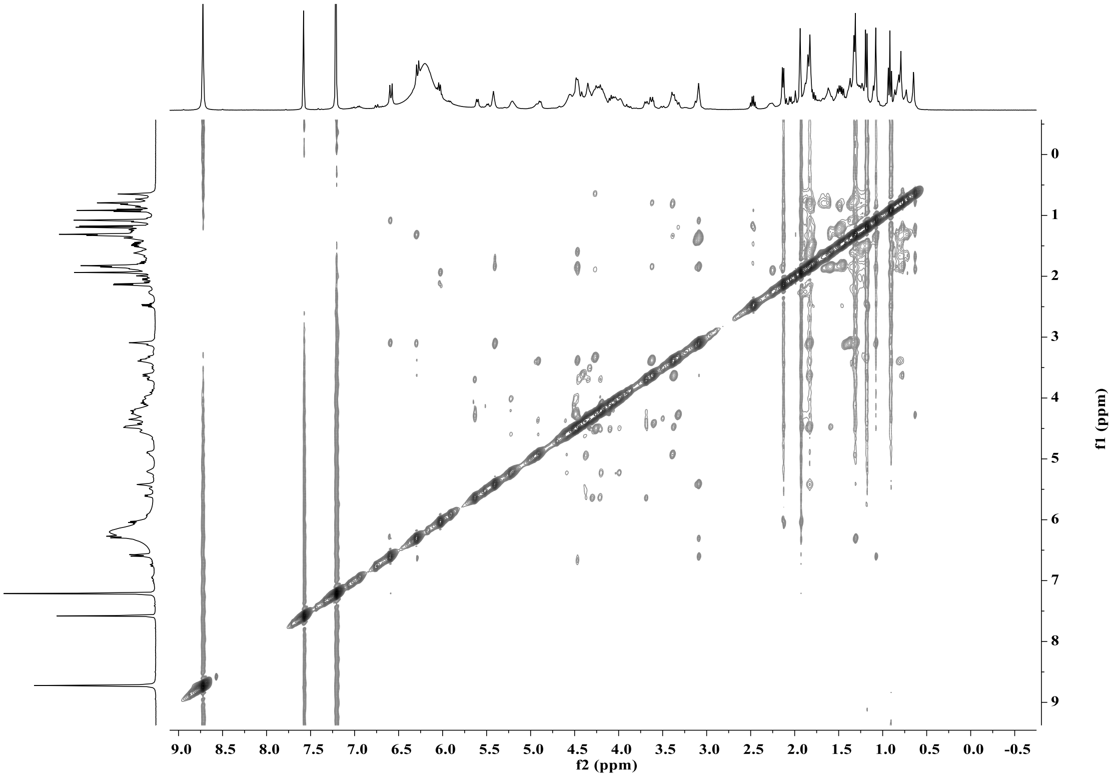

S55. NOESY Spectrum of the new compound **8**

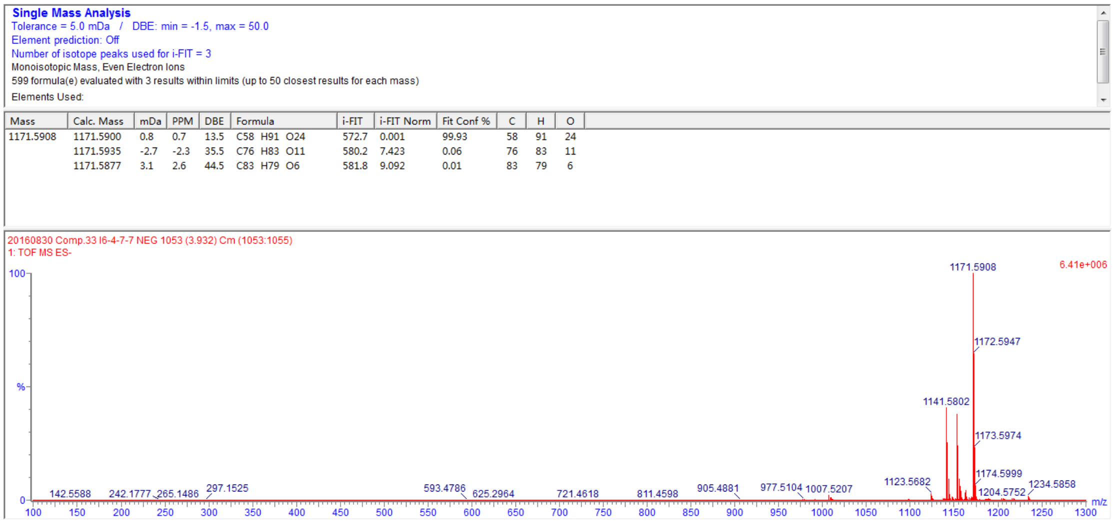

Single Mass Analysis
Tolerance = 5.0 mDa / DBE: min = -1.5, max = 50.0
Element prediction: Off
Number of isotope peaks used for i-FIT = 3
Monoisotopic Mass, Even Electron Ions
599 formula(e) evaluated with 3 results within limits (up to 50 closest results for each mass)
Elements Used:

Mass	Calc. Mass	mDa	PPM	DBE	Formula	i-FIT	i-FIT Norm	Fit Conf %	C	H	O
1171.5908	1171.5900	0.8	0.7	13.5	C58 H91 O24	572.7	0.001	99.93	58	91	24
	1171.5935	-2.7	-2.3	35.5	C76 H83 O11	580.2	7.423	0.06	76	83	11
	1171.5877	3.1	2.6	44.5	C83 H79 O6	581.8	9.092	0.01	83	79	6

S56. HRESIMS spectrum of the new compound **8**

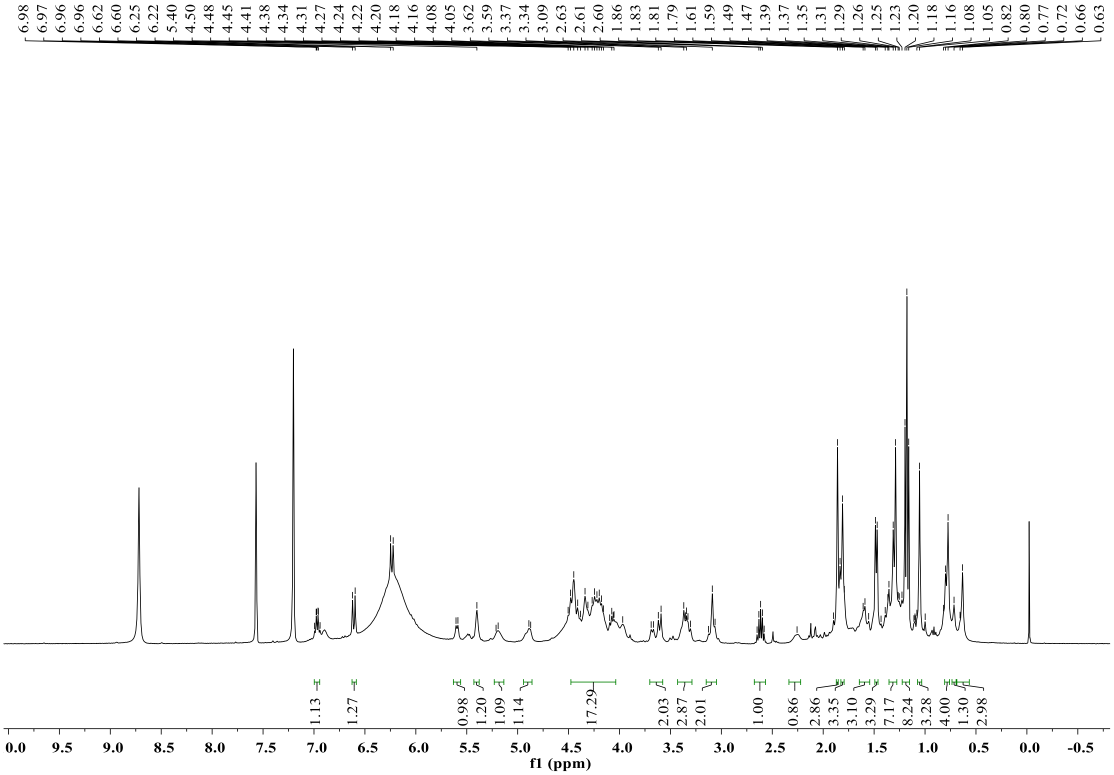

S57. ^{1}H NMR Spectrum (600 MHZ, Pyridine-d_5) of the new compound **9**

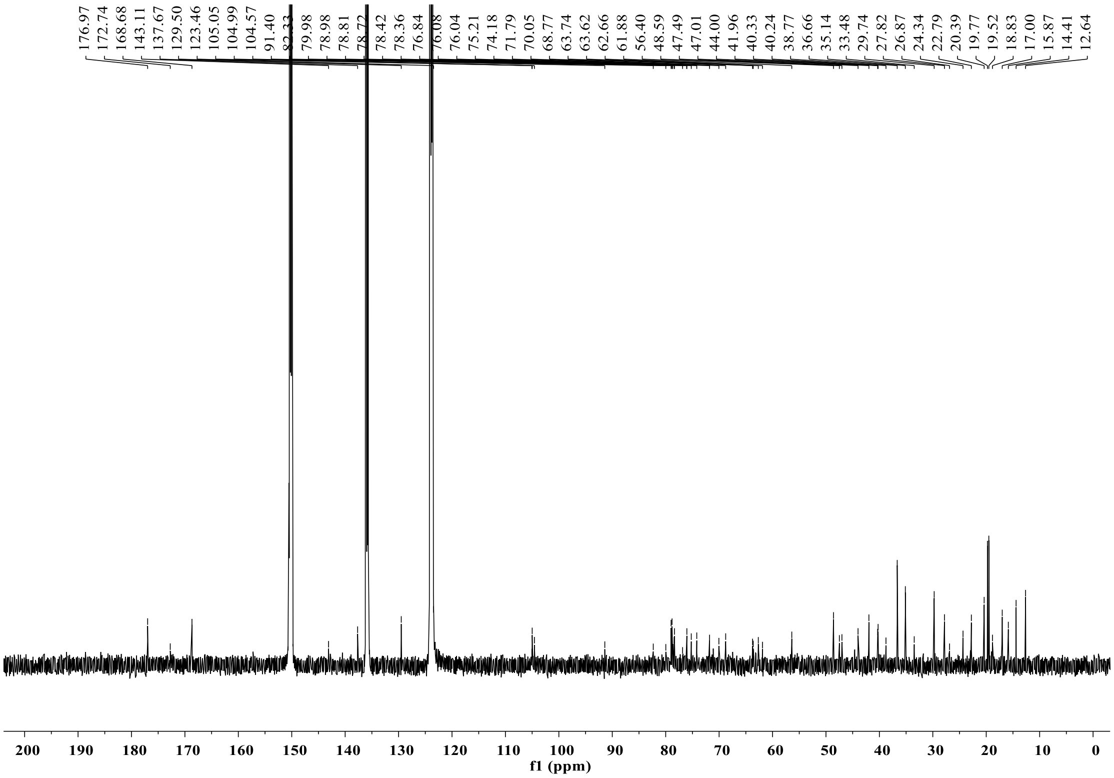

S58. ^{13}C NMR Spectrum (150 MHZ, Pyridine-d_5) of the new compound **9**

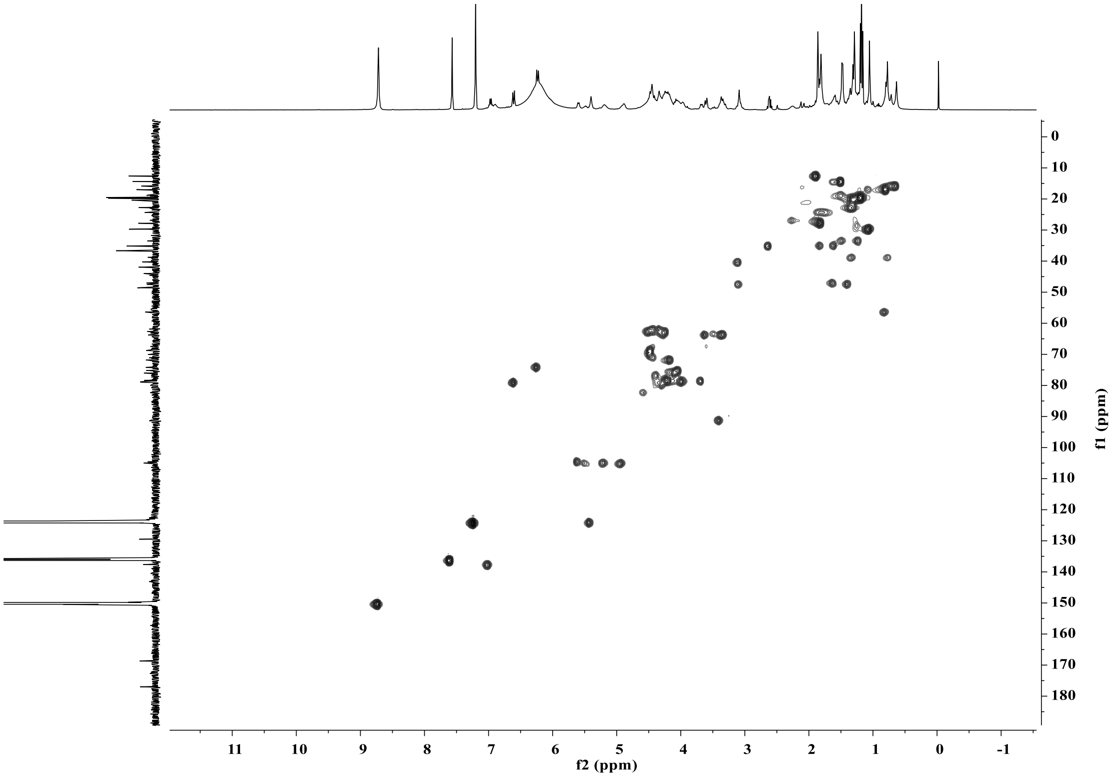

S59. HSQC Spectrum of the new compound **9**

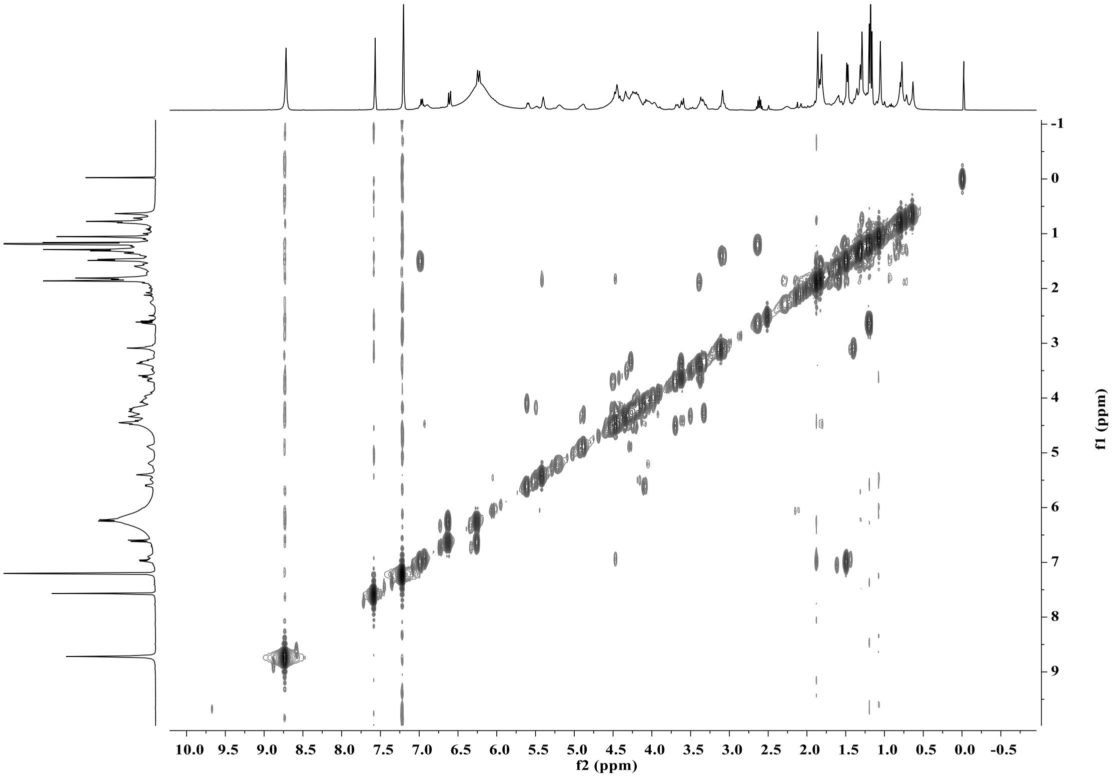

S60. ^{1}H-^{1}H COSY Spectrum of the new compound **9**

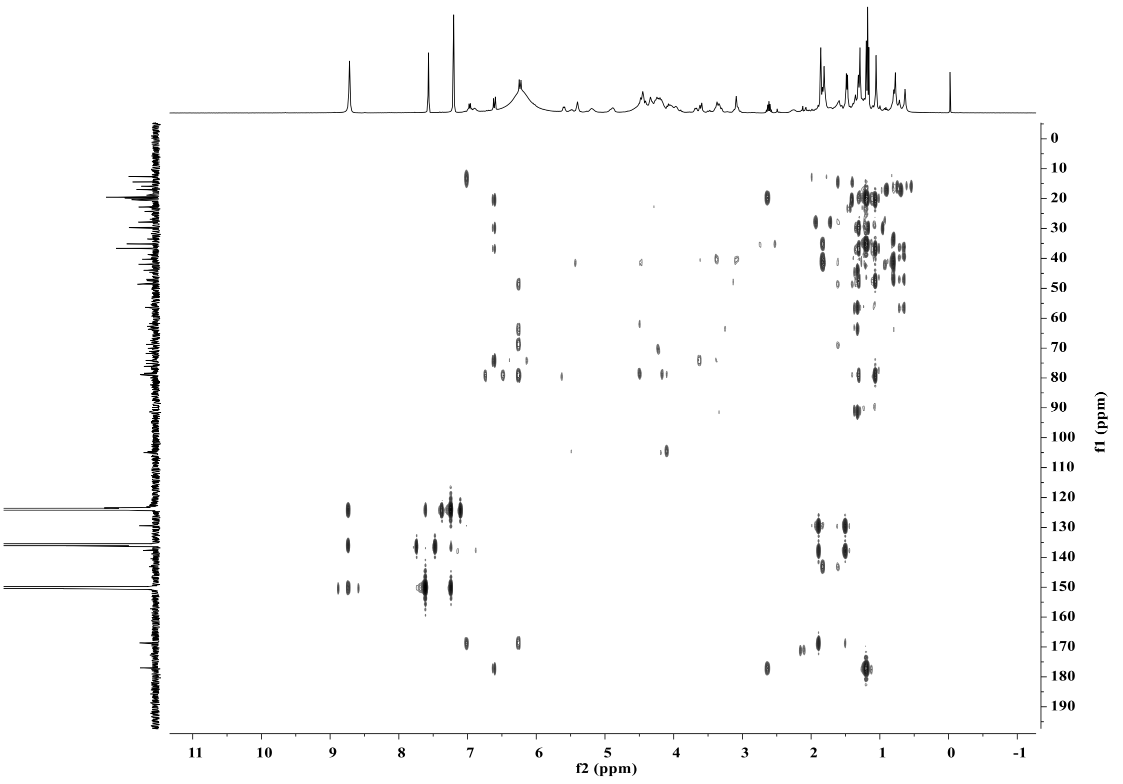

S61. HMBC Spectrum of the new compound **9**

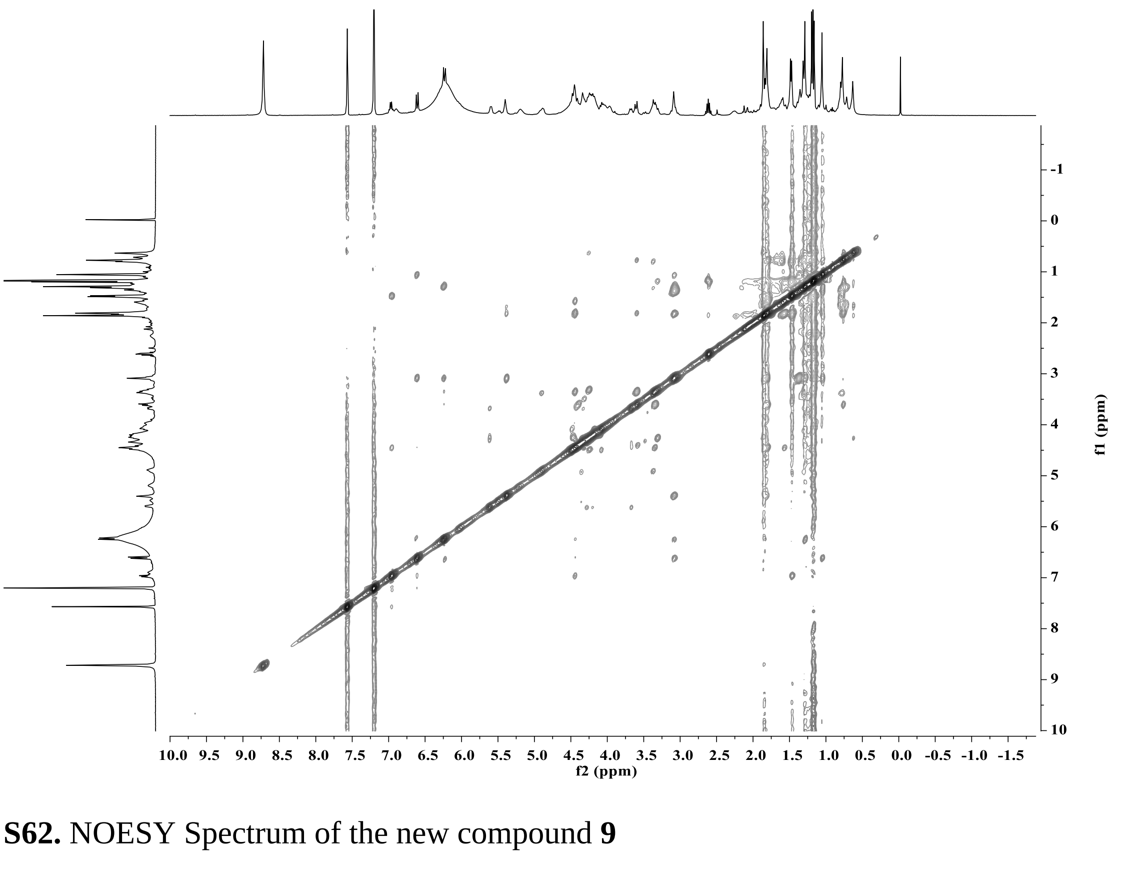

S62. NOESY Spectrum of the new compound **9**

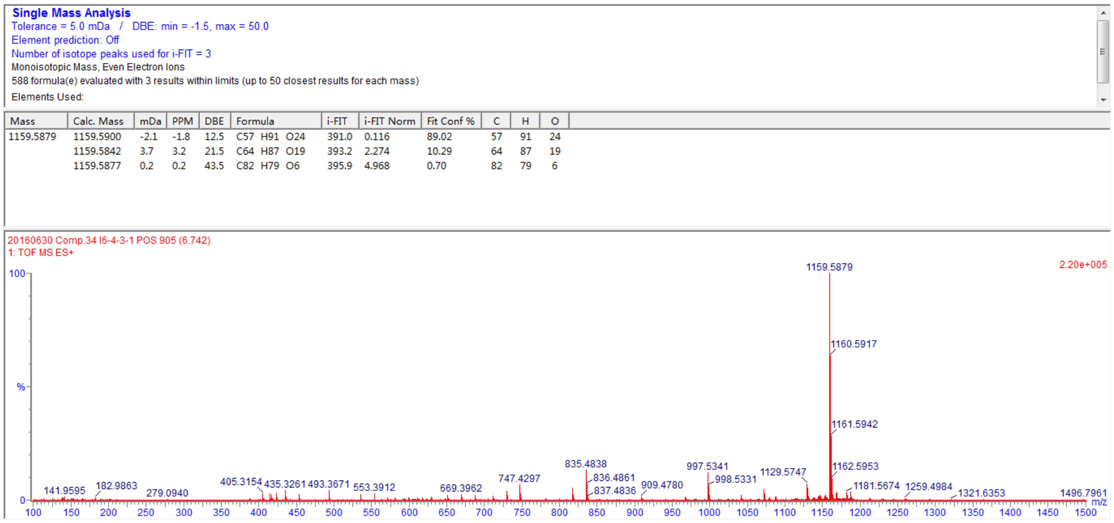

S63. HRESIMS spectrum of the new compound **9**

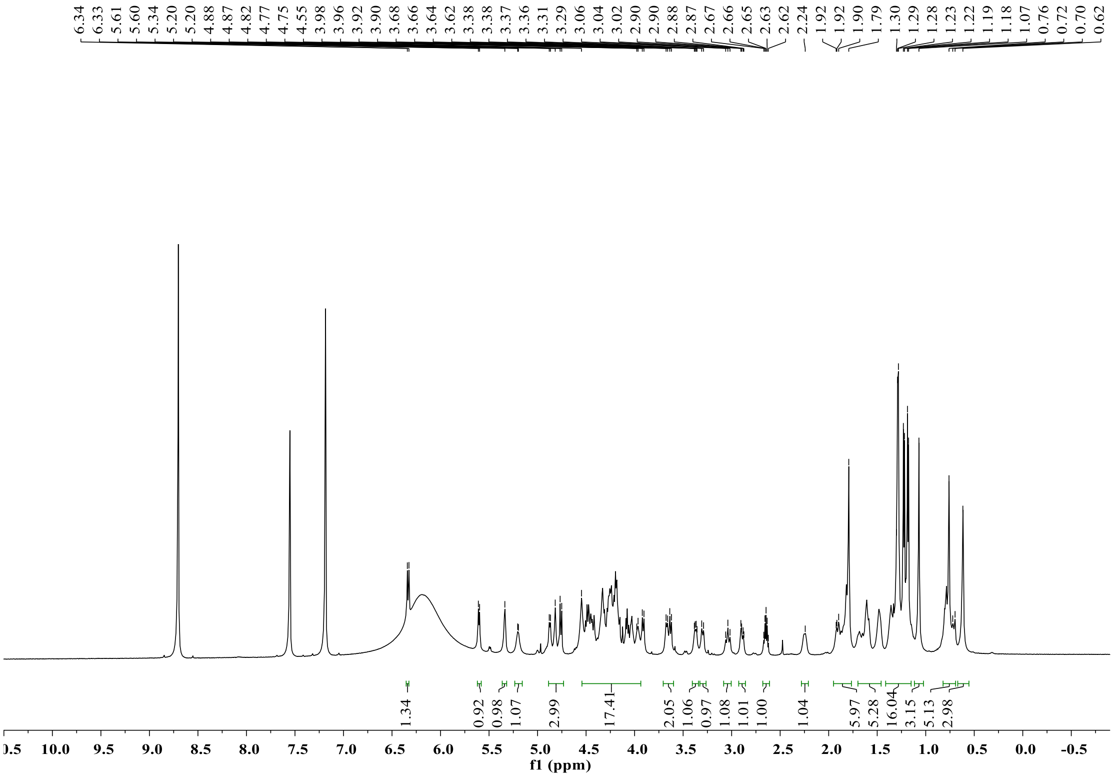

S64. ^{1}H NMR Spectrum (600 MHZ, Pyridine-d_5) of the new compound **10**

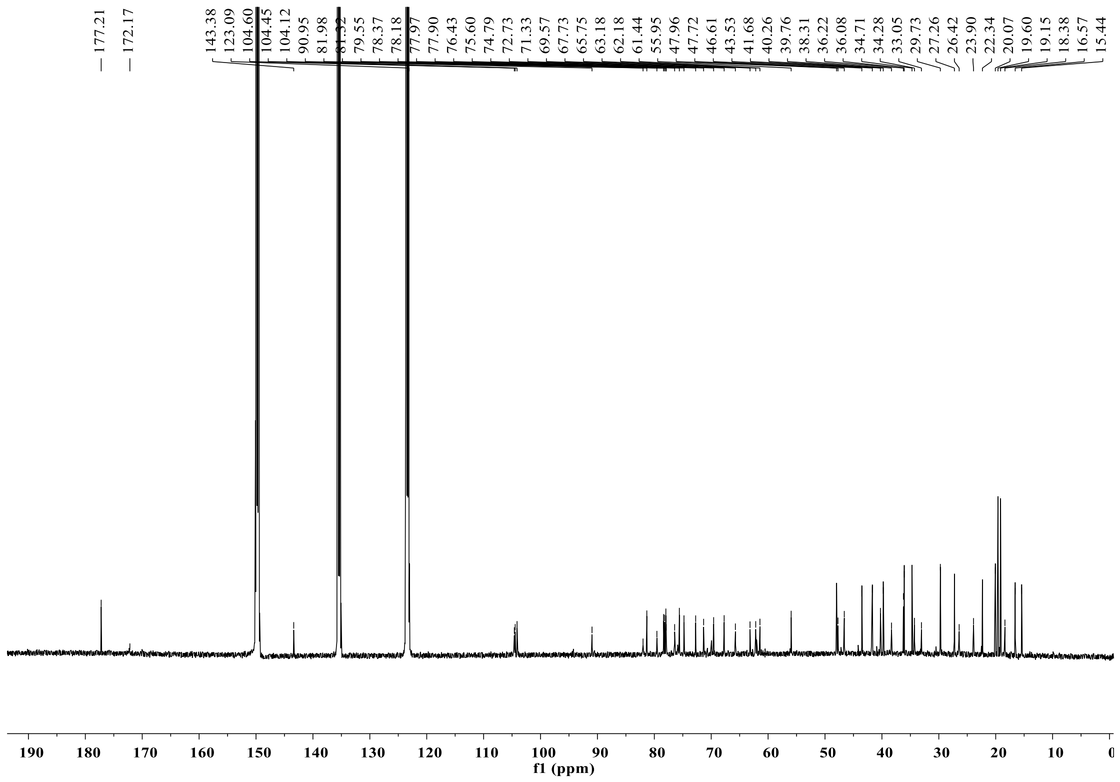

S65. ^{13}C NMR Spectrum (150 MHZ, Pyridine-d_5) of the new compound **10**

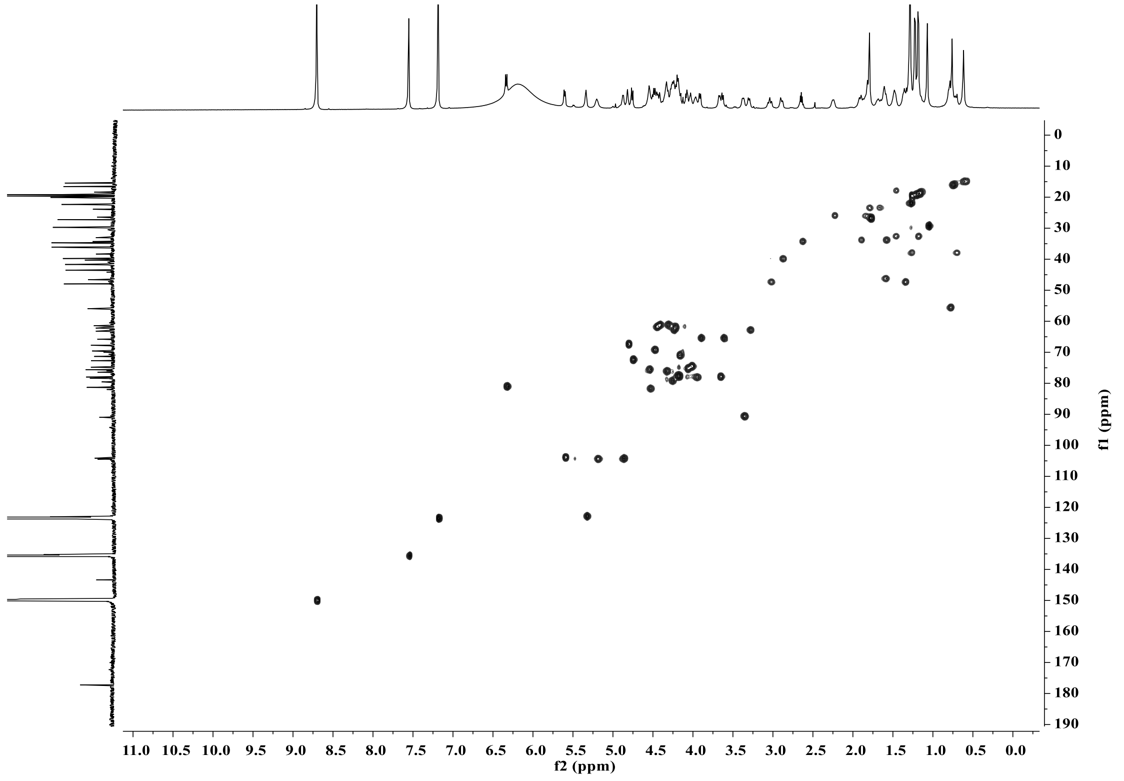

S66. HSQC Spectrum of the new compound **10**

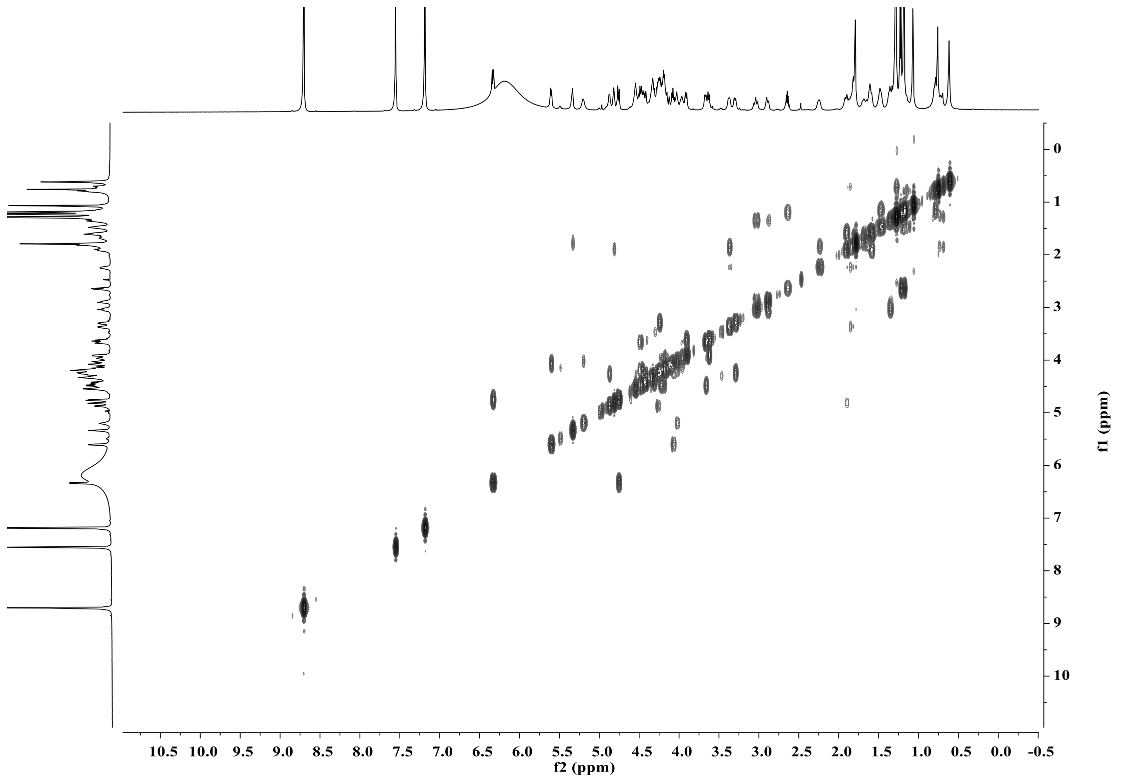

S67. ^{1}H-^{1}H COSY Spectrum of the new compound **10**

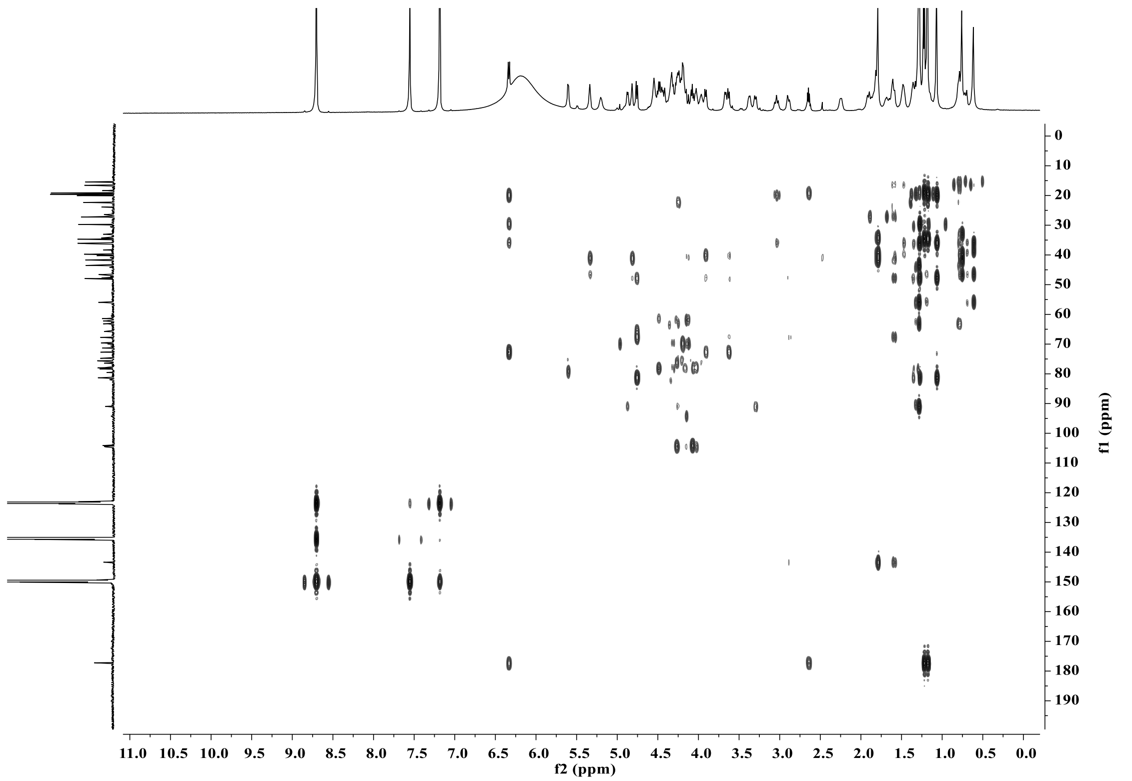

S68. HMBC Spectrum of the new compound **10**

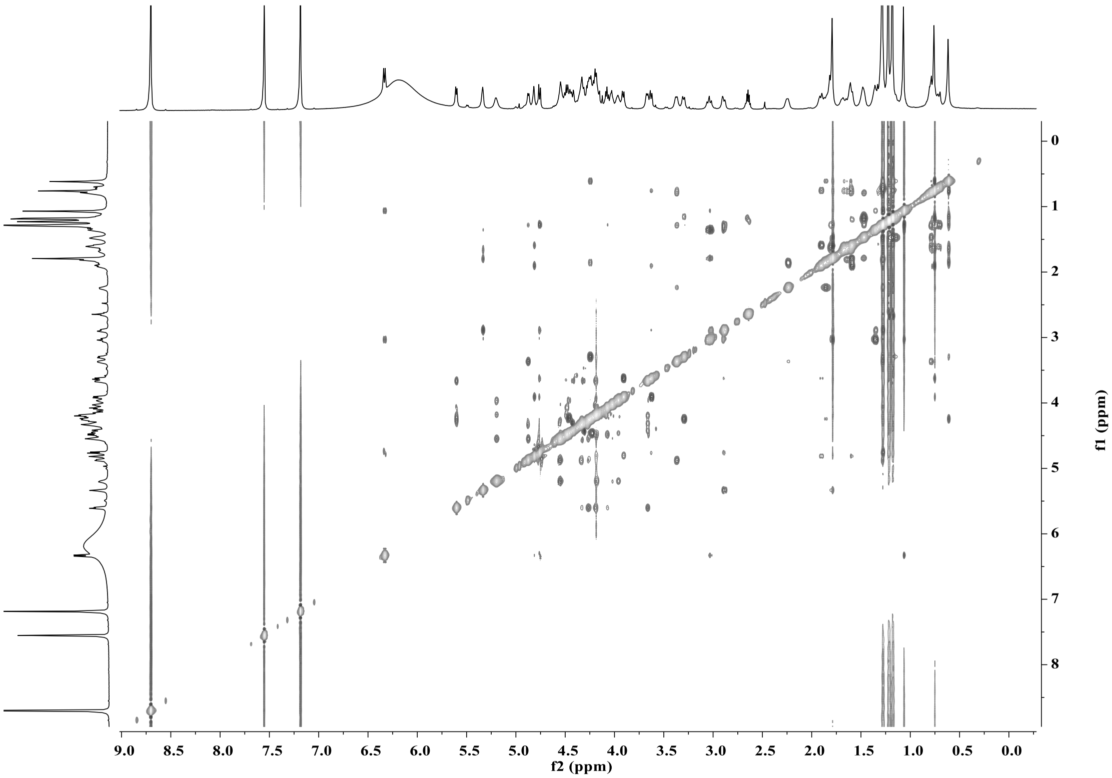

S69. NOESY Spectrum of the new compound **10**

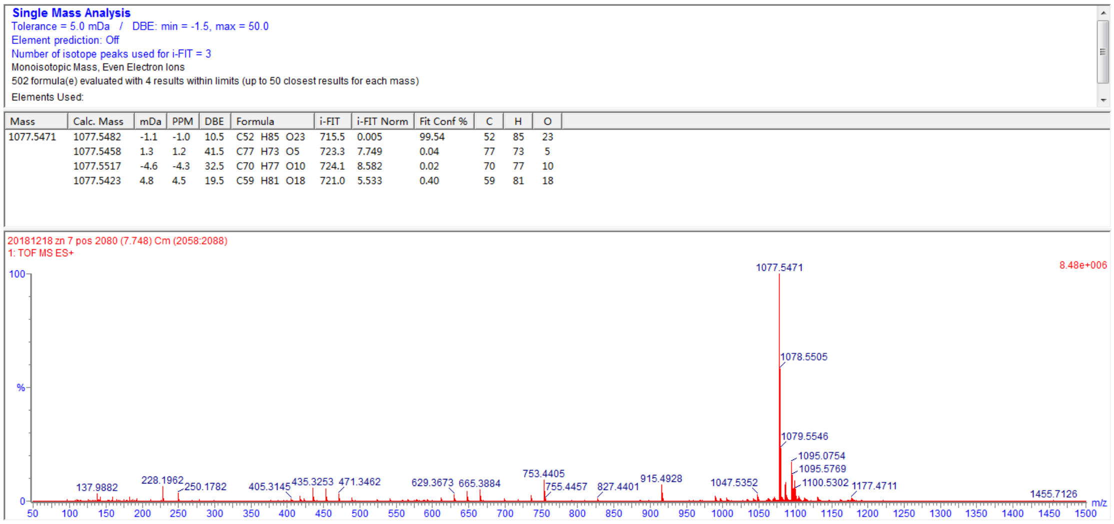

Mass	Calc. Mass	mDa	PPM	DBE	Formula	i-FIT	i-FIT Norm	Fit Conf %	C	H	O
1077.5471	1077.5482	-1.1	-1.0	10.5	C52 H85 O23	715.5	0.005	99.54	52	85	23
	1077.5458	1.3	1.2	41.5	C77 H73 O5	723.3	7.749	0.04	77	73	5
	1077.5517	-4.6	-4.3	32.5	C70 H77 O10	724.1	8.582	0.02	70	77	10
	1077.5423	4.8	4.5	19.5	C59 H81 O18	721.0	5.533	0.40	59	81	18

S70. HRESIMS spectrum of the new compound **10**

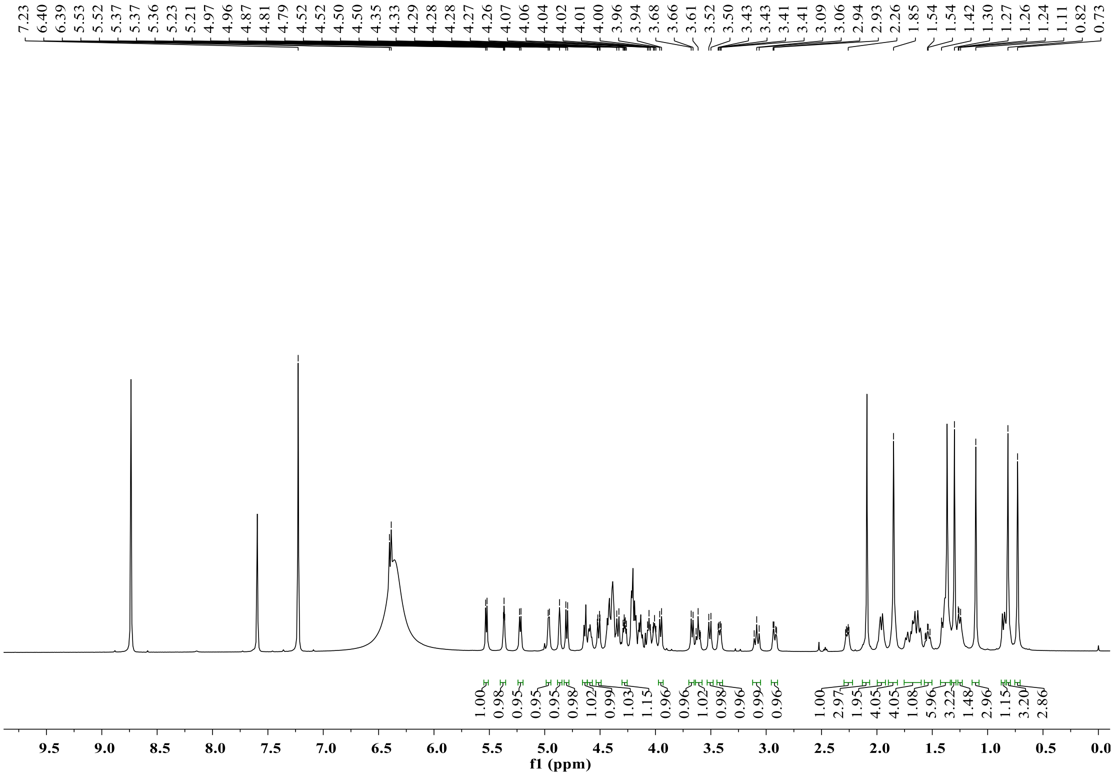

S71. ^{1}H NMR Spectrum (600 MHZ, Pyridine-d_5) of the new compound **11**

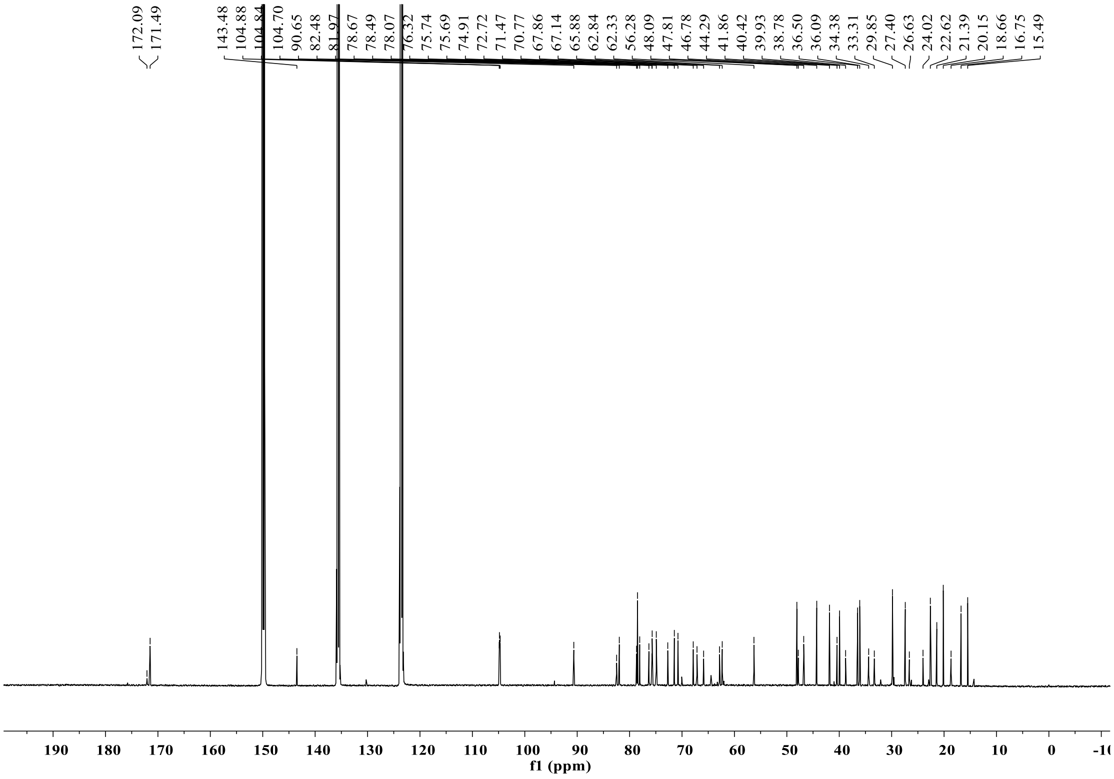

S72. ^{13}C NMR Spectrum (150 MHZ, Pyridine-d_5) of the new compound **11**

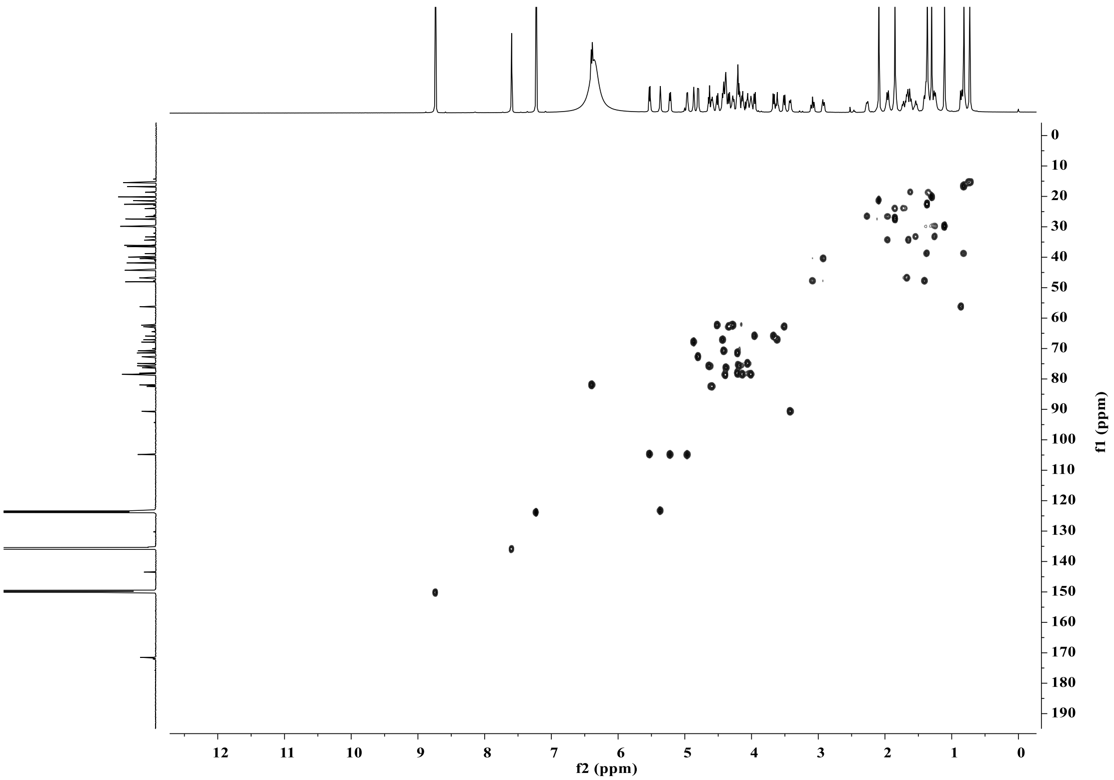

S73. HSQC Spectrum of the new compound **11**

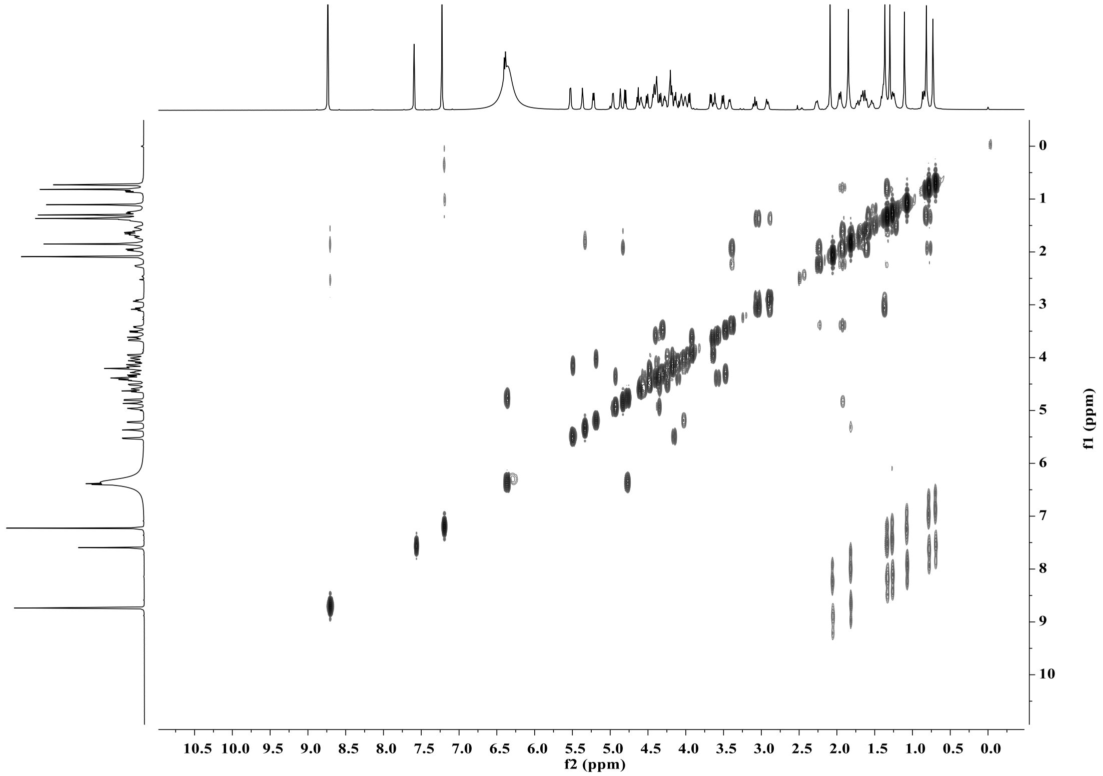

S74. ^{1}H-^{1}H COSY Spectrum of the new compound **11**

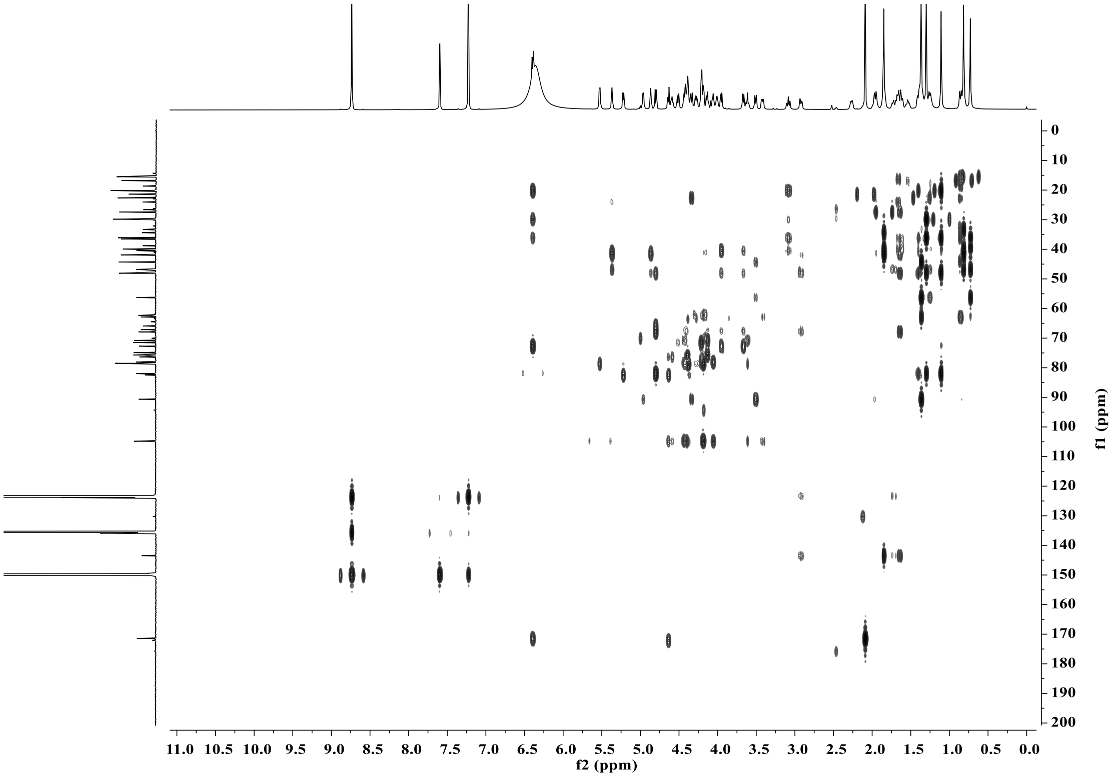

S75. HMBC Spectrum of the new compound **11**

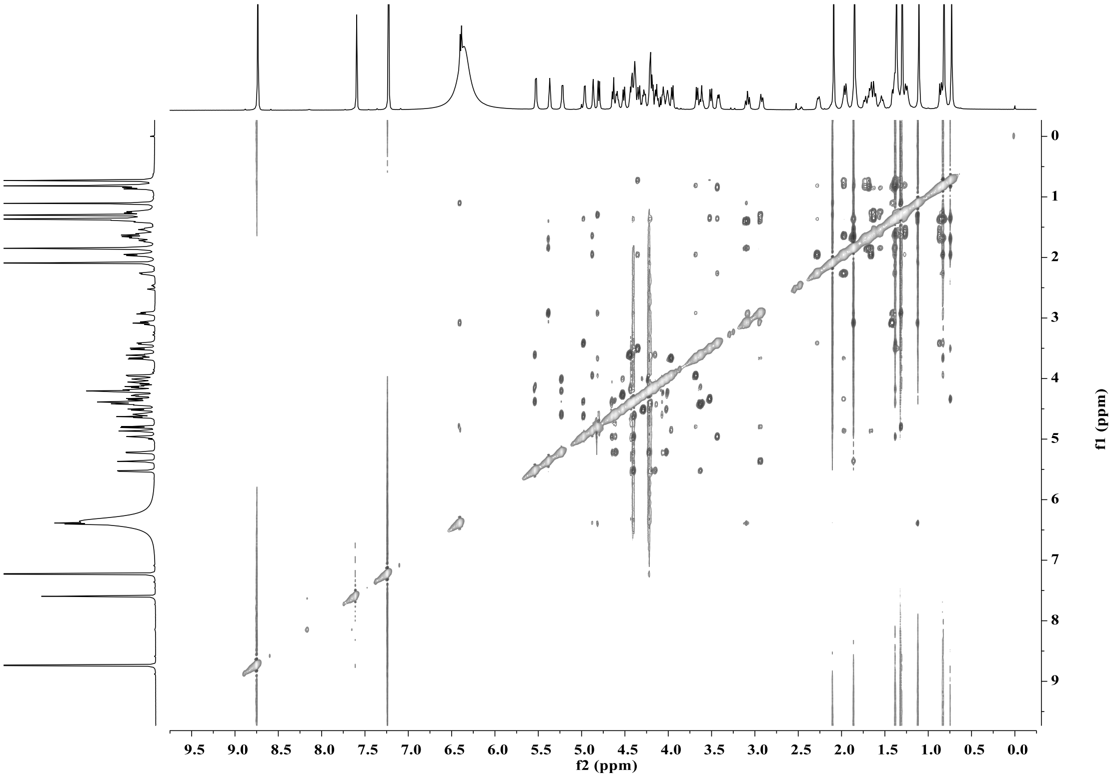

S76. NOESY Spectrum of the new compound **11**

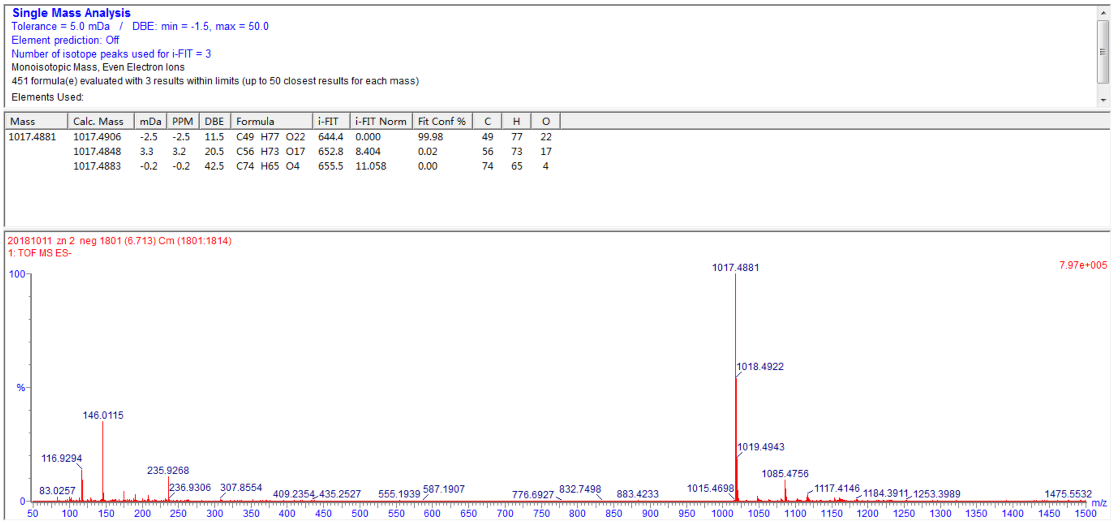

S77. HRESIMS spectrum of the new compound **11**

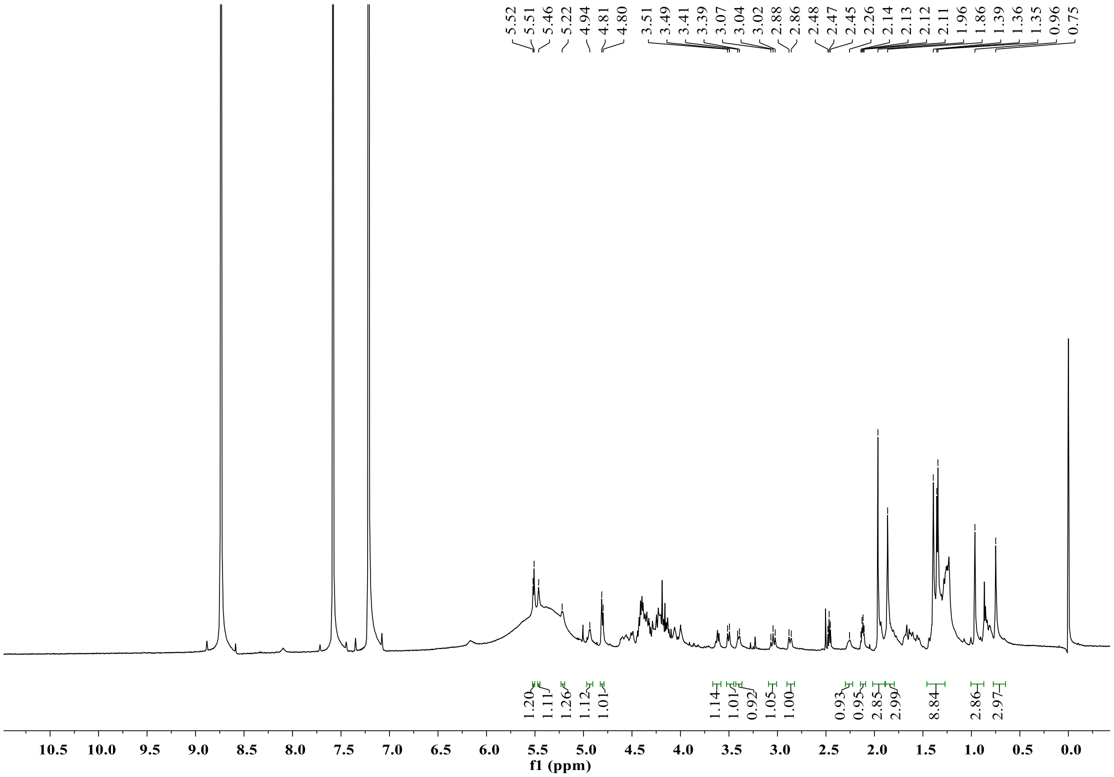

S78. ^{1}H NMR Spectrum (600 MHZ, Pyridine-d_5) of the new compound **12**

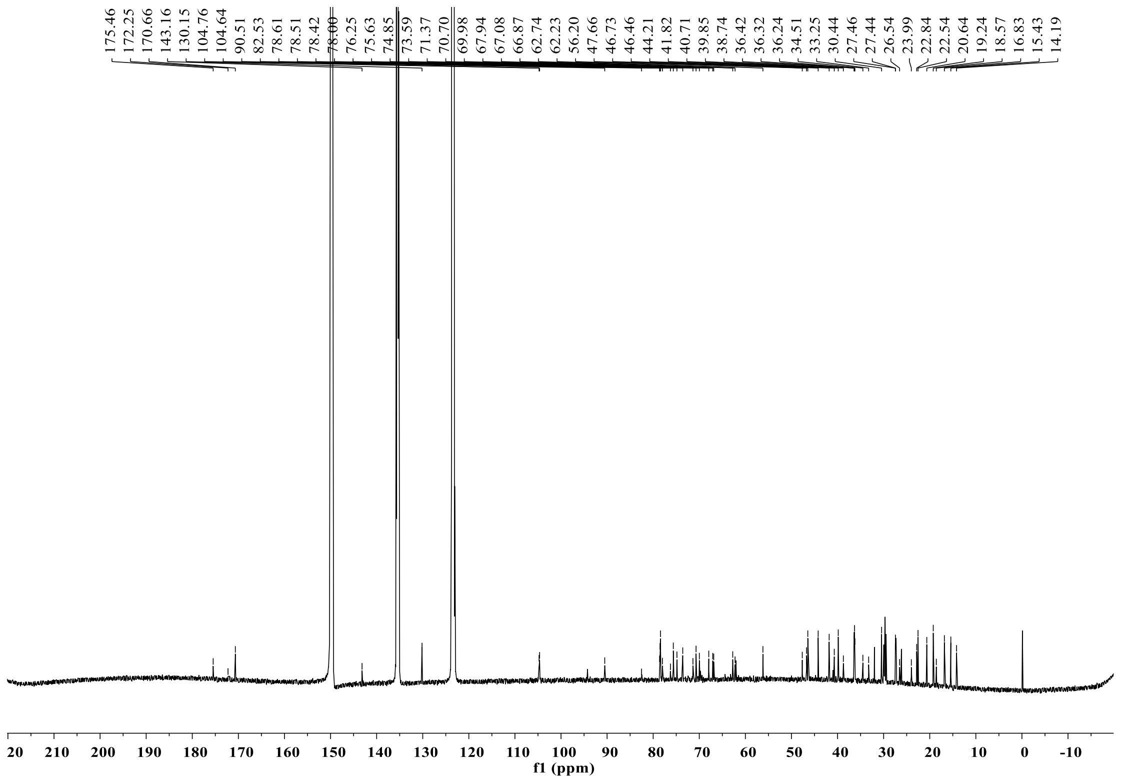

S79. ^{13}C NMR Spectrum (150 MHZ, Pyridine-d_5) of the new compound **12**

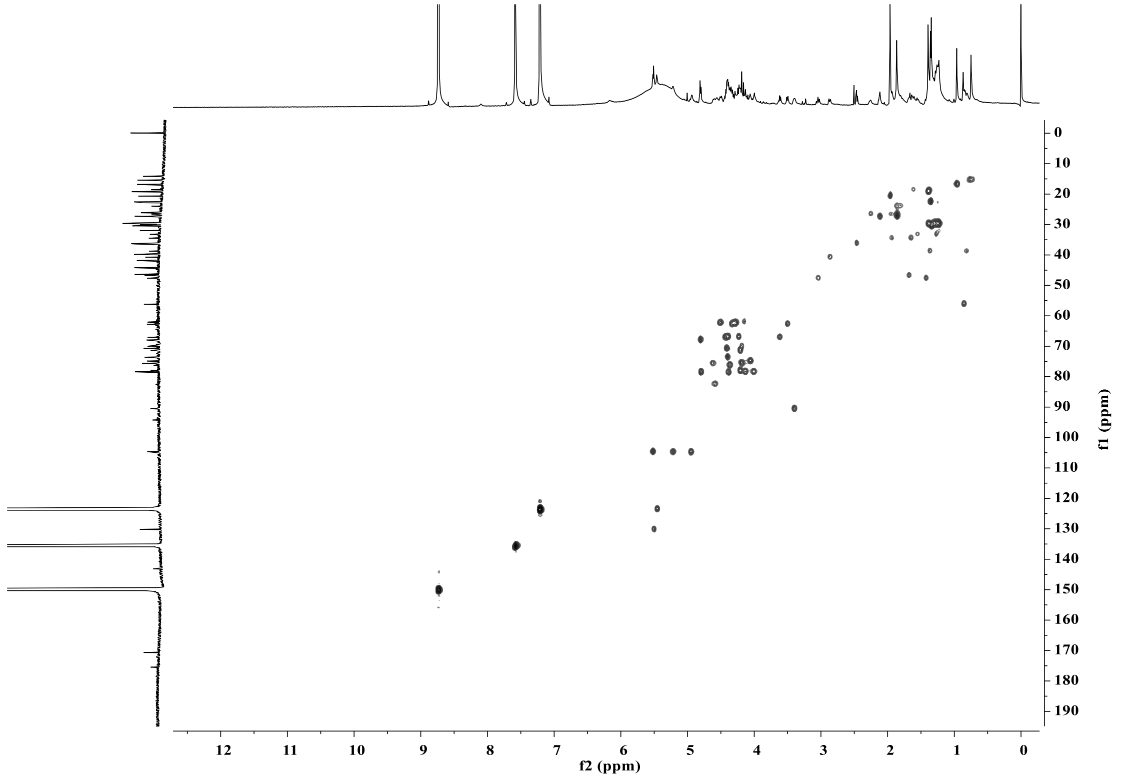

S80. HSQC Spectrum of the new compound **12**

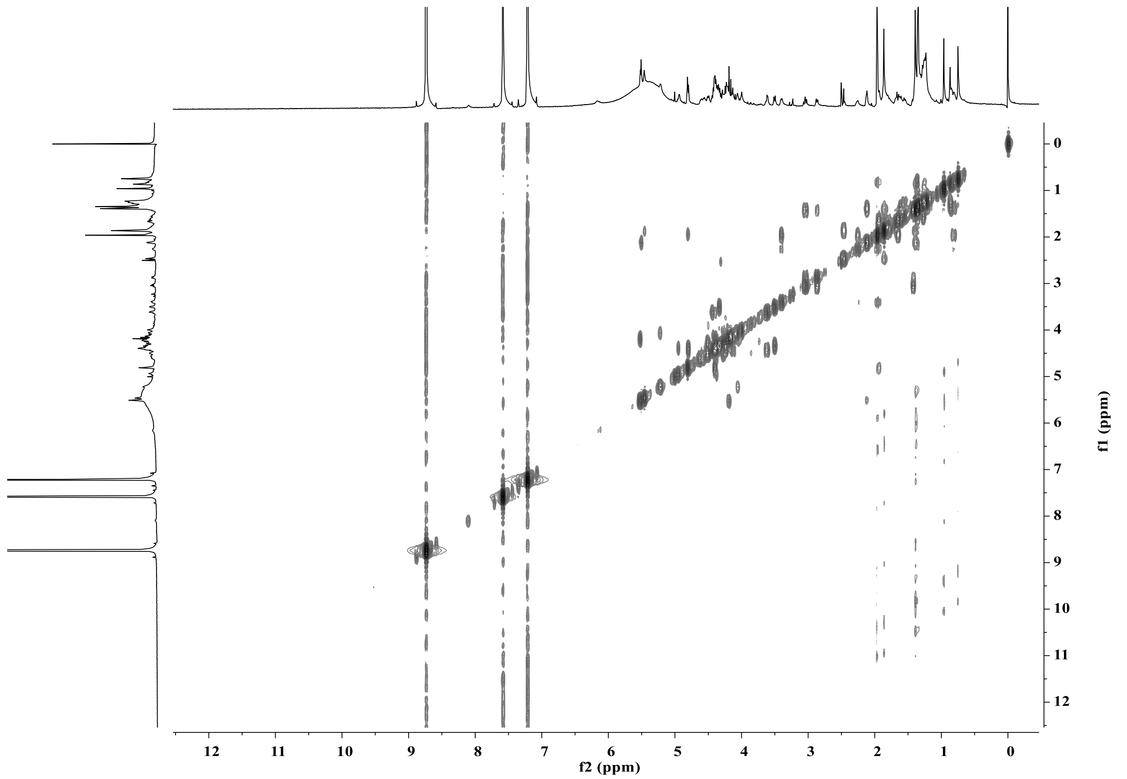

S81. ^{1}H-^{1}H COSY Spectrum of the new compound **12**

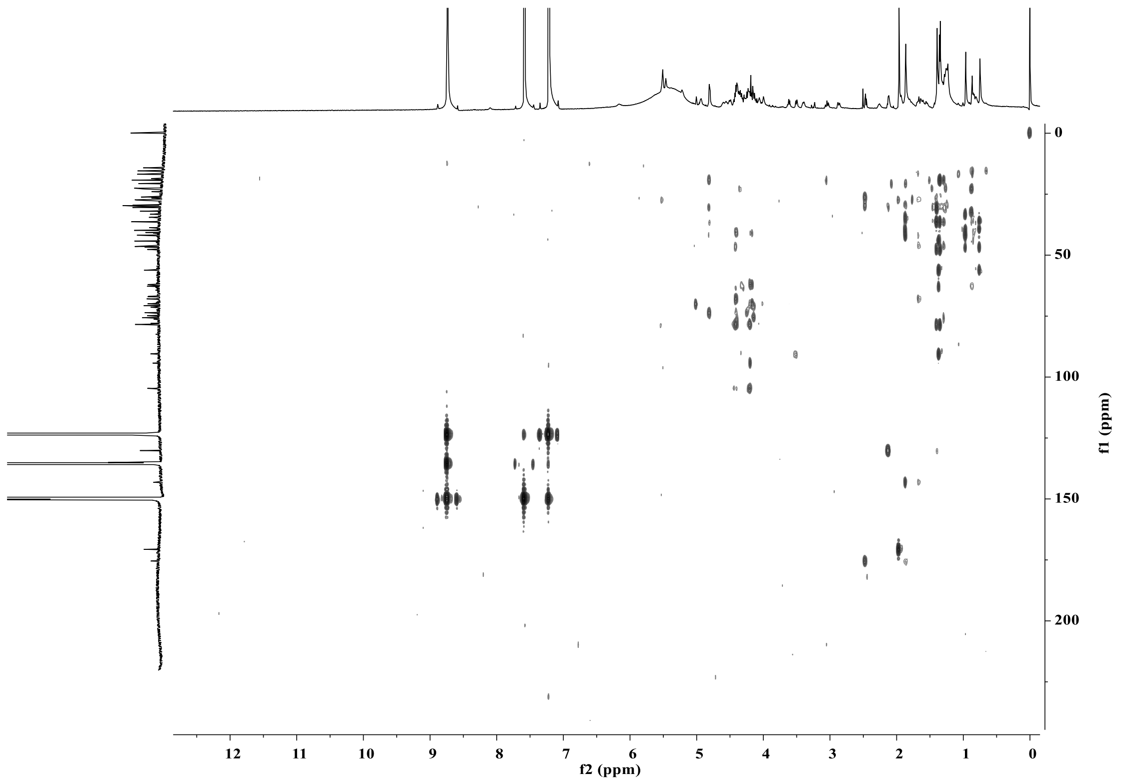

S82. HMBC Spectrum of the new compound **12**

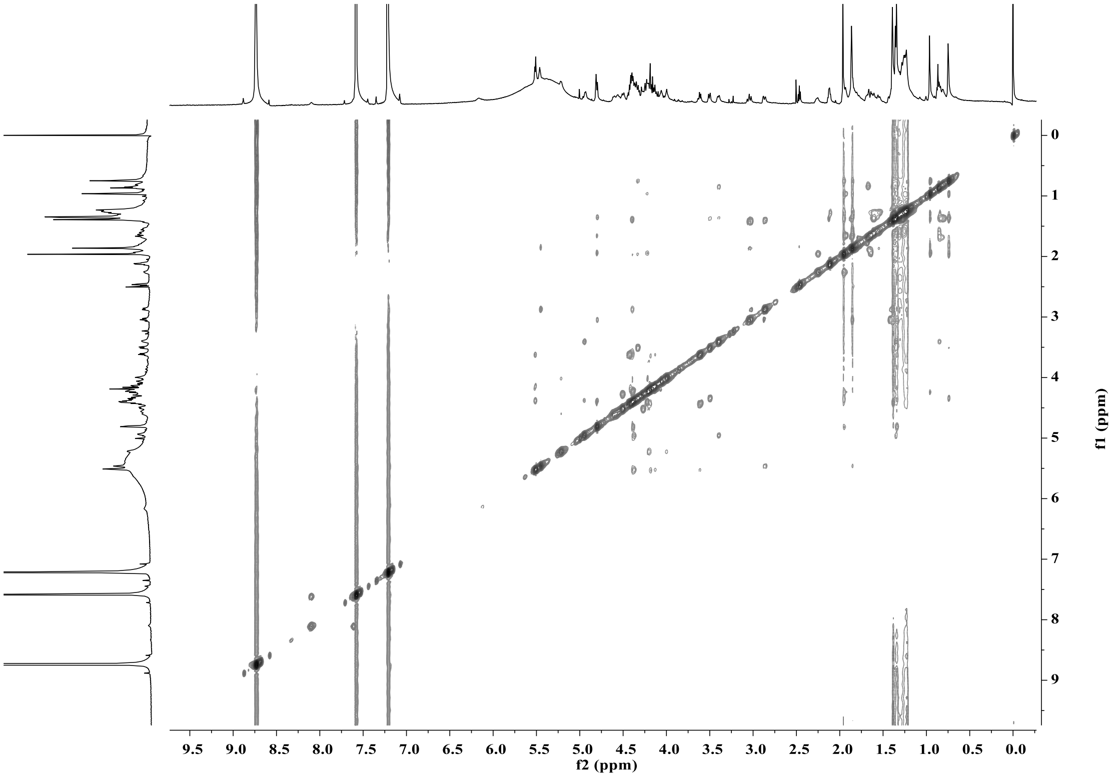

S83. NOESY Spectrum of the new compound **12**

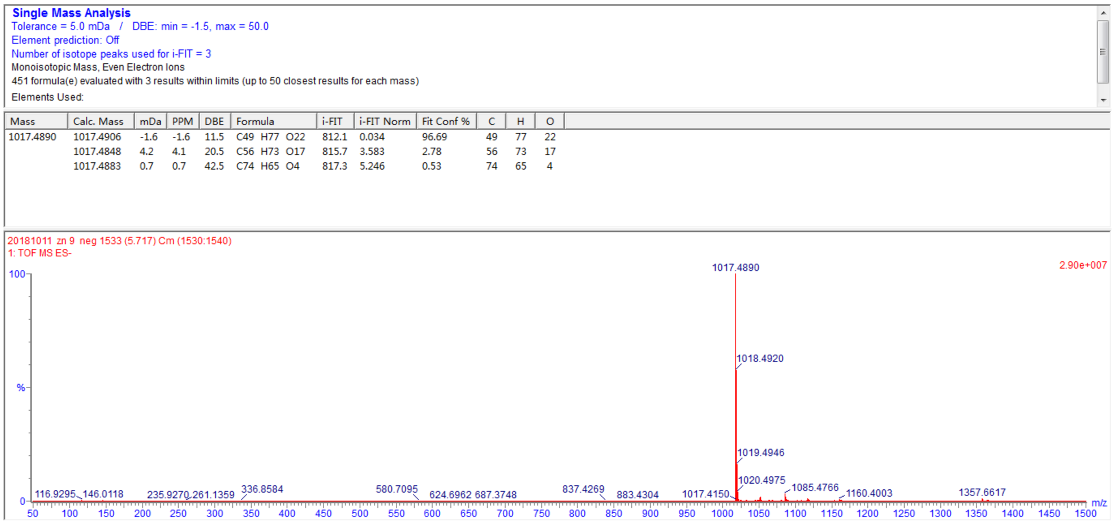

Single Mass Analysis
Tolerance = 5.0 mDa / DBE: min = -1.5, max = 50.0
Element prediction: Off
Number of isotope peaks used for i-FIT = 3
Monoisotopic Mass, Even Electron Ions
451 formula(e) evaluated with 3 results within limits (up to 50 closest results for each mass)
Elements Used:

Mass	Calc. Mass	mDa	PPM	DBE	Formula	i-FIT	i-FIT Norm	Fit Conf %	C	H	O
1017.4890	1017.4906	-1.6	-1.6	11.5	C49 H77 O22	812.1	0.034	96.69	49	77	22
	1017.4848	4.2	4.1	20.5	C56 H73 O17	815.7	3.583	2.78	56	73	17
	1017.4883	0.7	0.7	42.5	C74 H65 O4	817.3	5.246	0.53	74	65	4

S84. HRESIMS spectrum of the new compound **12**

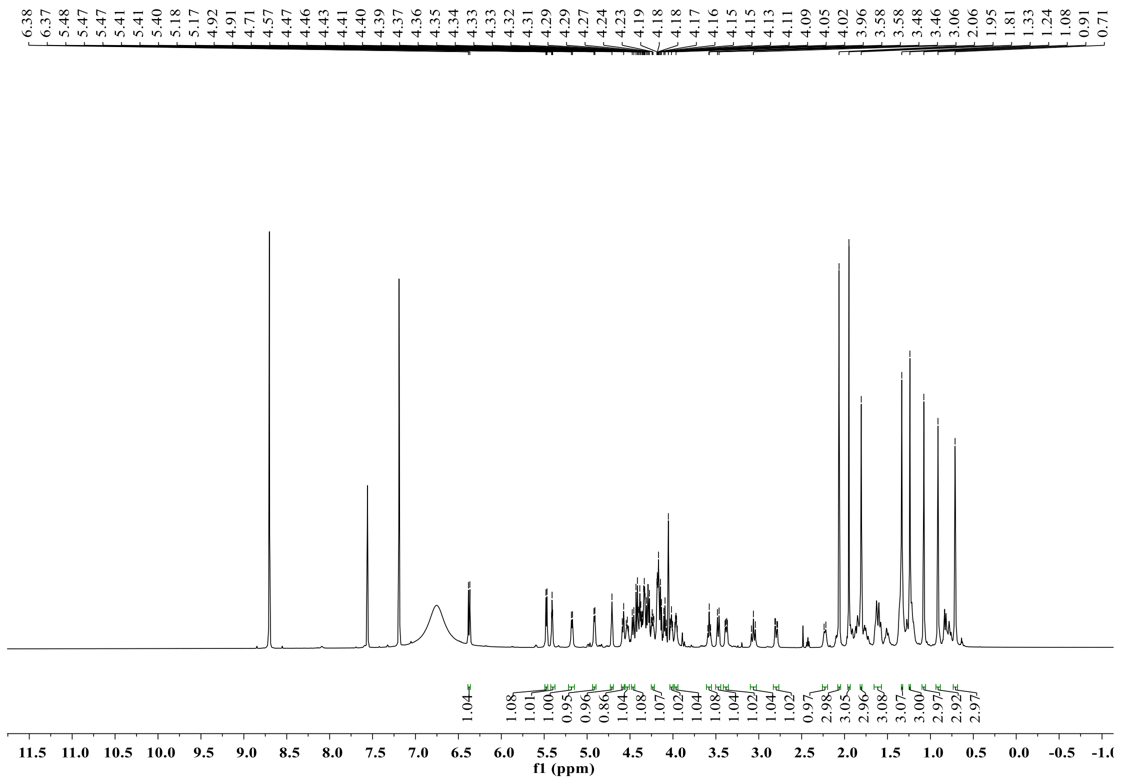

S85. ^{1}H NMR Spectrum (600 MHZ, Pyridine-d_5) of the new compound **13**

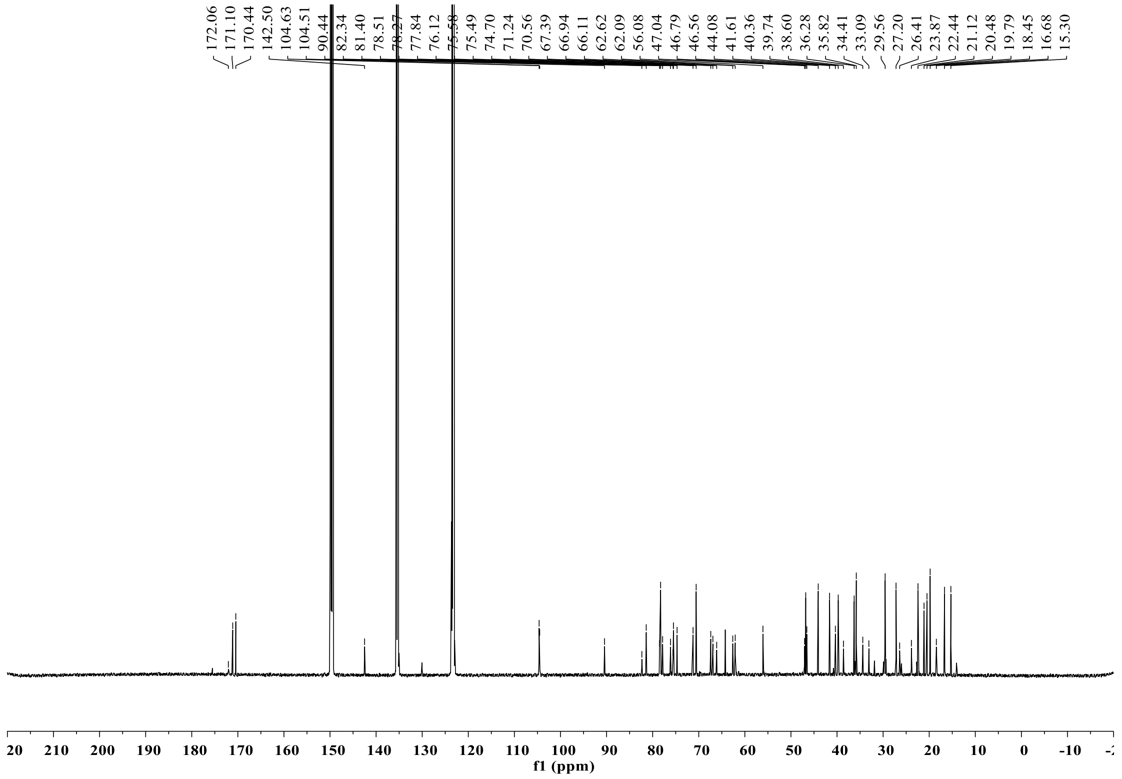

S86. ^{13}C NMR Spectrum (150 MHZ, Pyridine-d_5) of the new compound **13**

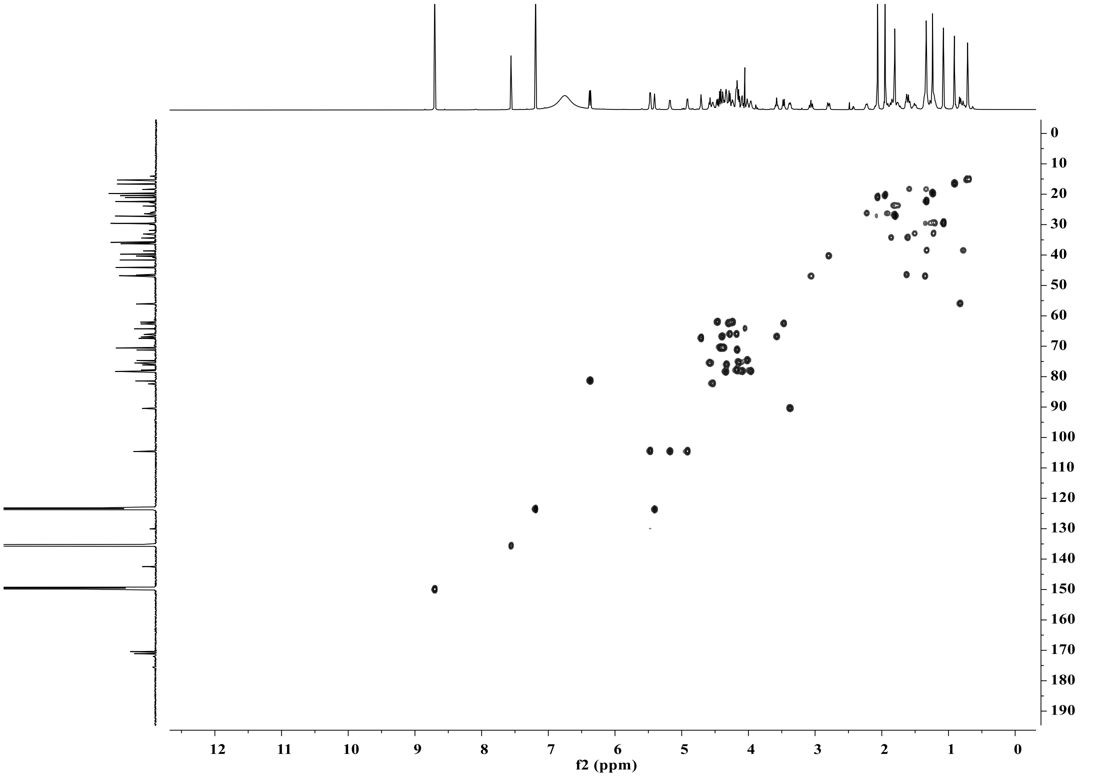

S87. HSQC Spectrum of the new compound **13**

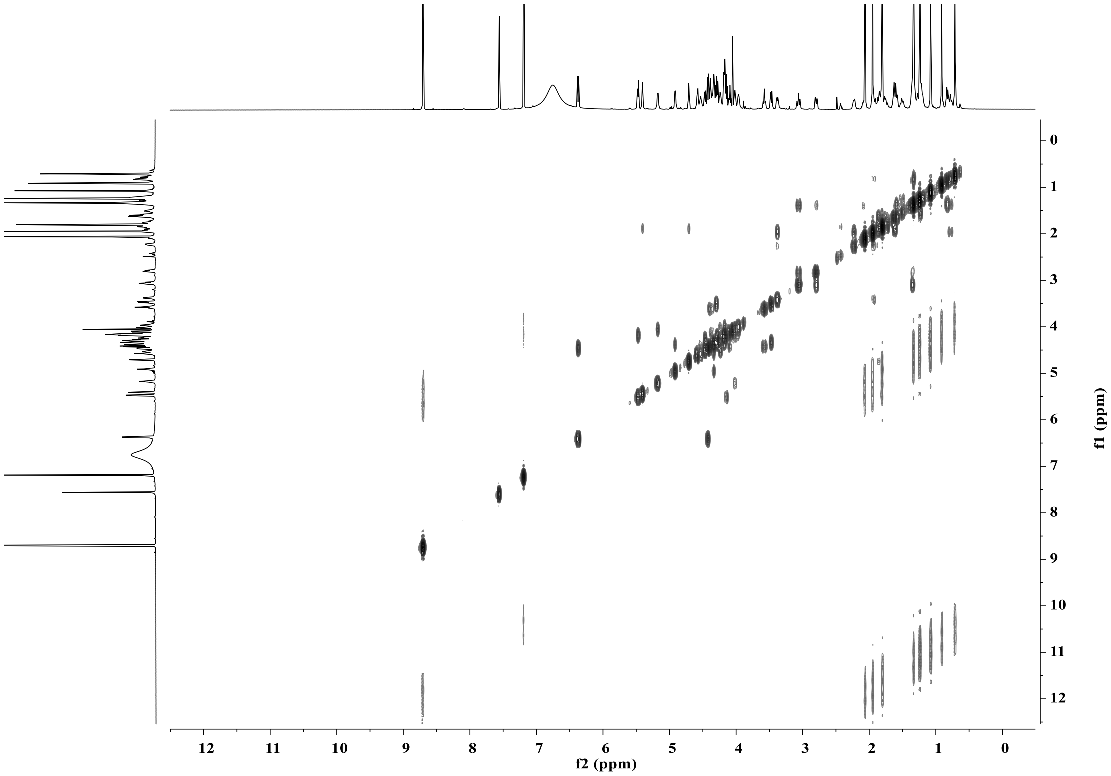

S88. ^{1}H-^{1}H COSY Spectrum of the new compound **13**

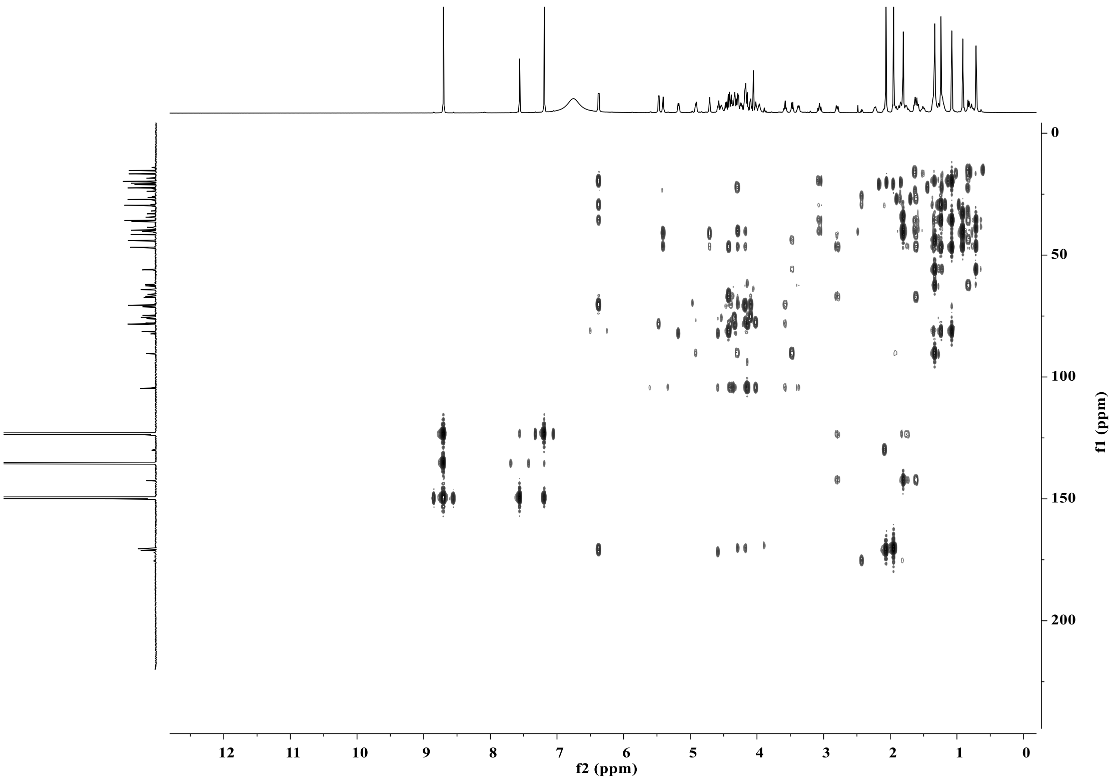

S89. HMBC Spectrum of the new compound **13**

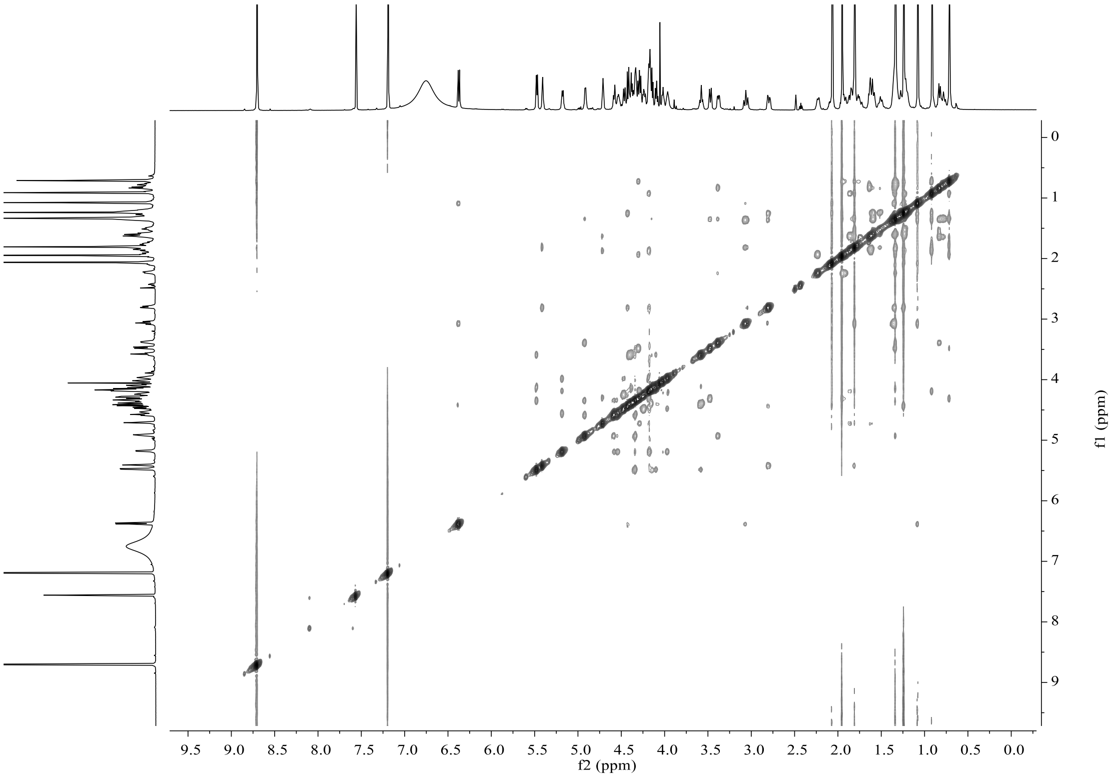

S90. NOESY Spectrum of the new compound **13**

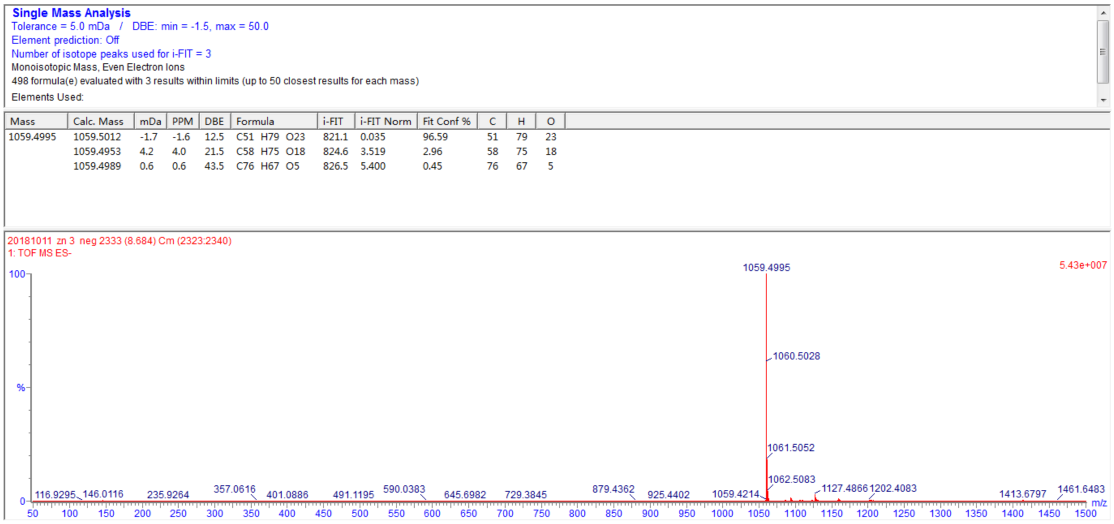

S91. HRESIMS spectrum of the new compound **13**

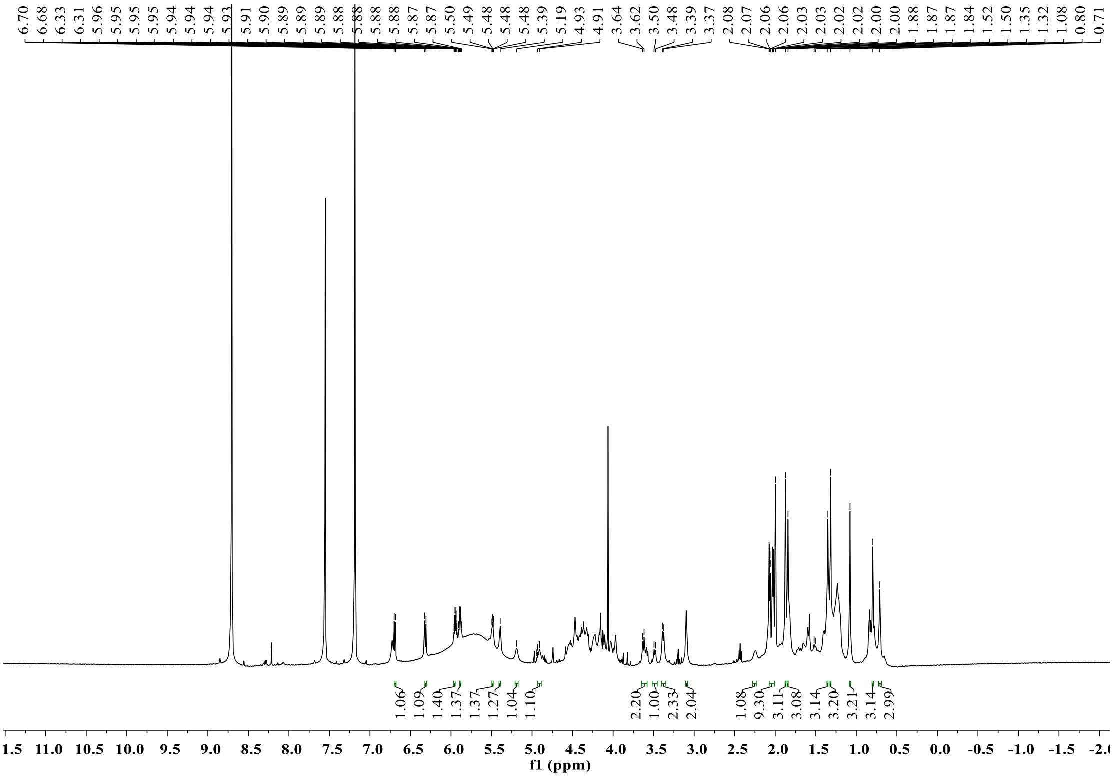

S92. ^{1}H NMR Spectrum (600 MHZ, Pyridine-d_5) of the new compound **14**

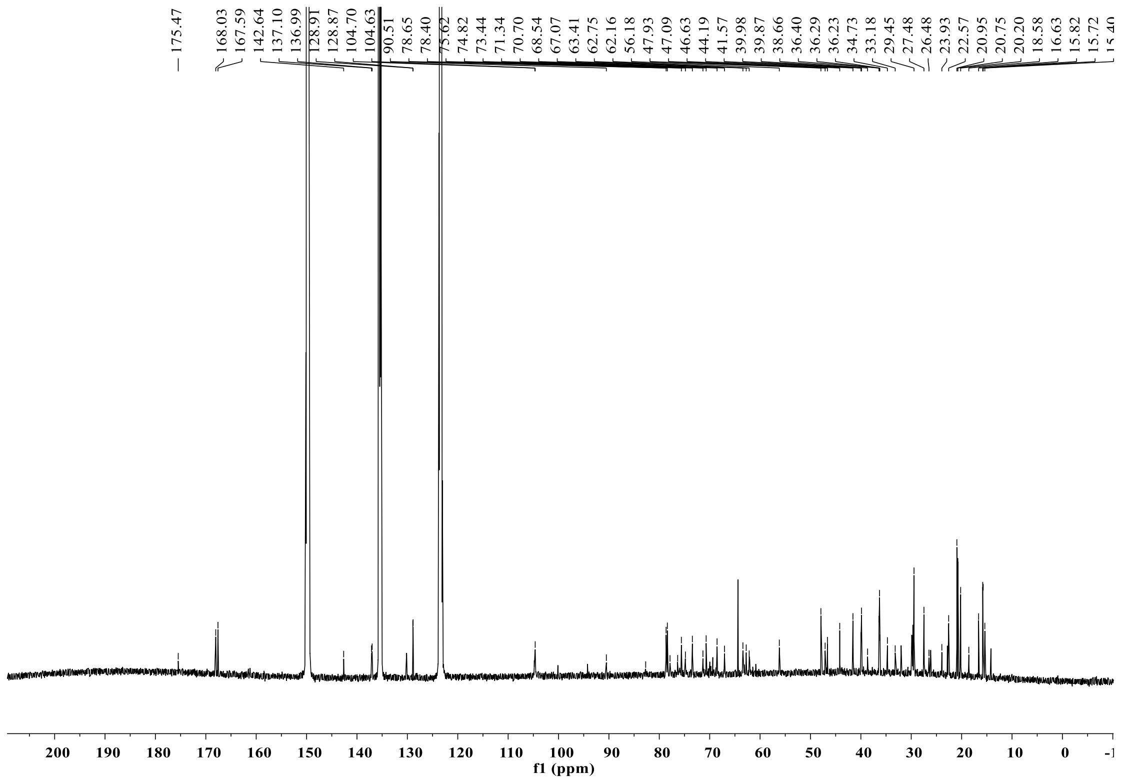

S93. ^{13}C NMR Spectrum (150 MHZ, Pyridine-d_5) of the new compound **14**

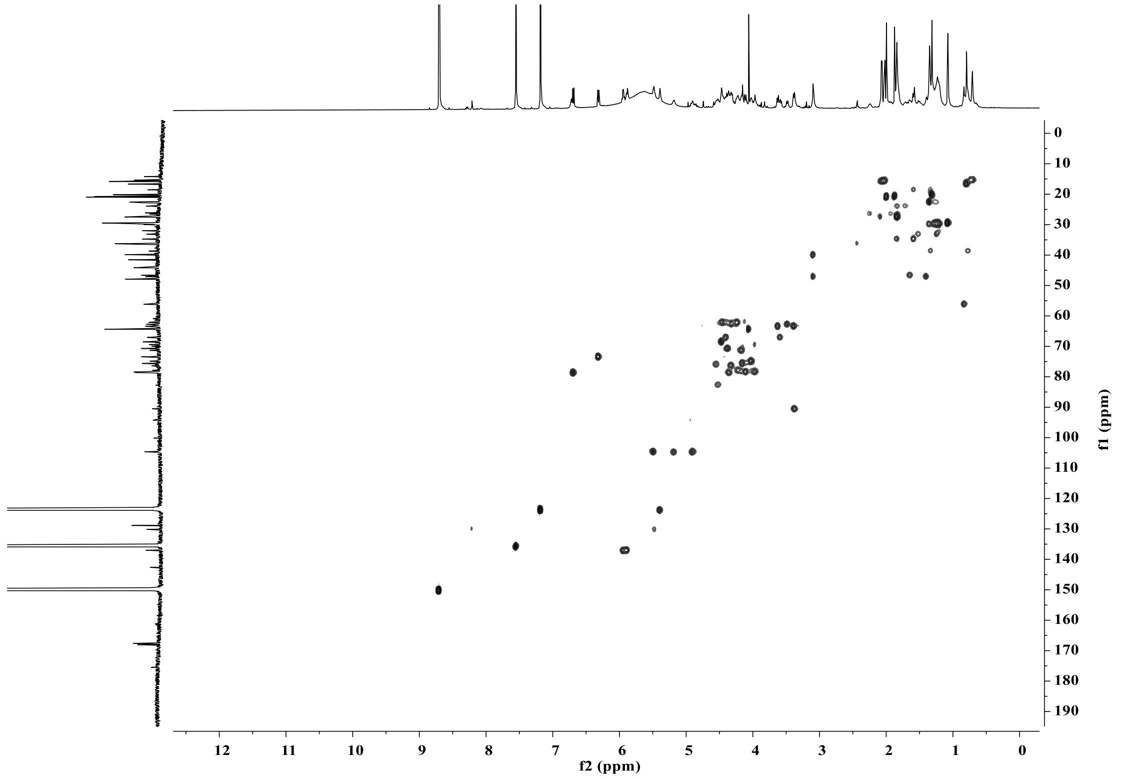

S94. HSQC Spectrum of the new compound **14**

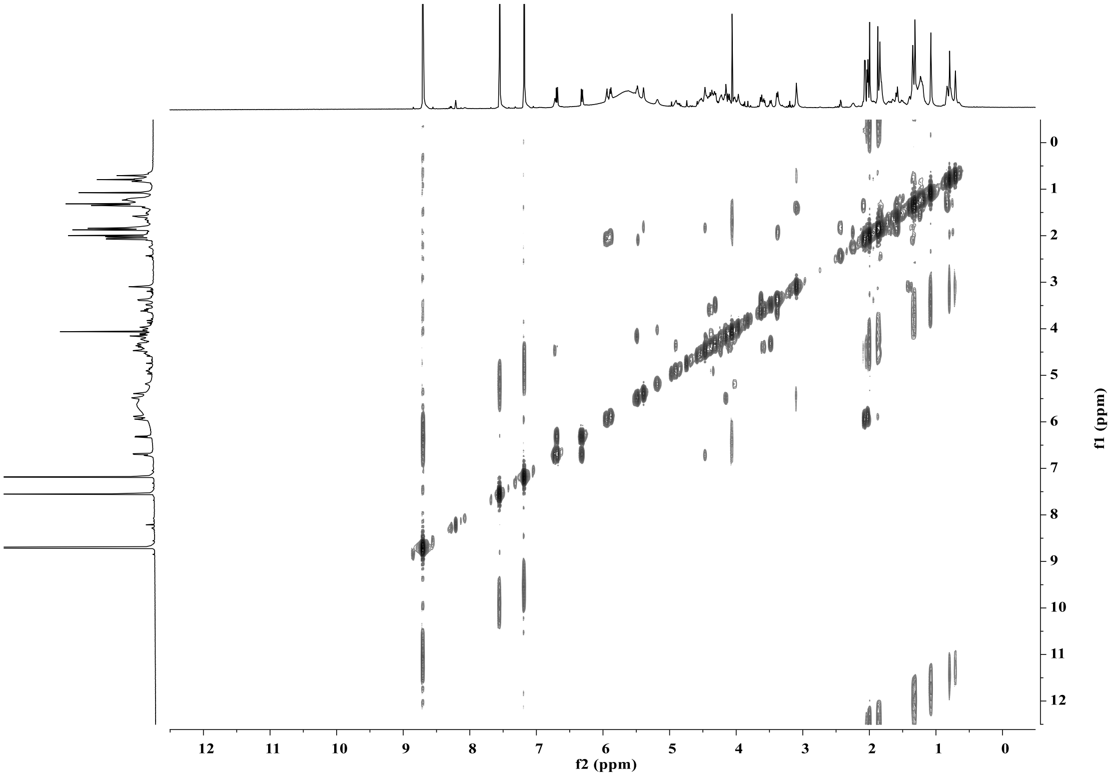

S95. ^{1}H-^{1}H COSY Spectrum of the new compound **14**

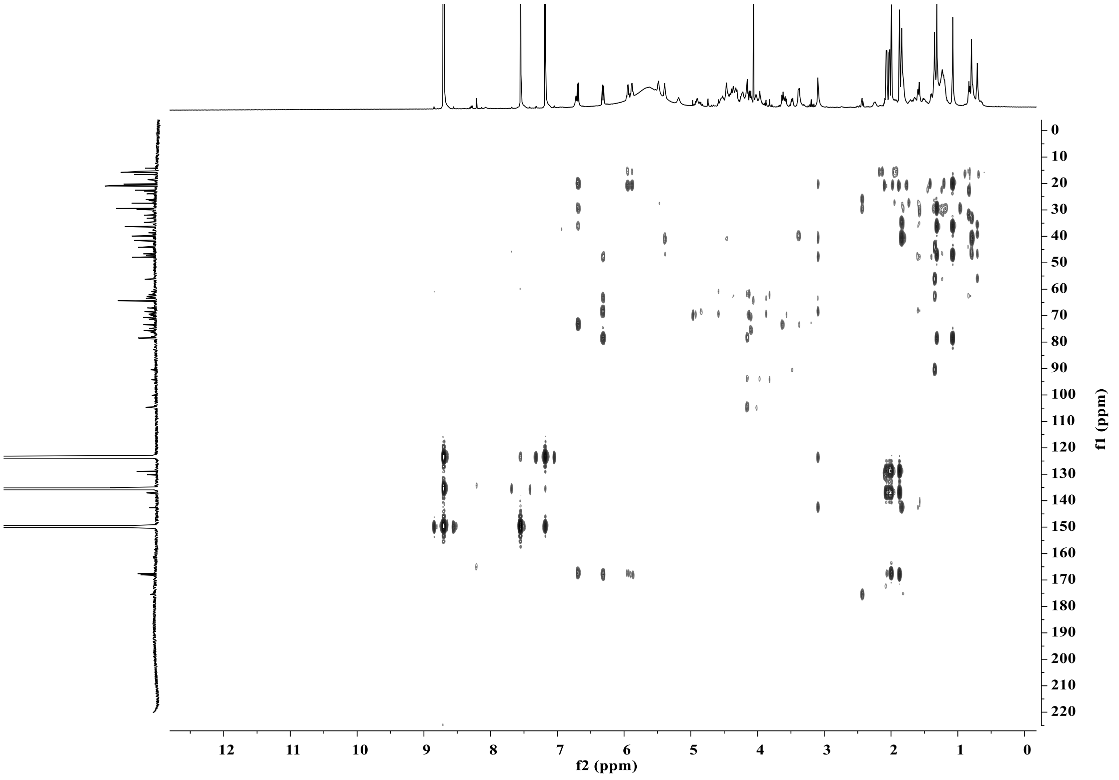

S96. HMBC Spectrum of the new compound **14**

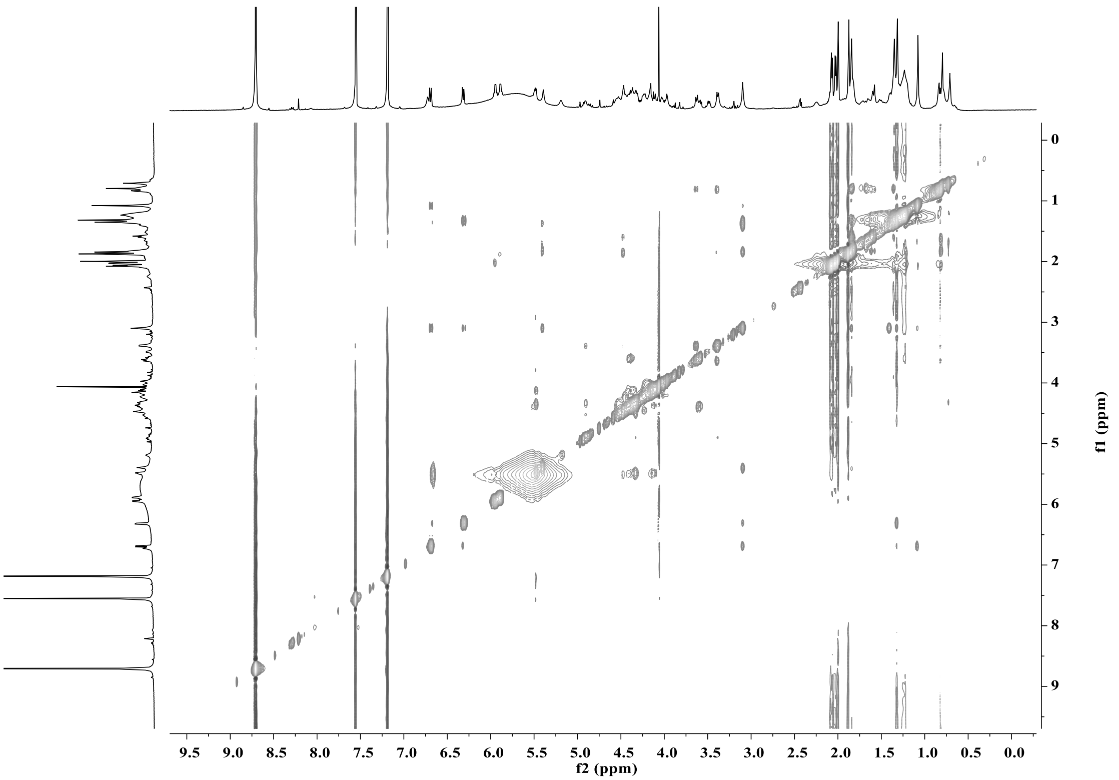

S97. NOESY Spectrum of the new compound **14**

Single Mass Analysis
Tolerance = 5.0 mDa / DBE: min = -1.5, max = 50.0
Element prediction: Off
Number of isotope peaks used for i-FIT = 3
Monoisotopic Mass, Even Electron Ions
559 formula(e) evaluated with 4 results within limits (up to 50 closest results for each mass)
Elements Used:

Mass	Calc. Mass	mDa	PPM	DBE	Formula	i-FIT	i-FIT Norm	Fit Conf %	C	H	O
1141.5785	1141.5795	-1.0	-0.9	13.5	C57 H89 O23	468.6	0.000	100.00	57	89	23
	1141.5771	1.4	1.2	44.5	C82 H77 O5	481.1	12.544	0.00	82	77	5
	1141.5830	-4.5	-3.9	35.5	C75 H81 O10	482.3	13.761	0.00	75	81	10
	1141.5736	4.9	4.3	22.5	C64 H85 O18	479.2	10.669	0.00	64	85	18

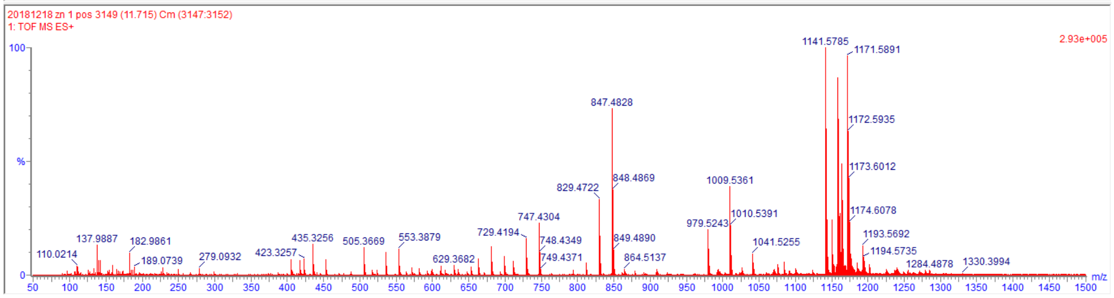

S98. HRESIMS spectrum of the new compound **14**

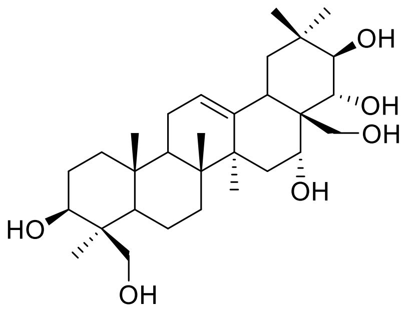

S99. The structure of compound **14a**

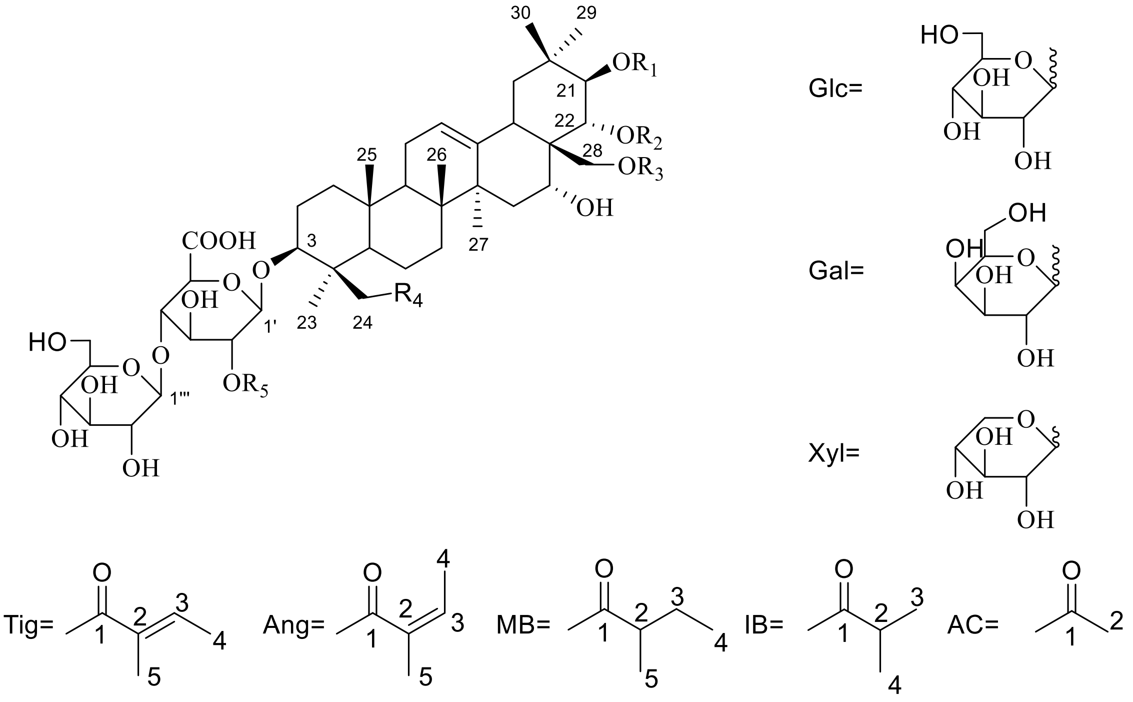

15	R_1=Tig,	R_2=Tig,	R_3=H,	R_4=H,	R_5=Gal-*p*
16	R_1=Ang,	R_2=Ang,	R_3=H,	R_4=OH,	R_5=Glc-*p*
17	R_1=Tig,	R_2=Ac,	R_3=H,	R_4=OH,	R_5=Glc-*p*
18	R_1=Ang,	R_2=Ac,	R_3=H,	R_4=OH,	R_5=Glc-*p*
19	R_1=Tig,	R_2=H,	R_3=Ac,	R_4=OH,	R_5=Glc-*p*
20	R_1=Ang,	R_2=H,	R_3=Ac,	R_4=OH,	R_5=Glc-*p*
21	R_1=Ang,	R_2=H,	R_3=Ac,	R_4=OH,	R_5=Xyl-*p*
22	R_1=Tig,	R_2=Ac,	R_3=H,	R_4=H,	R_5=Gal-*p*
23	R_1=Ac,	R_2=Ac,	R_3=H,	R_4=OH,	R_5=Glc-*p*
24	R_1=IB,	R_2=Ac,	R_3=H,	R_4=OH,	R_5=Glc-*p*
25	R_1=H,	R_2=H,	R_3=Ac,	R_4=OH,	R_5=Glc-*p*
26	R_1=H,	R_2=Ac,	R_3=Ac,	R_4=OH,	R_5=Glc-*p*
27	R_1=Ac,	R_2=H,	R_3=H,	R_4=OH,	R_5=Glc-*p*
28	R_1=Tig,	R_2=H,	R_3=H,	R_4=OH,	R_5=Glc-*p*
29	R_1=Ang,	R_2=H,	R_3=H,	R_4=OH,	R_5=Glc-*p*
30	R_1=Ac,	R_2=H,	R_3=Ac,	R_4=OH,	R_5=Glc-*p*
31	R_1=H,	R_2=H,	R_3=H,	R_4=OH,	R_5=Glc-*p*
32	R_1=H,	R_2=H,	R_3=H,	R_4=OH,	R_5=Xyl-*p*
33	R_1=Ang,	R_2=H,	R_3=H,	R_4=OH,	R_5=Xyl-*p*

S100. The structures of compounds **15-33**

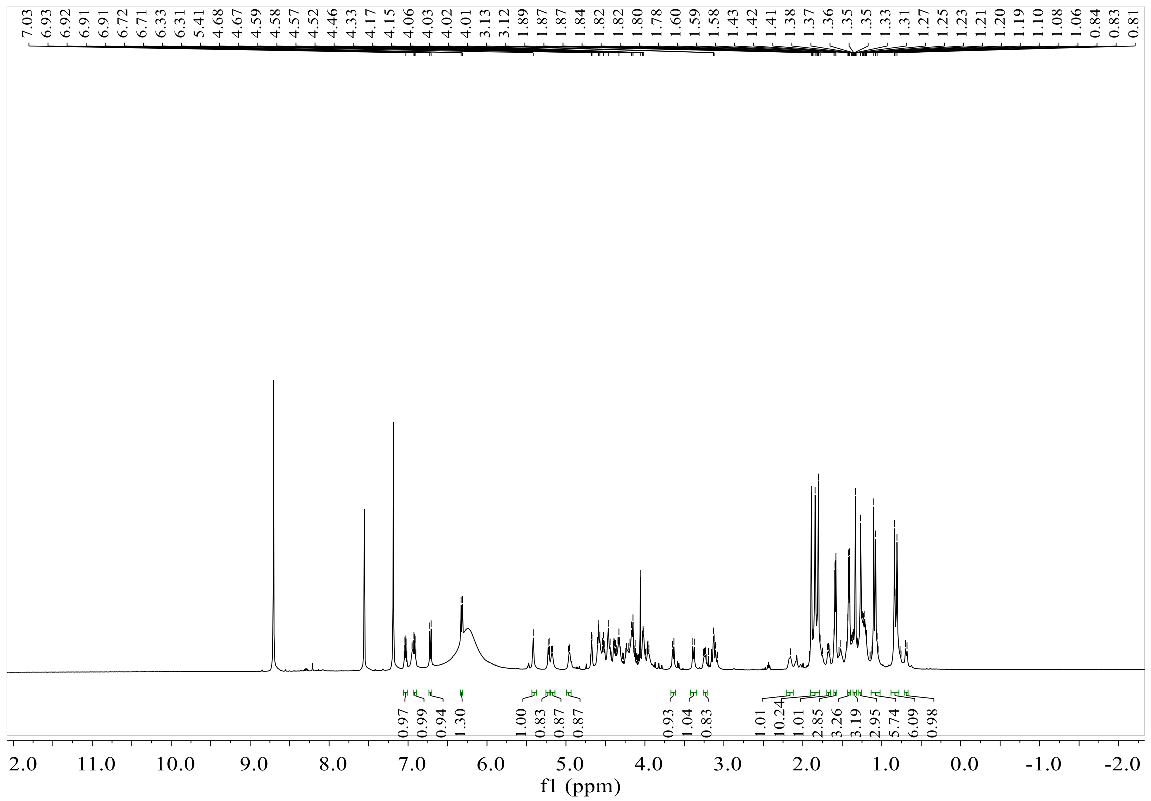

S101. ^{1}H NMR Spectrum (600 MHZ, Pyridine-d_5) of the new compound **15**

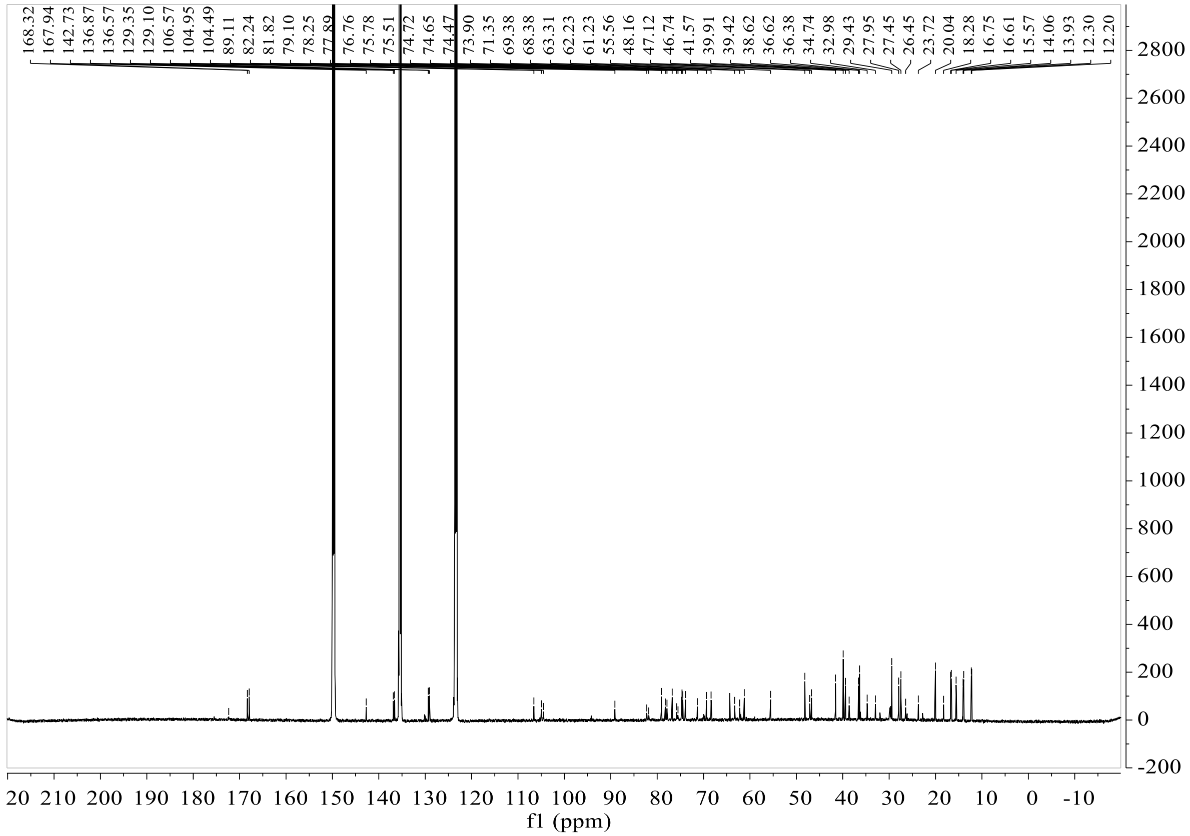

S102. ^{13}C NMR Spectrum (150 MHZ, Pyridine-d_5) of the new compound **15**

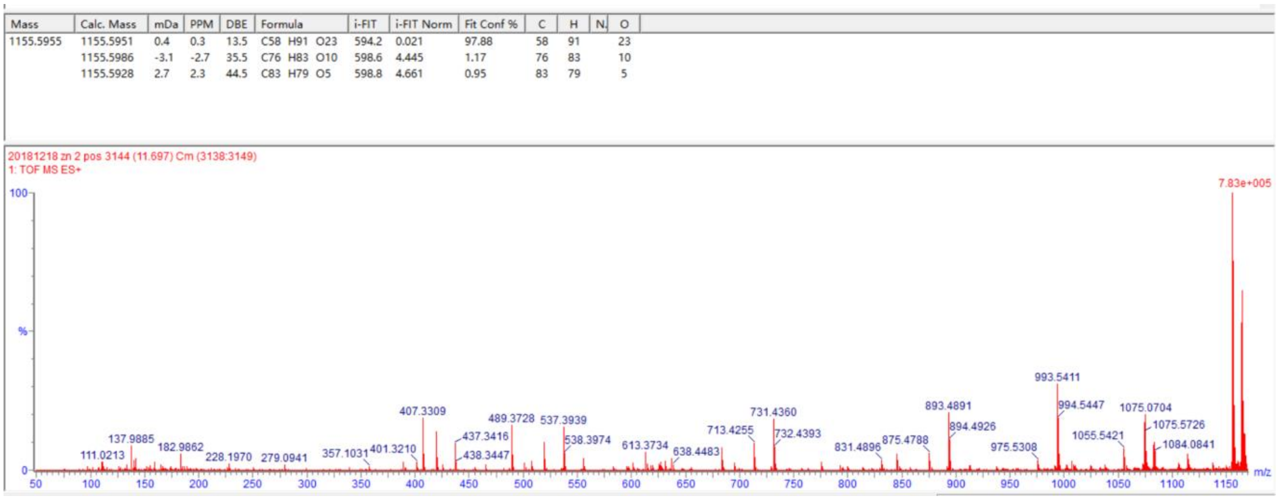

S103. HRESIMS spectrum of the new compound **15**

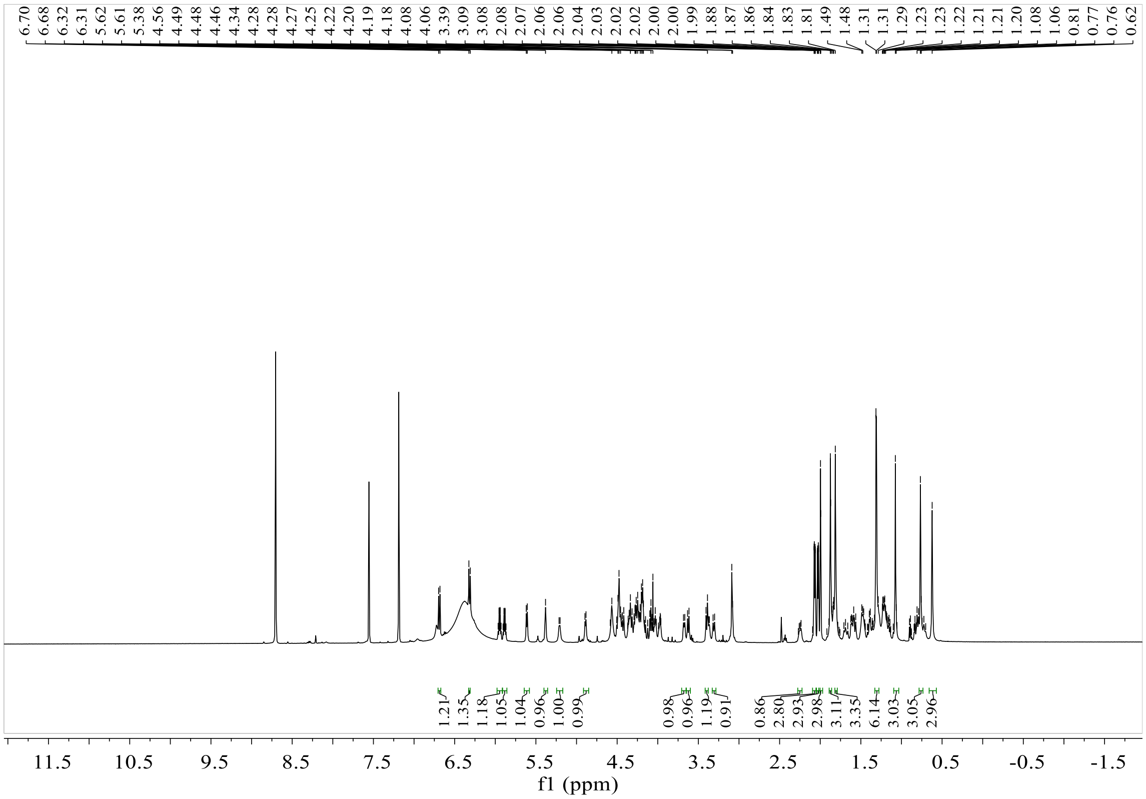

S104. ^{1}H NMR Spectrum (600 MHZ, Pyridine-d_5) of the new compound **16**

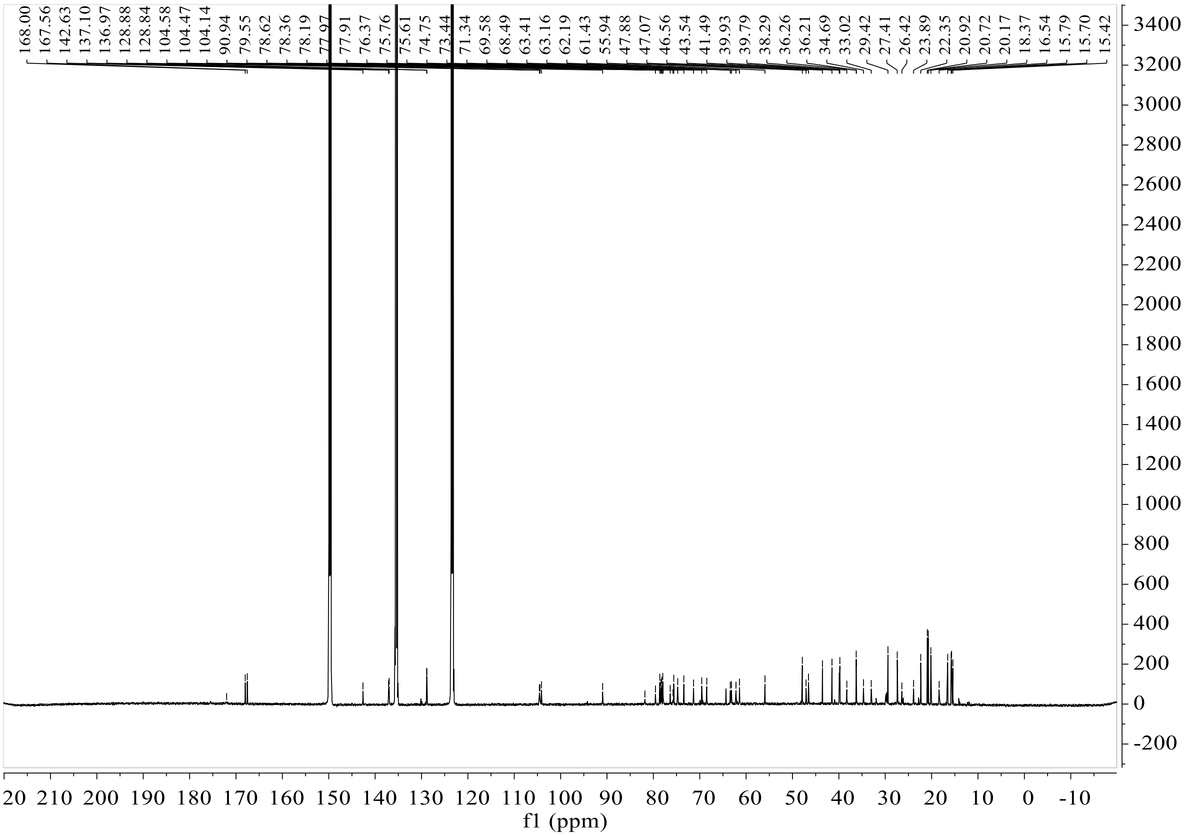

S105. ^{13}C NMR Spectrum (150 MHZ, Pyridine-d_5) of the new compound **16**

Mass	Calc. Mass	mDa	PPM	DBE	Formula	i-FIT	i-FIT Norm	Fit Conf %	C	H	N	O
1171.5903	1171.5900	0.3	0.3	13.5	C58 H91 O24	694.8	n/a	n/a	58	91		24

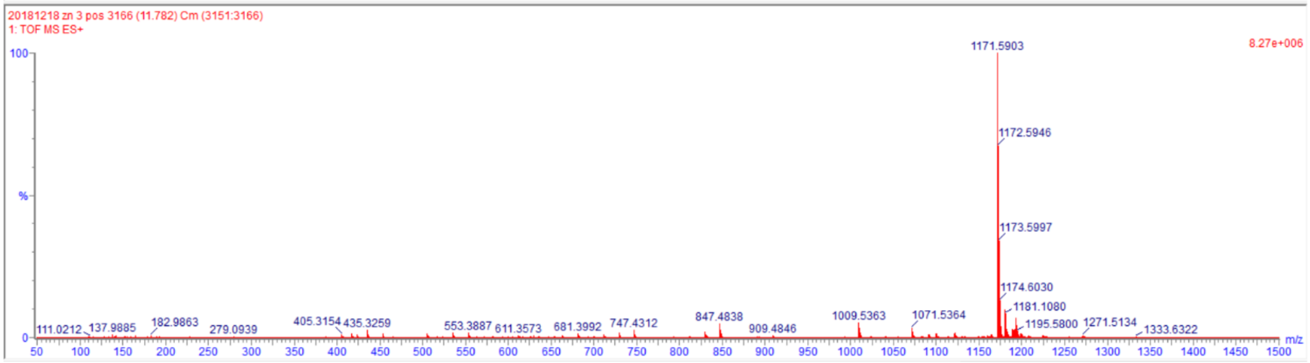

S106. HRESIMS spectrum of the new compound **16**

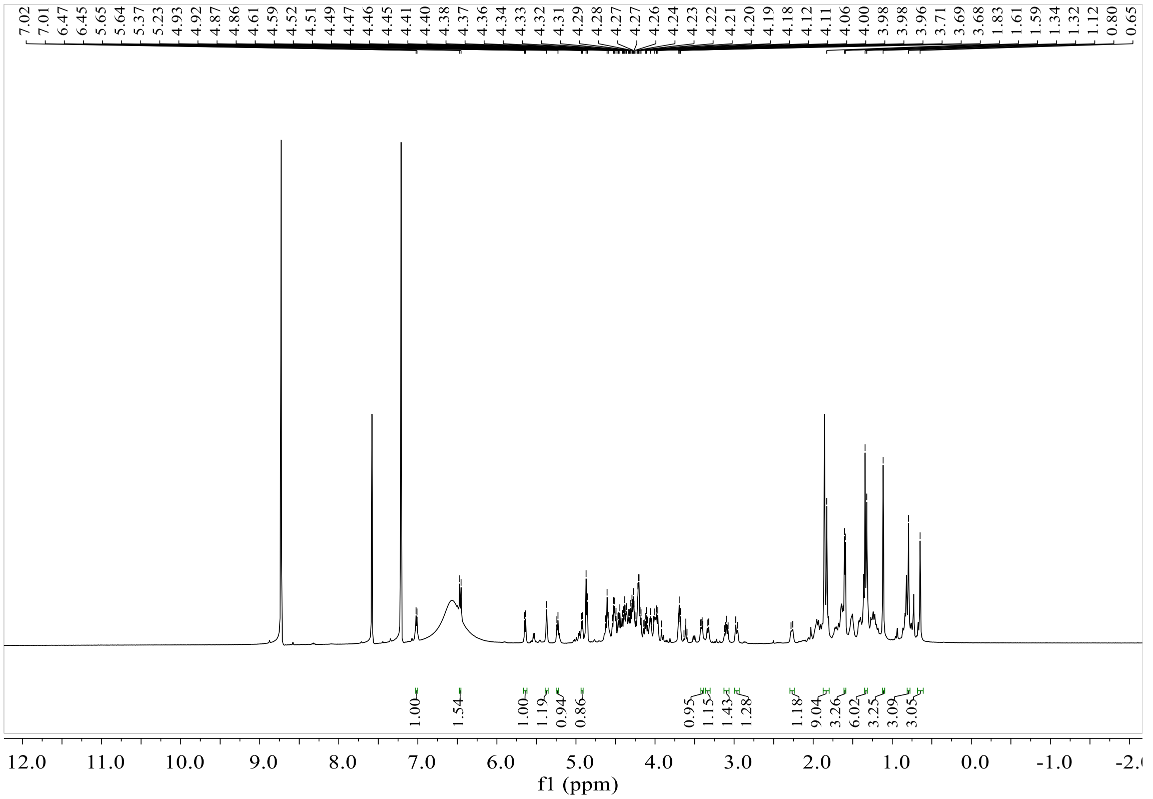

S107. ^{1}H NMR Spectrum (600 MHZ, Pyridine-d_5) of the new compound **17**

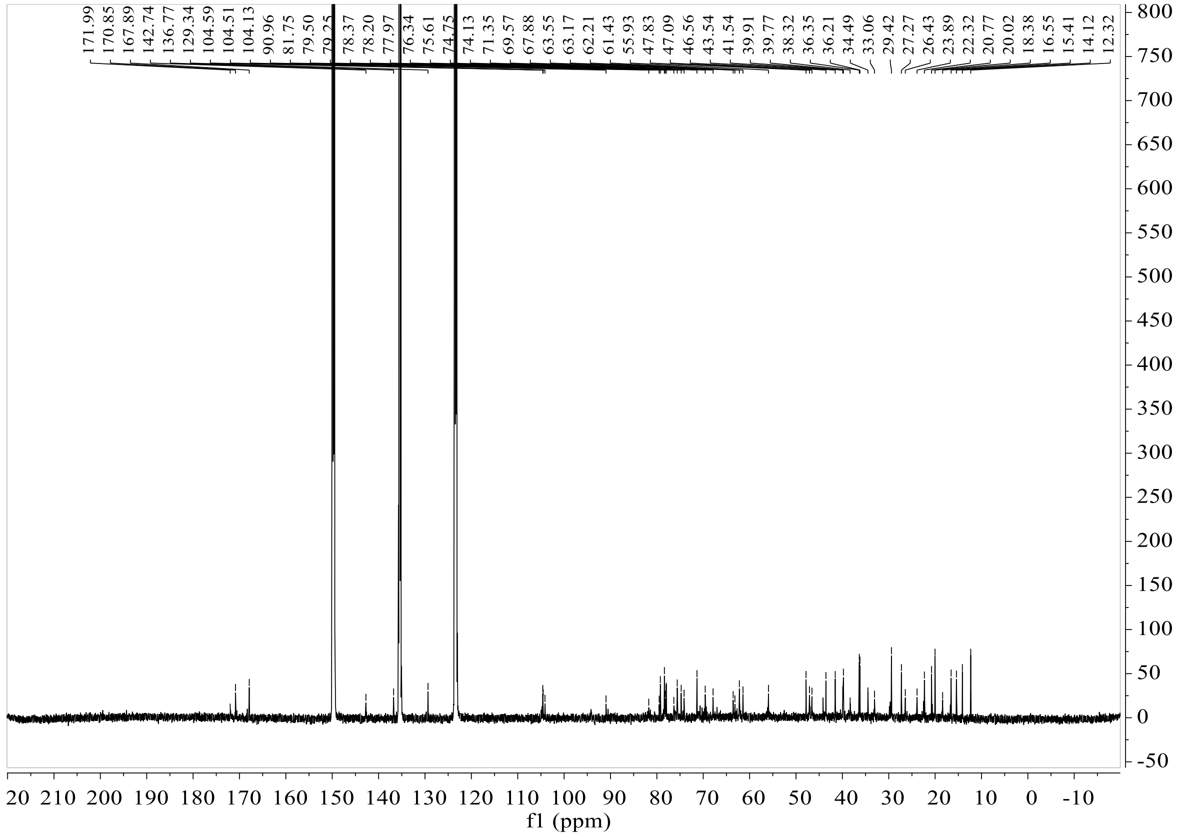

S108. ^{13}C NMR Spectrum (150 MHZ, Pyridine-d_5) of the new compound **17**

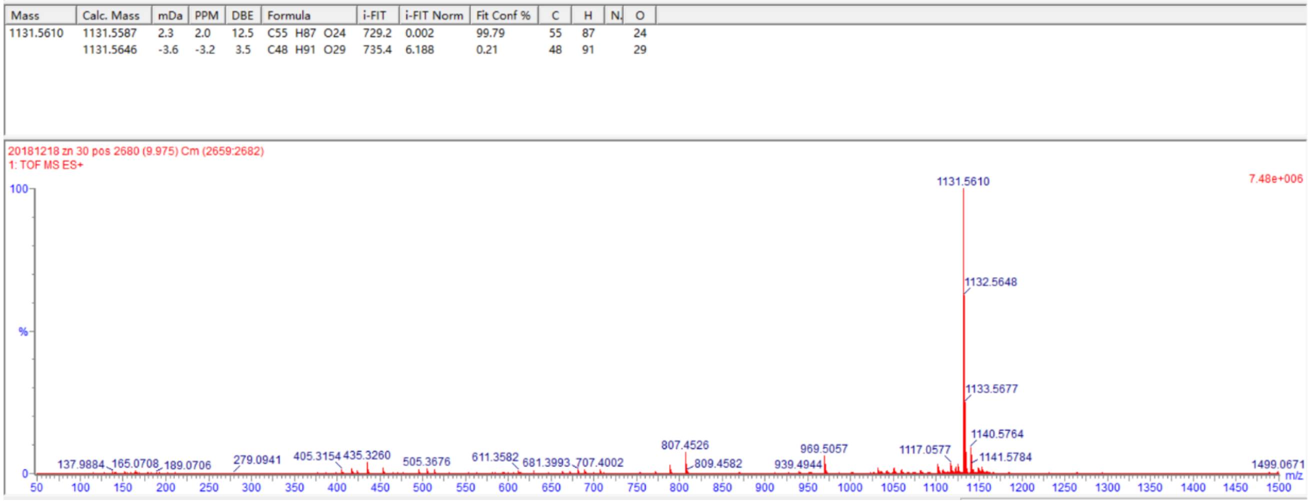

S109. HRESIMS spectrum of the new compound **17**

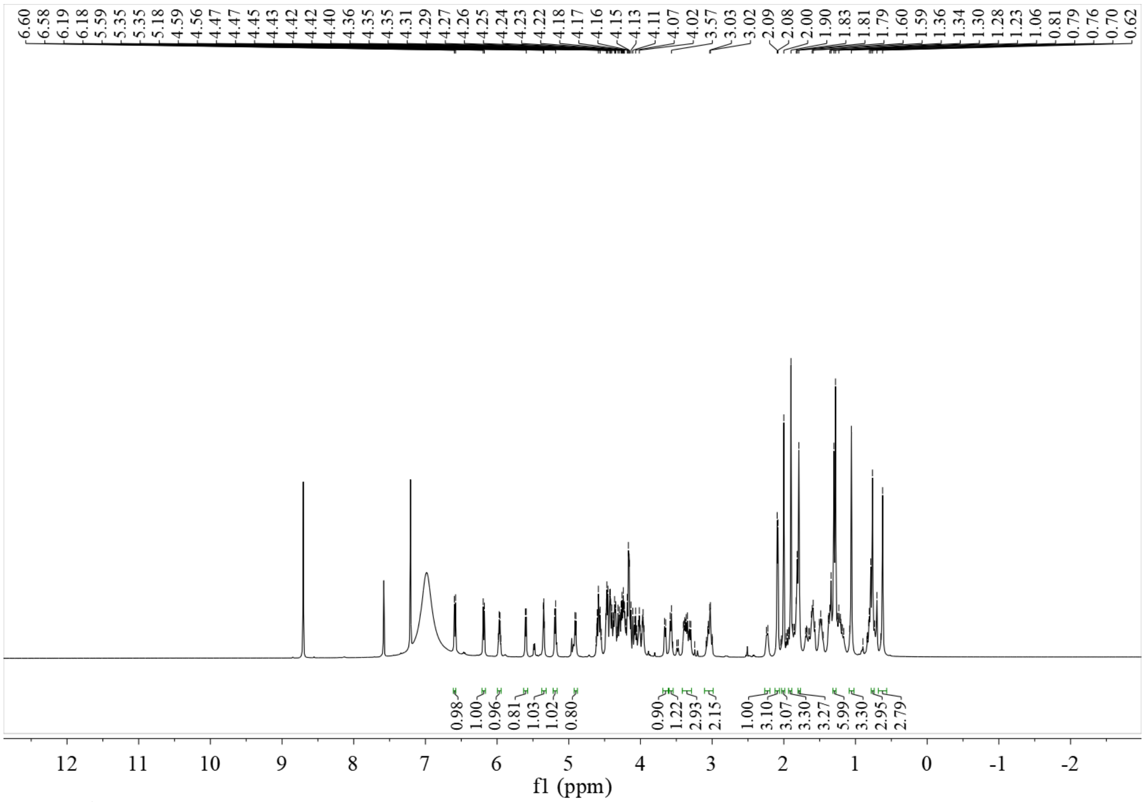

S110. ^{1}H NMR Spectrum (600 MHZ, Pyridine-d_5) of the new compound **18**

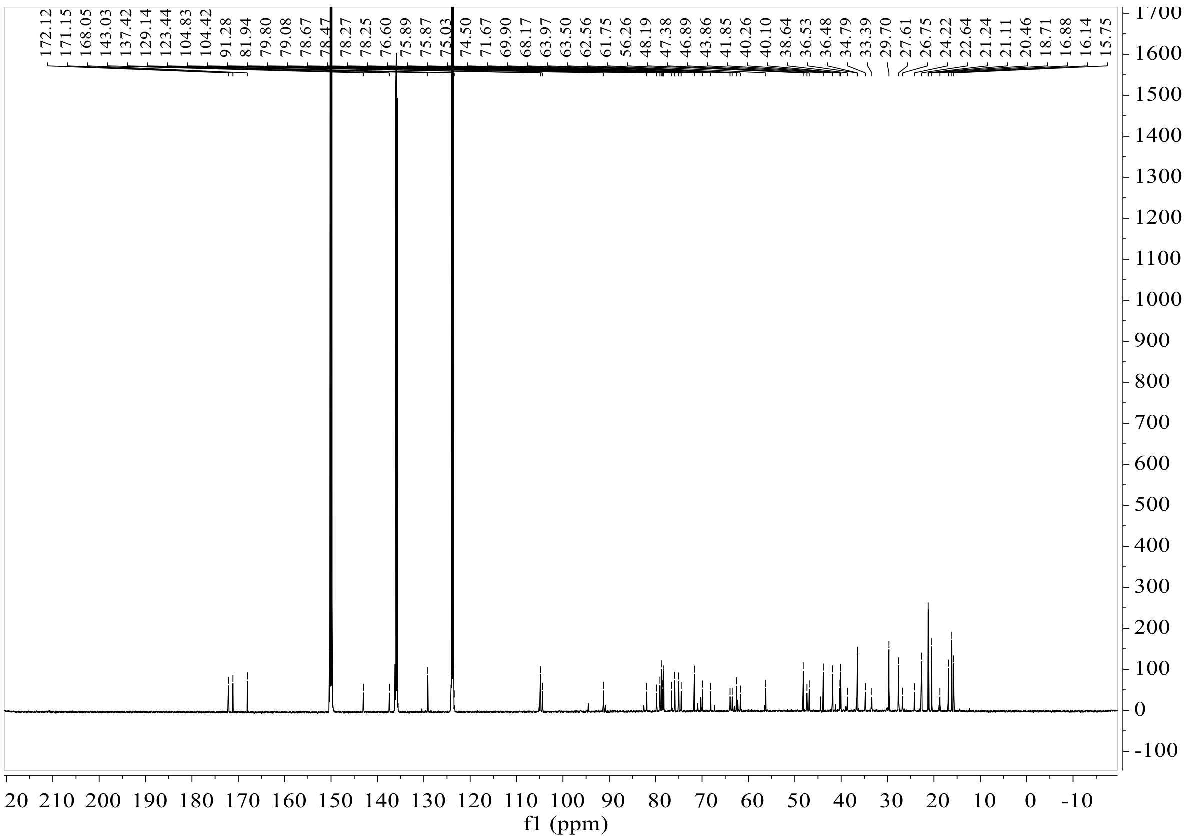

S111. ^{13}C NMR Spectrum (150 MHZ, Pyridine-d_5) of the new compound **18**

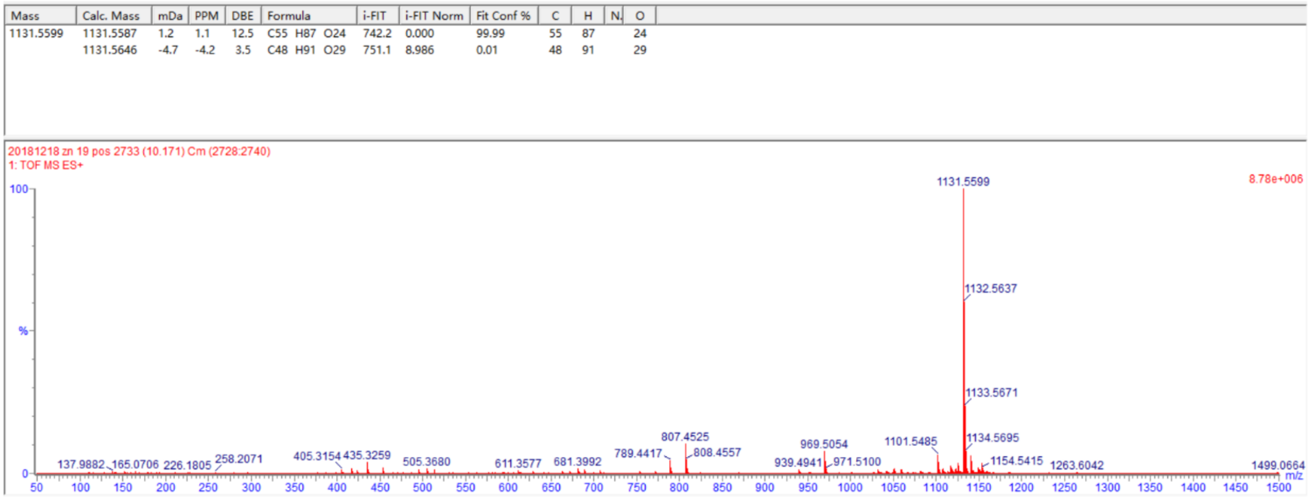

Mass	Calc. Mass	mDa	PPM	DBE	Formula	i-FIT	i-FIT Norm	Fit Conf %	C	H	N	O
1131.5599	1131.5587	1.2	1.1	12.5	C55 H87 O24	742.2	0.000	99.99	55	87		24
	1131.5646	-4.7	-4.2	3.5	C48 H91 O29	751.1	8.986	0.01	48	91		29

S112. HRESIMS spectrum of the new compound **18**

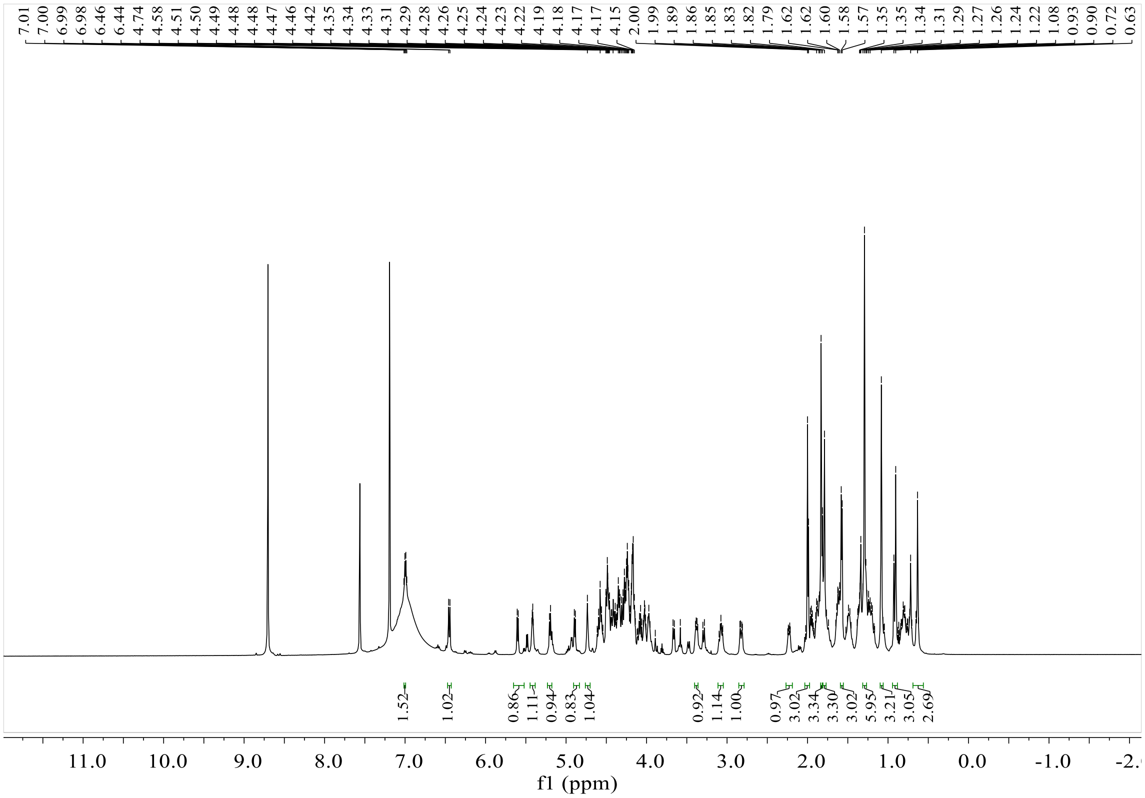

S113. ^{1}H NMR Spectrum (600 MHZ, Pyridine-d_5) of the new compound **19**

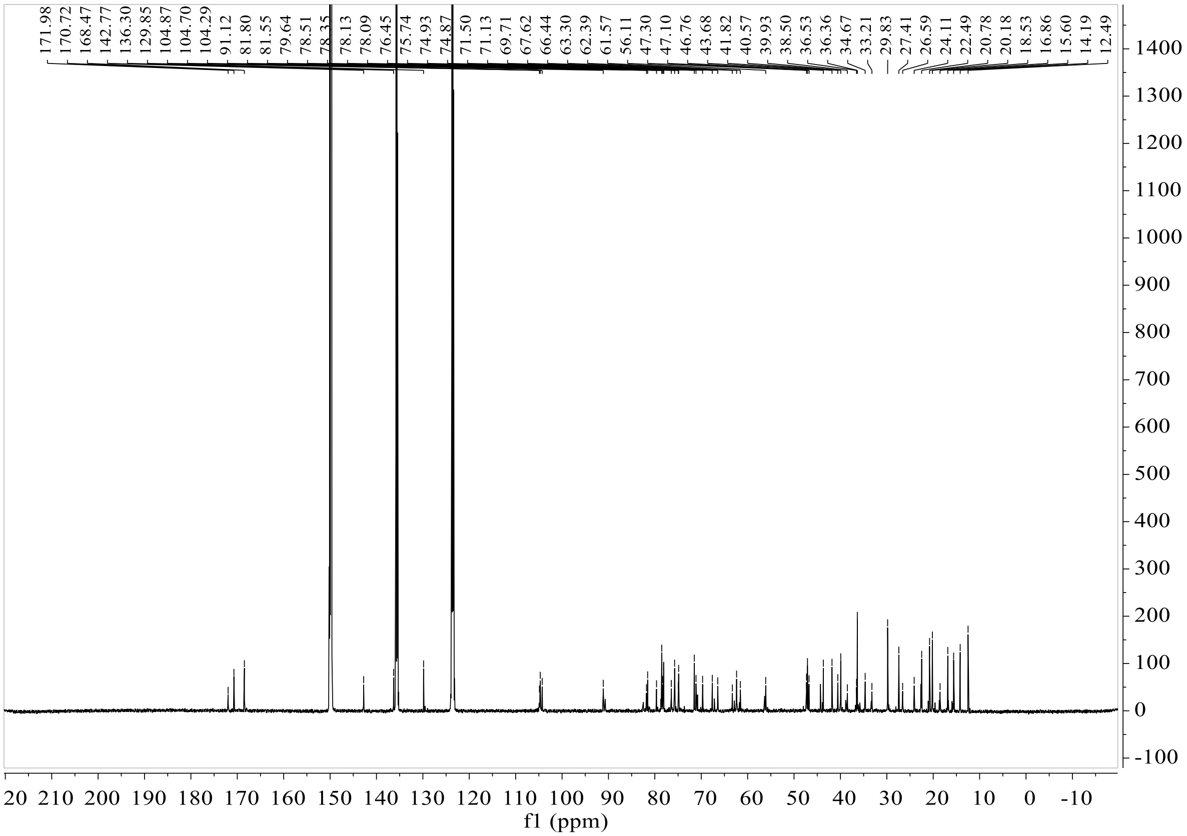

S114. ^{13}C NMR Spectrum (150 MHZ, Pyridine-d_5) of the new compound **19**

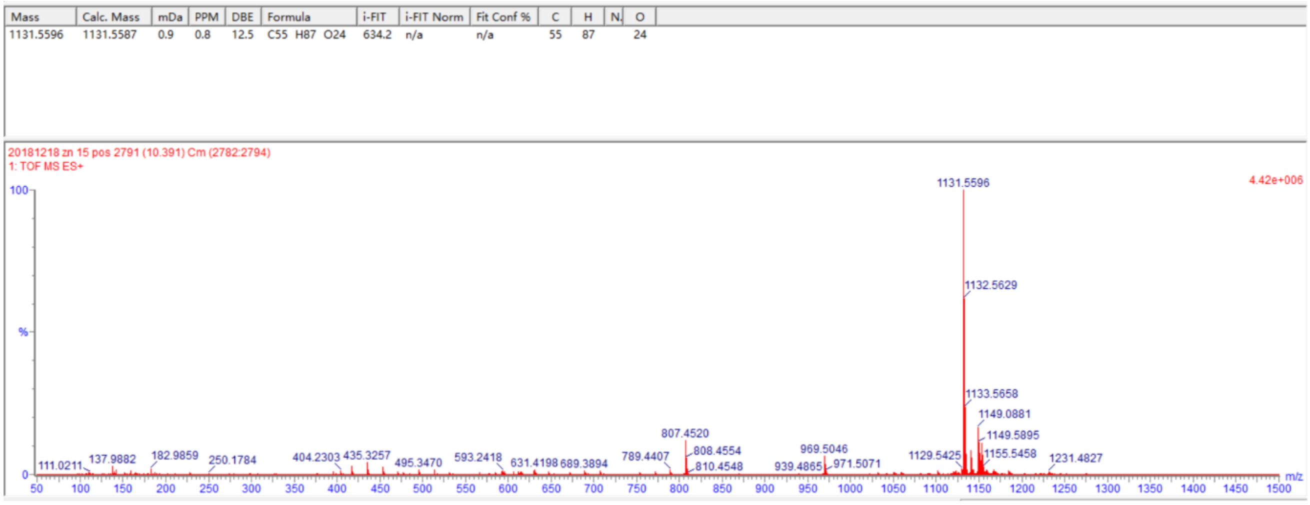

S115. HRESIMS spectrum of the new compound **19**

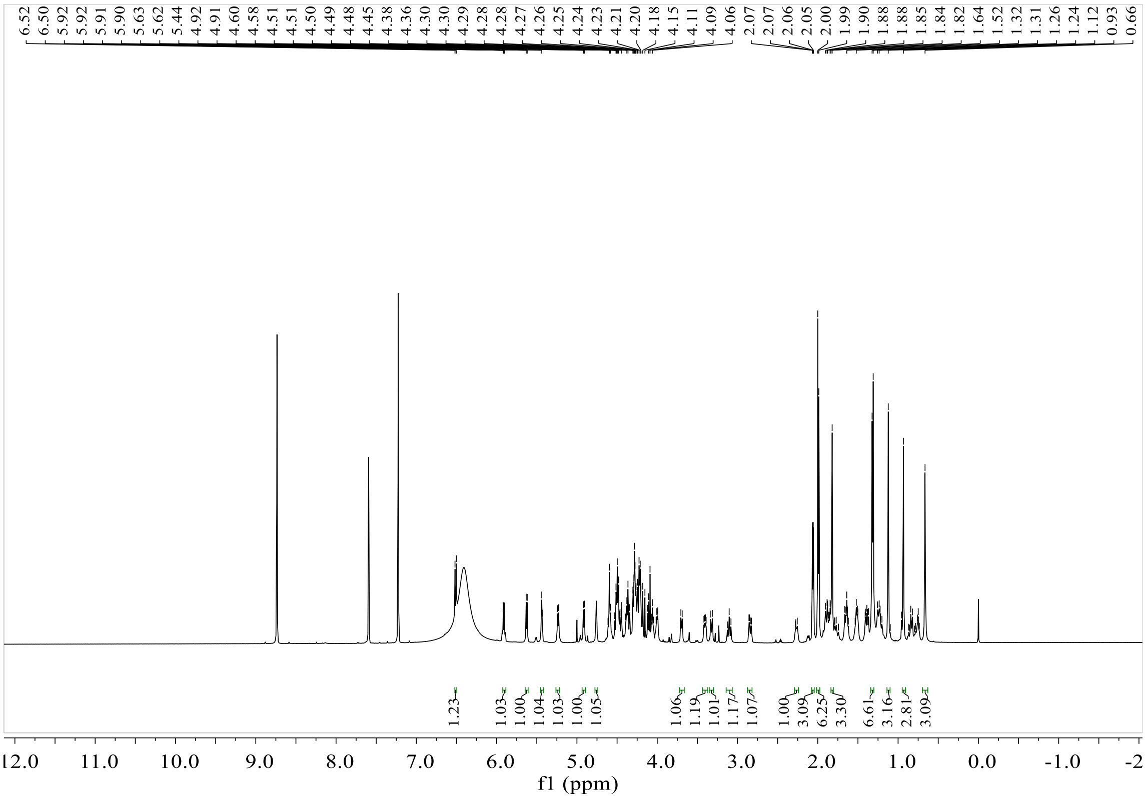

S116. ^{1}H NMR Spectrum (600 MHZ, Pyridine-d_5) of the new compound **20**

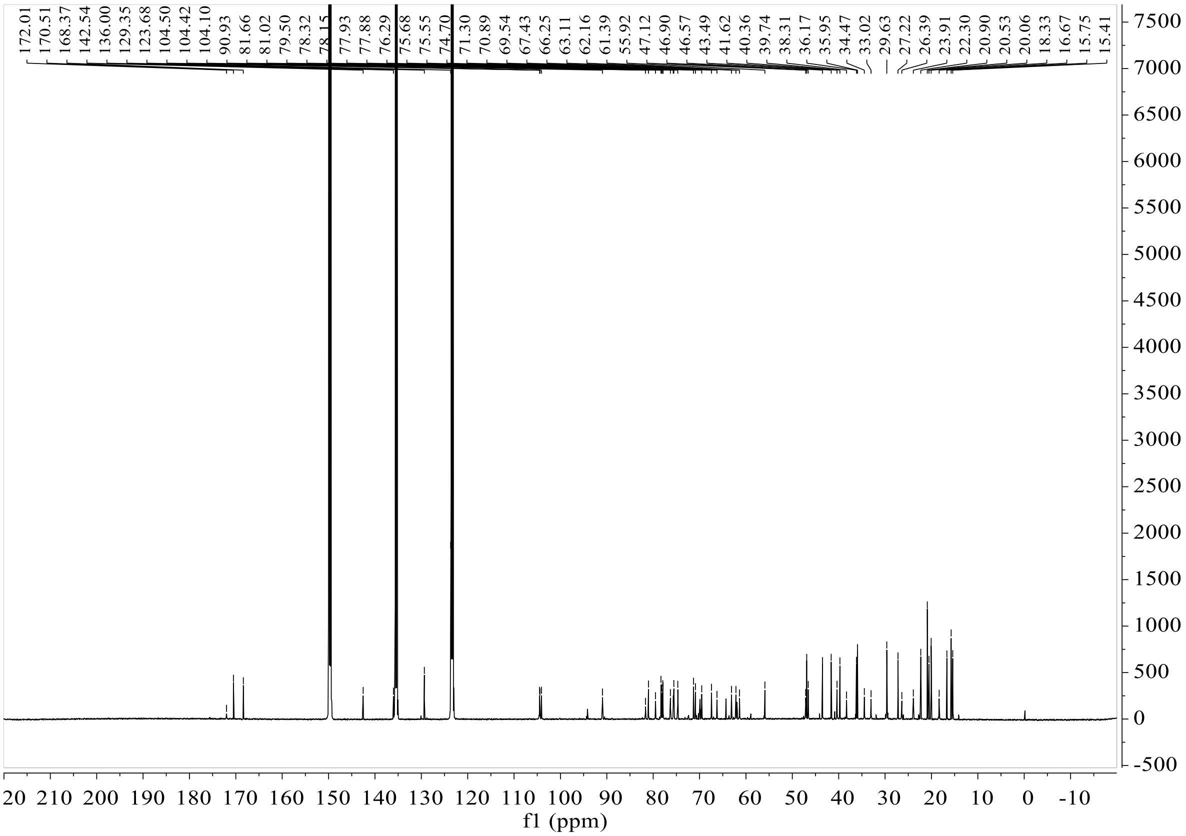

S117. ^{13}C NMR Spectrum (150 MHZ, Pyridine-d_5) of the new compound **20**

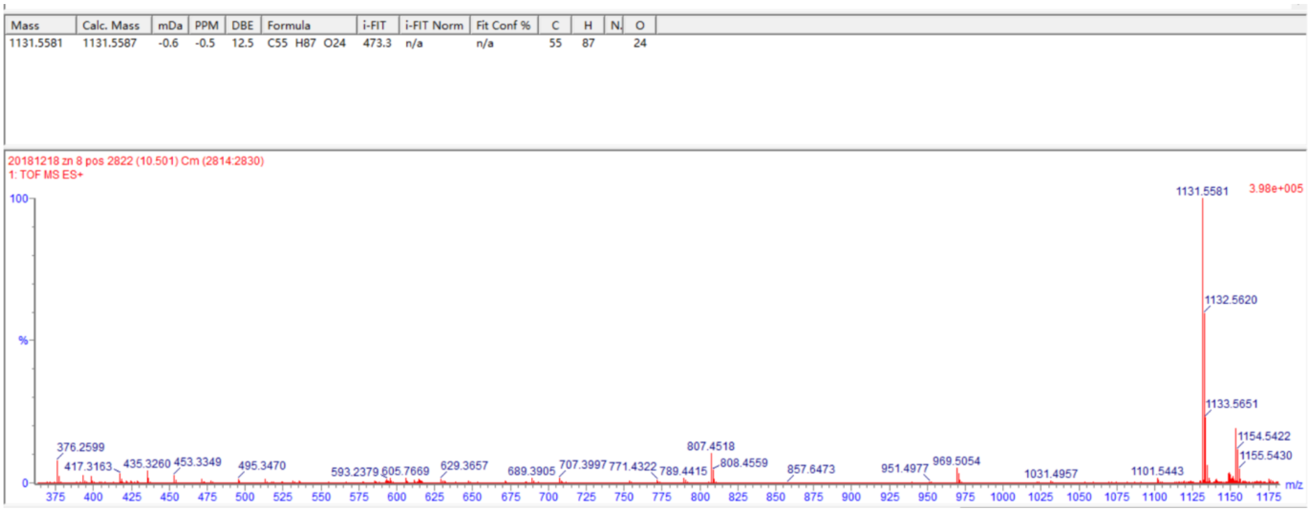

Mass	Calc. Mass	mDa	PPM	DBE	Formula	i-FIT	i-FIT Norm	Fit Conf %	C	H	N	O
1131.5581	1131.5587	-0.6	-0.5	12.5	C55 H87 O24	473.3	n/a	n/a	55	87		24

S118. HRESIMS spectrum of the new compound **20**

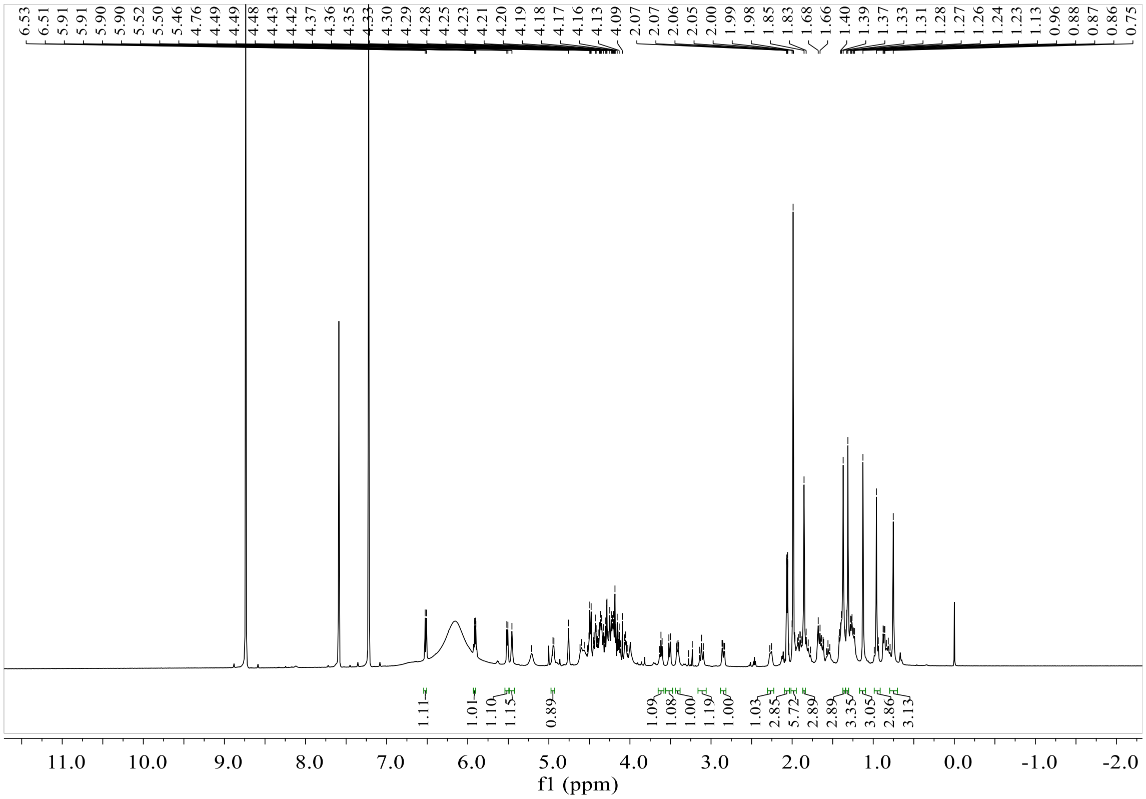

S119. ^{1}H NMR Spectrum (600 MHZ, Pyridine-d_5) of the new compound **21**

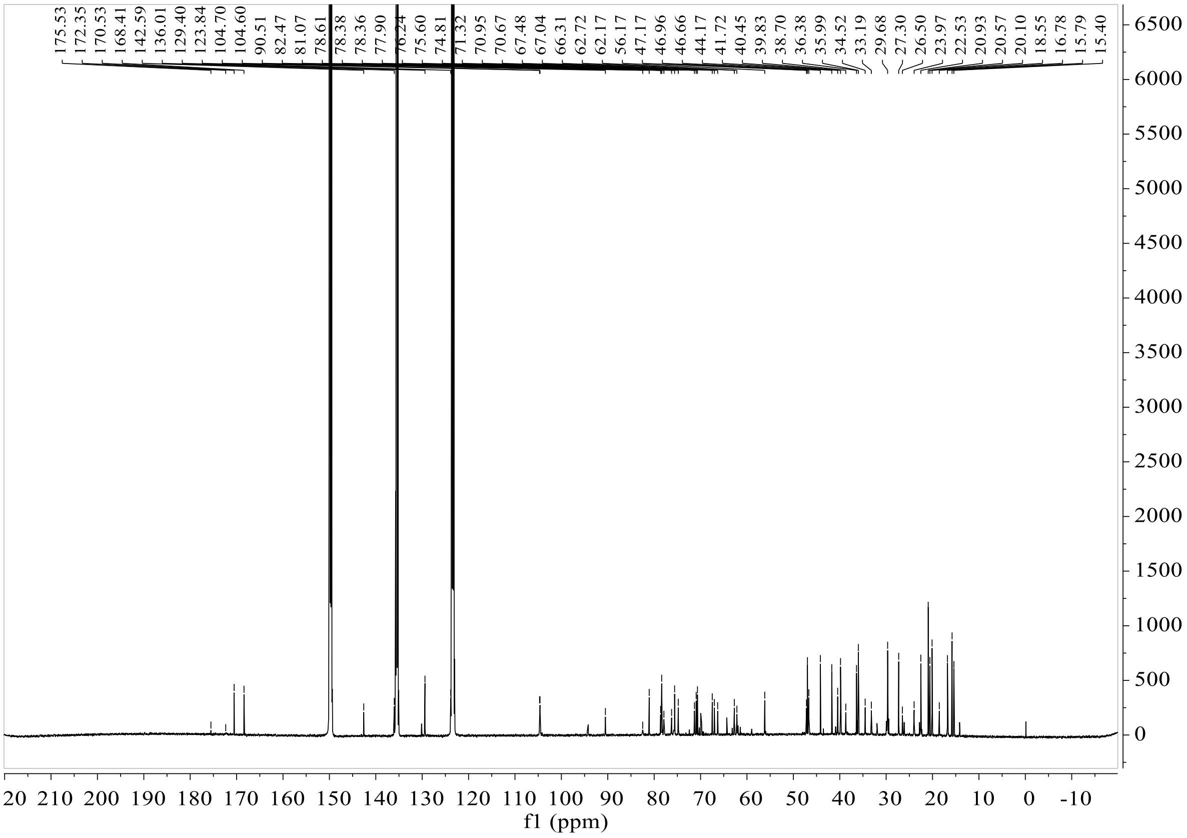

S120. ^{13}C NMR Spectrum (150 MHZ, Pyridine-d_5) of the new compound **21**

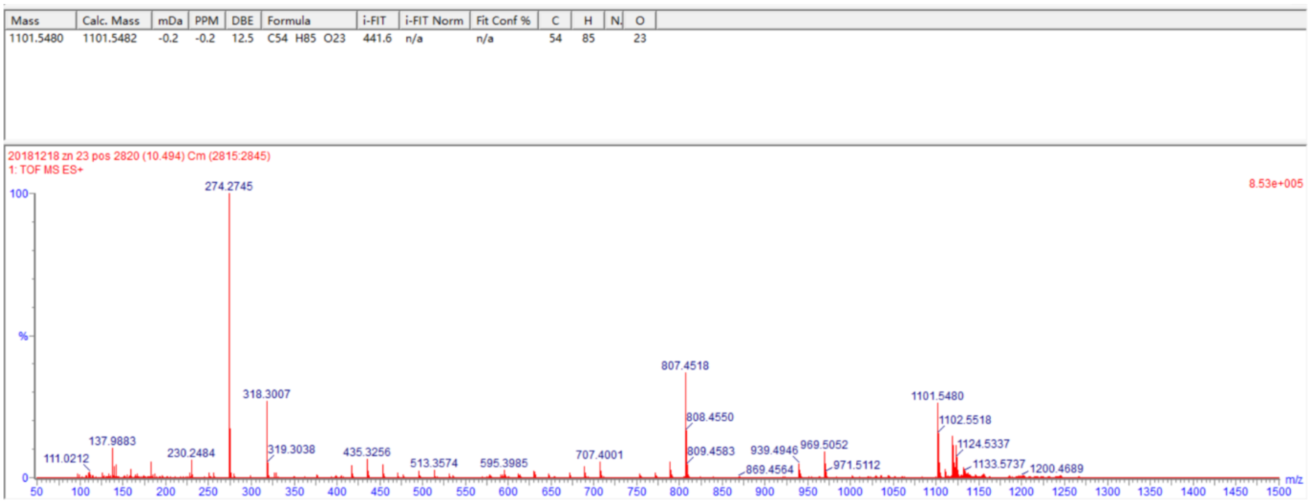

S121. HRESIMS spectrum of the new compound **21**

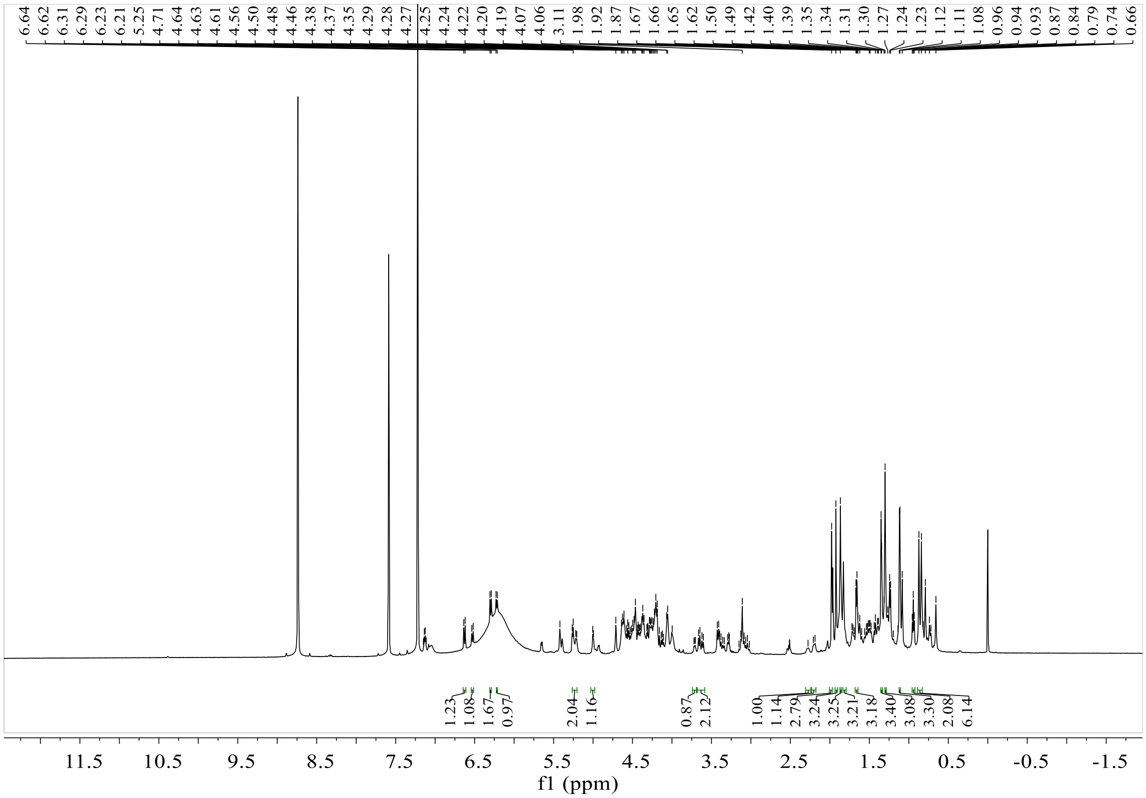

S122. ^{1}H NMR Spectrum (600 MHZ, Pyridine-d_5) of the new compound **22**

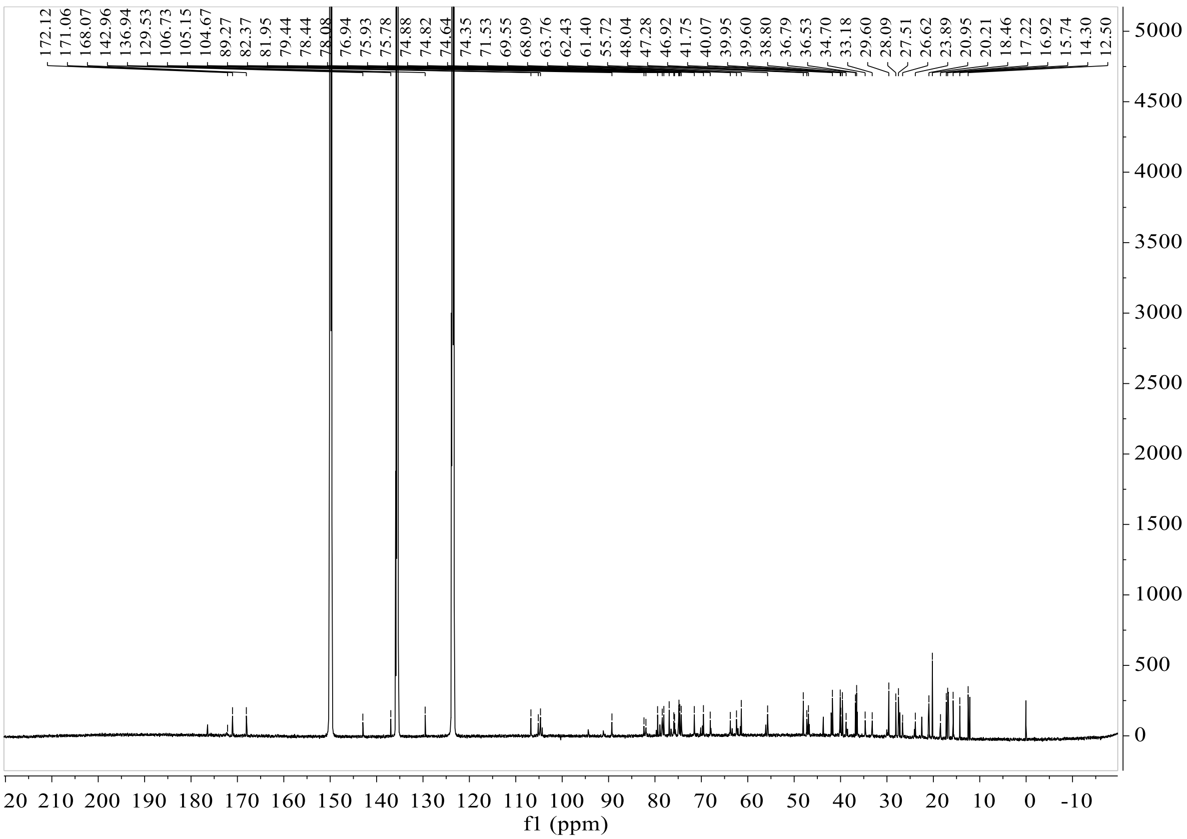

S123. ^{13}C NMR Spectrum (150 MHZ, Pyridine-d_5) of the new compound **22**

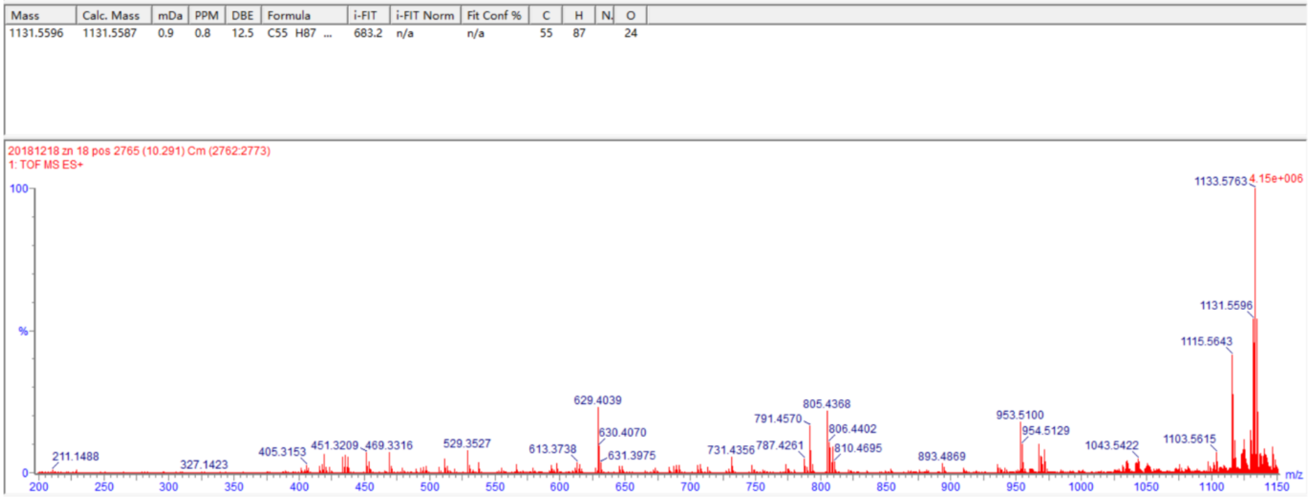

Mass	Calc. Mass	mDa	PPM	DBE	Formula	i-FIT	i-FIT Norm	Fit Conf %	C	H	N	O
1131.5596	1131.5587	0.9	0.8	12.5	C55 H87 ...	683.2	n/a	n/a	55	87		24

S124. HRESIMS spectrum of the new compound **22**

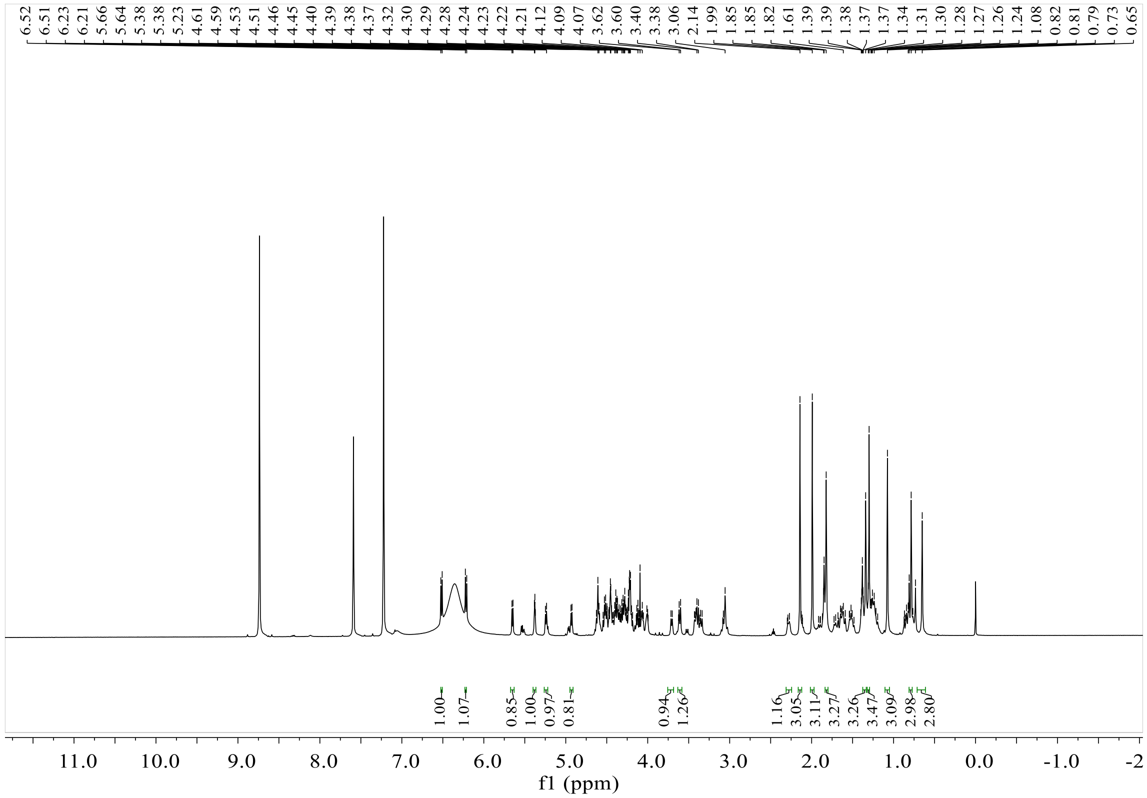

S125. ^{1}H NMR Spectrum (600 MHZ, Pyridine-d_5) of the new compound **23**

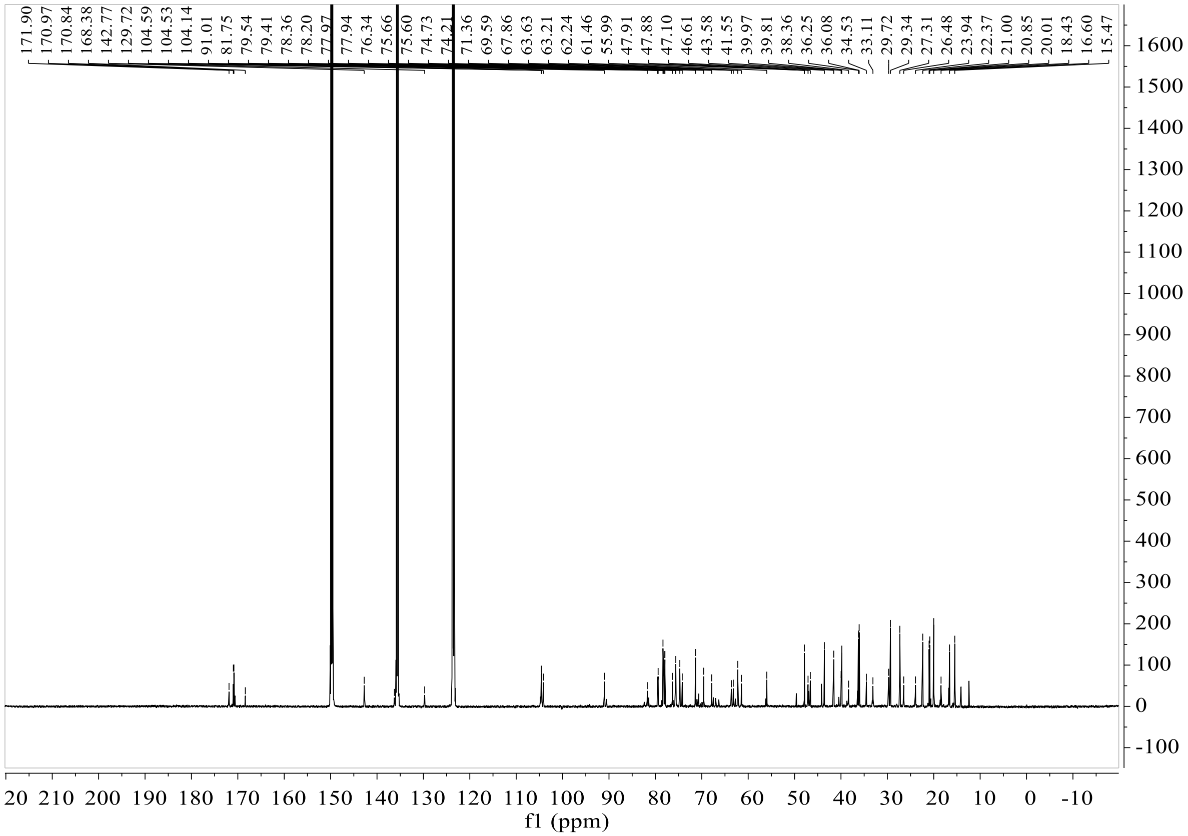

S126. ^{13}C NMR Spectrum (150 MHZ, Pyridine-d_5) of the new compound **23**

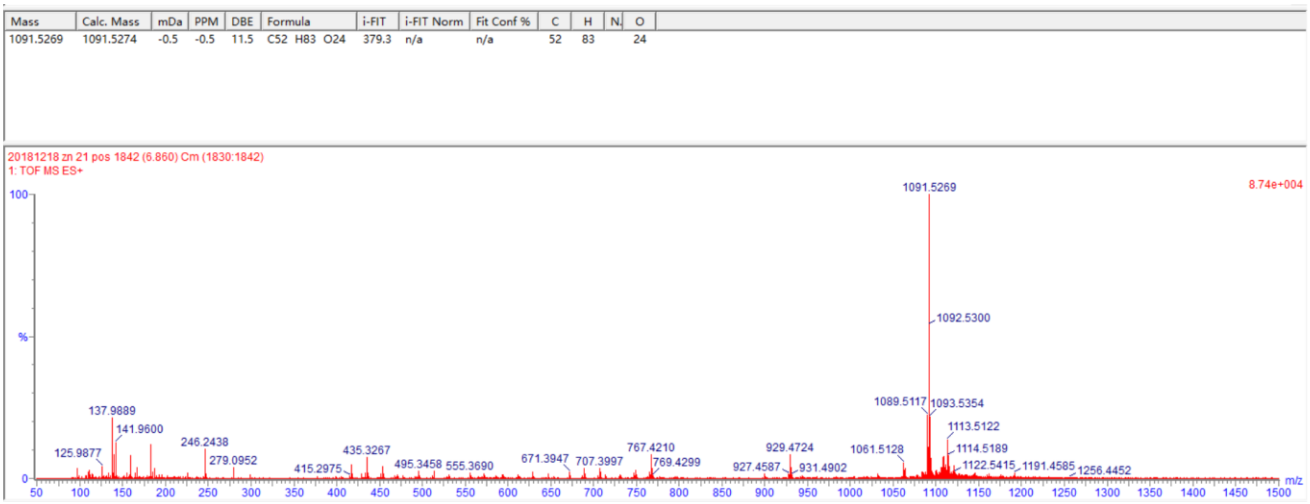

Mass	Calc. Mass	mDa	PPM	DBE	Formula	i-FIT	i-FIT Norm	Fit Conf %	C	H	N	O
1091.5269	1091.5274	-0.5	-0.5	11.5	C52 H83 O24	379.3	n/a	n/a	52	83		24

S127. HRESIMS spectrum of the new compound **23**

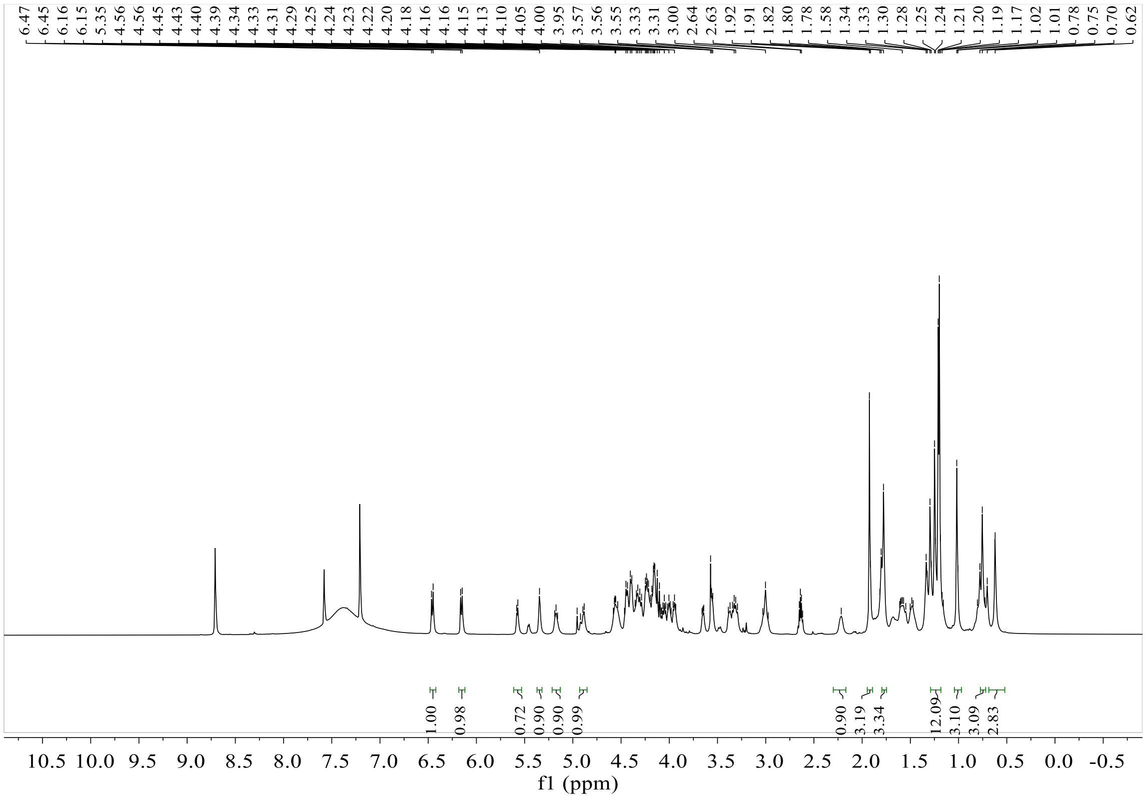

S128. ^{1}H NMR Spectrum (600 MHZ, Pyridine-d_5) of the new compound **24**

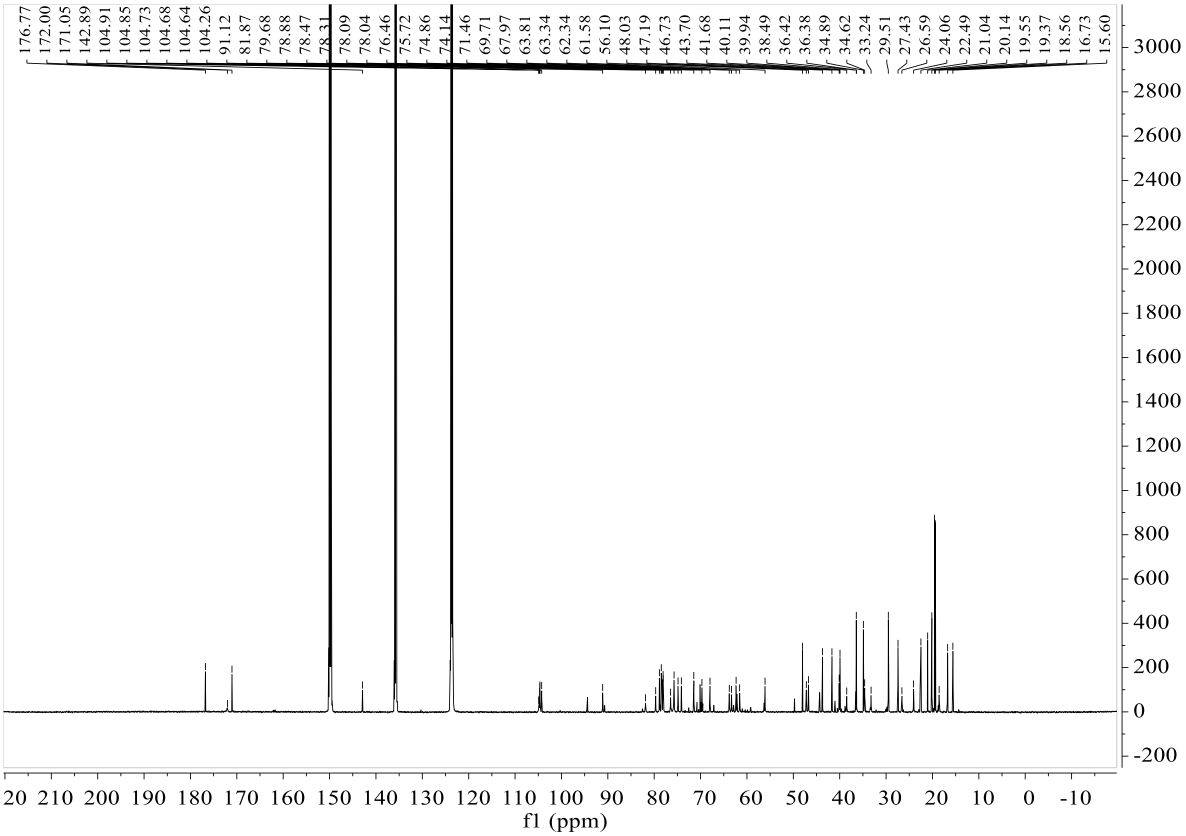

S129. ^{13}C NMR Spectrum (150 MHZ, Pyridine-d_5) of the new compound **24**

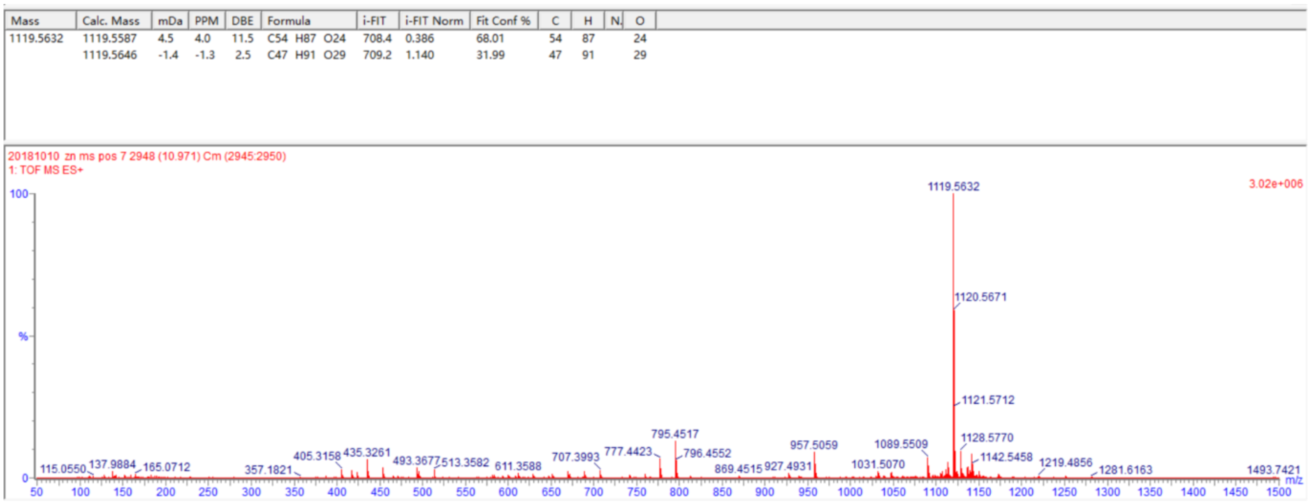

Mass	Calc. Mass	mDa	PPM	DBE	Formula	i-FIT	i-FIT Norm	Fit Conf %	C	H	N	O
1119.5632	1119.5587	4.5	4.0	11.5	C54 H87 O24	708.4	0.386	68.01	54	87		24
	1119.5646	-1.4	-1.3	2.5	C47 H91 O29	709.2	1.140	31.99	47	91		29

S130. HRESIMS spectrum of the new compound **24**

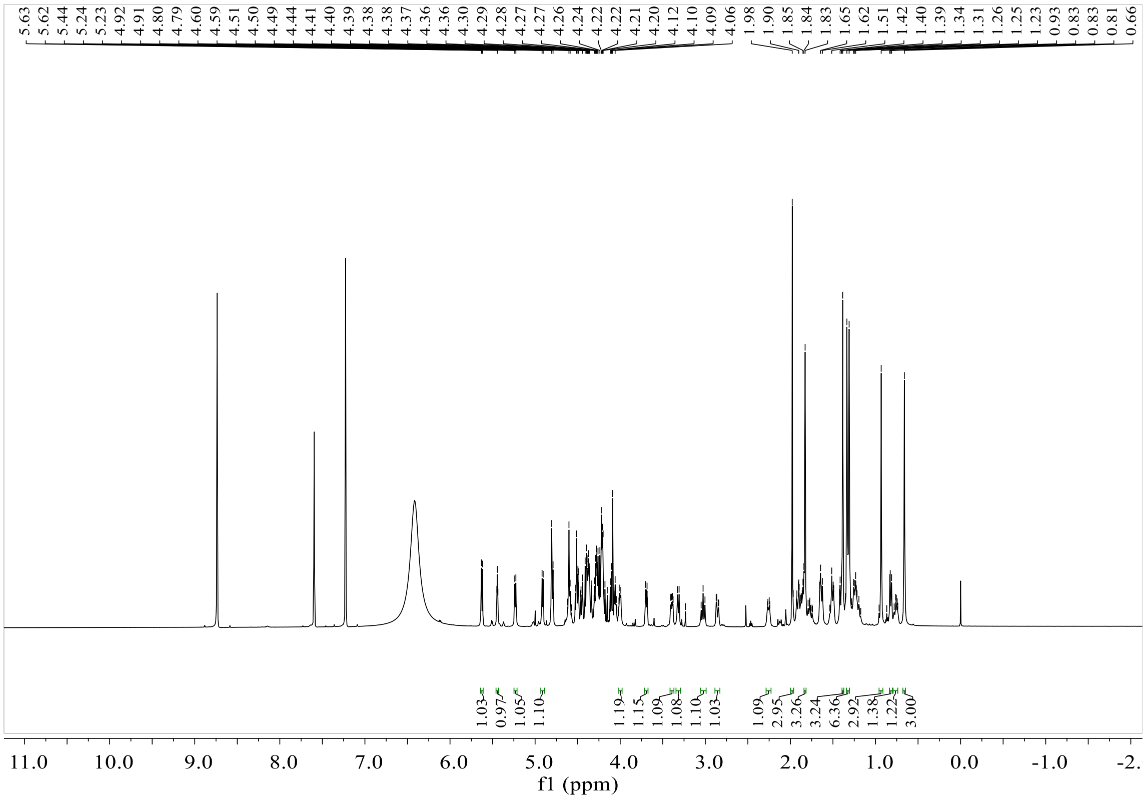

S131. ^{1}H NMR Spectrum (600 MHZ, Pyridine-d_5) of the new compound **25**

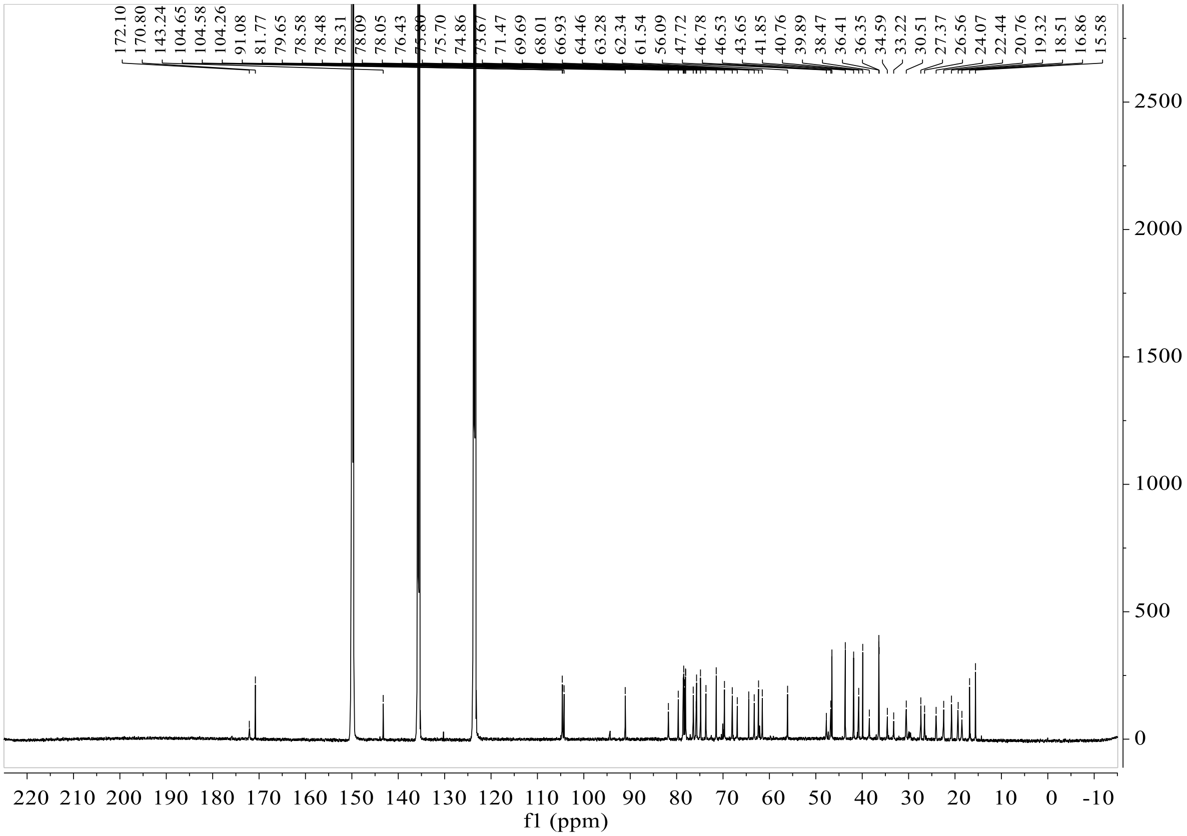

S132. ^{13}C NMR Spectrum (150 MHZ, Pyridine-d_5) of the new compound **25**

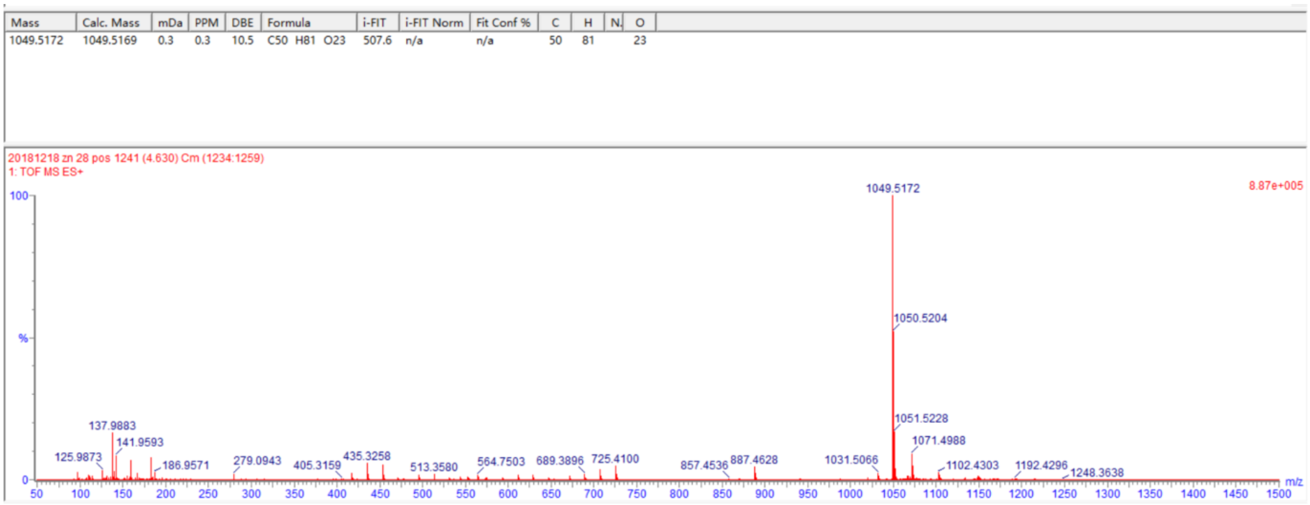

S133. HRESIMS spectrum of the new compound **25**

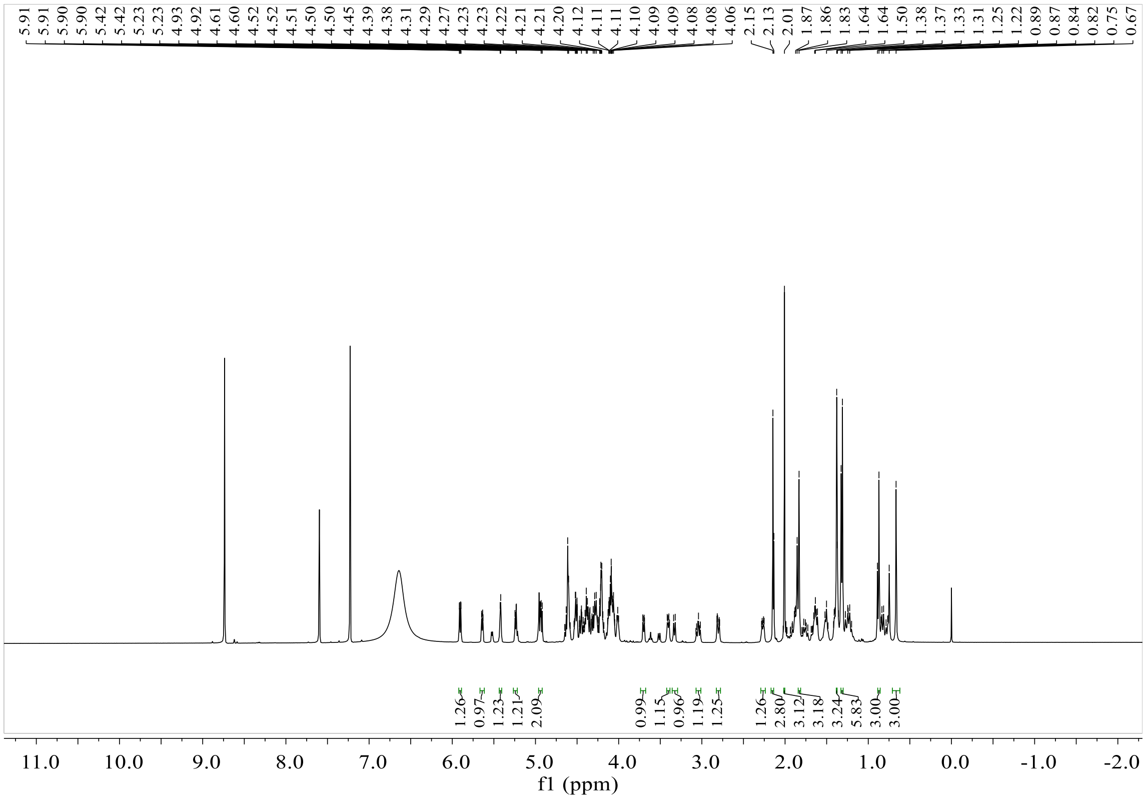

S134. ^{1}H NMR Spectrum (600 MHZ, Pyridine-d_5) of the new compound **26**

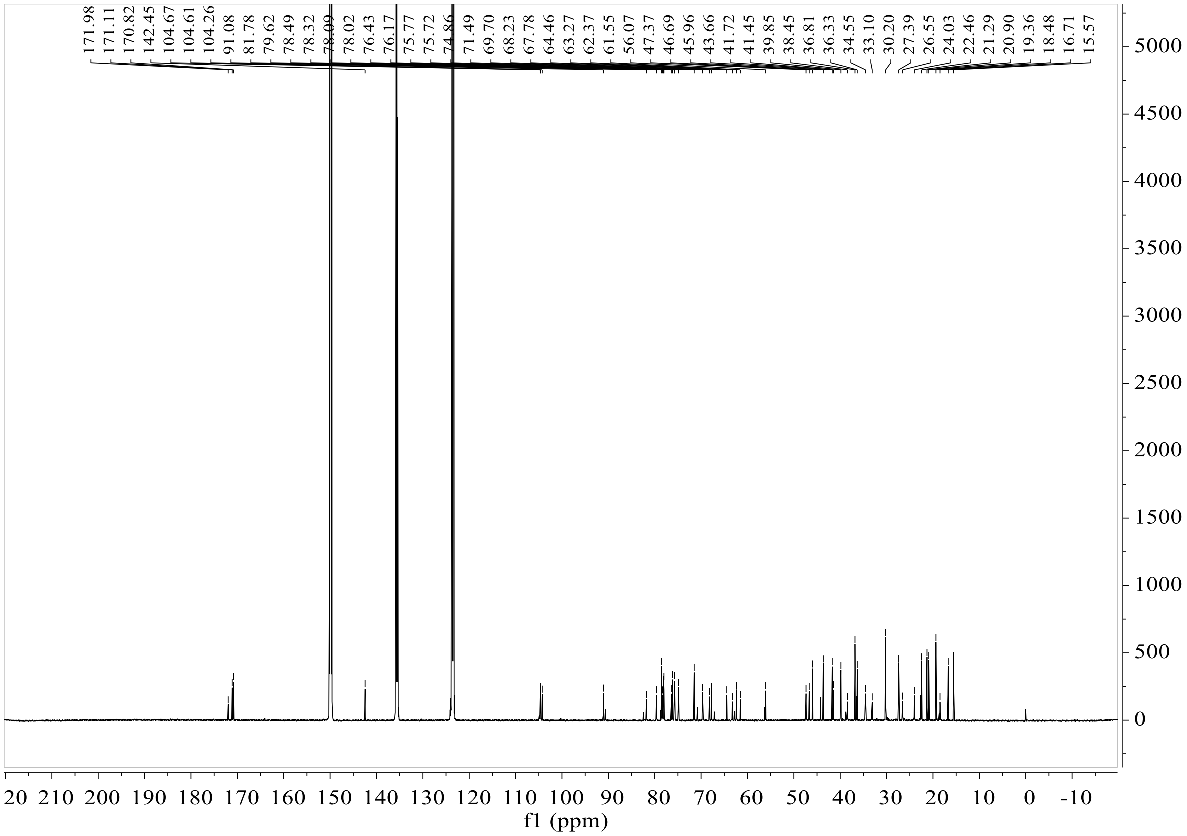

S135. ^{13}C NMR Spectrum (150 MHZ, Pyridine-d_5) of the new compound **26**

Mass	Calc. Mass	mDa	PPM	DBE	Formula	i-FIT	i-FIT Norm	Fit Conf %	C	H	N	O
1091.5289	1091.5274	1.5	1.4	11.5	C52 H83 O24	667.1	0.001	99.93	52	83		24
	1091.5333	-4.4	-4.0	2.5	C45 H87 O29	674.4	7.222	0.07	45	87		29

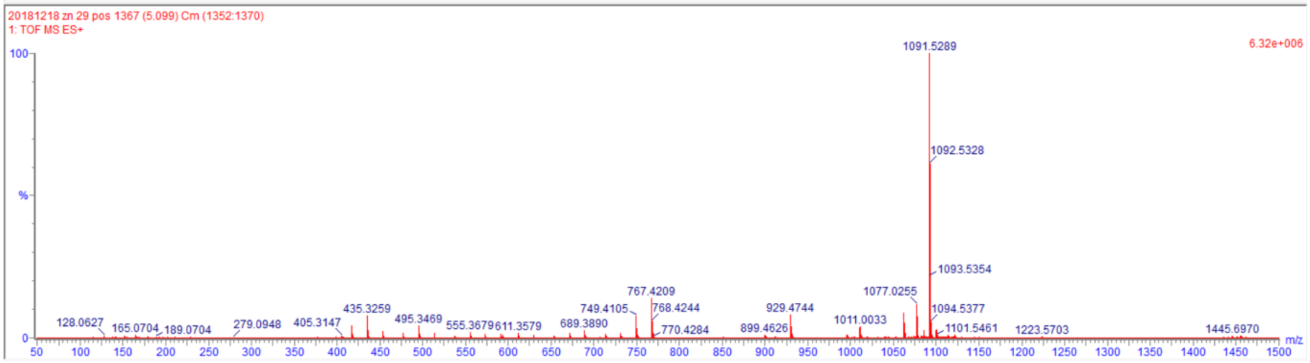

S136. HRESIMS spectrum of the new compound **26**

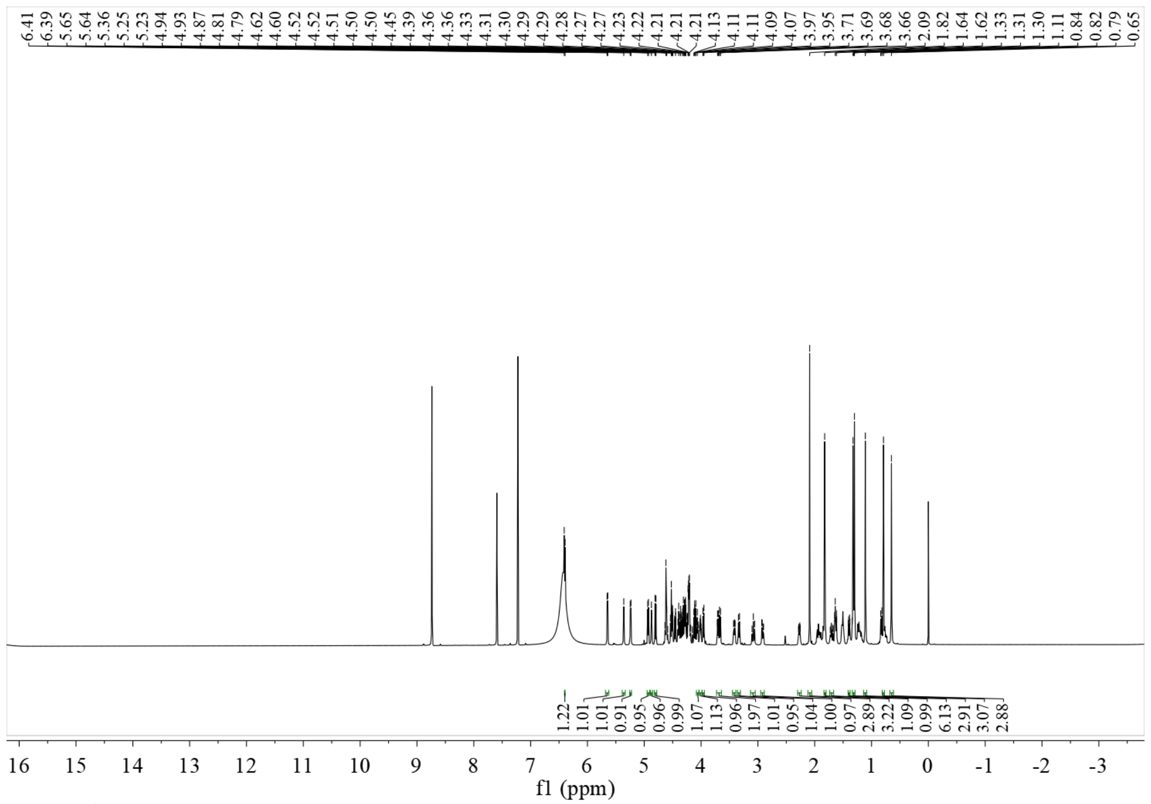

S137. ^{1}H NMR Spectrum (600 MHZ, Pyridine-d_5) of the new compound **27**

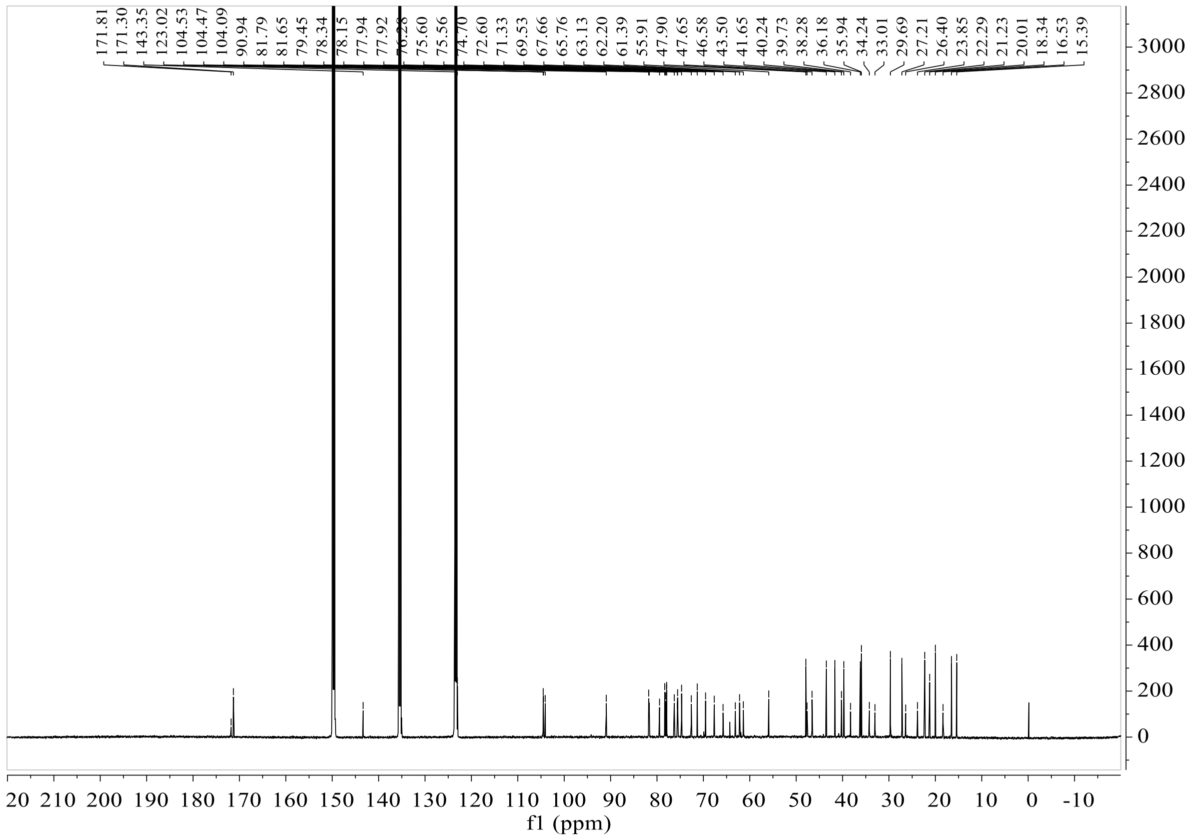

S138. ^{13}C NMR Spectrum (150 MHZ, Pyridine-d_5) of the new compound **27**

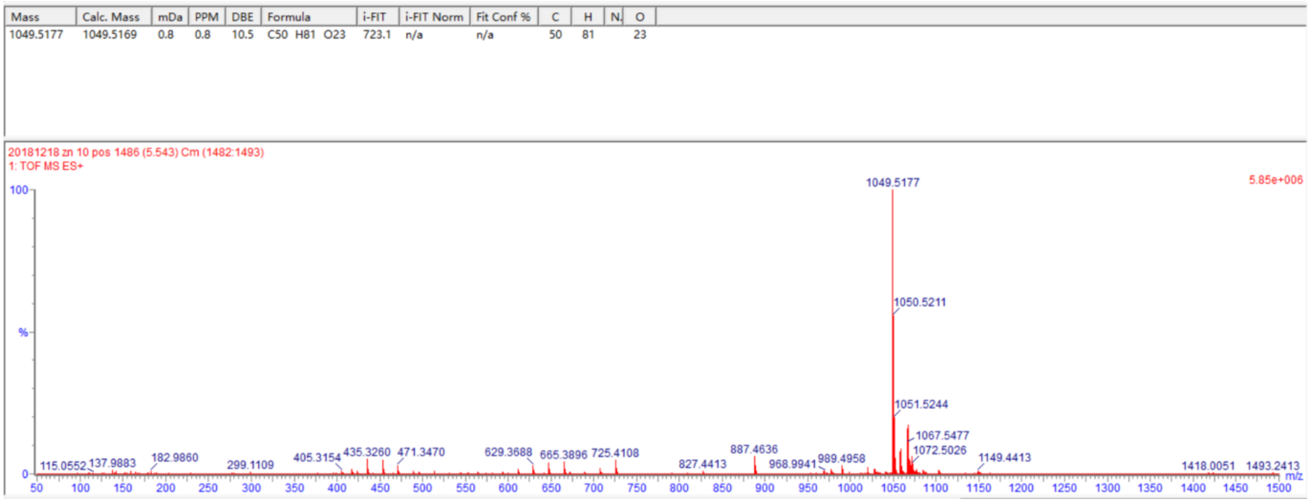

S139. HRESIMS spectrum of the new compound **27**

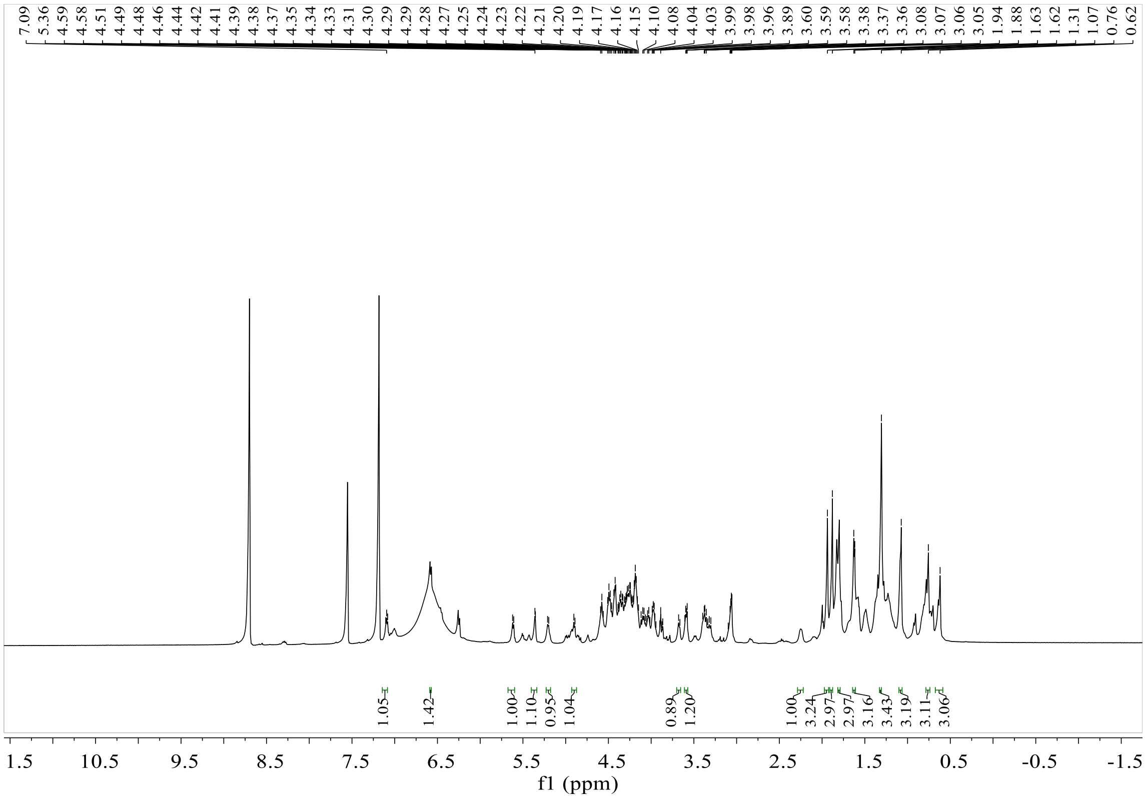

S140. ^{1}H NMR Spectrum (600 MHZ, Pyridine-d_5) of the new compound **28**

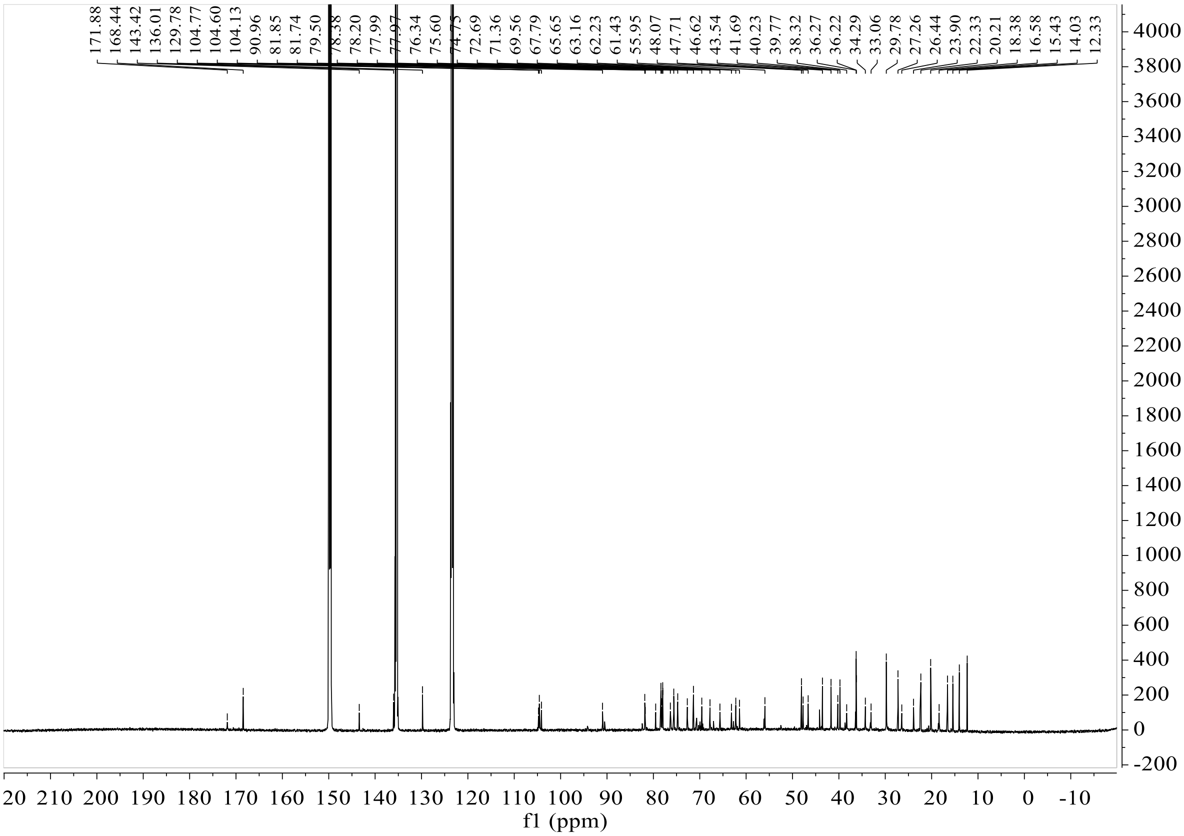

S141. ^{13}C NMR Spectrum (150 MHZ, Pyridine-d_5) of the new compound **28**

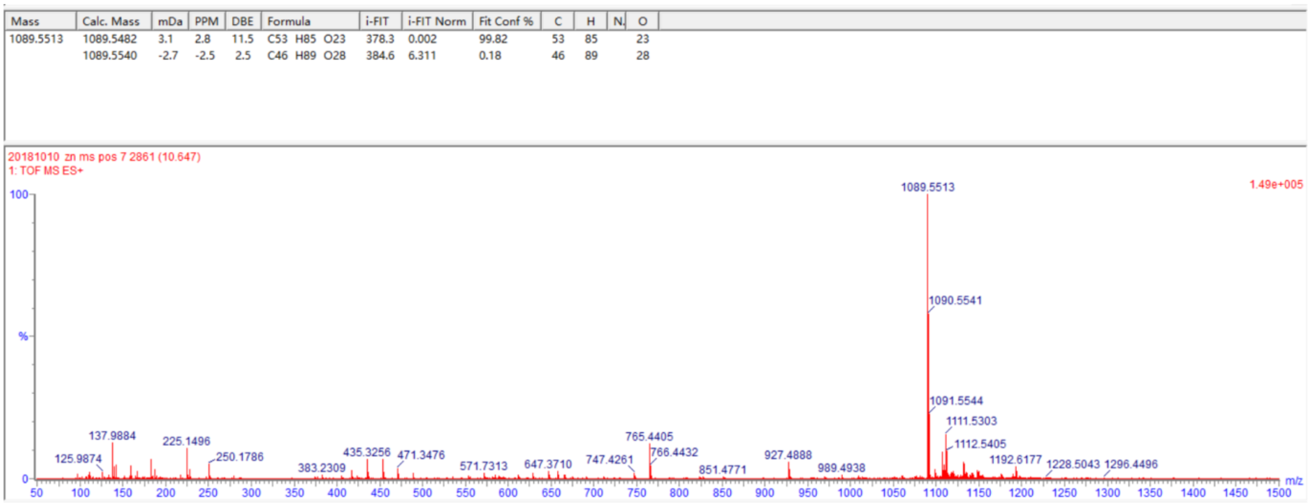

Mass	Calc. Mass	mDa	PPM	DBE	Formula	i-FIT	i-FIT Norm	Fit Conf %	C	H	N	O
1089.5513	1089.5482	3.1	2.8	11.5	C53 H85 O23	378.3	0.002	99.82	53	85		23
	1089.5540	-2.7	-2.5	2.5	C46 H89 O28	384.6	6.311	0.18	46	89		28

S142. HRESIMS spectrum of the new compound **28**

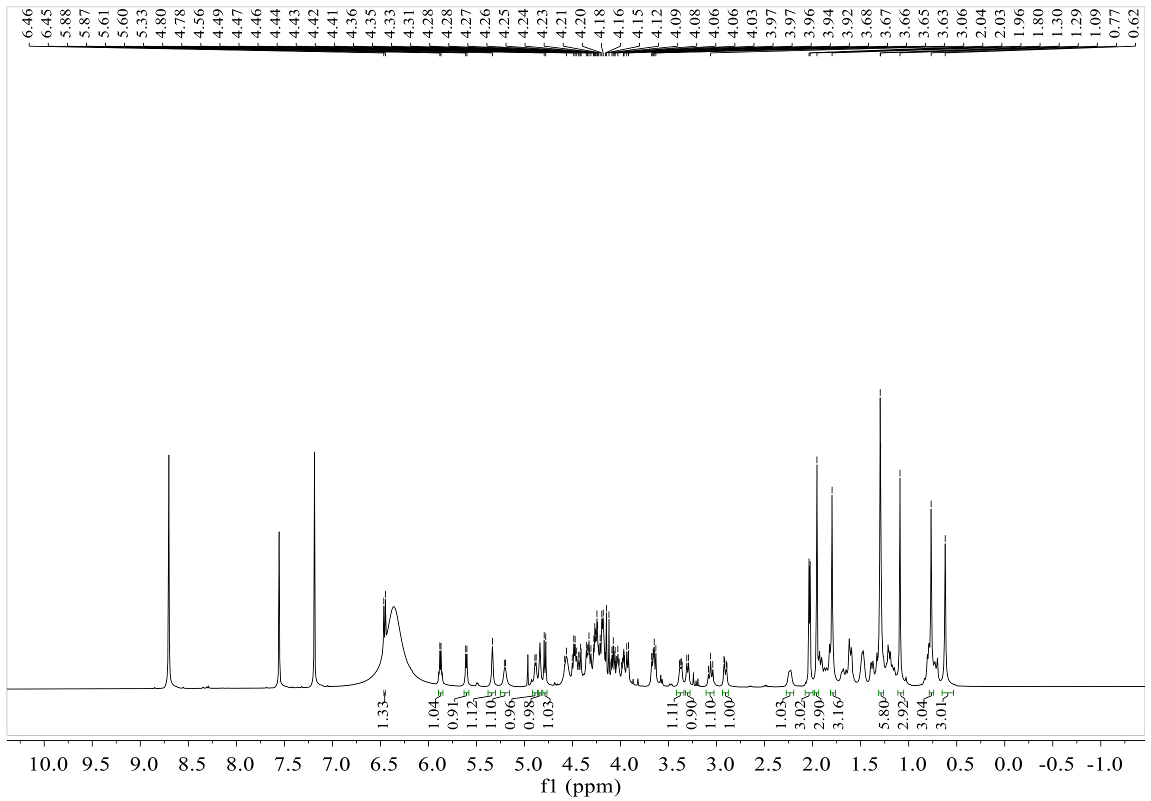

S143. ^{1}H NMR Spectrum (600 MHZ, Pyridine-d_5) of the new compound **29**

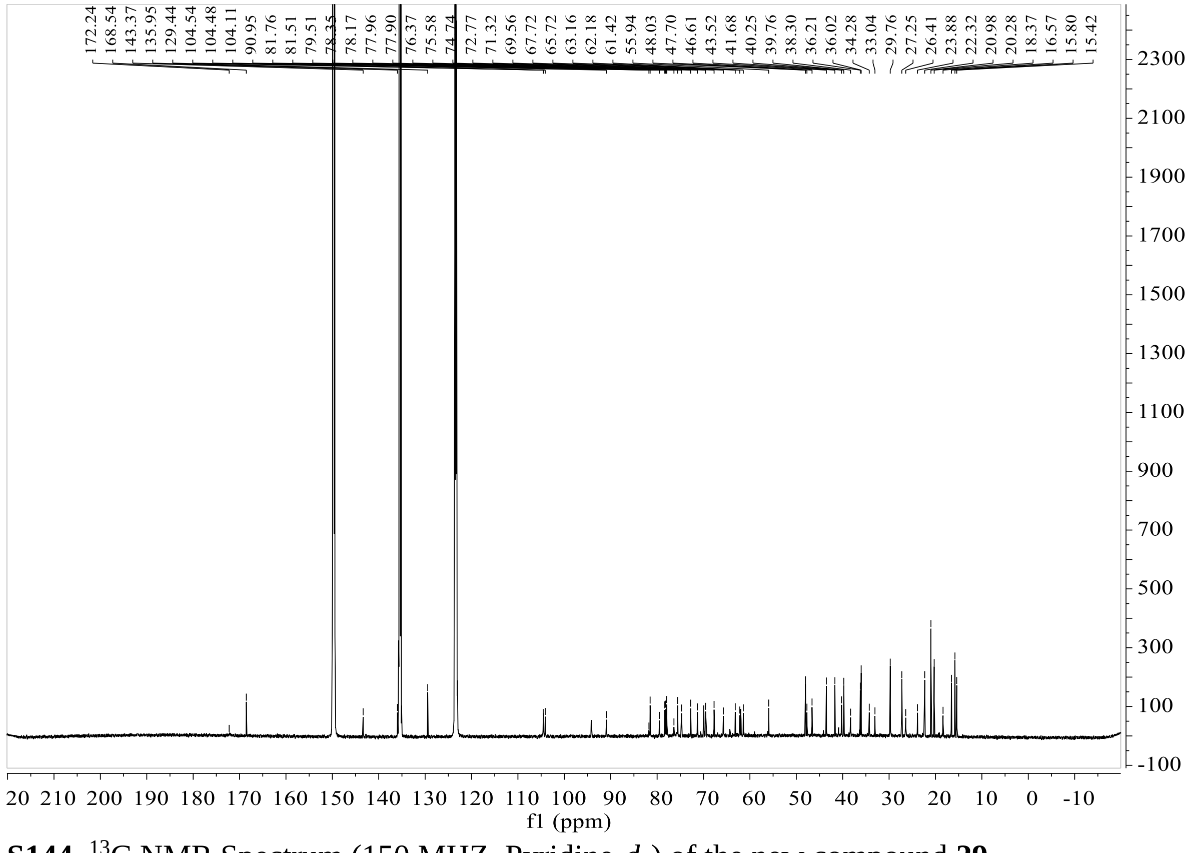

S144. ^{13}C NMR Spectrum (150 MHZ, Pyridine-d_5) of the new compound **29**

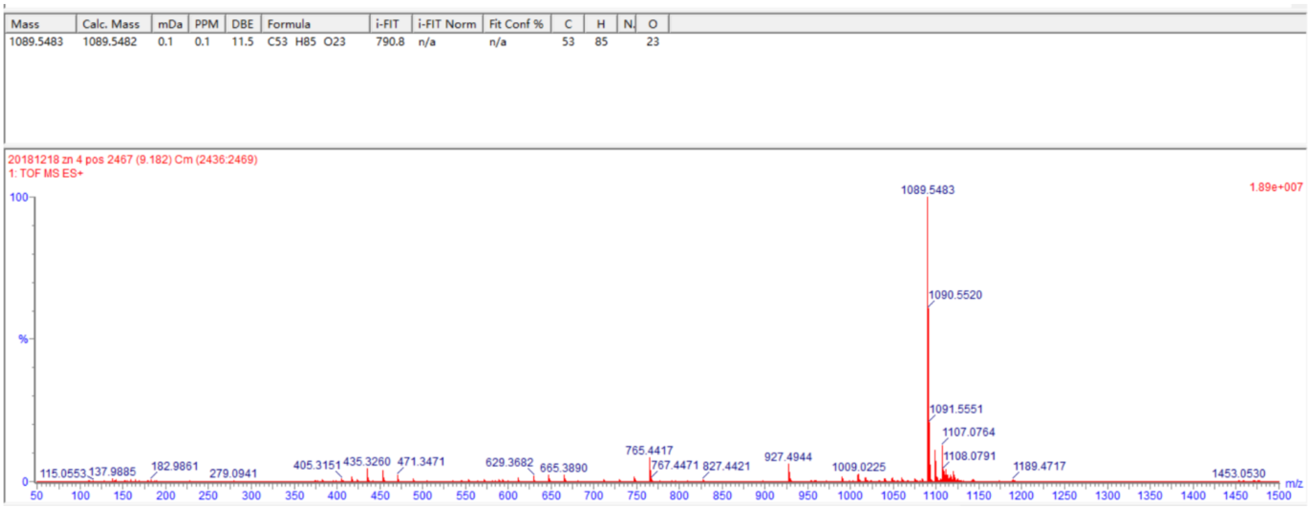

S145. HRESIMS spectrum of the new compound **29**

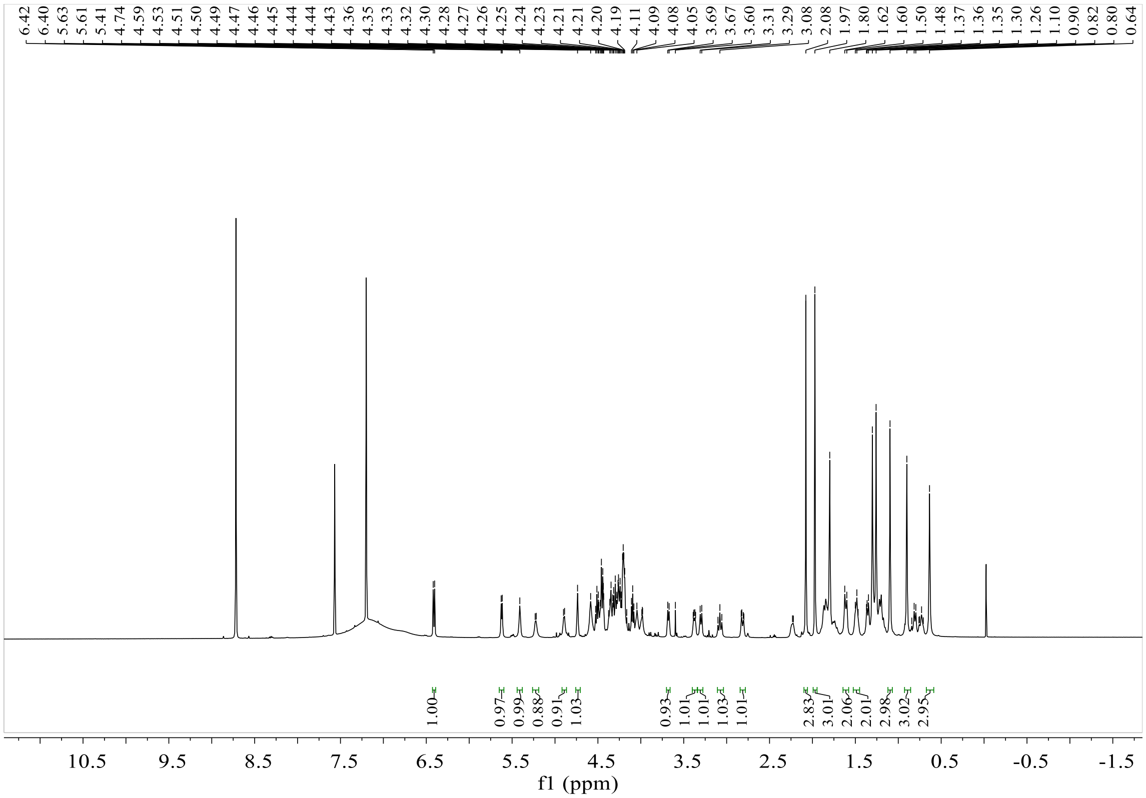

S146. ^{1}H NMR Spectrum (600 MHZ, Pyridine-d_5) of the new compound **30**

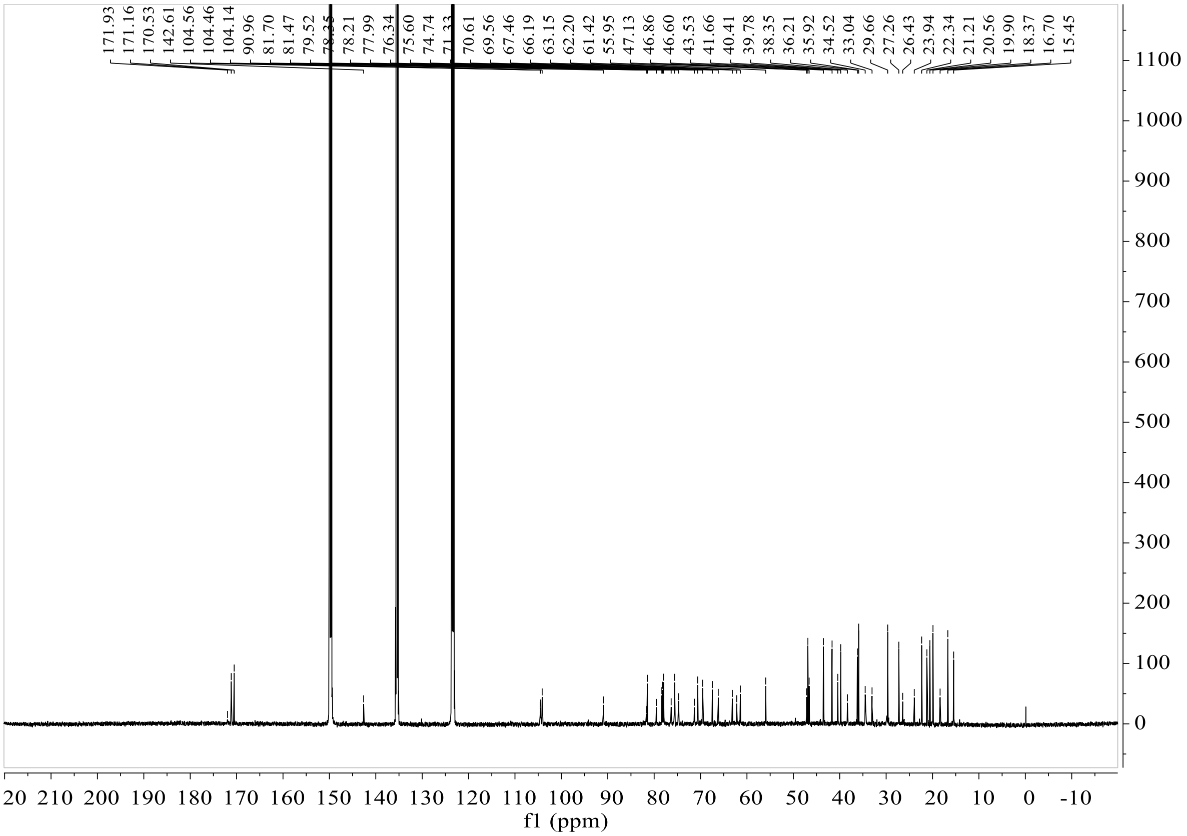

S147. ^{13}C NMR Spectrum (150 MHZ, Pyridine-d_5) of the new compound **30**

Mass	Calc. Mass	mDa	PPM	DBE	Formula	i-FIT	i-FIT Norm	Fit Conf %	C	H	N	O
1091.5306	1091.5274	3.2	2.9	11.5	C52 H83 O24	678.3	0.011	98.87	52	83		24
	1091.5333	-2.7	-2.5	2.5	C45 H87 O29	682.8	4.486	1.13	45	87		29

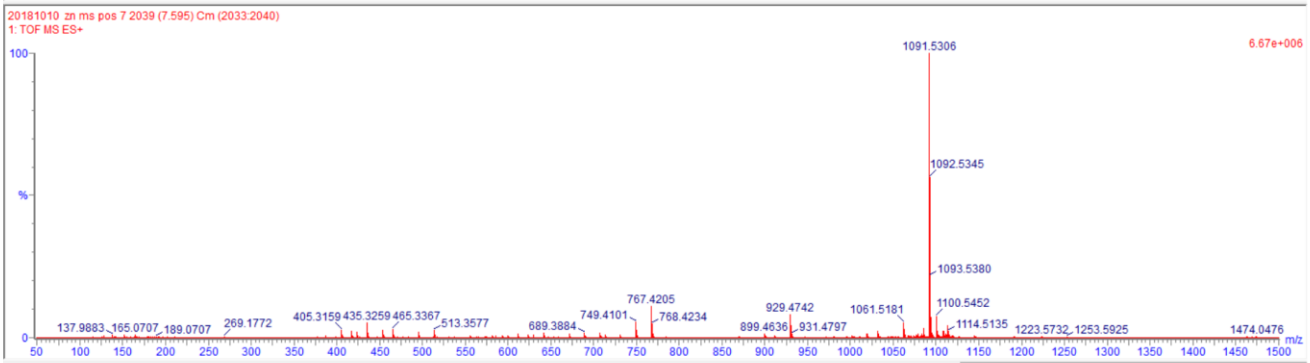

S148. HRESIMS spectrum of the new compound **30**

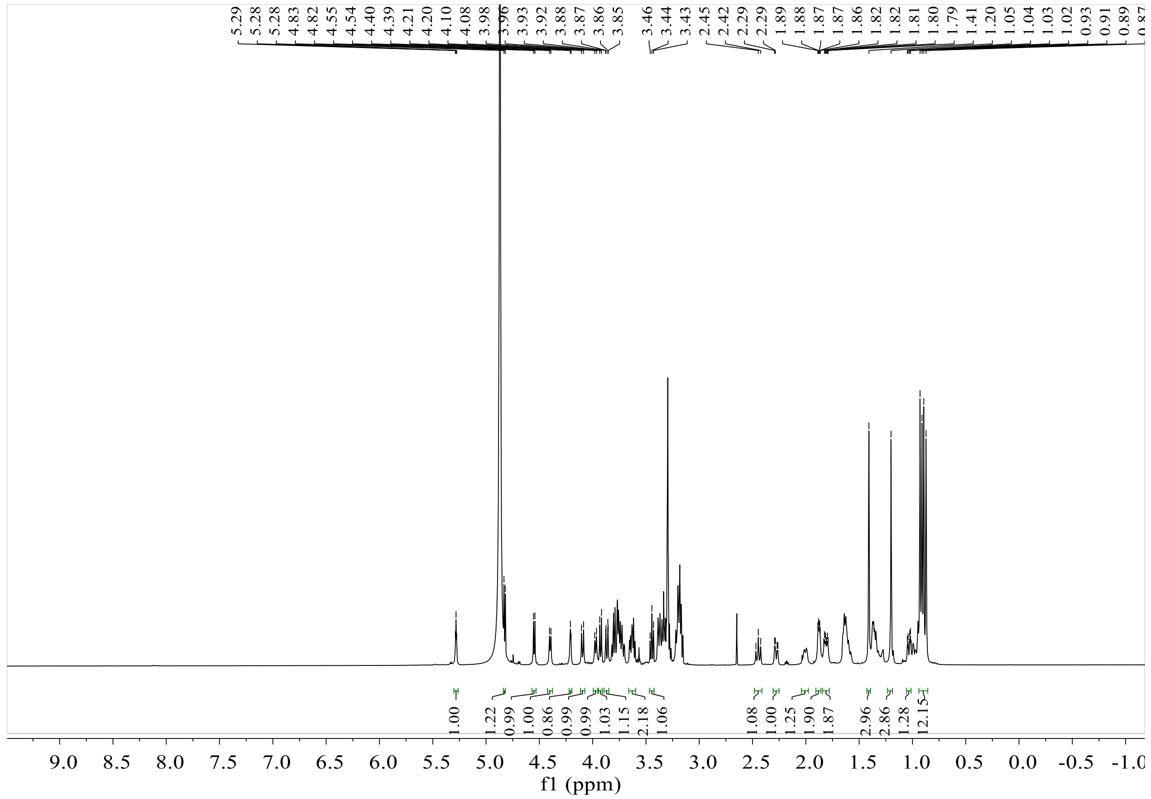

S149. ^{1}H NMR Spectrum (600 MHZ, Methanol-d_4) of the new compound **31**

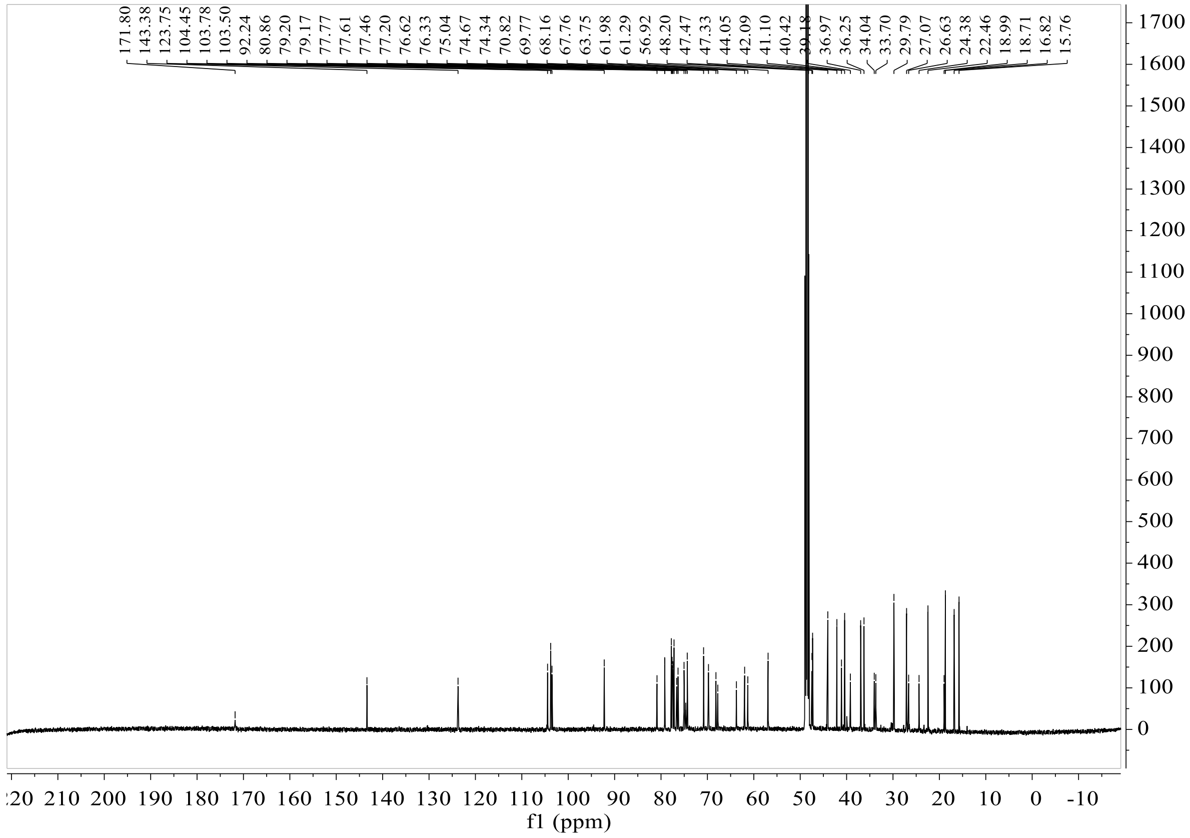

S150. ^{13}C NMR Spectrum (150 MHZ, Methanol-d_4) of the new compound **31**

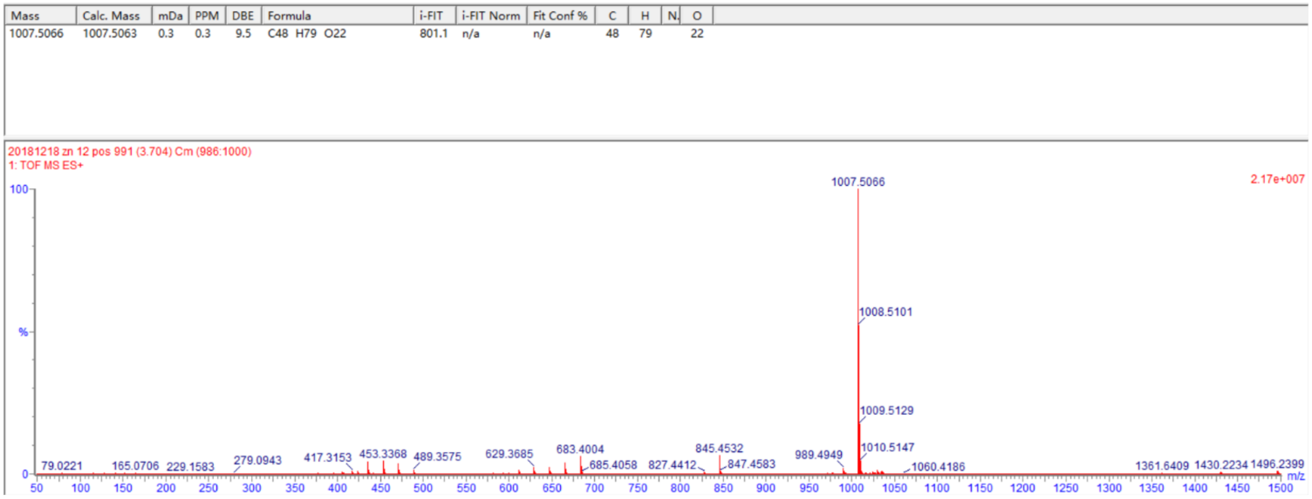

S151. HRESIMS spectrum of the new compound **31**

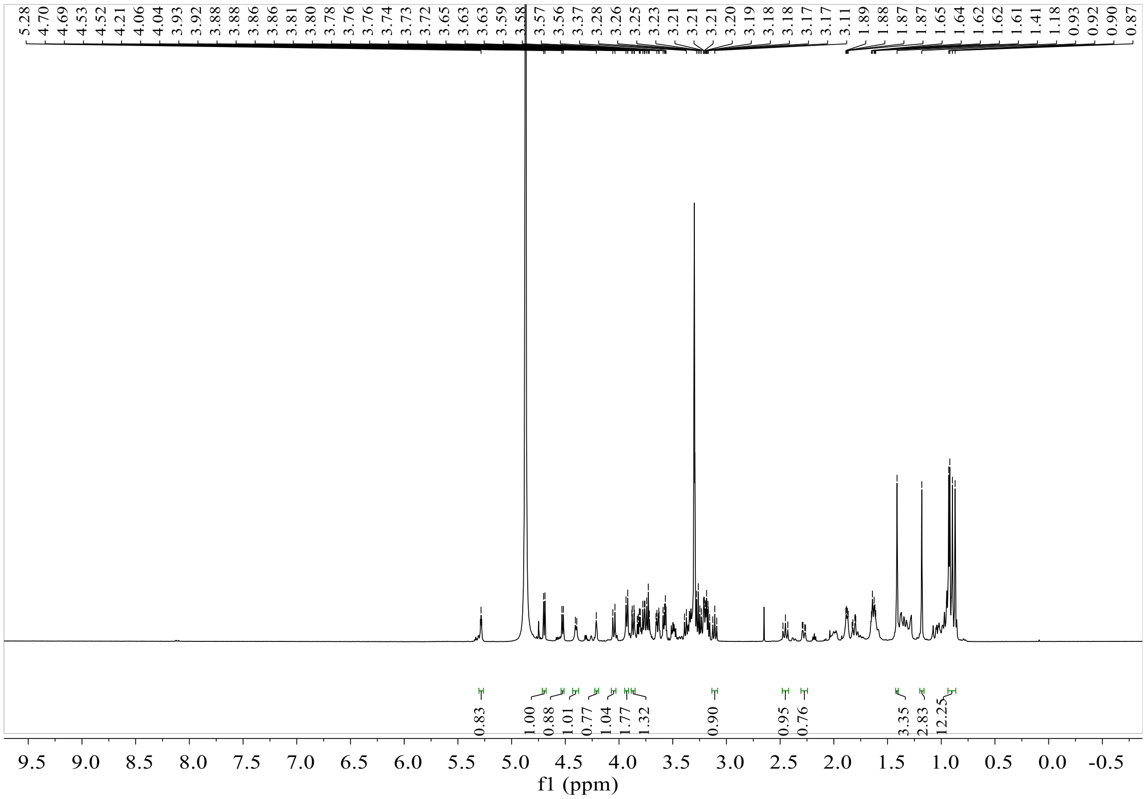

S152. ^{1}H NMR Spectrum (600 MHZ, Methanol-d_4) of the new compound **32**

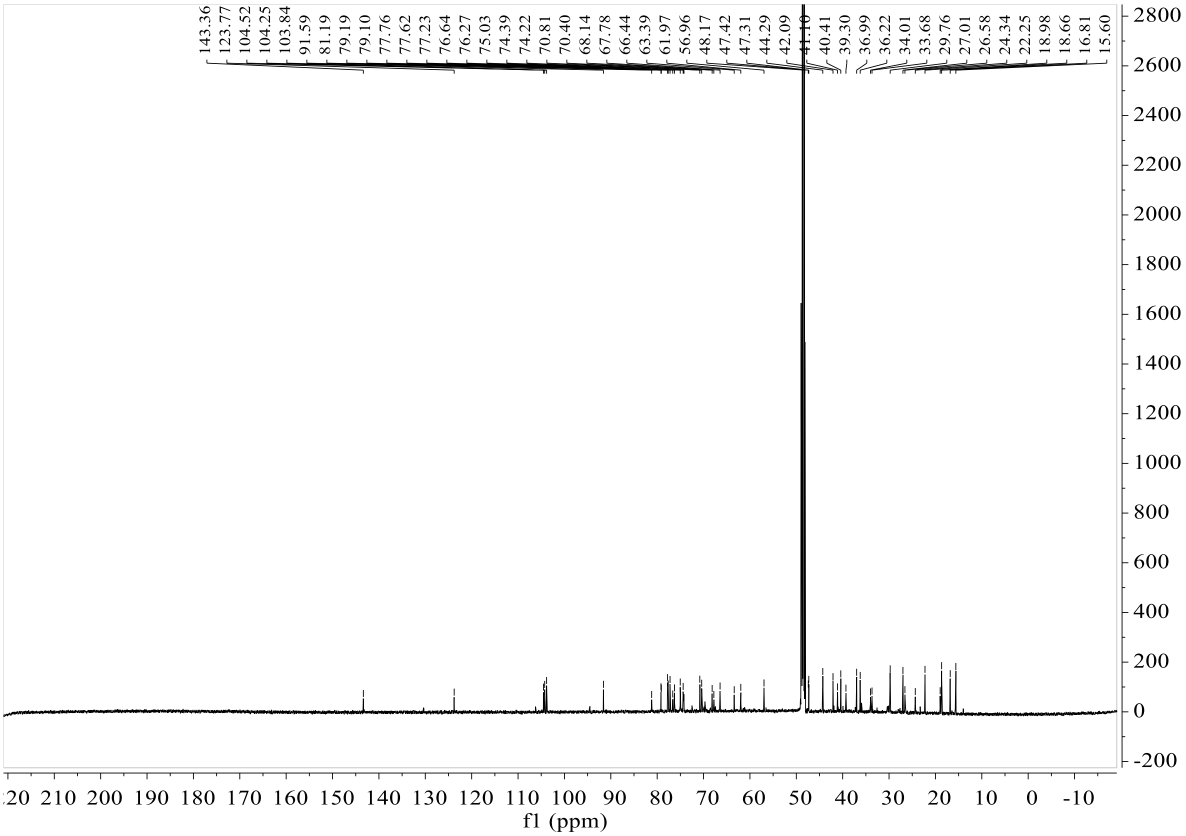

S153. ^{13}C NMR Spectrum (150 MHZ, Methanol-d_4) of the new compound **32**

Mass	Calc. Mass	mDa	PPM	DBE	Formula	i-FIT	i-FIT Norm	Fit Conf %	C	H	N	O
977.4974	977.4957	1.7	1.7	9.5	C47 H77 O21	562.5	0.127	88.07	47	77		21
	977.5016	-4.2	-4.3	0.5	C40 H81 O26	564.5	2.126	11.93	40	81		26

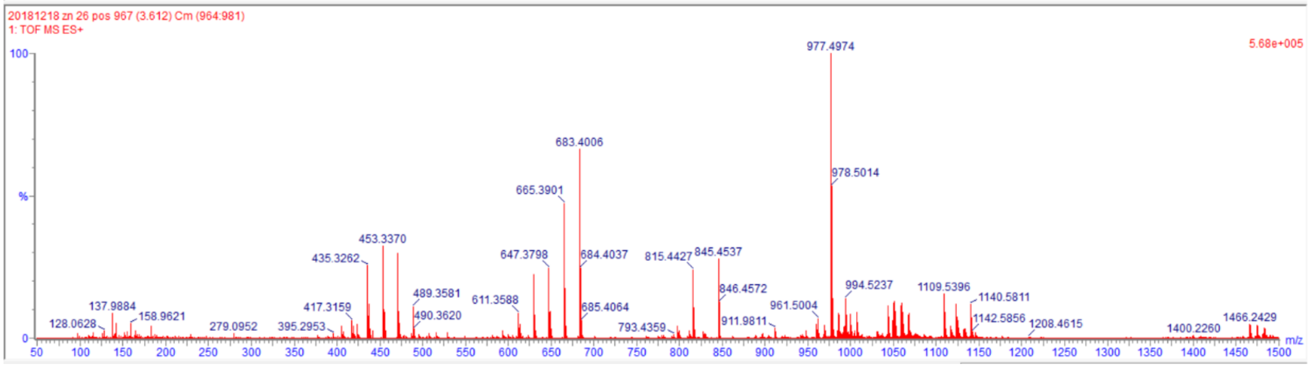

S154. HRESIMS spectrum of the new compound **32**

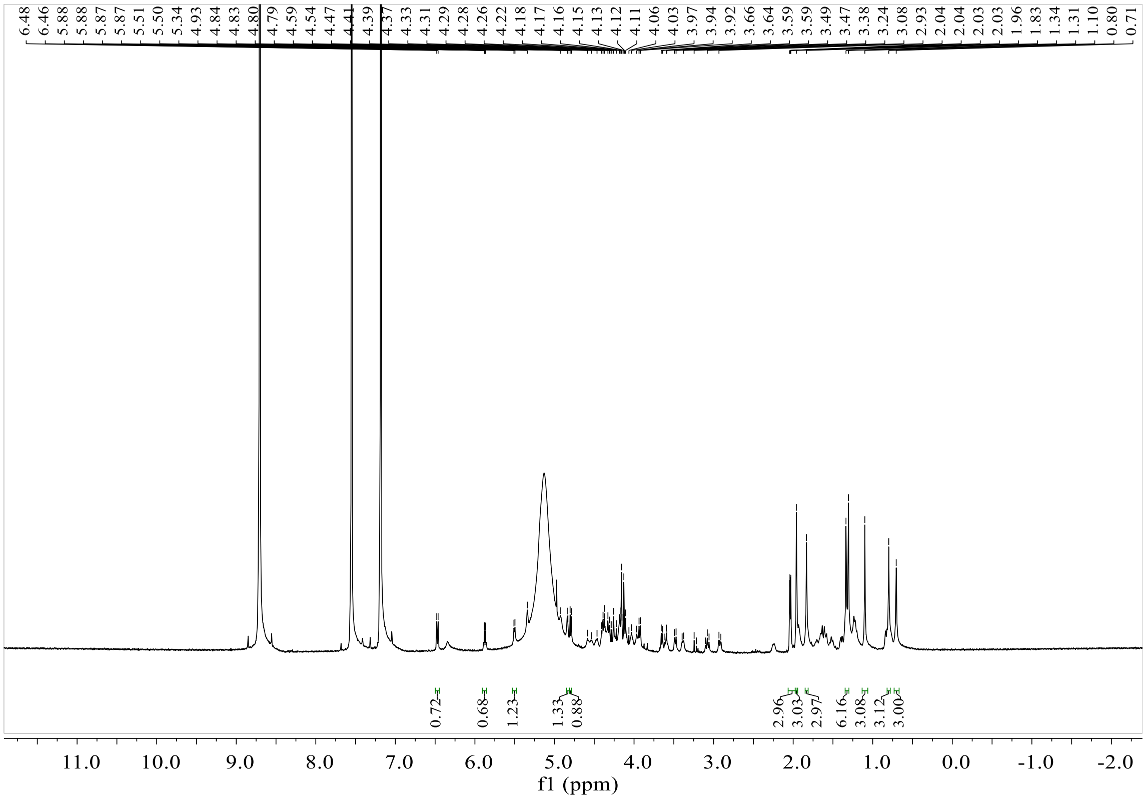

S155. ^{1}H NMR Spectrum (600 MHZ, Pyridine-d_5) of the new compound **33**

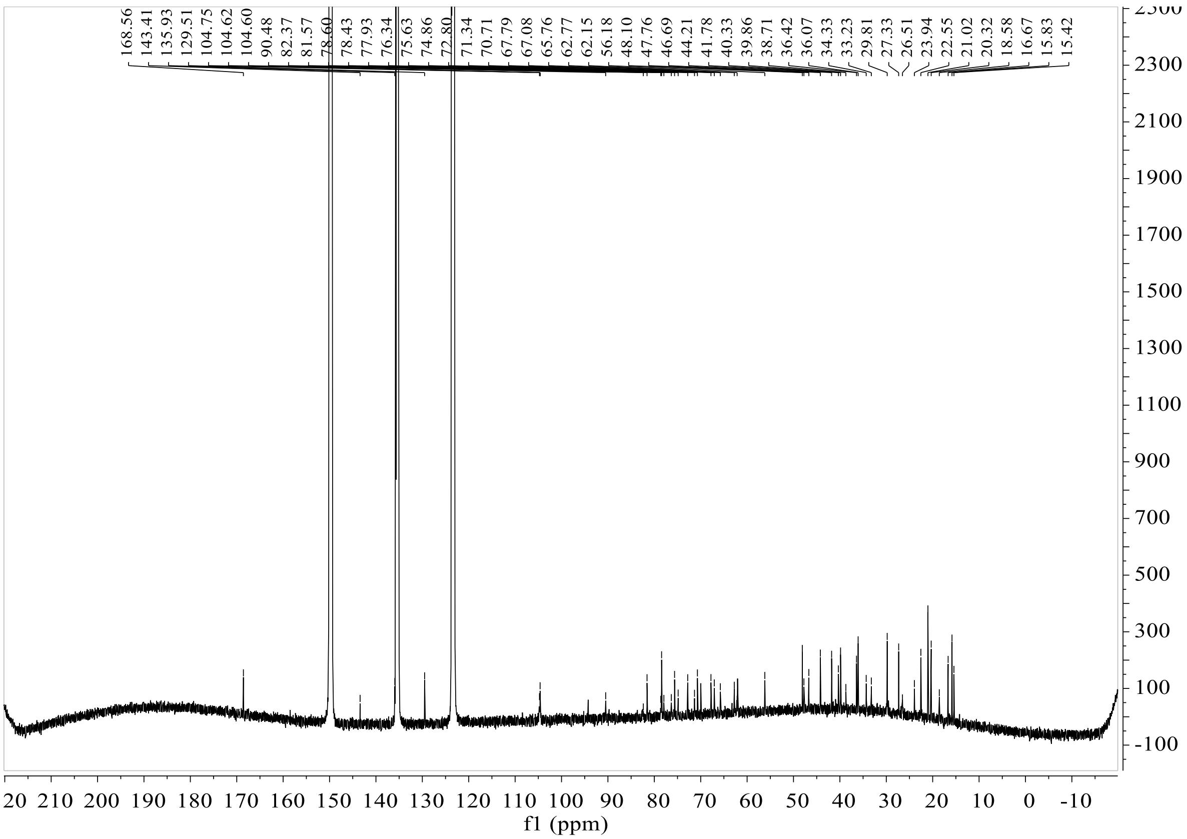

S156. ^{13}C NMR Spectrum (150 MHZ, Pyridine-d_5) of the new compound **33**

Mass	Calc. Mass	mDa	PPM	DBE	Formula	i-FIT	i-FIT Norm	Fit Conf %	C	H	N	O
1059.5391	1059.5376	1.5	1.4	11.5	C52 H83 O22	644.0	0.007	99.28	52	83		22
	1059.5435	-4.4	-4.2	2.5	C45 H87 O27	648.9	4.936	0.72	45	87		27

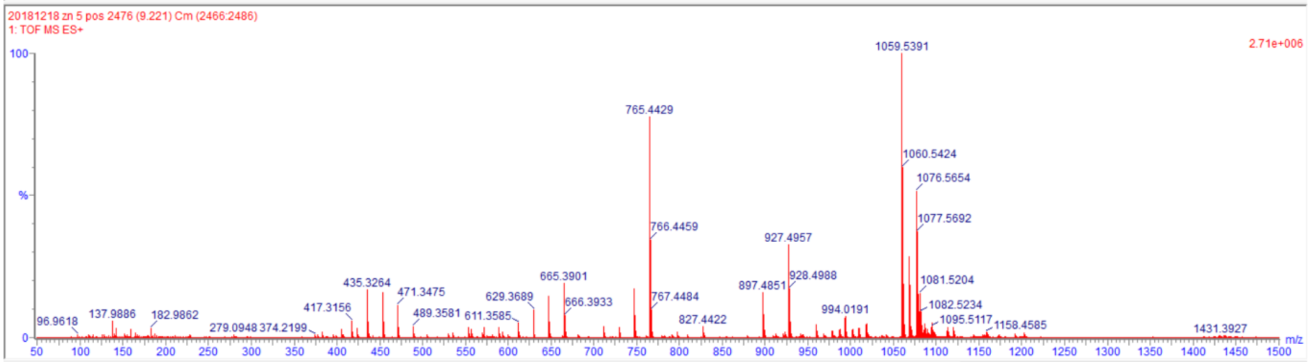

S157. HRESIMS spectrum of the new compound **33**
